# Blood Activating and Stasis Removing Chinese Patent Medicine in Perioperative Period of Percutaneous Coronary Intervention for Myocardial Infarction: A Protocol for a Living Clinical Practice Guideline

**DOI:** 10.1002/gin2.70068

**Published:** 2026-04-01

**Authors:** Zumao Cao, Yongbo Wang, Xiaoyu Zhang, Lei Zhang, Zhicong Zeng, Fengxia Lin, Hairong Wang, Jingyu Huang, Chong Zou, Zhengan Bi, Yinghui Jin, Hongcai Shang, Romina Brignardello‐Petersen

**Affiliations:** ^1^ Key Laboratory of Internal Medicine of Traditional Chinese Medicine of Ministry of Education and Beijing Beijing University of Chinese Medicine Beijing China; ^2^ Dongzhimen Hospital Beijing University of Chinese Medicine Beijing China; ^3^ Center for Evidence‐Based and Translational Medicine Zhongnan Hospital of Wuhan University Wuhan China; ^4^ Institute of Basic Research in Clinical Medicine China Academy of Chinese Medical Sciences Beijing China; ^5^ Institute of Information on Traditional Chinese Medicine China Academy of Chinese Medical Sciences Beijing China; ^6^ Shenzhen Bao'an District Traditional Chinese Medicine Hospital Shenzhen China; ^7^ Department of Cardiology Zhongnan Hospital of Wuhan University Wuhan China; ^8^ Department of Thoracic Surgery Zhongnan Hospital of Wuhan University Wuhan China; ^9^ Daye Hospital of Traditional Chinese Medicine Daye China; ^10^ Jiangsu Provincial Hospital of Traditional Chinese Medicine Nanjing China; ^11^ Hubei Provincial Hospital of Traditional Chinese Medicine Wuhan China; ^12^ Dongfang Hospital Beijing University of Traditional Chinese Medicine Beijing China; ^13^ Department of Health Research Methods, Evidence, and Impact McMaster University Hamilton Ontario Canada

**Keywords:** Chinese patent medicines, guideline, myocardial infarction, percutaneous coronary intervention, protocol

## Abstract

**Background:**

Myocardial infarction (MI) remains a major global health burden, with percutaneous coronary intervention (PCI) as the primary revascularization strategy. However, complications such as no‐reflow, reperfusion injury and in‐stent restenosis persist, requiring adjunctive therapies. Traditional Chinese Medicine (TCM), especially blood‐activating and stasis‐resolving Chinese patent medicine (CPM), is increasingly used in MI management.

**Objective:**

To develop a living clinical practice guideline for the perioperative use of CPMs in the perioperative period of PCI for MI, based on rigorous and transparent methodology.

**Methods:**

The guideline followed the WHO Handbook for Guideline Development, methods for developing integrated Chinese–Western medicine guidelines, and the living guideline framework. It will be reported in accordance with the RIGHT‐TCM extension. Clinical questions and outcomes were framed, selected and rated through structured discussions with the Guideline Steering Committee and the Guideline Development Group. A systematic review and Bayesian network meta‐analysis will evaluate the efficacy and safety of CPM. The certainty of evidence will be assessed using the GRADE approach, and recommendations will be formulated via the Evidence‐to‐Decision framework, incorporating benefits, harms, evidence certainty, feasibility and costs. When new clinical studies are included and critical outcomes' combined effect demonstrates directional or magnitude changes, the consensus and guideline development groups will jointly decide on new updates.

**Conclusion:**

By addressing key clinical uncertainties and leveraging real‐time evidence updates, the guideline aims to enhance patient outcomes and support clinicians in decision‐making.

**Trial Registration:** Guideline Registration Number: PREPARE‐2024CN596

## Summary

- It rigorously adheres to the WHO Handbook, the living guideline framework and the RIGHT‐TCM extension standards.
- The guideline utilizes Bayesian network meta‐analysis to evaluate the efficacy and safety of 47 blood‐activating and stasis‐resolving Chinese patent medicines.
- Recommendations will be formulated via the GRADE approach and Evidence‐to‐Decision (EtD) framework.
- A living update mechanism driven by continuous evidence retrieval and the MAGICapp platform ensures recommendations remain current and clinically relevant.

## 1 Introduction

Myocardial infarction (MI), commonly known as a heart attack, results from an abrupt reduction or complete blockage of coronary blood flow, leading to ischaemic necrosis of myocardial tissue [1]. According to the 2017 Global Burden of Disease study, the global incidence of acute myocardial infarction (AMI) was 106.37 per 100,000 population [2], making it one of the leading causes of death worldwide [3]. In China, AMI‐related mortality has been rising [4], reaching 60.29 per 100,000 in urban and 78.65 in rural areas in 2020 [5]. With an aging population, the burden of MI is expected to increase, with annual hospitalization costs exceeding RMB 34.7 billion.

Timely reperfusion therapy remains the cornerstone of MI treatment [6]. Among available methods, percutaneous coronary intervention (PCI, a procedure using percutaneous puncture to restore coronary blood flow via balloon catheters or devices) [7] is widely preferred due to its convenience, efficacy and low risk [8]. However, challenges such as no‐reflow, ischaemia–reperfusion injury, perioperative myocardial injury, in‐stent restenosis and stent thrombosis can compromise outcomes and long‐term survival [9]. Hence, exploring adjunctive treatments to enhance PCI efficacy and improve quality of life is critical.

In recent years, Traditional Chinese Medicine (TCM), particularly blood‐activating and stasis‐resolving Chinese patent medicine (CPMs), has been increasingly integrated into MI management. Between 2001 and 2011, TCM use rose from 78% to 82% in rural areas and from 44% to 61% in urban areas [10]. In TCM theory, MI falls under categories such as ‘chest impediment’, ‘heart pain’ and ‘true heart pain’, characterized by chest pain as the primary symptom. Its pathogenesis is often attributed to deficiencies in qi, blood, yin and yang, accompanied by obstruction of heart meridians by pathological factors like phlegm and blood stasis—leading to stagnation of chest yang and disrupted qi flow, thereby driving disease development. Treatment centres on tonifying qi, promoting blood circulation, resolving phlegm and unblocking meridians as core strategies.

Modern management of MI involves dual antiplatelet therapy as a core antithrombotic strategy. However, individual variability in response and long‐term side effects limit its universal effectiveness. TCM offers multi‐target interventions that may complement conventional therapies. Studies have shown that CPMs exert effects on platelet aggregation, blood flow, oxidative stress and vascular function [11, 12, 13], highlighting their potential as adjunctive treatments. Emerging evidence supports their efficacy and safety in MI, especially when applied at specific disease stages [14, 15, 16, 17].

Clinical practice guidelines provide optimal guiding recommendations tailored for specific clinical concerns, grounded in systematic evaluations of evidence and comprehensive analyses of the pros and cons of various alternative interventions [18]. Compared to traditional guidelines, living guidelines incorporate new evidence in real time, maintaining clinical relevance [19]. Given the increasing demand for CPMs during the perioperative PCI period and the need to standardize their use, it is imperative to develop a living guideline for their application. This guideline aims to provide clear, evidence‐based recommendations, integrate with clinical workflows, and serve as a reference both nationally and internationally.

## 2 Objectives

We aim to develop a guideline for the use of TCM in MI PCI perioperative care, with the following objectives:

1. To evaluate the efficacy and safety of CPMs in the treatment of MI patients during the perioperative period of PCI, and to provide recommendations for integrating TCM into clinical practice.
2. To optimize the utilization of CPM resources and enhance the efficiency of the healthcare system through the formulation and implementation of guidelines, thereby reducing the economic burden of treatment.

## 3 Methods

**Figure 1:**
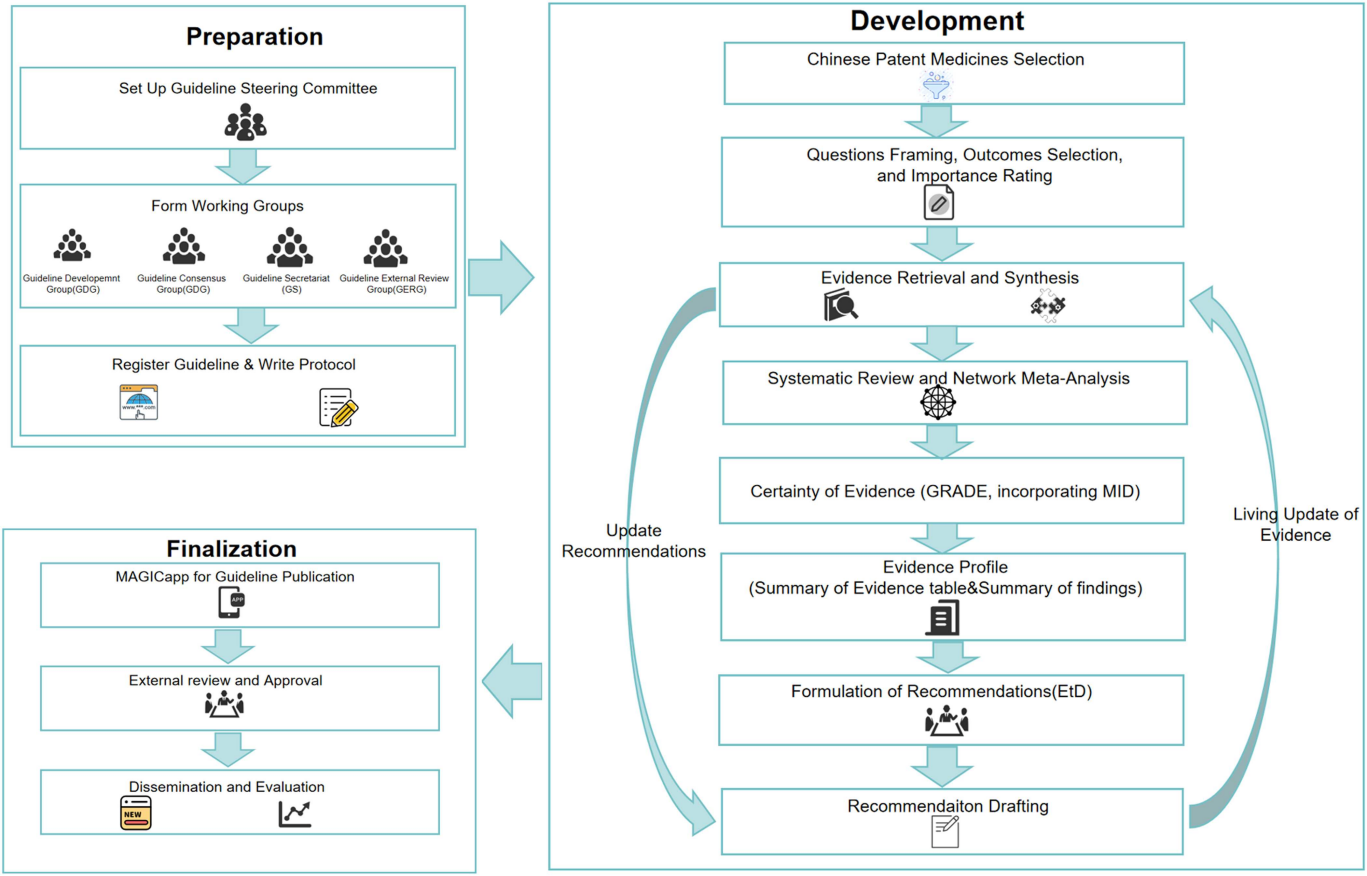
Living guideline development flowchart.

The guideline protocol and final guideline will be prepared based on the WHO Handbook for Guideline Development [20], the Methods and Procedures for Developing Integrated Chinese and Western Medicine Clinical Practice Guidelines [21] and the Methods for Developing Living Guidelines [22], and will be reported in accordance with the relevant requirements of The RIGHT Extension Statement for Traditional Chinese Medicine [23]. This protocol follows established methodologies for guideline development, ensuring rigour, transparency and applicability. A visual representation of our approach can be found in Figure 1.

### 3.1 Guideline Development Organization

This guideline is supported by the National Key R&D Program of China (2017YFC1700402) and the Qihuang Scholars Program of ‘Thousand Million’ Talents Project (Qihuang Project) for Chinese Medicine Inheritance and Innovation. Participating institutions include Dongzhimen Hospital, Beijing University of Chinese Medicine; Dongfang Hospital, Beijing University of Traditional Chinese Medicine, Beijing, China; Baoan District Hospital of Traditional Chinese Medicine, Shenzhen; Zhongnan Hospital of Wuhan University.

### 3.2 Guideline Registration

The guideline has been registered in both Chinese and English on the Practice guideline REgistration for transPAREnCy (PREPARE) (https://www.guidelines-registry.cn) with a registration number of PREPARE‐2024CN596.

### 3.3 Guideline Working Groups

The development of this guideline involved five working groups: the Guideline Steering Committee (GSC), Guideline Development Group (GDG), Guideline Consensus Group (GCG), Guideline Secretariat and Guideline External Review Group. These groups include experts from TCM, WM, evidence‐based medicine and related fields. They are responsible for guiding, developing, reviewing, reaching consensus, documenting and externally evaluating the guideline, which ensures its scientific rigour, methodological integrity and practical applicability. Details regarding the composition and responsibilities of each group are provided in Supporting Information S1: File 1.

### 3.4 Declaration of Interests

To ensure fairness and transparency, all participants must complete a Declaration of Interests Form (see Supporting Information S2: File 2) and disclose any direct or indirect interests that may compromise their objectivity. All COIs will be declared upfront and reviewed by the GSC to ensure proper management. COI records will be published alongside the guidelines and updated annually.

Detailed definitions, classifications and management procedures of COI are provided in Supporting Information S3: File 3.

### 3.5 Determination of Disease Naming

Clarification of disease nomenclature is crucial for maintaining consistency and facilitating effective communication between TCM and modern Western medicine. As TCM does not employ the term ‘myocardial infarction’ directly, mapping traditional disease patterns to modern clinical diagnoses enhances the relevance and applicability of the guideline, particularly in the context of integrative care.

**Figure 2:**
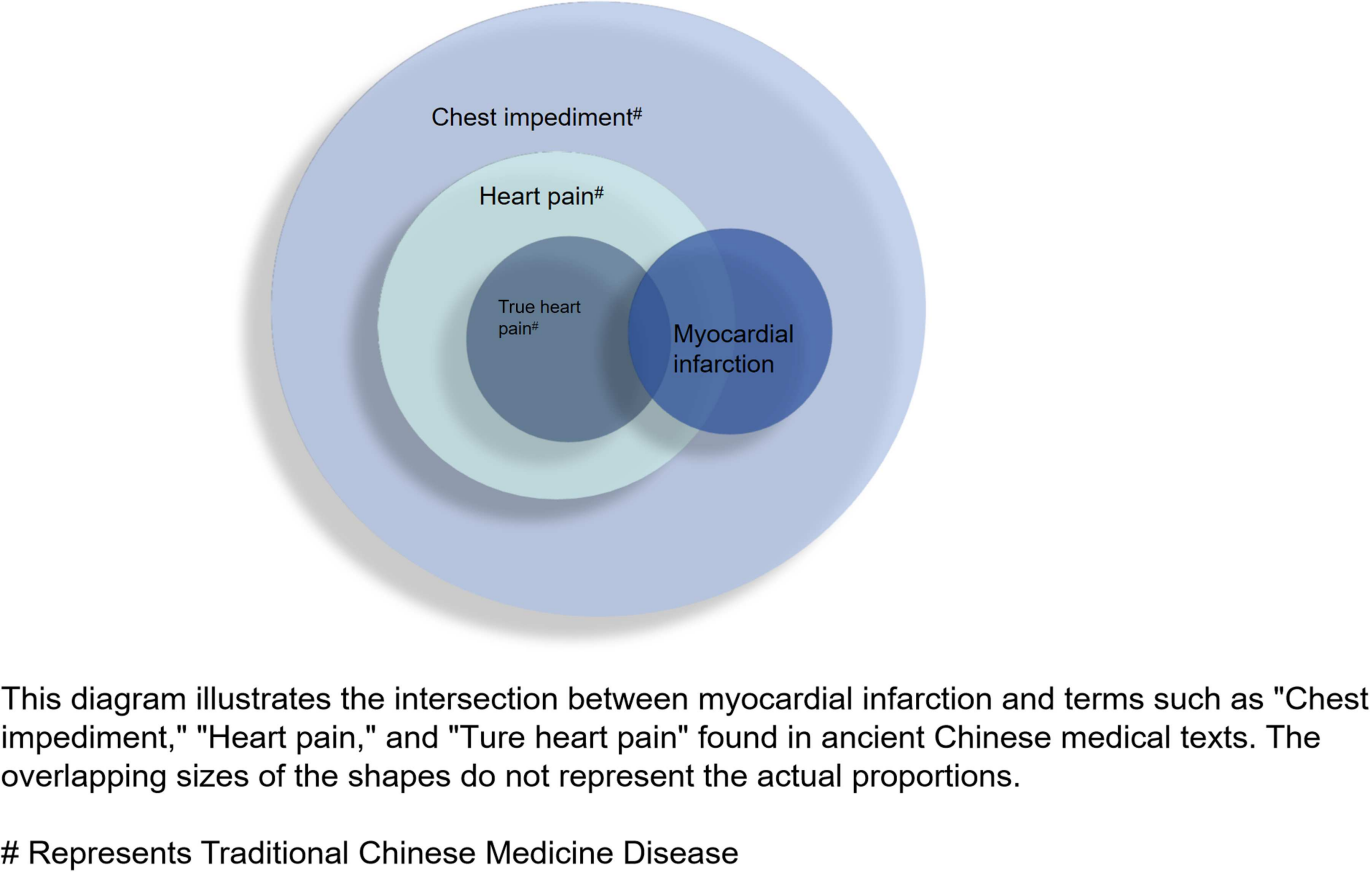
Relationship between myocardial infarction and TCM disease terms.

Although TCM does not explicitly use the modern medical term ‘myocardial infarction’, its descriptions of symptoms such as chest pain, chest tightness and palpitations bear significant similarity to the clinical manifestations of MI. By comparing descriptions of medical conditions such as Chest Impediment, Heartache and True Heartache in ten ancient texts including the *Huangdi Neijing* (The Yellow Emperor's Classic of Internal Medicine), *JinGui Yaolue* (Essentials from the Golden Cabinet) and *Danxi Xinfa* (Danxi′s Mastery of Medicine), a graphical representation of their relationship has been created in Figure 2. Following in‐depth discussions and consensus among the GSC, it was decided to adopt the Western medical term ‘myocardial infarction’ in this guideline to facilitate its promotion and use, especially in Western medical settings. Additionally, six TCM syndrome differentiations for MI have been adopted, including ‘qi deficiency and blood stasis’, ‘phlegm and blood stasis mutual obstruction’, ‘qi stagnation and blood stasis’, ‘cold congealing in the heart meridian’, ‘qi and yin deficiency’ and ‘syndrome of qi deficiency and yang collapse’ [24].

### 3.6 Target Audience for the Guidelines

The guidelines are applicable to patient populations who have been definitively diagnosed with MI (including ST‐segment elevation myocardial infarction (STEMI) and non‐ST‐segment elevation myocardial infarction (NSTEMI)); the target users are clinical practitioners of Traditional Chinese Medicine, Western Medicine and integrated Chinese and Western Medicine engaged in the treatment of MI.

### 3.7 Integration of Traditional Chinese and WM

This guideline adheres to the treatment principle of ‘disease differentiation as the primary consideration, syndrome differentiation as a supplement’, representing a ‘combination of WM diseases and TCM syndrome differentiation’. ‘Disease differentiation’ entails the clear diagnosis of MI by WM, while ‘syndrome differentiation’ involves identifying specific TCM syndromes in patients. In this framework, the WM diagnosis underpins the treatment, ensuring the specificity and scientific rigour of therapy.

### 3.8 Selection of CPMs

**Figure 3:**
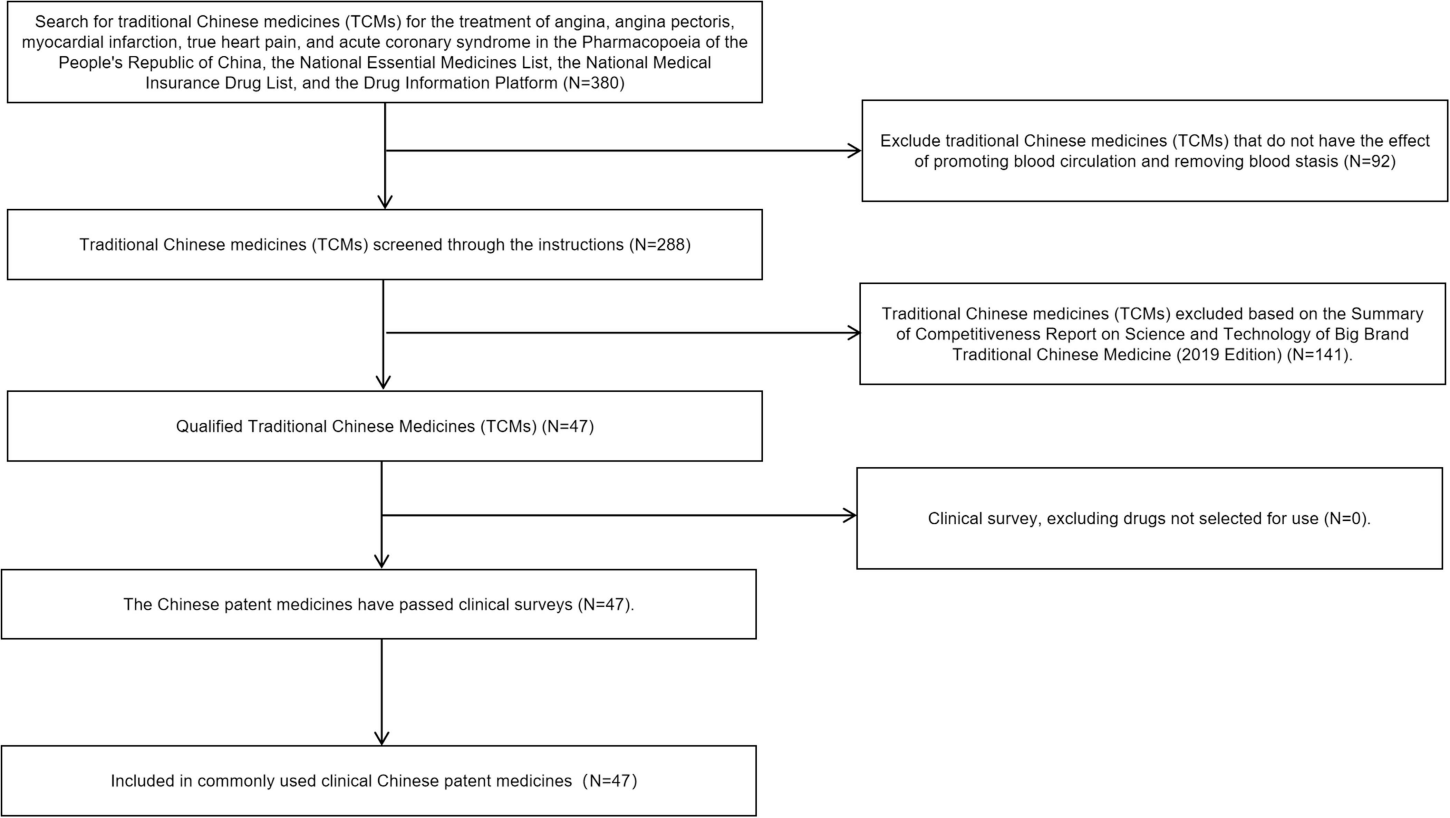
Screening workflow of Chinese patent medicines.

To ensure the scientific validity and clinical relevance of the included CPMs, GDG adopted a multi‐step selection strategy that integrated authoritative sources, brand competitiveness data and expert clinical input. A total of 47 high‐priority CPMs were ultimately selected for evaluation. For details, see Supporting Information S4 and S5: Files 4 and 5; the selection process is illustrated in Figure 3.

### 3.9 Framing Questions, Selecting Outcomes and Rating Importance

Clinicians performing PCI procedures were recruited through convenience sampling to complete a questionnaire that used a Likert scale (with five being the most important and one the least, indicating clinical insignificance). Their responses were subsequently evaluated by the GDG. Through two Delphi rounds, questions were retained only if they met both criteria: mean score > 4.0 and coefficient of variation < 0.25, ensuring strong consensus. These questions were then confirmed by the GSC. Based on the previously established Core Outcome Set for MI and the review of relevant literature [25], the GDG used the GRADE Guidelines to assess the importance of each outcome, with scores of 7–9 indicating critical outcomes, 4–6 indicating important outcomes and 1–3 indicating general outcomes, and the final outcomes were confirmed by the GSC. The final questions and outcomes are detailed in Supporting Information S6: File 6.

### 3.10 Evidence Retrieval

Our evidence retrieval focuses on the efficacy and safety of blood‐activating and stasis‐resolving CPMs in the perioperative period of PCI for MI. Due to the patient perspective of the guidelines, this review did not include cost‐effectiveness or other economic‐related factors. Given that the majority of these medicines are currently covered by the health insurance system, cost is not expected to play a major role in shaping the recommendations. This limitation will be clearly stated in the guideline to ensure transparency in the evidence review process.

#### 3.10.1 Retrieval of Resources

Systematic searches have been conducted in English literature databases, including PubMed, Embase, the Cochrane Library and Web of Science, as well as Chinese literature databases, including CNKI, WanFang Data and VIP. The search covered the period from the inception of the databases to 16 March 2024, with an updated search extending through 15 June 2025. The search language was limited to English or Chinese.

#### 3.10.2 Search Terms

Search terms included ‘myocardial infarction (myocardial infarction, myocardial infarct*, heart infarct*, etc.)’, ‘acute coronary syndrome (Acute Coronary Syndrome, Acute Coronary Syndrome*, NSTE‐ACS, etc.)’, ‘PCI (percutaneous coronary intervention, percutaneous coronary intervention*, percutaneous coronary revascularization*, etc.)’ and ‘CPM (danhong injection, danhong, etc.)’. See Supporting Information S7: File 7 for details of search terms.

#### 3.10.3 Inclusion and Exclusion Criteria

The inclusion criteria were established based on predefined questions, ensuring that the scope of evidence synthesis aligned precisely with the needs of the guideline.

This systematic review included only randomized controlled trials (RCTs). It included patients diagnosed with AMI, encompassing both STEMI and NSTEMI, according to Western diagnostic guidelines. There were no restrictions regarding participants' gender, geographic location, ethnicity or referral source (e.g., hospitals, outpatient clinics, community health centres, specialized cardiac care facilities, or referrals from general practitioners, specialists, or emergency departments). Additionally, there were no constraints on the disease duration.

The intervention group received standard care along with 1 of the 47 selected CPMs, while the control group received standard treatment, with the option of also using any of the 47 CPMs. Both groups were allowed to receive standard co‐interventions, including general care (e.g., monitoring vital signs and providing symptom relief), PCI and any form of pharmacotherapy (such as antiplatelet agents, anticoagulants, lipid‐lowering drugs and antihypertensive medications). The classification of interventions was determined by the predefined questions, focusing on the specific drug rather than dosage variations, to ensure consistency in the evidence synthesis. Western medications were included at any dosage within the standard treatment range, while CPMs were included only if their dosages adhered to package insert recommendations.

#### 3.10.4 Literature Quality Assessment

Appropriate evaluation tools will be selected based on the study design to assess the quality of included literature: RoB (Risk of Bias) 2.0 tool [26] for RCTs. The risk of bias will be evaluated across the following five domains: (1) bias arising from the randomization process, (2) bias due to deviations from intended interventions, (3) bias due to missing outcome data, (4) bias in the measurement of outcomes and (5) bias in the selection of reported results. Each domain will be categorized as ‘low risk of bias’, ‘some concerns’, or ‘high risk of bias’. The overall risk of bias for each study will be determined as follows: studies will be classified as ‘low risk of bias’ if all domains are judged to have a low risk of bias, ‘some concerns’ if at least one domain raise some concerns but no domain is rated as high risk, and ‘high risk of bias’ if at least one domain is rated as high risk or if multiple domains raise some concerns. Disagreements between reviewers will be resolved through discussion or consultation with a third reviewer.

#### 3.10.5 Search Frequency

Based on the update frequency of the research field and the needs of clinical practice, searches will be updated every 6 months and continued for 3 years.

#### 3.10.6 Study Selection

After removing duplicates using EndNote software, two reviewers will independently screen the titles and abstracts of the retrieved records against the inclusion criteria. Potentially eligible studies will be retrieved for full‐text review. Two reviewers will then independently assess the full texts to decide on final inclusion. Any reasons for exclusion at the full‐text stage will be recorded. Disagreements between the two reviewers will be resolved through discussion or, if necessary, by consulting a third reviewer. The study selection process will be documented in a PRISMA flow diagram.

#### 3.10.7 Data Extraction and Management

Two reviewers will independently extract data from the included studies using a standardized, pilot‐tested data extraction form. For continuous outcomes, if means and standard deviations (SDs) are reported, they will be extracted directly. If SDs are missing, we will calculate them from standard errors, credible intervals (CrIs), or *t* values/*p* values [27], or estimate them from medians and ranges/interquartile ranges [28]. Discrepancies will be resolved by consensus or adjudication by a third reviewer. The following information will be extracted:

1. Study characteristics: author, year of publication, study design, sample size and duration of follow‐up.
2. Participant characteristics: age, sex, type of MI (STEMI/NSTEMI), comorbidities and baseline cardiac function.
3. Intervention and comparator details: specific CPM used, dosage, frequency, duration of treatment and details of standard care/co‐interventions.
4. Outcomes: data on primary and secondary outcomes (e.g., number of events for MACE, bleeding).

#### 3.10.8 Effect Measures

For dichotomous outcomes (e.g., incidence of MACE, mortality, bleeding), we will use the Relative Risk (RR) with its 95% CrI as the effect measure. For continuous outcomes (if applicable), we will use the Mean Difference or Standardized Mean Difference with 95% CrIs.

### 3.11 Evidence Synthesis for Guideline Development

#### 3.11.1 Network Meta‐Analysis

As part of the guideline development process, the GDG will conduct a network meta‐analysis (NMA) to address all predefined questions [29, 30]. We will employ a Bayesian random‐effects model for all comparisons. We will assume a common heterogeneity parameter (*τ* ^2^) across all treatment comparisons. Global heterogeneity will be quantified by estimating the posterior median and 95% CrIs of the between‐study variance (*τ* ^2^). Regarding the assessment of inconsistency (incoherence), our network is expected to be ‘star‐shaped’ (i.e., lacking closed loops), where each treatment strategy is compared solely with a common comparator (standard care). Consequently, formal statistical assessment of inconsistency, such as node‐splitting, is not applicable.

We will perform the NMA using R software version 4.2.2 (R Foundation for Statistical Computing) with the BUGSnet package. This model will be used for all comparisons with default priors, producing estimated treatment effects for each comparison along with 95% CrIs. By default, independent normal priors will be used with a mean of 0 and SD of 15 u (where u is the largest maximum likelihood estimator in single trials), yielding 200,000 iterations with 5000 burn‐in iterations, and a thinning interval of 10 will be used to calculate the posterior distributions of the model parameters. Additionally, model fit will be assessed by comparing the total residual deviance with the number of data points. A network diagram will be used to illustrate the direct relationships between all interventions. The lines between nodes represent direct comparisons between interventions, with the thickness of the line indicating the inverse of the variance of the direct estimate, reflecting the precision of the evidence. The size of the nodes represents the number of participants allocated to the treatment. Finally, the Surface Under the Cumulative Ranking curve will be employed to rank the different interventions.

#### 3.11.2 Network Meta‐Regression and Subgroup Analyses

Network meta‐regression and subgroup analyses will be applied to address different analytical objectives. Meta‐regression will be used to control for potential effect modifiers—such as MI type (ST‐elevation, non‐ST‐elevation), dosage form (pills, tablets, injections, capsules) and TCM syndromes (phlegm and blood stasis mutual obstruction, Qi deficiency and blood stasis, Qi and Yin deficiency, Qi stagnation and blood stasis, or unspecified)—to reduce intransitivity in treatment effects. We will present the coefficients and predictive effects of analyses involving a minimum of six trials. In contrast, subgroup analyses are exploratory and focus on quantifying treatment effects within each subgroup and comparing these effects across subgroups. Specifically, we will conduct exploratory subgroup analyses using network meta‐regression. We will estimate treatment effects for specific subgroup levels to explore potential sources of heterogeneity and inconsistency, rather than making definitive clinical predictions. All subgroup factors were predefined, and details of these factors, including their classifications, are provided in Supporting Information S8: File 8. We will not adjust for multiple comparisons in these exploratory analyses. We will also conduct a sensitivity analysis to assess the robustness of the findings by excluding studies assessed as having a high risk of bias.

#### 3.11.3 Determining the Minimum Important Difference

To elucidate whether the intervention has an important impact on specific outcomes within the perioperative period of PCI for MI, we will conduct a patient values and preferences survey to determine the minimum important difference (MID) for outcomes [31]. The survey questionnaire includes five outcomes (MACE, MACCE, TIMI, major bleeding and minor bleeding). Based on the extent of the intervention's impact on the perioperative period following post‐MI PCI, eight scenarios are set for each outcome. For beneficial outcomes (MACE, MACCE, TIMI), the first scenario is ‘In patients who undergo percutaneous coronary intervention after MI, the use of blood activating and stasis removing CPM combined with standard treatment reduces the incidence of outcome events by 1/1000 (i.e., one fewer event per 1000)’. It should be noted that for the Number of patients achieving TIMI Grade 3 flow, the change in incidence represents an increased effect. Subsequently, in the following scenarios, the change in outcome events is 20, 3, 15, 5, 10, 8 and 12/1000 (a ping‐pong method from one extreme to another, gradually narrowing the difference) [31]. In each scenario, the survey asks experts to infer the proportion of patients who consider the impact on a specific outcome event to be important. Options include the following proportions: almost all patients; most patients, majority of patients, minority of patients, few patients, very few patients, almost no patients. When an expert changes their answer from ‘almost all patients consider this reduction in risk to be important’ to ‘almost no patients consider this reduction in risk to be important’ (or vice versa), the survey has determined the narrow range in which the MID lies. For risk outcomes (major bleeding and minor bleeding), the first scenario is ‘In patients who undergo percutaneous coronary intervention after a myocardial infarction, the use of blood activating and stasis removing CPM combined with standard treatment increases the incidence of outcome events by 1/1000 (i.e., one additional event per 1000)’. The change in outcome events in the following scenarios is the same as for beneficial outcomes. The subsequent enquiry method is also the same as for beneficial outcomes. Subsequently, we distributed the questionnaire to experts in the relevant field via an online survey tool for completion, including clinical physicians, methodological experts and pharmacological experts. We will finally describe the results in terms of distribution giving the median and range.

### 3.12 Certainty of Evidence

The Grading of Recommendations Assessment, Development and Evaluation (GRADE) approach will be used to assess the certainty of the evidence for all outcomes [32, 33]. Evidence from direct comparisons will start as high certainty evidence and could be down rated for risk of bias, inconsistency (heterogeneity), indirectness and publication bias [34]. Evidence from indirect comparisons could be further down rated for intransitivity [32, 35]. The results for the treatment effects will be derived from the initial NMA. We will assess intransitivity by comparing the distributions of potential effect modifiers, specifically the three factors included in the meta‐regression: MI type, dosage form and TCM syndromes, across direct comparisons and outcomes. This assessment will be supplemented by meta‐regressions evaluating the relationship between these modifiers and treatment effects. The final certainty of the network estimate could be down rated for incoherence and imprecision [32]. We will rate imprecision following the GRADE guidance [36, 37]. When point estimates prove less than the MID, we will rate certainty in little or no effect, otherwise in non‐zero effect (i.e., null effect threshold). We will down rate for imprecision by two levels when the 95% CrI crosses more than one threshold of importance [38].

To classify the relative impact of interventions, we will use the null effect as the decision threshold and standard care as the reference intervention [35]. Treatments will be first categorized as either different or not different from the standard treatments. Subsequently, they will be further categorized based on whether they differ from at least one treatment already identified as distinct from the standard treatments. This iterative process will result in five categories, ranking interventions from the most effective to the least effective. Interventions will be then stratified by the certainty of evidence, distinguishing between high or moderate versus low or very low certainty, relative to the standard treatments [39, 40].

### 3.13 Summary of Evidence Table

To improve interpretability of benefits and harms, our summary of evidence will include both relative and absolute effect sizes. We will estimate the baseline risk (i.e., the control group event rate) by conducting a random‐effects single‐arm meta‐analysis of proportions using data (number of events and sample sizes) extracted from the standard care arms of eligible trials. To ensure robust estimation, particularly for outcomes with low event rates, we will employ a Generalized Linear Mixed Model. Absolute Risk Reduction (ARR) or Increase (ARI) will be derived from RRs and the estimated baseline risk [41]. Only trials with standard care‐only groups will be included. We will also calculate the Number Needed to Treat (NNT/NNH per 1000 = 1000 × ARR/ARI) [42]. The full summary of evidence is provided in Supporting Information S9: File 9.

### 3.14 Formulation of Recommendations

The recommendations for each CPM will be based on the results of the NMA and the summary of findings tables (Supporting Information S10: File 10). We will employ the GRADE Evidence to Decision (EtD) framework (Supporting Information S11: File 11) and the Nominal Group Technique to reach expert consensus through structured anonymous voting. The strength of recommendations will be categorized as strong or weak for and against. Detailed voting procedures and decision criteria will be provided in Supporting Information S12: File 12.

### 3.15 Living Updates

Evidence retrieval will be conducted every 6 months by the GDG. When new evidences are incorporated and the combined effect of critical outcomes shows a directional change or magnitude, a new round of guideline updates will be initiated. This decision will be made jointly by the GCG and the GDG.

### 3.16 External Review of Guideline Recommendations and Guideline Reports

Guideline recommendations will be externally reviewed by experts and publicly posted for feedback. The GDG will review all input and finalize the guidelines. If revisions are needed, the issue will be referred to the GCG for discussion and decision. Final recommendations will be drafted in accordance with the RIGHT Extension Statement for Traditional Chinese Medicine [23] and submitted to the GSC for approval.

### 3.17 Guidelines Publication and Updates

The guideline will be edited and developed on MAGICapp (https://app.magicapp.org/#/guidelines), a platform specifically designed for creating and managing clinical practice guidelines. MAGICapp supports the development of evidence‐based guidelines by providing a structured and systematic approach. It allows for collaboration among experts, facilitates the integration of real‐time evidence and supports the living updating of recommendations. Through this platform, we can ensure that the guideline incorporates the latest research, expert consensus and clinical best practices.

The use of MAGICapp also enhances transparency in the guideline development process, as it allows for the clear documentation of the evidence, expert opinions and decision‐making processes that led to the final recommendations. This feature is crucial for fostering trust and ensuring that the guideline reflects the best possible practices in the field of MI treatment during PCI.

## 4 Discussion

CPM, deeply rooted in TCM, not only reflects millennia of medical wisdom in China but also enjoys widespread usage due to its simplicity and high patient acceptance. With the growing influence of evidence‐based medicine, CPM is gaining recognition in the global market. Some CPMs have been registered as over‐the‐counter drugs in the European Union, Russia and Canada, and have undergone clinical trials approved by the FDA [43]. As of 2021, the annual revenue of CPM had soared to 486.2 billion yuan, underscoring its significant and expanding market potential [44].

The compound formulations of CPM, through the synergistic effects of multiple components, provide a multi‐targeted, holistic treatment approach for MI patients. This approach improves patient symptoms and prognosis, offering more treatment options while enhancing the specificity and effectiveness of therapy [45]. However, current integrated Chinese and Western medicine guidelines often merely list treatment approaches without fully utilizing the strengths of both systems. Additionally, these guidelines suffer from long update cycles, unclear methods and lack of clear procedures, limiting their applicability in rapidly evolving clinical practice [21, 46]. The traditional fixed‐cycle update model for clinical guidelines has inherent limitations, as it cannot ensure the timeliness of all recommendations. Some may become outdated quickly, while others remain valid for a long period. Research shows that the median ‘survival’ of systematic reviews is only 2.9 years [47], meaning that guideline recommendations based on these reviews may already be outdated at publication [48, 49, 50].

This protocol has several strengths, including the adoption of a living guideline framework to ensure currency and the rigorous adherence to GRADE and RIGHT‐TCM standards. However, potential limitations must be acknowledged. First, as a protocol, this study focuses on methodological design rather than reporting empirical clinical results; the rigorous execution of the proposed methods will determine the final guideline's quality. Second, we anticipate that direct head‐to‐head comparisons between different CPM may be scarce, resulting in a ‘star‐shaped’ network centred on standard care. This network structure precludes the assessment of statistical inconsistency (incoherence) between direct and indirect evidence. Consequently, the relative efficacy of different CPM will primarily rely on indirect comparisons, which may result in wider CrIs and lower certainty of evidence for some comparisons. We will address this by carefully assessing the transitivity assumption and strictly applying GRADE criteria to rate the certainty of network estimates. Third, this protocol excludes cost‐effectiveness analysis due to the general scarcity of comparative pharmacoeconomic data across the 47 included interventions. We acknowledge that cost is a critical factor for implementation. However, in the primary setting of this guideline (China), the majority of these medicines are covered by national health insurance, meaning out‐of‐pocket costs are not a primary barrier for most patients. For international settings where these medicines are not covered by health insurance, stakeholders will need to consider local economic feasibility alongside our clinical recommendations.

This protocol explores new methodological approaches in integrating Chinese and Western Medicine guidelines, including living updates, multi‐intervention comparisons through NMA and collaboration with the MAGIC organization. Its goal is to rigorously and comprehensively assess the efficacy and safety of blood‐activating and stasis‐resolving CPMs during the perioperative period of PCI, ultimately promoting the rational and appropriate use of CPMs.

## Author Contributions

**Zumao Cao:** conceptualization, methodology, writing – original draft. **Yongbo Wang:** conceptualization, methodology, writing – original draft. **Xiaoyu Zhang:** investigation, methodology. **Lei Zhang:** investigation, methodology. **Zhicong Zeng:** investigation, methodology. **Fengxia Lin:** investigation, methodology. **Hairong Wang:** investigation, methodology. **Jingyu Huang:** investigation, methodology. **Chong Zou:** investigation, methodology. **Zhengan Bi:** investigation, methodology. **Romina Brignardello‐Petersen:** methodology, supervision, review and editing. **Yinghui Jin:** conceptualization, methodology, supervision, project administration, review and editing. **Hongcai Shang:** conceptualization, methodology, supervision, project administration, review and editing. All authors read and approved the final manuscript. Zumao Cao and Yongbo Wang contributed equally to this work and share first authorship. Yinghui Jin and Hongcai Shang are the corresponding authors.

## Conflicts of Interest

The authors declare no conflicts of interest.

## Supporting information


**Supporting File 1:** Composition and responsibilities of guideline working groups.

**Supporting File 2:** Form for conflict of interest survey (not for publication).

**Supporting File 3:** Definition, classification and management of conflicts of interest.

**Supporting File 4:** Selection process of Chinese patent medicines for systematic evidence retrieval.

**Supporting File 5:** Survey questionnaire on the application of Chinese patent medicine in myocardial infarction.

**Supporting File 6:** Form of questions and outcomes.

**Supporting File 7:** Search strategy.

**Supporting File 8:** Subgroup details table.

**Supporting File 9:** Evidence summary table.

**Supporting File 10:** Summary of findings table.

**Supporting File 11:** Evidence to decision table.

**Supporting File 12:** Voting procedures and decision criteria for formulating recommendations.

## Data Availability

Data sharing is not applicable to this article as no data sets were generated or analyzed during the current study.

## References

[1] K. Thygesen , J. S. Alpert , A. S. Jaffe , et al., “Fourth Universal Definition of Myocardial Infarction (2018),” Circulation 138, no. 20 (2018): e618–e651. 30571511 10.1161/CIR.0000000000000617

[2] GBD 2017 Disease and Injury Incidence and Prevalence Collaborators , “Global, Regional, and National Incidence, Prevalence, and Years Lived With Disability for 354 Diseases and Injuries for 195 Countries and Territories, 1990‐2017: A Systematic Analysis for the Global Burden of Disease Study 2017,” Lancet 392, no. 10159 (2018): 1789–1858. 30496104 10.1016/S0140-6736(18)32279-7 PMC6227754

[3] GBD 2021 Causes of Death Collaborators , “Global Burden of 288 Causes of Death and Life Expectancy Decomposition in 204 Countries and Territories and 811 Subnational Locations, 1990‐2021: A Systematic Analysis for the Global Burden of Disease Study 2021,” Lancet 403, no. 10440 (2024): 2100–2132. 38582094 10.1016/S0140-6736(24)00367-2 PMC11126520

[4] The Writing Committee of the Report on Cardiovascular Health and Diseases in China , “Report on Cardiovascular Health and Diseases in China 2021: An Updated Summary,” Journal of Geriatric Cardiology 20, no. 6 (2023): 399–430. 37416519 10.26599/1671-5411.2023.06.001 PMC10320777

[5] The Writing Committee of the Report on Cardiovascular Health and Diseases in China , “Report on Cardiovascular Health and Diseases in China 2022: An Updated Summary,” Biomedical and Environmental Sciences 36, no. 8 (2023): 669–701. 37711081 10.3967/bes2023.106

[6] H. Zhang and R. Chang , “Effects of Exercise After Percutaneous Coronary Intervention on Cardiac Function and Cardiovascular Adverse Events in Patients With Coronary Heart Disease: Systematic Review and Meta‐Analysis,” Journal of Sports Science & Medicine 18, no. 2 (2019): 213–222. 31191090 PMC6543998

[7] S. R. Kandan and T. W. Johnson , “Management of Percutaneous Coronary Intervention Complications,” Heart 105, no. 1 (2019): 75–86. 29760245 10.1136/heartjnl-2017-311155

[8] P. T. O'Gara , F. G. Kushner , D. D. Ascheim , et al., “ACCF/AHA Guideline for the Management of ST‐Elevation Myocardial Infarction: A Report of the American College of Cardiology Foundation/American Heart Association Task Force on Practice Guidelines,” Circulation 127, no. 4 (2013): e362–e425. 23247304 10.1161/CIR.0b013e3182742cf6

[9] J. Allencherril , H. Jneid , D. Atar , et al., “Pathophysiology, Diagnosis, and Management of the No‐Reflow Phenomenon,” Cardiovascular Drugs and Therapy 33, no. 5 (2019): 589–597. 31418141 10.1007/s10557-019-06901-0

[10] X. Li , K. Murugiah , J. Li , et al., “Urban‐Rural Comparisons in Hospital Admission, Treatments, and Outcomes for ST‐Segment‐Elevation Myocardial Infarction in China From 2001 to 2011: A Retrospective Analysis From the China PEACE Study (Patient‐Centered Evaluative Assessment of Cardiac Events),” Circulation: Cardiovascular Quality and Outcomes 10, no. 11 (2017): e003905, 10.1161/CIRCOUTCOMES.117.003905. 29158421 PMC6312853

[11] P. Liao , K. Chen , J. Ge , and M. Zhang , “Clinical Practice Guideline of Integrative Chinese and Western Medicine for Acute Myocardial Infarction,” Chinese Journal of Integrative Medicine 26, no. 7 (2020): 539–551. 30972537 10.1007/s11655-019-3154-z

[12] H. Liu , S. Wang , Y. Lei , and J. Shang , “Characteristics and Advantages of Traditional Chinese Medicine in the Treatment of Acute Myocardial Infarction,” Journal of Traditional Chinese Medicine 31, no. 4 (2011): 269–272. 22462229 10.1016/s0254-6272(12)60002-8

[13] J. Luo , H. Xu , and K. Chen , “Systematic Review of Compound Danshen Dropping Pill: A Chinese Patent Medicine for Acute Myocardial Infarction,” Evidence‐Based Complementary and Alternative Medicine 2013 (2013): 808076. 23843882 10.1155/2013/808076 PMC3703382

[14] M. Guo , P. Wang , J. Du , et al., “Xinyue Capsule in Patients With Stable Coronary Artery Disease After Percutaneous Coronary Intervention: A Multicenter, Randomized, Placebo‐Controlled Trial,” Pharmacological Research 158 (2020): 104883. 32446979 10.1016/j.phrs.2020.104883

[15] Z. Hu , H. Wang , G. Fan , et al., “Danhong Injection Mobilizes Endothelial Progenitor Cells to Repair Vascular Endothelium Injury via Upregulating the Expression of Akt, eNOS and MMP‐9,” Phytomedicine 61 (2019): 152850. 31035054 10.1016/j.phymed.2019.152850

[16] H. T. Zhang , Z. H. Jia , J. Zhang , et al., “No‐Reflow Protection and Long‐Term Efficacy for Acute Myocardial Infarction With Tongxinluo: A Randomized Double‐Blind Placebo‐Controlled Multicenter Clinical Trial (ENLEAT Trial),” Chinese Medical Journal 123, no. 20 (2010): 2858–2864. 21034597

[17] Y. Yang , X. Li , G. Chen , et al., “Traditional Chinese Medicine Compound (Tongxinluo) and Clinical Outcomes of Patients With Acute Myocardial Infarction: The CTS‐AMI Randomized Clinical Trial,” Journal of the American Medical Association 330, no. 16 (2023): 1534–1545. 37874574 10.1001/jama.2023.19524 PMC10599127

[18] Institute of Medicine , Clinical Practice Guidelines we can Trust (National Academies Press, 2011), 15. 24983061

[19] S. Cheyne , D. Fraile Navarro , K. Hill , et al., “Methods for Living Guidelines: Early Guidance Based on Practical Experience. Paper 1: Introduction,” Journal of Clinical Epidemiology 155 (2023): 84–96. 36639038 10.1016/j.jclinepi.2022.12.024

[20] WHO , WHO Handbook for Guideline Development (World Health Organization, 2011).

[21] Y. H. Jin , Y. P. Wang , Y. L. Xie , et al., “Research on the Development Methodology for Clinical Practice Guidelines for Organic Integration of Traditional Chinese and Western Medicine,” Military Medical Research 10, no. 1 (2023): 45. 37752599 10.1186/s40779-023-00481-9 PMC10523673

[22] E. A. Akl , J. J. Meerpohl , J. Elliott , et al., “Living Systematic Reviews: 4. Living Guideline Recommendations,” Journal of Clinical Epidemiology 91 (2017): 47–53. 28911999 10.1016/j.jclinepi.2017.08.009

[23] R. Xie , Y. Xia , Y. Chen , et al., “The RIGHT Extension Statement for Traditional Chinese Medicine: Development, Recommendations, and Explanation,” Pharmacological Research 160 (2020): 105178. 32889127 10.1016/j.phrs.2020.105178 PMC7462769

[24] Doctor Society of Integrative Medicine, Chinese Medical Doctor Association, Intensive Care Medicine Professional Committee of the Chinese Society of Integrative Medicine, et al ., “Diagnosis and Treatment Guideline of Integrative Chinese and Western Medicine for Acute Myocardial Infarction,” Chinese Journal of Integrated Traditional and Western Medicine 38, no. 3 (2018): 272–284.

[25] R. Qiu , S. Wan , C. Zhong , et al., “Core Outcome Sets for Myocardial Infarction in Clinical Trials of Traditional Chinese Medicine and Western Medicine,” Journal of Evidence‐Based Medicine 17, no. 1 (2024): 86–94. 38214702 10.1111/jebm.12579

[26] J. A. C. Sterne , J. Savović , M. J. Page , et al., “RoB 2: A Revised Tool for Assessing Risk of Bias in Randomised Trials,” BMJ 366 (2019): l4898. 31462531 10.1136/bmj.l4898

[27] J. P. T. Higgins , J. Thomas , J. Chandler , et al., eds., Cochrane Handbook for Systematic Reviews of Interventions, 2nd ed. (John Wiley & Sons, 2019).

[28] X. Wan , W. Wang , J. Liu , and T. Tong , “Estimating the Sample Mean and Standard Deviation From the Sample Size, Median, Range and/or Interquartile Range,” BMC Medical Research Methodology, 2014 14, no. 1 (2014): 135. 25524443 10.1186/1471-2288-14-135 PMC4383202

[29] A. E. Ades , N. J. Welton , S. Dias , D. M. Phillippo , and D. M. Caldwell , “Twenty Years of Network Meta‐Analysis: Continuing Controversies and Recent Developments,” Research Synthesis Methods 15, no. 5 (2024): 702–727. 38234221 10.1002/jrsm.1700

[30] G. Salanti , “Indirect and Mixed‐Treatment Comparison, Network, or Multiple‐Treatments Meta‐Analysis: Many Names, Many Benefits, Many Concerns for the Next Generation Evidence Synthesis Tool,” Research Synthesis Methods 3, no. 2 (2012): 80–97. 26062083 10.1002/jrsm.1037

[31] L. Zeng , L. M. Helsingen , M. Bretthauer , et al., “A Novel Framework for Incorporating Patient Values and Preferences in Making Guideline Recommendations: Guideline Panel Surveys,” Journal of Clinical Epidemiology 161 (2023): 164–172. 37453455 10.1016/j.jclinepi.2023.07.003

[32] M. A. Puhan , H. J. Schunemann , M. H. Murad , et al., “A GRADE Working Group Approach for Rating the Quality of Treatment Effect Estimates From Network Meta‐Analysis,” BMJ 349 (2014): g5630. 25252733 10.1136/bmj.g5630

[33] A. Izcovich , D. K. Chu , R. A. Mustafa , G. Guyatt , and R. Brignardello‐Petersen , “A Guide and Pragmatic Considerations for Applying GRADE to Network Meta‐Analysis,” BMJ 381 (2023): e074495. 37369385 10.1136/bmj-2022-074495

[34] H. Balshem , M. Helfand , H. J. Schünemann , et al., “GRADE Guidelines: 3. Rating the Quality of Evidence,” Journal of Clinical Epidemiology 64, no. 4 (2011): 401–406. 21208779 10.1016/j.jclinepi.2010.07.015

[35] R. Brignardello‐Petersen , G. Tomlinson , I. Florez , et al., “Grading of Recommendations Assessment, Development, and Evaluation Concept Article 5: Addressing Intransitivity in a Network Meta‐Analysis,” Journal of Clinical Epidemiology 160 (2023): 151–159. 37348573 10.1016/j.jclinepi.2023.06.010

[36] L. Zeng , R. Brignardello‐Petersen , M. Hultcrantz , et al., “GRADE Guidelines 32: GRADE Offers Guidance on Choosing Targets of GRADE Certainty of Evidence Ratings,” Journal of Clinical Epidemiology 137 (2021): 163–175. 33857619 10.1016/j.jclinepi.2021.03.026

[37] R. Brignardello‐Petersen , G. H. Guyatt , R. A. Mustafa , et al., “GRADE Guidelines 33: Addressing Imprecision in a Network Meta‐Analysis,” Journal of Clinical Epidemiology 139 (2021): 49–56. 34293434 10.1016/j.jclinepi.2021.07.011

[38] L. Zeng , R. Brignardello‐Petersen , M. Hultcrantz , et al., “GRADE Guidance 34: Update on Rating Imprecision Using a Minimally Contextualized Approach,” Journal of Clinical Epidemiology 150 (2022): 216–224. 35934265 10.1016/j.jclinepi.2022.07.014

[39] Q. Shi , K. Nong , P. O. Vandvik , et al., “Benefits and Harms of Drug Treatment for Type 2 Diabetes: Systematic Review and Network Meta‐Analysis of Randomised Controlled Trials,” BMJ 381 (2023): e074068. 37024129 10.1136/bmj-2022-074068 PMC10077111

[40] R. Brignardello‐Petersen , I. D. Florez , A. Izcovich , et al., “GRADE Approach to Drawing Conclusions From a Network Meta‐Analysis Using a Minimally Contextualised Framework,” BMJ 371 (2020): m3900. 33177059 10.1136/bmj.m3900

[41] M. Noordzij , M. van Diepen , F. C. Caskey , and K. J. Jager , “Relative Risk Versus Absolute Risk: One Cannot be Interpreted Without The Other,” Nephrology Dialysis Transplantation 32, no. suppl_2 (2017): ii13–ii18. 10.1093/ndt/gfw465 28339913

[42] G. Li , G. Y. H. Lip , M. Marcucci , L. Thabane , J. Tian , and M. A. H. Levine , “The Number Needed to Treat for Net Effect (NNTnet) as a Metric for Measuring Combined Benefits and Harms,” Journal of Clinical Epidemiology 125 (2020): 100–107. 32512185 10.1016/j.jclinepi.2020.05.031

[43] B. Zhang , W. Pei , P. Cai , Z. Wang , and F. Qi , “Recent Advances in Chinese Patent Medicines Entering the International Market,” Drug Discoveries & Therapeutics 16, no. 6 (2022): 258–272. 36543180 10.5582/ddt.2022.01115

[44] National Medical Products Administration , 2021 National Blue Book of Chinese Medicinal Herbs Regulation (Chinese Medicine Science and Technology Publishing House, 2021).

[45] L. Cao , H. Ni , X. Gong , Z. Zang , and H. Chang , “Chinese Herbal Medicines for Coronary Heart Disease: Clinical Evidence, Pharmacological Mechanisms, and the Interaction With Gut Microbiota,” Drugs 84, no. 2 (2024): 179–202. 38265546 10.1007/s40265-024-01994-w

[46] Y. Xie , F. Han , Y. Jin , et al., “Organic Integration of Traditional Chinese and Western Medicines‐Future of Clinical Practice Guidelines of Integrated Traditional Chinese and Western Medicines,” Chinese Journal of Integrative Medicine 30, no. 4 (2024): 359–365. 37528326 10.1007/s11655-023-3739-9

[47] K. G. Shojania , M. Sampson , M. T. Ansari , J. Ji , S. Doucette , and D. Moher , “How Quickly do Systematic Reviews go Out of Date? A Survival Analysis,” Annals of Internal Medicine 147, no. 4 (2007): 224–233, 10.7326/0003-4819-147-4-200708210-00179. 17638714

[48] M. D. Neuman , J. N. Goldstein , M. A. Cirullo , and J. S. Schwartz , “Durability of Class I American College of Cardiology/American Heart Association Clinical Practice Guideline Recommendations,” Journal of the American Medical Association 311, no. 20 (2014): 2092–2100, 10.1001/jama.2014.4949. 24867012 PMC4346183

[49] L. Martínez García , A. J. Sanabria , E. García Alvarez , et al., “The Validity of Recommendations From Clinical Guidelines: A Survival Analysis,” Canadian Medical Association Journal 186, no. 16 (2014): 1211–1219, 10.1503/cmaj.140547. 25200758 PMC4216254

[50] L. J. H. Alderson , P. Alderson , and T. Tan , “Median Life Span of a Cohort of National Institute for Health and Care Excellence Clinical Guidelines was About 60 Months,” Journal of Clinical Epidemiology 67, no. 1 (2014): 52–55, 10.1016/j.jclinepi.2013.07.012. 24139089

