# Supplementary material for: Blood Activating and Stasis Removing Chinese Patent Medicine in Perioperative Period of Percutaneous Coronary Intervention for Myocardial Infarction: A Protocol for a Living Clinical Practice Guideline
Source: Clin Public Health Guidel. 2026 Apr 1;3(2):e70068. doi: 10.1002/gin2.70068 (PMC13563398; doi:10.1002/gin2.70068)
Supplement: Supplementary file 1 [file GIN2-3-e70068-s002.pdf]

Chinese patent medicine：Tongxinluo

Classification of Outcome Importance	Classification of the Outcome Type	Outcome Measure	RR/MD and Confidence Interval	Number Needed to Treat and Confidence Interval	Certainty of Evidence（GRADE）	Evidence—Effect Classification
Critical Outcome	Effectiveness (Binary)					
Important Outcome	Effectiveness (Binary)					
	Effectiveness (continuous)					
	Safety (Binary)					

Bringing Guidelines to the digital Age

Evidence certainty is high or Moderate	Evidence certainty is low or very low
One of the most effective/safe drugs	One of the potentially most effective/safe drugs
One of the Moderately effective/safe drugs	Potentially one of the Moderately effective/safe drugs
No significant difference compared to standard treatment	Potentially no significant difference compared to standard treatment
