# Supplementary material for: Blood Activating and Stasis Removing Chinese Patent Medicine in Perioperative Period of Percutaneous Coronary Intervention for Myocardial Infarction: A Protocol for a Living Clinical Practice Guideline
Source: Clin Public Health Guidel. 2026 Apr 1;3(2):e70068. doi: 10.1002/gin2.70068 (PMC13563398; doi:10.1002/gin2.70068)
Supplement: Supplementary file 2 [file GIN2-3-e70068-s001.docx]

**Definition, Classification, and Management of Conflicts of Interest**

**(1) Direct COIs (No participation is allowed)**: Direct COIs involve financial relationships that may unduly influence judgment, including: **Personal income:** Employment, consultancy fees, honoraria, or gifts from entities affected by the guidelines. **Financial stakes:** Stock ownership, patents, or royalties related to interventions covered by the guideline. **Research funding:** Direct payments (>50000 RMB/year) or material support from industry sponsors. Individuals with direct COIs are strictly prohibited from participating in any part of the guideline development process. This includes serving as members of the guideline working group or engaging in any related activities.

**(2) Indirect COIs (Participation is restricted)**: Indirect COIs include non-financial interests or secondary financial ties that may introduce bias, such as: **Institutional funding:** Grants or donations to the participant’s affiliated institution. **Academic advocacy:** Leadership roles in professional societies or public endorsements of specific treatments. **Non-monetary benefits**: Familial ties to patients, personal beliefs (e.g., political/religious views), or career advancement linked to guideline outcomes. Members with indirect COIs should refrain from participating in evidence retrieval, synthesis, assessment, recommendation development, and voting, but can participate in discussions not related to the formulation of recommendations.
