# Supplementary material for: Blood Activating and Stasis Removing Chinese Patent Medicine in Perioperative Period of Percutaneous Coronary Intervention for Myocardial Infarction: A Protocol for a Living Clinical Practice Guideline
Source: Clin Public Health Guidel. 2026 Apr 1;3(2):e70068. doi: 10.1002/gin2.70068 (PMC13563398; doi:10.1002/gin2.70068)
Supplement: Supplementary file 3 [file GIN2-3-e70068-s003.docx]

Survey Questionnaire on the Application of Chinese Patent Medicine in Myocardial Infarction

Q1.Have you ever been involved in the clinical diagnosis and treatment of coronary heart disease?

YES☐

NO☐

Q2. How long have you been involved in the clinical diagnosis and treatment of coronary heart disease?

3YEARS☐

4-9YEARS☐

10 YEARS☐

Q3. What is the nature of the medical institution you work in?

Inclined to Traditional Chinese Medicine☐

Inclined to Western Medicine☐

Other☐

Q4. What is the level of your medical institution?

Tertiary Hospital☐

Secondary Hospital☐

Primary Hospital☐

Other☐

Q5. Which province in China is your medical institution located in?

Jiangxi Province☐

Henan Province☐

Guangdong Province☐

Shanxi Province☐

Liaoning Province☐

Shandong Province☐

Beijing Municipality☐

Hebei Province☐

Jiangsu Province☐

Zhejiang Province☐

Heilongjiang Province☐

Shaanxi Province☐

Sichuan Province☐

Hunan Province☐

Fujian Province☐

Tianjin Municipality☐

Jilin Province☐

Hubei Province☐

Yunnan Province☐

Anhui Province☐

Inner Mongolia Autonomous Region☐

Shanghai Municipality☐

Guangxi Zhuang Autonomous Region☐

Xinjiang Uygur Autonomous Region☐

Chongqing Municipality☐

Guizhou Province☐

Ningxia Hui Autonomous Region☐

Qinghai Province☐

Taiwan Province☐

Overseas☐

Q6.Have you used Chinese Patent Medicine in the treatment of myocardial infarction?

Yes☐

No☐

Q7. What are the reasons for not using Chinese Patent Medicine? (Multiple choices)

Never used Chinese Patent Medicine☐

Pharmacy does not have Chinese Patent Medicine for treating this type of disease☐

Uncertain about how to use or lack of medication guidance☐

Other☐

Q8. Why do you use Chinese Patent Medicine? (Multiple choices)

Have experience in application, and there is efficacy☐

Follow guidelines/clinical pathways☐

Hospital/department requirements☐

Patient or caregiver requirements☐

Other☐

Q9.Which of the following Chinese Patent Medicine for promoting blood circulation and removing blood stasis have you used in the treatment of myocardial infarction? (Multiple choices)

Dazhu Hongjingtian injection☐

Dazhu Hongjingtian capsule☐

Danhong injection☐

Danshen injection☐

Danshen Dizhuye☐

Danxiang Guanxin injection☐

Danlou tablet☐

Xinyuan capsule☐

Xinkeshu tablet☐

Xinnaoning capsule☐

XinNaoQingRuanJiaoNang☐

Xinnaoshutong capsule☐

Xinshu capsule☐

Kedaling tablet☐

Lemai granule☐

Xinxuekang soft capsule☐

Di'ao Xinxuekang capsule☐

xuefu zhuyu capsule☐

Xueshuan Xinmaining Tablet☐

Dengzhanshengmai capsule☐

Dengzhanxixin injection☐

dengzhanxixin granule☐

Qishen Yiqi dropping pill☐

Kudiezi injection☐

Salvianolate injection☐

breviscapine☐

Safflower yellow pigment for injection☐

Xiangdan injection☐

Compound Danshen tablet☐

compound Danshen dripping pill☐

Compound Xueshuantong Capsule☐

yangxinshi tablet☐

Guanxin Danshen dropping pill☐

guanxinshutong capsule☐

guanxinjing☐

Suxiao Jiuxin pill☐

Naoxintong Capsule☐

Naoxinqing tablet☐

Yixinshu capsule☐

NuoDiKangJiaoNang☐

Tongxinluo capsule☐

yindan xinnaotong☐

Ginkgo biloba capsule☐

Ginkgo leaf drop pill☐

Ginkgo Ketone Ester Dispersible tablet☐

Ginkgo Ketone Ester dropping pill☐

Shexiang Baoxin pill☐

Q10. In your opinion, what are the advantages of using Chinese Patent Medicine for promoting blood circulation and removing blood stasis in myocardial infarction?
