# Supplementary material for: Blood Activating and Stasis Removing Chinese Patent Medicine in Perioperative Period of Percutaneous Coronary Intervention for Myocardial Infarction: A Protocol for a Living Clinical Practice Guideline
Source: Clin Public Health Guidel. 2026 Apr 1;3(2):e70068. doi: 10.1002/gin2.70068 (PMC13563398; doi:10.1002/gin2.70068)
Supplement: Supplementary file 4 [file GIN2-3-e70068-s004.docx]

**The items of Questions**

| **Serial Number** | **Question** |
| --- | --- |
| 1 | In patients with myocardial infarction in the perioperative period of PCI, should blood-activating and stasis-resolving Chinese patent medicines be used in combination with standard treatment compared to standard treatment alone? |
| 2 | In patients with different types of myocardial infarction (STEMI vs. NSTEMI), should the choice of Chinese patent medicine treatment be adjusted for optimal efficacy? |
| 3 | For patients receiving blood-activating and stasis-resolving Chinese patent medicines, does the dosage form (pills, tablets, injections, or capsules) affect therapeutic outcomes? |
| 4 | Should the choice of blood-activating and stasis-resolving Chinese patent medicines be tailored based on Traditional Chinese Medicine syndrome differentiation (e.g., qi deficiency and blood stasis, phlegm stasis coagulation, etc.) for better clinical outcomes? |

**Definition, effect measure and follow-up time of outcomes**

| **Outcome** **at the group level** | **Classification of outcomes** | **Definition of outcome at each group** | **Effect measure** | **Follow-up time** |
| --- | --- | --- | --- | --- |
| Death | Critical outcome | Number of patients who died due to any reason within follow-ups. | Relative Risk | 1, 3, 6, and 12 months |
| Myocardial Reinfarction | Critical outcome | Number patients who have previously experienced a myocardial infarction (MI), a new episode of myocardial necrosis occurring after a certain period of time. | Relative Risk | 1, 3, 6, and 12 months |
| Heart failure | Critical outcome | Number of patients with heart failure. | Relative Risk | 1, 3, 6, and 12 months |
| Stroke | Critical outcome | Number of patients who suffered a stroke. | Relative Risk | 1, 3, 6, and 12 months |
| TIMI | Critical outcome | Number of patients achieving Thrombolysis in MI (TIMI) grade 3 flow. | Relative Risk | postoperative, 1, 3 months |
| LVEF | Important outcome | Mean percentage of blood ejected | Mean Difference | 7 days, 1, and 3 months |
| CTnI | Important outcome | Mean Concentration of Troponin I in the blood. | Mean Difference | 1, 3, and 7 days |
| CTnT | Important outcome | Mean Concentration of Troponin T in the blood. | Mean Difference | 1, 3, and 7 days |
| CKMB | Important outcome | Mean Concentration of CKMB in the blood. | Mean Difference | 1, 3, and 7 days |
| NTproBNP | Important outcome | Mean Concentration of NTproBNP in the blood. | Mean Difference | 7 days, 1, 3 months |
| Traditional Chinese Medicine Syndrome Score Scale | Important outcome | By observing each patient's symptoms, signs, tongue, and pulse, and based on Traditional Chinese Medicine theory, the syndrome of chest pain, chest tightness, palpitations, and shortness of breath is quantified and scored, resulting in the average score for each patient. | Mean Difference | 7 days, 1, 3 months |
| Angin | Important outcome | Number of patients with chest pain or discomfort. | Relative Risk | 1, 3, 6, and 12 months |
| Major bleeding | Important outcome | Number of patients with intracranial hemorrhage, spinal cord hemorrhage, vision loss due to ocular bleeding, or a decrease in hemoglobin concentration of ≥ 5 g/dL. | Relative Risk | 1, 3, 6, and 12 months |
| Minor bleeding | Important outcome | Number of people with bleeding other than major bleeding. | Relative Risk | 1, 3, 6, and 12 months |
| Adverse effects | Important outcome | Number of people with liver dysfunction, kidney dysfunction, dizziness, vertigo, rash, headache, gastrointestinal reactions, shortness of breath, hypoglycemia, and anaphylactic shock. | Relative Risk | 1, 3, 6, and 12 months |
