# Supplementary material for: Blood Activating and Stasis Removing Chinese Patent Medicine in Perioperative Period of Percutaneous Coronary Intervention for Myocardial Infarction: A Protocol for a Living Clinical Practice Guideline
Source: Clin Public Health Guidel. 2026 Apr 1;3(2):e70068. doi: 10.1002/gin2.70068 (PMC13563398; doi:10.1002/gin2.70068)
Supplement: Supplementary file 5 [file GIN2-3-e70068-s005.docx]

Evidence to Decision Table

| Chinese patent medicine | |  | | |
| --- | --- | --- | --- | --- |
| Population (P) | |  | | |
| Intervention (I) | |  | | |
| Control (C) | |  | | |
| Outcome (O) | |  | | |
| Application Environment | |  | | |
| Perspective | |  | | |
| Subgroup Population | |  | | |
| Clinical Scenario | |  | | |
| Conflict of Interest | | The guideline development group has no conflicts of interest. | | |
| Assessment | | | | |
| Judgment | | Evidence | | |
| 1. How substantial are the desirable anticipated effects? | | | | |
| □Trivial  □Small  □Moderate  □Large  □Varies  □Don't know | |  | | |
| 1. How substantial are the undesirable anticipated effects? | | | | |
| □Trivial  □Small  □Moderate  □Large  □Varies  □Don't know | |  | | |
| 1. What is the overall quality certainty of the evidence for the effect? | | | | |
| □Very low  □Low  □Moderate  □High  □No included studies | |  | | |
| 1. Does the balance between desirable and undesirable effects favour the option or the comparison? | | | | |
| □Favors the comparison  □Probably favors the comparison  □Does not favor either the option or the comparison  □Probably favors the option  □Favors the option  □Varies  □Don't know | |  | | |
| 1. Is the option feasible to implement? | | | | |
| □No  □Possibly not  □Possibly  □Yes  □Varies  □Don't know | |  | | |
| 1. How large are the resource requirements (costs)? | | | | |
| □Large savings  □Moderate savings  □Negligible costs or savings  □Moderate costs  □Large costs  □Varies  □Don't know | |  | | |
| Conclusion | | | | |
| Strong Recommendation□ | Weak Recommendation□ | Unable to make a recommendation □ | Strong Recommendation Against□ | Weak Recommendation Against□ |
