# Supplementary material for: Blood Activating and Stasis Removing Chinese Patent Medicine in Perioperative Period of Percutaneous Coronary Intervention for Myocardial Infarction: A Protocol for a Living Clinical Practice Guideline
Source: Clin Public Health Guidel. 2026 Apr 1;3(2):e70068. doi: 10.1002/gin2.70068 (PMC13563398; doi:10.1002/gin2.70068)
Supplement: Supplementary file 6 [file GIN2-3-e70068-s006.docx]

Living Clinical Practice Guidelines on the Perioperative Period of PCI for Myocardial Infarction Treated with Blood Activating and Stasis Removing Chinese Patent Medicine: Conflict of Interest Disclosure Form

Table 1: Basic Information Form for Experts

| Name |  | | Sex |  | Unit | |  | | |
| --- | --- | --- | --- | --- | --- | --- | --- | --- | --- |
| Rank |  | | Duty |  | | | Professional field | |  |
| Phone number | |  | | | | E-mail | |  | |

●All experts on the guideline steering committee, the guideline consensus group, the guideline development working group and the guideline external review group must disclose all potential conflicts of interest (e.g. all interests that affect or may affect the objectivity and independence of the experts).

●On this conflict of interest statement, you must disclose any commercial, professional or other conflicts of interest related to the subject of this guideline, and any interests that may be affected by the results of this guideline.

**Please copy the "√" next to the "yes" or "no" option. If you choose "yes", please copy "√" after the name of the relevant enterprise or company you choose, such as Shanghai GeneoDx Biotech Co., Ltd√. If the enterprise or company related to you is not listed, please add it to the horizontal line after ‘other enterprise or companies’.**

Table 2: Declaration of Economic Interests Conflict

| I have held stocks in companies that have a vested interest in this guideline over the past three years, or received royalties or license fees. | |
| --- | --- |
| Dazhu Hongjingtian injection( produced by Tonghua Yusheng Pharmaceutical Co., Ltd./Other company or organization __________) | YES□ NO□ |
| Dazhu Hongjingtian capsul(Produced by Jiangsu Kangyuan Pharmaceutical Co., Ltd./Other Company or Organization___________) | YES□ NO□ |
| Danhong Injection (Produced by Shandong Danhong Pharmaceutical Co., Ltd./Other Company or Organization___________) | YES□ NO□ |
| Danshen injection(CHIATAI QINGCHUNBAO Pharmaceutical Co., Ltd./DaLi Pharmaceutical Co., Ltd./ Hubei Tiansheng Pharmaceutical Co., Ltd. / Ma'anshan Fengyuan Pharmaceutical Co., Ltd. / Changshu Lei Yun Shang Pharmaceutical Co., Ltd. / Guangdong Lei Yun Shang Pharmaceutical Co., Ltd. / Fujian Gutian Pharmaceutical Co., Ltd./Jilin Huinan Changlong Biochemical Pharmaceutical Co., Ltd./Other Enterprises or Companies ___________） | YES□ NO□ |
| Danshen dizhuye（Shanghai Huayuan Anhui Jinhui Pharmaceutical Co., Ltd/ Anhui Tianyang Pharmaceutical Co., Ltd./Other enterprises or companies ___________） | YES□ NO□ |
| Danxiang Guanxin injection（Shanghai Pharmaceutical (Group) Co., Ltd., Jiangsu Hengfeng Pharmaceutical Co., Ltd., Shanghai Pharmaceuticals Holding Co., Ltd., Wanbangde Pharmaceutical Group Co., Ltd., Other enterprises or companies ___________） | YES□ NO□ |
| Danlou tablet（Jilin Connell Pharmaceutical Co., Ltd./Other enterprises or companies ___________） | YES□ NO□ |
| Xinyuan capsules（AUSPICIOUSNESS Peace(sichuan) Pharmacy co.Ltd, Other enterprises or companies ___________） | YES□ NO□ |
| Xinkeshu tablet（Shandong Wohua Pharmaceutical Technology Co., Ltd. , Other enterprises or companies ___________） | YES□ NO□ |
| Xinnaoning capsule（Guizhou Jingcheng Pharmaceutical Co., Ltd./Other enterprises or companies ___________） | YES□ NO□ |
| XinNaoQingRuanJiaoNang（Shanxi Liye Pharmaceutical Co., Ltd./Shineway Pharmaceutical Group Co., Ltd./Guangzhou Baiyunshan Xinqun (Pharmaceutical) Co., Ltd./Jilin Hengxing Technology Pharmaceutical Co., Ltd./Henan Shengmingniao Pharmaceutical Co., Ltd.Sionpharm Pharmaceutical Group Corporation Zhonglian Pharmaceutical Co., Ltd./Other enterprises or companies ___________） | YES□ NO□ |
| Xinnaoshutong capsule（Jilin Aodong Taonan Pharmaceutical Co., Ltd./Other enterprises or companies ___________） | YES□ NO□ |
| Xinshu capsule（Guizhou Xinbang Pharmaceutical Co., Ltd./Jilin Tianyao Bencao Tang Pharmaceutical Co., Ltd./Shandong Zhongjiankangqiao Pharmaceutical Co., Ltd./Guizhou Bait Pharmaceutical Co., Ltd./Other enterprises or companies ___________） | YES□ NO□ |
| Kedaling tablets（Zhejiang Conba Pharmaceutical Co., Ltd./Other enterprises or companies ___________） | YES□ NO□ |
| Lemai granule（Sichuan Chuan Da Huaxi Pharmaceutical Co., Ltd. /Other enterprises or companies ___________） | YES□ NO□ |
| Di'ao Xinxuekang（Chengdu Di'ao Pharmaceutical Group Co., Ltd. /Other enterprises or companies ___________） | YES□ NO□ |
| xuefu zhuyu capsule（Tianjin Hongrentang Pharmaceutical Co., Ltd. /Other enterprises or companies ___________） | YES□ NO□ |
| Xueshuan Xinmaining Tablet（Jilin Huakang Pharmaceutical Co., Ltd./Other enterprises or companies ___________） | YES□ NO□ |
| Dengzhanshengmai capsule（Yunnan Biogu Pharmaceutical Co., Ltd./Other enterprises or companies ___________） | YES□ NO□ |
| Dengzhanxixin injection（Yunnan Biogu Pharmaceutical Co., Ltd./Other enterprises or companies ___________） | YES□ NO□ |
| dengzhanxixin granule（Yunnan Botanical Pharmaceutical Co., Ltd./Other enterprises or companies ___________） | YES□ NO□ |
| Qishen Yiqi dropping pill（Tasly Holding Group Co., Ltd./Other enterprises or companies ___________） | YES□ NO□ |
| Kudiezi injection（Tonghua Huaxia Pharmaceutical Co., Ltd./Shenyang Shuangding Pharmaceutical Co., Ltd./Other enterprises or companies ___________） | YES□ NO□ |
| Salvianolate injection（Shanghai Lvgu Pharmaceutical Co., Ltd./Other enterprises or companies ___________） | YES□ NO□ |
| breviscapine（Hunan Hengsheng Pharmaceutical Co., Ltd./Kunming Longjin Pharmaceutical Co., Ltd./Other enterprises or companies ___________） | YES□ NO□ |
| Safflower yellow pigment for injection（Shanxi Deyuan Tang Pharmaceutical Co., Ltd./Zhejiang Yongning Pharmaceutical Co., Ltd./Other enterprises or companies ___________） | YES□ NO□ |
| Xiangdan injection（Kunming Yusi Pharmaceutical Co., Ltd./Sinopharm Sanjiu (Ya'an) Pharmaceutical Co., Ltd./Shanxi Pude Pharmaceutical Co., Ltd./Jilin Feino Pharmaceutical Co., Ltd./Sichuan Chuan Da Huaxi Pharmaceutical Co., Ltd./Other enterprises or companies ___________） | YES□ NO□ |
| Compound Danshen tablet（Techpool Pharmaceutical Group Co., Ltd./Huangshan Tianmu Pharmaceutical Co., Ltd./Beijing Shunxin Xiangyun Pharmaceutical Co., Ltd./Taiji Group Zhejiang Dongfang Pharmaceutical Co., Ltd./Other enterprises or companies ___________）（650） | YES□ NO□ |
| compound Danshen dripping pill（Tasly Pharmaceutical Group Co., Ltd./Other enterprises or companies ___________） | YES□ NO□ |
| Compound Xueshuantong Capsule（Guangdong Zhongsheng Pharmaceutical Co., Ltd./Other enterprises or companies ___________） | YES□ NO□ |
| yangxinshi tablet（Shanghai Pharmaceuticals Group Qingdao Guofeng Pharmaceutical Co., Ltd./Other enterprises or companies ___________） | YES□ NO□ |
| Guanxin Danshen dropping pill（Harbin Yerui Pharmaceutical Co., Ltd./Other enterprises or companies ___________） | YES□ NO□ |
| guanxinshutong capsule（Shaanxi Buchang Pharmaceutical Co., Ltd./Other enterprises or companies ___________） | YES□ NO□ |
| guanxinjing（Baoding Traditional Chinese Medicine Pharmaceutical Co., Ltd./Other enterprises or companies ___________） | YES□ NO□ |
| Suxiao Jiuxin pill（Tianjin Yaoda Rentang Group Co., Ltd. Sixth Traditional Chinese Medicine Factory/Other enterprises or companies ___________） | YES□ NO□ |
| Naoxintong Capsule（Shaanxi Buchang Pharmaceutical Co., Ltd./Other enterprises or companies ___________） | YES□ NO□ |
| Naoxinqing tablet（Guangzhou Baiyunshan Heji Huangpu Traditional Chinese Medicine Co., Ltd./Other enterprises or companies ___________） | YES□ NO□ |
| Yixinshu capsule（Guizhou Xinbang Pharmaceutical Co., Ltd./Other enterprises or companies ___________） | YES□ NO□ |
| NuoDiKangJiaoNang（Sichuan Nuodikangwei Guang Pharmaceutical Co., Ltd./Other enterprises or companies ___________） | YES□ NO□ |
| Tongxinluo capsule（Shijiazhuang Yiling Pharmaceutical Co., Ltd./Other enterprises or companies ___________） | YES□ NO□ |
| yindan xinnaotong（Guizhou Bai Ling Enterprise Group Pharmaceutical Co., Ltd./Other enterprises or companies ___________） | YES□ NO□ |
| Ginkgo biloba capsule（Hunan Hansoh Pharmaceutical Co., Ltd./Guangxi Shengte Pharmaceutical Co., Ltd.; Guilin Honghui Pharmaceutical Co., Ltd./Shandong Zhongjiankangqiao Pharmaceutical Co., Ltd./Hangzhou Kangenbei Pharmaceutical Co., Ltd./Wuhu Luye Pharmaceutical Co., Ltd./Hunan Huabaotong Pharmaceutical Co., Ltd./Changzhou Lanling Pharmaceutical Co., Ltd./Shanghai Xinyi Bairuida Pharmaceutical Co., Ltd./Yunnan Kangenbei Xitao Pharmaceutical Co., Ltd./Other enterprises or companies ___________） | YES□ NO□ |
| Ginkgo leaf drop pill（Wanbangde Pharmaceutical Group Co., Ltd./Other enterprises or companies ___________） | YES□ NO□ |
| Ginkgo Ketone Ester Dispersible tablet（Jiangsu Shenlong Pharmaceutical Co., Ltd./Other enterprises or companies ___________） | YES□ NO□ |
| Ginkgo Ketone Ester dropping pill（Zhejiang Shangyao Jiuxu Pharmaceutical Co., Ltd./Shanxi Qianhui Pharmaceutical Co., Ltd./Beijing Handian Pharmaceutical Co., Ltd./Other enterprises or companies ___________） | YES□ NO□ |
| Shexiang Baoxin pill（Shanghai Hutchison Pharmaceuticals Co., Ltd./Other enterprises or companies ___________） | YES□ NO□ |
| Shexiang Tongxin dripping pill（Inner Mongolia Kanghongbei Pharmaceutical Co., Ltd. Shenglong Branch/Other enterprises or companies ___________） | YES□ NO□ |
| In the past 24 months, have you received any consulting fees, lecture honoraria, or gifts from enterprises/institutions related to this guideline? | YES□ NO□ |
| I have published academic papers related to the treatment of myocardial infarction with traditional Chinese medicine in the past 2-3 months | YES□ NO□ |
| In the past 24 months, has your institution received any funding from enterprises related to the guideline topic? | YES□ NO□ |
| Have you been involved in any advocacy activities related to the 47 traditional Chinese medicine products listed above in the past 24 months? | YES□ NO□ |
| If your answer is “yes,” please list the relevant details and associated amounts. | |

Do you have any potential conflicts of interest that might affect the objectivity and impartiality of the guideline content (e.g., personal academic positions, religious beliefs, political views, or career advancement considerations)?

If you are currently participating in or hosting a treatment of myocardial infarction with traditional Chinese medicine, please fill in the project name in the box below.

In addition to the above, within the past 24 months, is there anything else that you need to declare, please fill in the box below.

In addition to the above, within the past 24 months, if your immediate family members (spouse and children) have conflicts of interest related to the above statement, please fill in the box below.

**Informed about the conflict of interest statement**: I agree to make the above completed content public to other guideline development members, and agree that the statement of interest form will be published in the guideline.

**Statement**: I promise that what I have stated is true and complete. If the information I have stated above changes at any time, I will promptly inform the guideline secretary group and complete a new statement of interest form.

**Signed: Date:**
