# Supplementary material for: Blood Activating and Stasis Removing Chinese Patent Medicine in Perioperative Period of Percutaneous Coronary Intervention for Myocardial Infarction: A Protocol for a Living Clinical Practice Guideline
Source: Clin Public Health Guidel. 2026 Apr 1;3(2):e70068. doi: 10.1002/gin2.70068 (PMC13563398; doi:10.1002/gin2.70068)
Supplement: Supplementary file 7 [file GIN2-3-e70068-s007.docx]

**Composition and Responsibilities of Guideline Working Groups**

**Guideline Working Groups**

**1 Guideline Steering Committee**

The Guideline Steering Committee (GSC) consists of five experts in the field of myocardial infarction from both TCM and WM. It oversees the entire guideline development process, including: approving the guideline plan, defining objectives, assembling relevant working groups, supervising the guideline development process, approving recommendations, reviewing and managing conflict of interest, promoting guideline publication, and facilitating subsequent updates. Additionally, collaborates with the Guideline Development Group in framing questions, selecting and rating outcomes.

**2 Guideline Development Group**

The Guideline Development Group (GDG) consists of experts from multiple disciplines, including 3 TCM cardiologists, 2 WM cardiologists, 3 evidence-based medicine experts, 2 medical statisticians. The GDG is responsible for selecting Chinese patent medicines, framing questions, selecting and rating outcomes (reviewed and approved by the GSC), developing the methodological framework, retrieving and evaluating evidence, conducting systematic reviews and network meta-analyses, and the drafting and revision of the initial guideline.

**3 Guideline Consensus Group**

The Guideline Consensus Group (GCG) consists of TCM and WM cardiovascular experts from multiple provinces in China. Its role is to vote on and reach a consensus on guideline recommendations. The selection of consensus group members is based on their expertise in TCM or WM and their extensive clinical experience in managing myocardial infarction. The selection process is supervised by the GSC and follows a rigorous candidate evaluation procedure, including expert nomination, background review, and experience verification, to ensure that members possess the necessary knowledge and experience while minimizing potential conflicts of interest.

**4 Guideline Secretariat**

Composed of 15 staff members with backgrounds in TCM internal medicine and evidence-based medicine research, they are primarily responsible for registering the guideline, recording consensus meetings, and documenting the entire process.

**5 Guideline External Review Group**

Composed of TCM internal medicine clinicians, WM cardiology clinicians, and methodological experts who do not directly participate in the guideline development, this group is responsible for assessing the accuracy and applicability of recommendations, providing feedback, and suggesting revisions. Members are selected from hospitals, academic institutions, and professional societies.
