# Supplementary material for: Blood Activating and Stasis Removing Chinese Patent Medicine in Perioperative Period of Percutaneous Coronary Intervention for Myocardial Infarction: A Protocol for a Living Clinical Practice Guideline
Source: Clin Public Health Guidel. 2026 Apr 1;3(2):e70068. doi: 10.1002/gin2.70068 (PMC13563398; doi:10.1002/gin2.70068)
Supplement: Supplementary file 8 [file GIN2-3-e70068-s008.docx]

1. Dazhu Hongjingtian Injection

CNKI

(TKA=‘大株红景天注射液’ + ‘大株红景天’) and ((TKA=‘心肌梗死’ + ‘心梗’ + ‘心肌梗塞’ + ‘急性冠脉综合征’+ ‘ACS’ + ‘STEMI’ + ‘NSTEMI’ + ‘胸痹’ + ‘胸痹心痛’ + ‘真心痛’ + ‘卒心痛’ + ‘心痛’ + ‘猝心痛’ + ‘厥心痛’) and (TKA=‘PCI’ + ‘PTCA’ + ‘经皮冠状动脉介入治疗术’ + ‘经皮冠状动脉介入’ + ‘经皮冠状动脉’ + ‘冠脉介入’ + ‘经皮冠脉’ + ‘血管成形术’ + ‘支架置入术’ + ‘冠状动脉成形术’ + ‘球囊’ + ‘支架’))

WangFang Data

主题:(“大株红景天注射液” OR “大株红景天”) and ((主题:心肌梗死 OR 心梗 OR 心肌梗塞 OR 急性冠脉综合征 OR ACS OR STEMI OR NSTEMI OR 胸痹 OR 胸痹心痛 OR 真心痛 OR 卒心痛 OR 心痛 OR 猝心痛 OR 厥心痛) and 主题:(PCI OR PTCA OR 经皮冠状动脉介入治疗术 OR 经皮冠状动脉介入 OR 经皮冠状动脉 OR 冠脉介入 OR 经皮冠脉 OR 血管成形术 OR 支架置入术 OR 冠状动脉成形术 OR 球囊 OR 支架))

VIP

(M=(“大株红景天注射液” OR “大株红景天”) OR R=(“大株红景天注射液” OR “大株红景天”)) AND ((M=(心肌梗死 OR 心梗 OR 心肌梗塞 OR 急性冠脉综合征 OR ACS OR STEMI OR NSTEMI OR 胸痹 OR 胸痹心痛 OR 真心痛 OR 卒心痛 OR 心痛 OR 猝心痛 OR 厥心痛) OR R=(心肌梗死 OR 心梗 OR 心肌梗塞 OR ACS OR STEMI OR NSTEMI OR 胸痹 OR 胸痹心痛 OR 真心痛 OR 卒心痛 OR 心痛 OR 猝心痛 OR 厥心痛)) AND (M=(PCI OR PTCA OR 经皮冠状动脉介入治疗术 OR 经皮冠状动脉介入 OR 经皮冠状动脉 OR 冠脉介入 OR 经皮冠脉 OR 血管成形术 OR 支架置入术 OR 冠状动脉成形术 OR 球囊 OR 支架) OR R=(PCI OR PTCA OR 经皮冠状动脉介入治疗术 OR 经皮冠状动脉介入 OR 经皮冠状动脉 OR 冠脉介入 OR 经皮冠脉 OR 血管成形术 OR 支架置入术 OR 冠状动脉成形术 OR 球囊 OR 支架)))

Web of Science

#1 TS=(Dazhu Hongjingtian injection OR Dazhu Hongjingtian OR Herba Rhodiolae dazhuhongjingtian)

#2 TS=(myocardial infarct* OR heart infarct* OR cardiac infarct* OR cardiovascular stroke* OR cardiogenic shock OR heart attack* OR ACS OR STEMI OR NSTEMI OR ST-segment elevation myocardial infarction OR Non-ST-segment elevation myocardial infarction OR MINOCA OR Myocardial infarction with no obstructive coronary atherosclerosis OR xiongbi OR xiongbixintong OR xiongbi Xintong OR zhenxintong OR cuxintong OR Xintong OR juexintong OR cardiovascular shock OR cardiogenic stroke*)

#3 TS=(percutaneous coronary intervention OR percutaneous coronary intervention* OR percutaneous coronary revascularization* OR coronary atherectom* OR transluminal coronary angioplast* OR transluminal coronary balloon dilation OR coronary balloon angioplast* OR PCI OR Percutaneous Transluminal Coronary Angioplast* OR PTCA OR Rotational Atherectom* OR Directional Coronary Atherectom* OR Balloon dilation OR Artery Stenting)

#4 #2 AND #3

#5 #1 AND #4

PubMed

#1 “Dazhu Hongjingtian injection”[Title/Abstract] OR “Dazhu Hongjingtian”[Title/Abstract] OR “Herba Rhodiolae”[Title/Abstract] OR “ dazhuhongjingtian”[Title/Abstract]

#2 "myocardial infarction"[MeSH Terms] OR “Acute Coronary Syndrome”[MeSH Terms] OR "myocardial infarct*"[Title/Abstract] OR “Acute Coronary Syndrome”[Title/Abstract] OR “ACS”[Title/Abstract] OR “Acute Coronary Syndrome*”[Title/Abstract]OR “NSTE-ACS”[Title/Abstract] OR “Non–ST-Segment Elevation Acute Coronary Syndrome*”[Title/Abstract] OR "heart infarct*"[Title/Abstract] OR "cardiac infarct*"[Title/Abstract] OR "cardiovascular stroke*"[Title/Abstract] OR "cardiogenic shock"[Title/Abstract] OR “cardiovascular shock” [Title/Abstract] OR “cardiogenic stroke*” [Title/Abstract] OR "heart attack*"[Title/Abstract] OR "STEMI"[Title/Abstract] OR "NSTEMI"[Title/Abstract] OR “ST-segment elevation myocardial infarction”[Title/Abstract] OR “Non-ST-segment elevation myocardial infarction”[Title/Abstract] OR “MINOCA”[Title/Abstract] OR “Myocardial infarction with no obstructive coronary atherosclerosis” [Title/Abstract] OR “xiongbi” [Title/Abstract] OR “xiongbixintong” [Title/Abstract] OR “xiongbi xintong” [Title/Abstract] OR “zhenxintong” [Title/Abstract] OR “cuxintong” [Title/Abstract] OR “Xintong” [Title/Abstract] OR “juexintong” [Title/Abstract]

#3 “percutaneous coronary intervention” [MeSH Terms] OR “percutaneous coronary intervention*”[Title/Abstract] OR “percutaneous coronary revascularization*” [Title/Abstract] OR “coronary atherectom*”[Title/Abstract] OR “transluminal coronary angioplast*”[Title/Abstract] OR “transluminal coronary balloon dilation”[Title/Abstract] OR “coronary balloon angioplast*”[Title/Abstract] OR “PCI”[Title/Abstract] OR “Percutaneous Transluminal Coronary Angioplast*”[Title/Abstract] OR “PTCA”[Title/Abstract] OR “Rotational Atherectom*”[Title/Abstract] OR

“Directional Coronary Atherectom*”[Title/Abstract] OR “Balloon dilation”[Title/Abstract] OR “Artery Stenting”[Title/Abstract]

#4 #2 AND #3

#5 #1 AND #4

Embase

#1 ‘Dazhu Hongjingtian injection':ab,ti OR ‘Dazhu Hongjingtian’:ab,ti OR ‘Herba Rhodiolae':ab,ti OR ‘dazhuhongjingtian’:ab,ti

#2 'heart infarction'/exp

#3 'Acute Coronary Syndrome'/exp

#4 'cardiac infarct':ab,ti OR 'cardiac infarction':ab,ti OR 'cardial infarct':ab,ti OR 'heart attack':ab,ti OR 'heart infarct':ab,ti OR 'heart micro infarction':ab,ti OR 'heart muscle infarction':ab,ti OR 'infarction, heart':ab,ti OR 'myocardial infarct':ab,ti OR 'myocardial infarction':ab,ti OR 'myocardium infarct':ab,ti OR 'myocardium infarction':ab,ti OR 'premonitory infarction sign':ab,ti OR 'second heart attack':ab,ti OR 'subendocardial infarction':ab,ti OR 'transmural cardiac infarction':ab,ti OR 'transmural heart infarction':ab,ti OR 'transmural infarction, heart':ab,ti OR 'heart infarction':ab,ti OR ‘Acute Coronary Syndrome’:ab,ti OR ‘ACS’:ab,ti OR ‘Acute Coronary Syndrome*’:ab,ti OR ‘NSTE-ACS’:ab,ti OR ‘Non–ST-Segment Elevation Acute Coronary Syndrome*’:ab,ti OR 'STEMI':ab,ti OR 'NSTEMI':ab,ti OR ‘ST-segment elevation myocardial infarction’:ab,ti OR ‘Non-ST-segment elevation myocardial infarction’:ab,ti OR ‘MINOCA’:ab,ti OR ‘Myocardial infarction with no obstructive coronary atherosclerosis’:ab,ti OR ‘xiongbi’:ab,ti OR ‘xiongbixintong’:ab,ti OR ‘xiongbi Xintong’:ab,ti OR ‘zhenxintong’:ab,ti OR ‘cuxintong’:ab,ti OR ‘Xintong’:ab,ti OR ‘juexintong’:ab,ti OR ‘cardiovascular shock’:ab,ti OR ‘cardiogenic stroke*’:ab,ti

#5 ‘percutaneous coronary intervention'/exp

#6 ‘percutaneous coronary intervention*':ab,ti OR 'percutaneous coronary revascularization*':ab,ti OR 'coronary atherectom*':ab,ti OR 'transluminal coronary angioplast*':ab,ti OR 'transluminal coronary balloon dilation':ab,ti OR 'coronary balloon angioplast*':ab,ti OR 'PCI':ab,ti OR 'Percutaneous Transluminal Coronary Angioplast*':ab,ti OR 'PTCA':ab,ti OR 'Rotational Atherectom*':ab,ti OR 'Directional Coronary Atherectom*':ab,ti OR 'Balloon dilation':ab,ti OR 'Artery Stenting’:ab,ti

#7 #2 OR #3

#8 #4 OR #7

#9 #5 OR #6

#10 #8 AND #9

#11 #1 AND #10

Cochrane

#1 (Dazhu Hongjingtian injection):ti,ab,kw OR (Dazhu Hongjingtian):ti,ab,kw OR (Herba Rhodiolae):ti,ab,kw OR (dazhuhongjingtian):ti,ab,kw

#2 MeSH descriptor: [Myocardial Infarction] explode all trees

#3 MeSH descriptor: [Acute Coronary Syndrome] explode all trees

#4 (myocardial infarct*):ti,ab,kw OR (heart infarct*):ti,ab,kw OR (cardiac infarct*):ti,ab,kw OR (cardiovascular stroke*):ti,ab,kw OR (cardiogenic shock):ti,ab,kw OR (heart attack*):ti,ab,kw OR (Acute Coronary Syndrome):ti,ab,kw OR (ACS):ti,ab,kw OR (Acute Coronary Syndrome*):ti,ab,kw OR (NSTE-ACS):ti,ab,kw OR (Non–ST-Segment Elevation Acute Coronary Syndrome*):ti,ab,kw OR (STEMI):ti,ab,kw OR (NSTEMI):ti,ab,kw OR (ST-segment elevation myocardial infarction):ti,ab,kw OR (Non-ST-segment elevation myocardial infarction):ti,ab,kw OR (MINOCA):ti,ab,kw OR (Myocardial infarction with no obstructive coronary atherosclerosis):ti,ab,kw OR (xiongbi):ti,ab,kw OR (xiongbixintong):ti,ab,kw OR (xiongbi Xintong):ti,ab,kw OR (zhenxintong):ti,ab,kw OR (cuxintong):ti,ab,kw OR (Xintong):ti,ab,kw OR (juexintong):ti,ab,kw OR (cardiovascular shock):ti,ab,kw OR (cardiogenic stroke*):ti,ab,kw

#5 MeSH descriptor: [Percutaneous Coronary Intervention] explode all trees

#6 (percutaneous coronary intervention):ti,ab,kw OR (percutaneous coronary intervention*):ti,ab,kw OR (percutaneous coronary revascularization*):ti,ab,kw OR (coronary atherectom*):ti,ab,kw OR (transluminal coronary angioplast*):ti,ab,kw OR (transluminal coronary balloon dilation):ti,ab,kw OR (coronary balloon angioplast*):ti,ab,kw OR (PCI):ti,ab,kw OR (Percutaneous Transluminal Coronary Angioplast*):ti,ab,kw OR (PTCA):ti,ab,kw OR (Rotational Atherectom*):ti,ab,kw OR (Directional Coronary Atherectom*):ti,ab,kw OR (Balloon dilation):ti,ab,kw OR (Artery Stenting):ti,ab,kw

#7 #2 OR #3

#8 #4 OR #7

#9 #5 OR #6

#10 #8 AND #9

#11 #1 AND #10

2.Dazhu Hongjingtian capsules

CNKI

(TKA=‘大株红景天胶囊’ + ‘大株红景天’) and ((TKA=‘心肌梗死’ + ‘心梗’ + ‘心肌梗塞’ + ‘急性冠脉综合征’+ ‘ACS’ + ‘STEMI’ + ‘NSTEMI’ + ‘胸痹’ + ‘胸痹心痛’ + ‘真心痛’ + ‘卒心痛’ + ‘心痛’ + ‘猝心痛’ + ‘厥心痛’) and (TKA=‘PCI’ + ‘PTCA’ + ‘经皮冠状动脉介入治疗术’ + ‘经皮冠状动脉介入’ + ‘经皮冠状动脉’ + ‘冠脉介入’ + ‘经皮冠脉’ + ‘血管成形术’ + ‘支架置入术’ + ‘冠状动脉成形术’ + ‘球囊’ + ‘支架’))

WangFang Data

主题:(“大株红景天胶囊” OR “大株红景天”) and ((主题:心肌梗死 OR 心梗 OR 心肌梗塞 OR 急性冠脉综合征 OR ACS OR STEMI OR NSTEMI OR 胸痹 OR 胸痹心痛 OR 真心痛 OR 卒心痛 OR 心痛 OR 猝心痛 OR 厥心痛) and 主题:(PCI OR PTCA OR 经皮冠状动脉介入治疗术 OR 经皮冠状动脉介入 OR 经皮冠状动脉 OR 冠脉介入 OR 经皮冠脉 OR 血管成形术 OR 支架置入术 OR 冠状动脉成形术 OR 球囊 OR 支架))

VIP

(M=(“大株红景天胶囊” OR “大株红景天”) OR R=(“大株红景天胶囊” OR “大株红景天”)) AND ((M=(心肌梗死 OR 心梗 OR 心肌梗塞 OR 急性冠脉综合征 OR ACS OR STEMI OR NSTEMI OR 胸痹 OR 胸痹心痛 OR 真心痛 OR 卒心痛 OR 心痛 OR 猝心痛 OR 厥心痛) OR R=(心肌梗死 OR 心梗 OR 心肌梗塞 OR ACS OR STEMI OR NSTEMI OR 胸痹 OR 胸痹心痛 OR 真心痛 OR 卒心痛 OR 心痛 OR 猝心痛 OR 厥心痛)) AND (M=(PCI OR PTCA OR 经皮冠状动脉介入治疗术 OR 经皮冠状动脉介入 OR 经皮冠状动脉 OR 冠脉介入 OR 经皮冠脉 OR 血管成形术 OR 支架置入术 OR 冠状动脉成形术 OR 球囊 OR 支架) OR R=(PCI OR PTCA OR 经皮冠状动脉介入治疗术 OR 经皮冠状动脉介入 OR 经皮冠状动脉 OR 冠脉介入 OR 经皮冠脉 OR 血管成形术 OR 支架置入术 OR 冠状动脉成形术 OR 球囊 OR 支架)))

Web of Science

#1 TS=(Dazhu Hongjingtian capsule* OR Herba Rhodiolae OR Dazhu Hongjingtian OR dazhuhongjingtian)

#2 TS=(myocardial infarct* OR heart infarct* OR cardiac infarct* OR cardiovascular stroke* OR cardiogenic shock OR heart attack* OR ACS OR STEMI OR NSTEMI OR ST-segment elevation myocardial infarction OR Non-ST-segment elevation myocardial infarction OR MINOCA OR Myocardial infarction with no obstructive coronary atherosclerosis OR xiongbi OR xiongbixintong OR xiongbi Xintong OR zhenxintong OR cuxintong OR Xintong OR juexintong OR cardiovascular shock OR cardiogenic stroke*)

#3 TS=(percutaneous coronary intervention OR percutaneous coronary intervention* OR percutaneous coronary revascularization* OR coronary atherectom* OR transluminal coronary angioplast* OR transluminal coronary balloon dilation OR coronary balloon angioplast* OR PCI OR Percutaneous Transluminal Coronary Angioplast* OR PTCA OR Rotational Atherectom* OR Directional Coronary Atherectom* OR Balloon dilation OR Artery Stenting)

#4 #2 AND #3

#5 #1 AND #4

PubMed

#1 “Dazhu Hongjingtian capsule*”[Title/Abstract] OR “Herba Rhodiolae”[Title/Abstract] OR “Dazhu Hongjingtian”[Title/Abstract] OR “ dazhuhongjingtian”[Title/Abstract]

#2 "myocardial infarction"[MeSH Terms] OR “Acute Coronary Syndrome”[MeSH Terms] OR "myocardial infarct*"[Title/Abstract] OR “Acute Coronary Syndrome”[Title/Abstract] OR “ACS”[Title/Abstract] OR “Acute Coronary Syndrome*”[Title/Abstract]OR “NSTE-ACS”[Title/Abstract] OR “Non–ST-Segment Elevation Acute Coronary Syndrome*”[Title/Abstract] OR "heart infarct*"[Title/Abstract] OR "cardiac infarct*"[Title/Abstract] OR "cardiovascular stroke*"[Title/Abstract] OR "cardiogenic shock"[Title/Abstract] OR “cardiovascular shock” [Title/Abstract] OR “cardiogenic stroke*” [Title/Abstract] OR "heart attack*"[Title/Abstract] OR "STEMI"[Title/Abstract] OR "NSTEMI"[Title/Abstract] OR “ST-segment elevation myocardial infarction”[Title/Abstract] OR “Non-ST-segment elevation myocardial infarction”[Title/Abstract] OR “MINOCA”[Title/Abstract] OR “Myocardial infarction with no obstructive coronary atherosclerosis” [Title/Abstract] OR “xiongbi” [Title/Abstract] OR “xiongbixintong” [Title/Abstract] OR “xiongbi xintong” [Title/Abstract] OR “zhenxintong” [Title/Abstract] OR “cuxintong” [Title/Abstract] OR “Xintong” [Title/Abstract] OR “juexintong” [Title/Abstract]

#3 “percutaneous coronary intervention” [MeSH Terms] OR “percutaneous coronary intervention*”[Title/Abstract] OR “percutaneous coronary revascularization*” [Title/Abstract] OR “coronary atherectom*”[Title/Abstract] OR “transluminal coronary angioplast*”[Title/Abstract] OR “transluminal coronary balloon dilation”[Title/Abstract] OR “coronary balloon angioplast*”[Title/Abstract] OR “PCI”[Title/Abstract] OR “Percutaneous Transluminal Coronary Angioplast*”[Title/Abstract] OR “PTCA”[Title/Abstract] OR “Rotational Atherectom*”[Title/Abstract] OR

“Directional Coronary Atherectom*”[Title/Abstract] OR “Balloon dilation”[Title/Abstract] OR “Artery Stenting”[Title/Abstract]

#4 #2 AND #3

#1 AND #4

Embase

#1 'Dazhu Hongjingtian capsule*':ab,ti OR ‘Dazhu Hongjingtian’:ab,ti OR ‘Herba Rhodiolae':ab,ti OR ‘dazhuhongjingtian':ab,ti

#2 'heart infarction'/exp

#3 'Acute Coronary Syndrome'/exp

#4 'cardiac infarct':ab,ti OR 'cardiac infarction':ab,ti OR 'cardial infarct':ab,ti OR 'heart attack':ab,ti OR 'heart infarct':ab,ti OR 'heart micro infarction':ab,ti OR 'heart muscle infarction':ab,ti OR 'infarction, heart':ab,ti OR 'myocardial infarct':ab,ti OR 'myocardial infarction':ab,ti OR 'myocardium infarct':ab,ti OR 'myocardium infarction':ab,ti OR 'premonitory infarction sign':ab,ti OR 'second heart attack':ab,ti OR 'subendocardial infarction':ab,ti OR 'transmural cardiac infarction':ab,ti OR 'transmural heart infarction':ab,ti OR 'transmural infarction, heart':ab,ti OR 'heart infarction':ab,ti OR ‘Acute Coronary Syndrome’:ab,ti OR ‘ACS’:ab,ti OR ‘Acute Coronary Syndrome*’:ab,ti OR ‘NSTE-ACS’:ab,ti OR ‘Non–ST-Segment Elevation Acute Coronary Syndrome*’:ab,ti OR 'STEMI':ab,ti OR 'NSTEMI':ab,ti OR ‘ST-segment elevation myocardial infarction’:ab,ti OR ‘Non-ST-segment elevation myocardial infarction’:ab,ti OR ‘MINOCA’:ab,ti OR ‘Myocardial infarction with no obstructive coronary atherosclerosis’:ab,ti OR ‘xiongbi’:ab,ti OR ‘xiongbixintong’:ab,ti OR ‘xiongbi Xintong’:ab,ti OR ‘zhenxintong’:ab,ti OR ‘cuxintong’:ab,ti OR ‘Xintong’:ab,ti OR ‘juexintong’:ab,ti OR ‘cardiovascular shock’:ab,ti OR ‘cardiogenic stroke*’:ab,ti

#5 ‘percutaneous coronary intervention'/exp

#6 ‘percutaneous coronary intervention*':ab,ti OR 'percutaneous coronary revascularization*':ab,ti OR 'coronary atherectom*':ab,ti OR 'transluminal coronary angioplast*':ab,ti OR 'transluminal coronary balloon dilation':ab,ti OR 'coronary balloon angioplast*':ab,ti OR 'PCI':ab,ti OR 'Percutaneous Transluminal Coronary Angioplast*':ab,ti OR 'PTCA':ab,ti OR 'Rotational Atherectom*':ab,ti OR 'Directional Coronary Atherectom*':ab,ti OR 'Balloon dilation':ab,ti OR 'Artery Stenting’:ab,ti

#7 #2 OR #3

#8 #4 OR #7

#9 #5 OR #6

#10 #8 AND #9

#11 #1 AND #10

Cochrane

#1 (Dazhu Hongjingtian capsule*):ti,ab,kw OR (Dazhu Hongjingtian):ti,ab,kw OR (Herba Rhodiolae):ti,ab,kw OR (dazhuhongjingtian):ti,ab,kw

#2 MeSH descriptor: [Myocardial Infarction] explode all trees

#3 MeSH descriptor: [Acute Coronary Syndrome] explode all trees

#4 (myocardial infarct*):ti,ab,kw OR (heart infarct*):ti,ab,kw OR (cardiac infarct*):ti,ab,kw OR (cardiovascular stroke*):ti,ab,kw OR (cardiogenic shock):ti,ab,kw OR (heart attack*):ti,ab,kw OR (Acute Coronary Syndrome):ti,ab,kw OR (ACS):ti,ab,kw OR (Acute Coronary Syndrome*):ti,ab,kw OR (NSTE-ACS):ti,ab,kw OR (Non–ST-Segment Elevation Acute Coronary Syndrome*):ti,ab,kw OR (STEMI):ti,ab,kw OR (NSTEMI):ti,ab,kw OR (ST-segment elevation myocardial infarction):ti,ab,kw OR (Non-ST-segment elevation myocardial infarction):ti,ab,kw OR (MINOCA):ti,ab,kw OR (Myocardial infarction with no obstructive coronary atherosclerosis):ti,ab,kw OR (xiongbi):ti,ab,kw OR (xiongbixintong):ti,ab,kw OR (xiongbi Xintong):ti,ab,kw OR (zhenxintong):ti,ab,kw OR (cuxintong):ti,ab,kw OR (Xintong):ti,ab,kw OR (juexintong):ti,ab,kw OR (cardiovascular shock):ti,ab,kw OR (cardiogenic stroke*):ti,ab,kw

#5 MeSH descriptor: [Percutaneous Coronary Intervention] explode all trees

#6 (percutaneous coronary intervention):ti,ab,kw OR (percutaneous coronary intervention*):ti,ab,kw OR (percutaneous coronary revascularization*):ti,ab,kw OR (coronary atherectom*):ti,ab,kw OR (transluminal coronary angioplast*):ti,ab,kw OR (transluminal coronary balloon dilation):ti,ab,kw OR (coronary balloon angioplast*):ti,ab,kw OR (PCI):ti,ab,kw OR (Percutaneous Transluminal Coronary Angioplast*):ti,ab,kw OR (PTCA):ti,ab,kw OR (Rotational Atherectom*):ti,ab,kw OR (Directional Coronary Atherectom*):ti,ab,kw OR (Balloon dilation):ti,ab,kw OR (Artery Stenting):ti,ab,kw

#7 #2 OR #3

#8 #4 OR #7

#9 #5 OR #6

#10 #8 AND #9

#11 #1 AND #10

3.Danhong Injection

CNKI

(TKA=‘丹红注射液’ + ‘丹红’) and ((TKA=‘心肌梗死’ + ‘心梗’ + ‘心肌梗塞’ + ‘急性冠脉综合征’+ ‘ACS’ + ‘STEMI’ + ‘NSTEMI’ + ‘胸痹’ + ‘胸痹心痛’ + ‘真心痛’ + ‘卒心痛’ + ‘心痛’ + ‘猝心痛’ + ‘厥心痛’) and (TKA=‘PCI’ + ‘PTCA’ + ‘经皮冠状动脉介入治疗术’ + ‘经皮冠状动脉介入’ + ‘经皮冠状动脉’ + ‘冠脉介入’ + ‘经皮冠脉’ + ‘血管成形术’ + ‘支架置入术’ + ‘冠状动脉成形术’ + ‘球囊’ + ‘支架’))

WangFang Data

主题:(“丹红注射液” OR “丹红”) and ((主题:心肌梗死 OR 心梗 OR 心肌梗塞 OR 急性冠脉综合征 OR ACS OR STEMI OR NSTEMI OR 胸痹 OR 胸痹心痛 OR 真心痛 OR 卒心痛 OR 心痛 OR 猝心痛 OR 厥心痛) and 主题:(PCI OR PTCA OR 经皮冠状动脉介入治疗术 OR 经皮冠状动脉介入 OR 经皮冠状动脉 OR 冠脉介入 OR 经皮冠脉 OR 血管成形术 OR 支架置入术 OR 冠状动脉成形术 OR 球囊 OR 支架))

VIP

(M=(“丹红注射液” OR “丹红”) OR R=(“丹红注射液” OR “丹红”)) AND ((M=(心肌梗死 OR 心梗 OR 心肌梗塞 OR 急性冠脉综合征 OR STEMI OR NSTEMI OR 胸痹 OR 胸痹心痛 OR 真心痛 OR 卒心痛 OR 心痛 OR 猝心痛 OR 厥心痛) OR R=(心肌梗死 OR 心梗 OR 心肌梗塞 OR ACS OR STEMI OR NSTEMI OR 胸痹 OR 胸痹心痛 OR 真心痛 OR 卒心痛 OR 心痛 OR 猝心痛 OR 厥心痛)) AND (M=(PCI OR PTCA OR 经皮冠状动脉介入治疗术 OR 经皮冠状动脉介入 OR 经皮冠状动脉 OR 冠脉介入 OR 经皮冠脉 OR 血管成形术 OR 支架置入术 OR 冠状动脉成形术 OR 球囊 OR 支架) OR R=(PCI OR PTCA OR 经皮冠状动脉介入治疗术 OR 经皮冠状动脉介入 OR 经皮冠状动脉 OR 冠脉介入 OR 经皮冠脉 OR 血管成形术 OR 支架置入术 OR 冠状动脉成形术 OR 球囊 OR 支架)))

Web of Science

#1 TS=(danhong injection OR danhong)

#2 TS=(myocardial infarct* OR heart infarct* OR cardiac infarct* OR cardiovascular stroke* OR cardiogenic shock OR heart attack* OR ACS OR STEMI OR NSTEMI OR ST-segment elevation myocardial infarction OR Non-ST-segment elevation myocardial infarction OR MINOCA OR Myocardial infarction with no obstructive coronary atherosclerosis OR xiongbi OR xiongbixintong OR xiongbi Xintong OR zhenxintong OR cuxintong OR Xintong OR juexintong OR cardiovascular shock OR cardiogenic stroke*)

#3 TS=(percutaneous coronary intervention OR percutaneous coronary intervention* OR percutaneous coronary revascularization* OR coronary atherectom* OR transluminal coronary angioplast* OR transluminal coronary balloon dilation OR coronary balloon angioplast* OR PCI OR Percutaneous Transluminal Coronary Angioplast* OR PTCA OR Rotational Atherectom* OR Directional Coronary Atherectom* OR Balloon dilation OR Artery Stenting)

#4 #2 AND #3

#5 #1 AND #4

PubMed

#1 “Danhong injection”[Title/Abstract] OR “Danhong”[Title/Abstract]

#2 "myocardial infarction"[MeSH Terms] OR “Acute Coronary Syndrome”[MeSH Terms] OR "myocardial infarct*"[Title/Abstract] OR “Acute Coronary Syndrome”[Title/Abstract] OR “ACS”[Title/Abstract] OR “Acute Coronary Syndrome*”[Title/Abstract]OR “NSTE-ACS”[Title/Abstract] OR “Non–ST-Segment Elevation Acute Coronary Syndrome*”[Title/Abstract] OR "heart infarct*"[Title/Abstract] OR "cardiac infarct*"[Title/Abstract] OR "cardiovascular stroke*"[Title/Abstract] OR "cardiogenic shock"[Title/Abstract] OR “cardiovascular shock” [Title/Abstract] OR “cardiogenic stroke*” [Title/Abstract] OR "heart attack*"[Title/Abstract] OR "STEMI"[Title/Abstract] OR "NSTEMI"[Title/Abstract] OR “ST-segment elevation myocardial infarction”[Title/Abstract] OR “Non-ST-segment elevation myocardial infarction”[Title/Abstract] OR “MINOCA”[Title/Abstract] OR “Myocardial infarction with no obstructive coronary atherosclerosis” [Title/Abstract] OR “xiongbi” [Title/Abstract] OR “xiongbixintong” [Title/Abstract] OR “xiongbi xintong” [Title/Abstract] OR “zhenxintong” [Title/Abstract] OR “cuxintong” [Title/Abstract] OR “Xintong” [Title/Abstract] OR “juexintong” [Title/Abstract]

#3 “percutaneous coronary intervention” [MeSH Terms] OR “percutaneous coronary intervention*”[Title/Abstract] OR “percutaneous coronary revascularization*” [Title/Abstract] OR “coronary atherectom*”[Title/Abstract] OR “transluminal coronary angioplast*”[Title/Abstract] OR “transluminal coronary balloon dilation”[Title/Abstract] OR “coronary balloon angioplast*”[Title/Abstract] OR “PCI”[Title/Abstract] OR “Percutaneous Transluminal Coronary Angioplast*”[Title/Abstract] OR “PTCA”[Title/Abstract] OR “Rotational Atherectom*”[Title/Abstract] OR

“Directional Coronary Atherectom*”[Title/Abstract] OR “Balloon dilation”[Title/Abstract] OR “Artery Stenting”[Title/Abstract]

#4 #2 AND #3

#1 AND #4

Embase

#1 'Danhong injection':ab,ti OR ‘Danhong’:ab,ti

#2 'heart infarction'/exp

#3 'Acute Coronary Syndrome'/exp

#4 'cardiac infarct':ab,ti OR 'cardiac infarction':ab,ti OR 'cardial infarct':ab,ti OR 'heart attack':ab,ti OR 'heart infarct':ab,ti OR 'heart micro infarction':ab,ti OR 'heart muscle infarction':ab,ti OR 'infarction, heart':ab,ti OR 'myocardial infarct':ab,ti OR 'myocardial infarction':ab,ti OR 'myocardium infarct':ab,ti OR 'myocardium infarction':ab,ti OR 'premonitory infarction sign':ab,ti OR 'second heart attack':ab,ti OR 'subendocardial infarction':ab,ti OR 'transmural cardiac infarction':ab,ti OR 'transmural heart infarction':ab,ti OR 'transmural infarction, heart':ab,ti OR 'heart infarction':ab,ti OR ‘Acute Coronary Syndrome’:ab,ti OR ‘ACS’:ab,ti OR ‘Acute Coronary Syndrome*’:ab,ti OR ‘NSTE-ACS’:ab,ti OR ‘Non–ST-Segment Elevation Acute Coronary Syndrome*’:ab,ti OR 'STEMI':ab,ti OR 'NSTEMI':ab,ti OR ‘ST-segment elevation myocardial infarction’:ab,ti OR ‘Non-ST-segment elevation myocardial infarction’:ab,ti OR ‘MINOCA’:ab,ti OR ‘Myocardial infarction with no obstructive coronary atherosclerosis’:ab,ti OR ‘xiongbi’:ab,ti OR ‘xiongbixintong’:ab,ti OR ‘xiongbi Xintong’:ab,ti OR ‘zhenxintong’:ab,ti OR ‘cuxintong’:ab,ti OR ‘Xintong’:ab,ti OR ‘juexintong’:ab,ti OR ‘cardiovascular shock’:ab,ti OR ‘cardiogenic stroke*’:ab,ti

#5 ‘percutaneous coronary intervention'/exp

#6 ‘percutaneous coronary intervention*':ab,ti OR 'percutaneous coronary revascularization*':ab,ti OR 'coronary atherectom*':ab,ti OR 'transluminal coronary angioplast*':ab,ti OR 'transluminal coronary balloon dilation':ab,ti OR 'coronary balloon angioplast*':ab,ti OR 'PCI':ab,ti OR 'Percutaneous Transluminal Coronary Angioplast*':ab,ti OR 'PTCA':ab,ti OR 'Rotational Atherectom*':ab,ti OR 'Directional Coronary Atherectom*':ab,ti OR 'Balloon dilation':ab,ti OR 'Artery Stenting’:ab,ti

#7 #2 OR #3

#8 #4 OR #7

#9 #5 OR #6

#10 #8 AND #9

#11 #1 AND #10

Cochrane

#1 (Danhong injection):ti,ab,kw OR (Danhong):ti,ab,kw

#2 MeSH descriptor: [Myocardial Infarction] explode all trees

#3 MeSH descriptor: [Acute Coronary Syndrome] explode all trees

#4 (myocardial infarct*):ti,ab,kw OR (heart infarct*):ti,ab,kw OR (cardiac infarct*):ti,ab,kw OR (cardiovascular stroke*):ti,ab,kw OR (cardiogenic shock):ti,ab,kw OR (heart attack*):ti,ab,kw OR (Acute Coronary Syndrome):ti,ab,kw OR (ACS):ti,ab,kw OR (Acute Coronary Syndrome*):ti,ab,kw OR (NSTE-ACS):ti,ab,kw OR (Non–ST-Segment Elevation Acute Coronary Syndrome*):ti,ab,kw OR (STEMI):ti,ab,kw OR (NSTEMI):ti,ab,kw OR (ST-segment elevation myocardial infarction):ti,ab,kw OR (Non-ST-segment elevation myocardial infarction):ti,ab,kw OR (MINOCA):ti,ab,kw OR (Myocardial infarction with no obstructive coronary atherosclerosis):ti,ab,kw OR (xiongbi):ti,ab,kw OR (xiongbixintong):ti,ab,kw OR (xiongbi Xintong):ti,ab,kw OR (zhenxintong):ti,ab,kw OR (cuxintong):ti,ab,kw OR (Xintong):ti,ab,kw OR (juexintong):ti,ab,kw OR (cardiovascular shock):ti,ab,kw OR (cardiogenic stroke*):ti,ab,kw

#5 MeSH descriptor: [Percutaneous Coronary Intervention] explode all trees

#6 (percutaneous coronary intervention):ti,ab,kw OR (percutaneous coronary intervention*):ti,ab,kw OR (percutaneous coronary revascularization*):ti,ab,kw OR (coronary atherectom*):ti,ab,kw OR (transluminal coronary angioplast*):ti,ab,kw OR (transluminal coronary balloon dilation):ti,ab,kw OR (coronary balloon angioplast*):ti,ab,kw OR (PCI):ti,ab,kw OR (Percutaneous Transluminal Coronary Angioplast*):ti,ab,kw OR (PTCA):ti,ab,kw OR (Rotational Atherectom*):ti,ab,kw OR (Directional Coronary Atherectom*):ti,ab,kw OR (Balloon dilation):ti,ab,kw OR (Artery Stenting):ti,ab,kw

#7 #2 OR #3

#8 #4 OR #7

#9 #5 OR #6

#10 #8 AND #9

#11 #1 AND #10

4.Danshen Injection

CNKI

(TKA=‘丹参注射液’ + ‘丹参’) and ((TKA=‘心肌梗死’ + ‘心梗’ + ‘心肌梗塞’ + ‘急性冠脉综合征’+ ‘ACS’ + ‘STEMI’ + ‘NSTEMI’ + ‘胸痹’ + ‘胸痹心痛’ + ‘真心痛’ + ‘卒心痛’ + ‘心痛’ + ‘猝心痛’ + ‘厥心痛’) and (TKA=‘PCI’ + ‘PTCA’ + ‘经皮冠状动脉介入治疗术’ + ‘经皮冠状动脉介入’ + ‘经皮冠状动脉’ + ‘冠脉介入’ + ‘经皮冠脉’ + ‘血管成形术’ + ‘支架置入术’ + ‘冠状动脉成形术’ + ‘球囊’ + ‘支架’))

WangFang Data

主题:(“丹参注射液” OR “丹参”) and ((主题:心肌梗死 OR 心梗 OR 心肌梗塞 OR 急性冠脉综合征 OR ACS OR STEMI OR NSTEMI OR 胸痹 OR 胸痹心痛 OR 真心痛 OR 卒心痛 OR 心痛 OR 猝心痛 OR 厥心痛) and 主题:(PCI OR PTCA OR 经皮冠状动脉介入治疗术 OR 经皮冠状动脉介入 OR 经皮冠状动脉 OR 冠脉介入 OR 经皮冠脉 OR 血管成形术 OR 支架置入术 OR 冠状动脉成形术 OR 球囊 OR 支架))

VIP

(M=(“丹参注射液” OR “丹参”) OR R=(“丹参注射液” OR “丹参”)) AND ((M=(心肌梗死 OR 心梗 OR 心肌梗塞 OR 急性冠脉综合征 OR STEMI OR NSTEMI OR 胸痹 OR 胸痹心痛 OR 真心痛 OR 卒心痛 OR 心痛 OR 猝心痛 OR 厥心痛) OR R=(心肌梗死 OR 心梗 OR 心肌梗塞 OR ACS OR STEMI OR NSTEMI OR 胸痹 OR 胸痹心痛 OR 真心痛 OR 卒心痛 OR 心痛 OR 猝心痛 OR 厥心痛)) AND (M=(PCI OR PTCA OR 经皮冠状动脉介入治疗术 OR 经皮冠状动脉介入 OR 经皮冠状动脉 OR 冠脉介入 OR 经皮冠脉 OR 血管成形术 OR 支架置入术 OR 冠状动脉成形术 OR 球囊 OR 支架) OR R=(PCI OR PTCA OR 经皮冠状动脉介入治疗术 OR 经皮冠状动脉介入 OR 经皮冠状动脉 OR 冠脉介入 OR 经皮冠脉 OR 血管成形术 OR 支架置入术 OR 冠状动脉成形术 OR 球囊 OR 支架)))

Web of Science

#1 TS=(danshen injection OR danshen)

#2 TS=(myocardial infarct* OR heart infarct* OR cardiac infarct* OR cardiovascular stroke* OR cardiogenic shock OR heart attack* OR ACS OR STEMI OR NSTEMI OR ST-segment elevation myocardial infarction OR Non-ST-segment elevation myocardial infarction OR MINOCA OR Myocardial infarction with no obstructive coronary atherosclerosis OR xiongbi OR xiongbixintong OR xiongbi Xintong OR zhenxintong OR cuxintong OR Xintong OR juexintong OR cardiovascular shock OR cardiogenic stroke*)

#3 TS=(percutaneous coronary intervention OR percutaneous coronary intervention* OR percutaneous coronary revascularization* OR coronary atherectom* OR transluminal coronary angioplast* OR transluminal coronary balloon dilation OR coronary balloon angioplast* OR PCI OR Percutaneous Transluminal Coronary Angioplast* OR PTCA OR Rotational Atherectom* OR Directional Coronary Atherectom* OR Balloon dilation OR Artery Stenting)

#4 #2 AND #3

#5 #1 AND #4

PubMed

#1 “Danshen injection”[Title/Abstract] OR “Danshen”[Title/Abstract]

#2 "myocardial infarction"[MeSH Terms] OR “Acute Coronary Syndrome”[MeSH Terms] OR "myocardial infarct*"[Title/Abstract] OR “Acute Coronary Syndrome”[Title/Abstract] OR “ACS”[Title/Abstract] OR “Acute Coronary Syndrome*”[Title/Abstract]OR “NSTE-ACS”[Title/Abstract] OR “Non–ST-Segment Elevation Acute Coronary Syndrome*”[Title/Abstract] OR "heart infarct*"[Title/Abstract] OR "cardiac infarct*"[Title/Abstract] OR "cardiovascular stroke*"[Title/Abstract] OR "cardiogenic shock"[Title/Abstract] OR “cardiovascular shock” [Title/Abstract] OR “cardiogenic stroke*” [Title/Abstract] OR "heart attack*"[Title/Abstract] OR "STEMI"[Title/Abstract] OR "NSTEMI"[Title/Abstract] OR “ST-segment elevation myocardial infarction”[Title/Abstract] OR “Non-ST-segment elevation myocardial infarction”[Title/Abstract] OR “MINOCA”[Title/Abstract] OR “Myocardial infarction with no obstructive coronary atherosclerosis” [Title/Abstract] OR “xiongbi” [Title/Abstract] OR “xiongbixintong” [Title/Abstract] OR “xiongbi xintong” [Title/Abstract] OR “zhenxintong” [Title/Abstract] OR “cuxintong” [Title/Abstract] OR “Xintong” [Title/Abstract] OR “juexintong” [Title/Abstract]

#3 “percutaneous coronary intervention” [MeSH Terms] OR “percutaneous coronary intervention*”[Title/Abstract] OR “percutaneous coronary revascularization*” [Title/Abstract] OR “coronary atherectom*”[Title/Abstract] OR “transluminal coronary angioplast*”[Title/Abstract] OR “transluminal coronary balloon dilation”[Title/Abstract] OR “coronary balloon angioplast*”[Title/Abstract] OR “PCI”[Title/Abstract] OR “Percutaneous Transluminal Coronary Angioplast*”[Title/Abstract] OR “PTCA”[Title/Abstract] OR “Rotational Atherectom*”[Title/Abstract] OR

“Directional Coronary Atherectom*”[Title/Abstract] OR “Balloon dilation”[Title/Abstract] OR “Artery Stenting”[Title/Abstract]

#4 #2 AND #3

#1 AND #4

Embase

#1 'Danshen injection':ab,ti OR ‘Danshen’:ab,ti

#2 'heart infarction'/exp

#3 'Acute Coronary Syndrome'/exp

#4 'cardiac infarct':ab,ti OR 'cardiac infarction':ab,ti OR 'cardial infarct':ab,ti OR 'heart attack':ab,ti OR 'heart infarct':ab,ti OR 'heart micro infarction':ab,ti OR 'heart muscle infarction':ab,ti OR 'infarction, heart':ab,ti OR 'myocardial infarct':ab,ti OR 'myocardial infarction':ab,ti OR 'myocardium infarct':ab,ti OR 'myocardium infarction':ab,ti OR 'premonitory infarction sign':ab,ti OR 'second heart attack':ab,ti OR 'subendocardial infarction':ab,ti OR 'transmural cardiac infarction':ab,ti OR 'transmural heart infarction':ab,ti OR 'transmural infarction, heart':ab,ti OR 'heart infarction':ab,ti OR ‘Acute Coronary Syndrome’:ab,ti OR ‘ACS’:ab,ti OR ‘Acute Coronary Syndrome*’:ab,ti OR ‘NSTE-ACS’:ab,ti OR ‘Non–ST-Segment Elevation Acute Coronary Syndrome*’:ab,ti OR 'STEMI':ab,ti OR 'NSTEMI':ab,ti OR ‘ST-segment elevation myocardial infarction’:ab,ti OR ‘Non-ST-segment elevation myocardial infarction’:ab,ti OR ‘MINOCA’:ab,ti OR ‘Myocardial infarction with no obstructive coronary atherosclerosis’:ab,ti OR ‘xiongbi’:ab,ti OR ‘xiongbixintong’:ab,ti OR ‘xiongbi Xintong’:ab,ti OR ‘zhenxintong’:ab,ti OR ‘cuxintong’:ab,ti OR ‘Xintong’:ab,ti OR ‘juexintong’:ab,ti OR ‘cardiovascular shock’:ab,ti OR ‘cardiogenic stroke*’:ab,ti

#5 ‘percutaneous coronary intervention'/exp

#6 ‘percutaneous coronary intervention*':ab,ti OR 'percutaneous coronary revascularization*':ab,ti OR 'coronary atherectom*':ab,ti OR 'transluminal coronary angioplast*':ab,ti OR 'transluminal coronary balloon dilation':ab,ti OR 'coronary balloon angioplast*':ab,ti OR 'PCI':ab,ti OR 'Percutaneous Transluminal Coronary Angioplast*':ab,ti OR 'PTCA':ab,ti OR 'Rotational Atherectom*':ab,ti OR 'Directional Coronary Atherectom*':ab,ti OR 'Balloon dilation':ab,ti OR 'Artery Stenting’:ab,ti

#7 #2 OR #3

#8 #4 OR #7

#9 #5 OR #6

#10 #8 AND #9

#11 #1 AND #10

Cochrane

#1 (Danshen injection):ti,ab,kw OR (Danshen):ti,ab,kw

#2 MeSH descriptor: [Myocardial Infarction] explode all trees

#3 MeSH descriptor: [Acute Coronary Syndrome] explode all trees

#4 (myocardial infarct*):ti,ab,kw OR (heart infarct*):ti,ab,kw OR (cardiac infarct*):ti,ab,kw OR (cardiovascular stroke*):ti,ab,kw OR (cardiogenic shock):ti,ab,kw OR (heart attack*):ti,ab,kw OR (Acute Coronary Syndrome):ti,ab,kw OR (ACS):ti,ab,kw OR (Acute Coronary Syndrome*):ti,ab,kw OR (NSTE-ACS):ti,ab,kw OR (Non–ST-Segment Elevation Acute Coronary Syndrome*):ti,ab,kw OR (STEMI):ti,ab,kw OR (NSTEMI):ti,ab,kw OR (ST-segment elevation myocardial infarction):ti,ab,kw OR (Non-ST-segment elevation myocardial infarction):ti,ab,kw OR (MINOCA):ti,ab,kw OR (Myocardial infarction with no obstructive coronary atherosclerosis):ti,ab,kw OR (xiongbi):ti,ab,kw OR (xiongbixintong):ti,ab,kw OR (xiongbi Xintong):ti,ab,kw OR (zhenxintong):ti,ab,kw OR (cuxintong):ti,ab,kw OR (Xintong):ti,ab,kw OR (juexintong):ti,ab,kw OR (cardiovascular shock):ti,ab,kw OR (cardiogenic stroke*):ti,ab,kw

#5 MeSH descriptor: [Percutaneous Coronary Intervention] explode all trees

#6 (percutaneous coronary intervention):ti,ab,kw OR (percutaneous coronary intervention*):ti,ab,kw OR (percutaneous coronary revascularization*):ti,ab,kw OR (coronary atherectom*):ti,ab,kw OR (transluminal coronary angioplast*):ti,ab,kw OR (transluminal coronary balloon dilation):ti,ab,kw OR (coronary balloon angioplast*):ti,ab,kw OR (PCI):ti,ab,kw OR (Percutaneous Transluminal Coronary Angioplast*):ti,ab,kw OR (PTCA):ti,ab,kw OR (Rotational Atherectom*):ti,ab,kw OR (Directional Coronary Atherectom*):ti,ab,kw OR (Balloon dilation):ti,ab,kw OR (Artery Stenting):ti,ab,kw

#7 #2 OR #3

#8 #4 OR #7

#9 #5 OR #6

#10 #8 AND #9

#11 #1 AND #10

5.Danshen Dizhuye

CNKI

(TKA=‘丹参滴注液’ + ‘丹参’) and ((TKA=‘心肌梗死’ + ‘心梗’ + ‘心肌梗塞’ + ‘急性冠脉综合征’+ ‘ACS’ + ‘STEMI’ + ‘NSTEMI’ + ‘胸痹’ + ‘胸痹心痛’ + ‘真心痛’ + ‘卒心痛’ + ‘心痛’ + ‘猝心痛’ + ‘厥心痛’) and (TKA=‘PCI’ + ‘PTCA’ + ‘经皮冠状动脉介入治疗术’ + ‘经皮冠状动脉介入’ + ‘经皮冠状动脉’ + ‘冠脉介入’ + ‘经皮冠脉’ + ‘血管成形术’ + ‘支架置入术’ + ‘冠状动脉成形术’ + ‘球囊’ + ‘支架’))

WangFang Data

主题:(“丹参滴注液” OR “丹参”) and ((主题:心肌梗死 OR 心梗 OR 心肌梗塞 OR 急性冠脉综合征 OR ACS OR STEMI OR NSTEMI OR 胸痹 OR 胸痹心痛 OR 真心痛 OR 卒心痛 OR 心痛 OR 猝心痛 OR 厥心痛) and 主题:(PCI OR PTCA OR 经皮冠状动脉介入治疗术 OR 经皮冠状动脉介入 OR 经皮冠状动脉 OR 冠脉介入 OR 经皮冠脉 OR 血管成形术 OR 支架置入术 OR 冠状动脉成形术 OR 球囊 OR 支架))

VIP

(M=(“丹参滴注液” OR “丹参”) OR R=(“丹参滴注液” OR “丹参”)) AND ((M=(心肌梗死 OR 心梗 OR 心肌梗塞 OR 急性冠脉综合征 OR STEMI OR NSTEMI OR 胸痹 OR 胸痹心痛 OR 真心痛 OR 卒心痛 OR 心痛 OR 猝心痛 OR 厥心痛) OR R=(心肌梗死 OR 心梗 OR 心肌梗塞 OR ACS OR STEMI OR NSTEMI OR 胸痹 OR 胸痹心痛 OR 真心痛 OR 卒心痛 OR 心痛 OR 猝心痛 OR 厥心痛)) AND (M=(PCI OR PTCA OR 经皮冠状动脉介入治疗术 OR 经皮冠状动脉介入 OR 经皮冠状动脉 OR 冠脉介入 OR 经皮冠脉 OR 血管成形术 OR 支架置入术 OR 冠状动脉成形术 OR 球囊 OR 支架) OR R=(PCI OR PTCA OR 经皮冠状动脉介入治疗术 OR 经皮冠状动脉介入 OR 经皮冠状动脉 OR 冠脉介入 OR 经皮冠脉 OR 血管成形术 OR 支架置入术 OR 冠状动脉成形术 OR 球囊 OR 支架)))

Web of Science

#1 TS=(Danshen Dizhuye OR Dan Shen Di Zhu Ye OR Danshen)

#2 TS=(myocardial infarct* OR heart infarct* OR cardiac infarct* OR cardiovascular stroke* OR cardiogenic shock OR heart attack* OR ACS OR STEMI OR NSTEMI OR ST-segment elevation myocardial infarction OR Non-ST-segment elevation myocardial infarction OR MINOCA OR Myocardial infarction with no obstructive coronary atherosclerosis OR xiongbi OR xiongbixintong OR xiongbi Xintong OR zhenxintong OR cuxintong OR Xintong OR juexintong OR cardiovascular shock OR cardiogenic stroke*)

#3 TS=(percutaneous coronary intervention OR percutaneous coronary intervention* OR percutaneous coronary revascularization* OR coronary atherectom* OR transluminal coronary angioplast* OR transluminal coronary balloon dilation OR coronary balloon angioplast* OR PCI OR Percutaneous Transluminal Coronary Angioplast* OR PTCA OR Rotational Atherectom* OR Directional Coronary Atherectom* OR Balloon dilation OR Artery Stenting)

#4 #2 AND #3

#5 #1 AND #4

PubMed

#1 “Danshen Dizhuye”[Title/Abstract] OR “Dan Shen Di Zhu Ye”[Title/Abstract] OR “Danshen”[Title/Abstract]

#2 "myocardial infarction"[MeSH Terms] OR “Acute Coronary Syndrome”[MeSH Terms] OR "myocardial infarct*"[Title/Abstract] OR “Acute Coronary Syndrome”[Title/Abstract] OR “ACS”[Title/Abstract] OR “Acute Coronary Syndrome*”[Title/Abstract]OR “NSTE-ACS”[Title/Abstract] OR “Non–ST-Segment Elevation Acute Coronary Syndrome*”[Title/Abstract] OR "heart infarct*"[Title/Abstract] OR "cardiac infarct*"[Title/Abstract] OR "cardiovascular stroke*"[Title/Abstract] OR "cardiogenic shock"[Title/Abstract] OR “cardiovascular shock” [Title/Abstract] OR “cardiogenic stroke*” [Title/Abstract] OR "heart attack*"[Title/Abstract] OR "STEMI"[Title/Abstract] OR "NSTEMI"[Title/Abstract] OR “ST-segment elevation myocardial infarction”[Title/Abstract] OR “Non-ST-segment elevation myocardial infarction”[Title/Abstract] OR “MINOCA”[Title/Abstract] OR “Myocardial infarction with no obstructive coronary atherosclerosis” [Title/Abstract] OR “xiongbi” [Title/Abstract] OR “xiongbixintong” [Title/Abstract] OR “xiongbi xintong” [Title/Abstract] OR “zhenxintong” [Title/Abstract] OR “cuxintong” [Title/Abstract] OR “Xintong” [Title/Abstract] OR “juexintong” [Title/Abstract]

#3 “percutaneous coronary intervention” [MeSH Terms] OR “percutaneous coronary intervention*”[Title/Abstract] OR “percutaneous coronary revascularization*” [Title/Abstract] OR “coronary atherectom*”[Title/Abstract] OR “transluminal coronary angioplast*”[Title/Abstract] OR “transluminal coronary balloon dilation”[Title/Abstract] OR “coronary balloon angioplast*”[Title/Abstract] OR “PCI”[Title/Abstract] OR “Percutaneous Transluminal Coronary Angioplast*”[Title/Abstract] OR “PTCA”[Title/Abstract] OR “Rotational Atherectom*”[Title/Abstract] OR

“Directional Coronary Atherectom*”[Title/Abstract] OR “Balloon dilation”[Title/Abstract] OR “Artery Stenting”[Title/Abstract]

#4 #2 AND #3

#1 AND #4

Embase

#1 'Danshen Dizhuye':ab,ti OR ‘Dan Shen Di Zhu Ye’:ab,ti OR ‘Danshen’:ab,ti

#2 'heart infarction'/exp

#3 'Acute Coronary Syndrome'/exp

#4 'cardiac infarct':ab,ti OR 'cardiac infarction':ab,ti OR 'cardial infarct':ab,ti OR 'heart attack':ab,ti OR 'heart infarct':ab,ti OR 'heart micro infarction':ab,ti OR 'heart muscle infarction':ab,ti OR 'infarction, heart':ab,ti OR 'myocardial infarct':ab,ti OR 'myocardial infarction':ab,ti OR 'myocardium infarct':ab,ti OR 'myocardium infarction':ab,ti OR 'premonitory infarction sign':ab,ti OR 'second heart attack':ab,ti OR 'subendocardial infarction':ab,ti OR 'transmural cardiac infarction':ab,ti OR 'transmural heart infarction':ab,ti OR 'transmural infarction, heart':ab,ti OR 'heart infarction':ab,ti OR ‘Acute Coronary Syndrome’:ab,ti OR ‘ACS’:ab,ti OR ‘Acute Coronary Syndrome*’:ab,ti OR ‘NSTE-ACS’:ab,ti OR ‘Non–ST-Segment Elevation Acute Coronary Syndrome*’:ab,ti OR 'STEMI':ab,ti OR 'NSTEMI':ab,ti OR ‘ST-segment elevation myocardial infarction’:ab,ti OR ‘Non-ST-segment elevation myocardial infarction’:ab,ti OR ‘MINOCA’:ab,ti OR ‘Myocardial infarction with no obstructive coronary atherosclerosis’:ab,ti OR ‘xiongbi’:ab,ti OR ‘xiongbixintong’:ab,ti OR ‘xiongbi Xintong’:ab,ti OR ‘zhenxintong’:ab,ti OR ‘cuxintong’:ab,ti OR ‘Xintong’:ab,ti OR ‘juexintong’:ab,ti OR ‘cardiovascular shock’:ab,ti OR ‘cardiogenic stroke*’:ab,ti

#5 ‘percutaneous coronary intervention'/exp

#6 ‘percutaneous coronary intervention*':ab,ti OR 'percutaneous coronary revascularization*':ab,ti OR 'coronary atherectom*':ab,ti OR 'transluminal coronary angioplast*':ab,ti OR 'transluminal coronary balloon dilation':ab,ti OR 'coronary balloon angioplast*':ab,ti OR 'PCI':ab,ti OR 'Percutaneous Transluminal Coronary Angioplast*':ab,ti OR 'PTCA':ab,ti OR 'Rotational Atherectom*':ab,ti OR 'Directional Coronary Atherectom*':ab,ti OR 'Balloon dilation':ab,ti OR 'Artery Stenting’:ab,ti

#7 #2 OR #3

#8 #4 OR #7

#9 #5 OR #6

#10 #8 AND #9

#11 #1 AND #10

Cochrane

#1 (Danshen Dizhuye):ti,ab,kw OR (Dan Shen Di Zhu Ye):ti,ab,kw OR (Danshen):ti,ab,kw

#2 MeSH descriptor: [Myocardial Infarction] explode all trees

#3 MeSH descriptor: [Acute Coronary Syndrome] explode all trees

#4 (myocardial infarct*):ti,ab,kw OR (heart infarct*):ti,ab,kw OR (cardiac infarct*):ti,ab,kw OR (cardiovascular stroke*):ti,ab,kw OR (cardiogenic shock):ti,ab,kw OR (heart attack*):ti,ab,kw OR (Acute Coronary Syndrome):ti,ab,kw OR (ACS):ti,ab,kw OR (Acute Coronary Syndrome*):ti,ab,kw OR (NSTE-ACS):ti,ab,kw OR (Non–ST-Segment Elevation Acute Coronary Syndrome*):ti,ab,kw OR (STEMI):ti,ab,kw OR (NSTEMI):ti,ab,kw OR (ST-segment elevation myocardial infarction):ti,ab,kw OR (Non-ST-segment elevation myocardial infarction):ti,ab,kw OR (MINOCA):ti,ab,kw OR (Myocardial infarction with no obstructive coronary atherosclerosis):ti,ab,kw OR (xiongbi):ti,ab,kw OR (xiongbixintong):ti,ab,kw OR (xiongbi Xintong):ti,ab,kw OR (zhenxintong):ti,ab,kw OR (cuxintong):ti,ab,kw OR (Xintong):ti,ab,kw OR (juexintong):ti,ab,kw OR (cardiovascular shock):ti,ab,kw OR (cardiogenic stroke*):ti,ab,kw

#5 MeSH descriptor: [Percutaneous Coronary Intervention] explode all trees

#6 (percutaneous coronary intervention):ti,ab,kw OR (percutaneous coronary intervention*):ti,ab,kw OR (percutaneous coronary revascularization*):ti,ab,kw OR (coronary atherectom*):ti,ab,kw OR (transluminal coronary angioplast*):ti,ab,kw OR (transluminal coronary balloon dilation):ti,ab,kw OR (coronary balloon angioplast*):ti,ab,kw OR (PCI):ti,ab,kw OR (Percutaneous Transluminal Coronary Angioplast*):ti,ab,kw OR (PTCA):ti,ab,kw OR (Rotational Atherectom*):ti,ab,kw OR (Directional Coronary Atherectom*):ti,ab,kw OR (Balloon dilation):ti,ab,kw OR (Artery Stenting):ti,ab,kw

#7 #2 OR #3

#8 #4 OR #7

#9 #5 OR #6

#10 #8 AND #9

#11 #1 AND #10

6.Danxiang Guanxin Injection

CNKI

(TKA=‘丹香冠心注射液’ + ‘丹香冠心’) and ((TKA=‘心肌梗死’ + ‘心梗’ + ‘心肌梗塞’ + ‘急性冠脉综合征’+ ‘ACS’ + ‘STEMI’ + ‘NSTEMI’ + ‘胸痹’ + ‘胸痹心痛’ + ‘真心痛’ + ‘卒心痛’ + ‘心痛’ + ‘猝心痛’ + ‘厥心痛’) and (TKA=‘PCI’ + ‘PTCA’ + ‘经皮冠状动脉介入治疗术’ + ‘经皮冠状动脉介入’ + ‘经皮冠状动脉’ + ‘冠脉介入’ + ‘经皮冠脉’ + ‘血管成形术’ + ‘支架置入术’ + ‘冠状动脉成形术’ + ‘球囊’ + ‘支架’))

WangFang Data

主题:(“丹香冠心注射液” OR “丹香冠心”) and ((主题:心肌梗死 OR 心梗 OR 心肌梗塞 OR 急性冠脉综合征 OR ACS OR STEMI OR NSTEMI OR 胸痹 OR 胸痹心痛 OR 真心痛 OR 卒心痛 OR 心痛 OR 猝心痛 OR 厥心痛) and 主题:(PCI OR PTCA OR 经皮冠状动脉介入治疗术 OR 经皮冠状动脉介入 OR 经皮冠状动脉 OR 冠脉介入 OR 经皮冠脉 OR 血管成形术 OR 支架置入术 OR 冠状动脉成形术 OR 球囊 OR 支架))

VIP

(M=(“丹香冠心注射液” OR “丹香冠心”) OR R=(“丹香冠心注射液” OR “丹香冠心”)) AND ((M=(心肌梗死 OR 心梗 OR 心肌梗塞 OR 急性冠脉综合征 OR STEMI OR NSTEMI OR 胸痹 OR 胸痹心痛 OR 真心痛 OR 卒心痛 OR 心痛 OR 猝心痛 OR 厥心痛) OR R=(心肌梗死 OR 心梗 OR 心肌梗塞 OR ACS OR STEMI OR NSTEMI OR 胸痹 OR 胸痹心痛 OR 真心痛 OR 卒心痛 OR 心痛 OR 猝心痛 OR 厥心痛)) AND (M=(PCI OR PTCA OR 经皮冠状动脉介入治疗术 OR 经皮冠状动脉介入 OR 经皮冠状动脉 OR 冠脉介入 OR 经皮冠脉 OR 血管成形术 OR 支架置入术 OR 冠状动脉成形术 OR 球囊 OR 支架) OR R=(PCI OR PTCA OR 经皮冠状动脉介入治疗术 OR 经皮冠状动脉介入 OR 经皮冠状动脉 OR 冠脉介入 OR 经皮冠脉 OR 血管成形术 OR 支架置入术 OR 冠状动脉成形术 OR 球囊 OR 支架)))

Web of Science

#1 TS=(Danxiang Guanxin injection OR danxiangguanxin OR Danxiang Guanxin)

#2 TS=(myocardial infarct* OR heart infarct* OR cardiac infarct* OR cardiovascular stroke* OR cardiogenic shock OR heart attack* OR ACS OR STEMI OR NSTEMI OR ST-segment elevation myocardial infarction OR Non-ST-segment elevation myocardial infarction OR MINOCA OR Myocardial infarction with no obstructive coronary atherosclerosis OR xiongbi OR xiongbixintong OR xiongbi Xintong OR zhenxintong OR cuxintong OR Xintong OR juexintong OR cardiovascular shock OR cardiogenic stroke*)

#3 TS=(percutaneous coronary intervention OR percutaneous coronary intervention* OR percutaneous coronary revascularization* OR coronary atherectom* OR transluminal coronary angioplast* OR transluminal coronary balloon dilation OR coronary balloon angioplast* OR PCI OR Percutaneous Transluminal Coronary Angioplast* OR PTCA OR Rotational Atherectom* OR Directional Coronary Atherectom* OR Balloon dilation OR Artery Stenting)

#4 #2 AND #3

#5 #1 AND #4

PubMed

#1 “Danxiang Guanxin injection”[Title/Abstract] OR “danxiangguanxin”[Title/Abstract] OR “Danxiang Guanxin”[Title/Abstract]

#2 "myocardial infarction"[MeSH Terms] OR “Acute Coronary Syndrome”[MeSH Terms] OR "myocardial infarct*"[Title/Abstract] OR “Acute Coronary Syndrome”[Title/Abstract] OR “ACS”[Title/Abstract] OR “Acute Coronary Syndrome*”[Title/Abstract]OR “NSTE-ACS”[Title/Abstract] OR “Non–ST-Segment Elevation Acute Coronary Syndrome*”[Title/Abstract] OR "heart infarct*"[Title/Abstract] OR "cardiac infarct*"[Title/Abstract] OR "cardiovascular stroke*"[Title/Abstract] OR "cardiogenic shock"[Title/Abstract] OR “cardiovascular shock” [Title/Abstract] OR “cardiogenic stroke*” [Title/Abstract] OR "heart attack*"[Title/Abstract] OR "STEMI"[Title/Abstract] OR "NSTEMI"[Title/Abstract] OR “ST-segment elevation myocardial infarction”[Title/Abstract] OR “Non-ST-segment elevation myocardial infarction”[Title/Abstract] OR “MINOCA”[Title/Abstract] OR “Myocardial infarction with no obstructive coronary atherosclerosis” [Title/Abstract] OR “xiongbi” [Title/Abstract] OR “xiongbixintong” [Title/Abstract] OR “xiongbi xintong” [Title/Abstract] OR “zhenxintong” [Title/Abstract] OR “cuxintong” [Title/Abstract] OR “Xintong” [Title/Abstract] OR “juexintong” [Title/Abstract]

#3 “percutaneous coronary intervention” [MeSH Terms] OR “percutaneous coronary intervention*”[Title/Abstract] OR “percutaneous coronary revascularization*” [Title/Abstract] OR “coronary atherectom*”[Title/Abstract] OR “transluminal coronary angioplast*”[Title/Abstract] OR “transluminal coronary balloon dilation”[Title/Abstract] OR “coronary balloon angioplast*”[Title/Abstract] OR “PCI”[Title/Abstract] OR “Percutaneous Transluminal Coronary Angioplast*”[Title/Abstract] OR “PTCA”[Title/Abstract] OR “Rotational Atherectom*”[Title/Abstract] OR

“Directional Coronary Atherectom*”[Title/Abstract] OR “Balloon dilation”[Title/Abstract] OR “Artery Stenting”[Title/Abstract]

#4 #2 AND #3

#1 AND #4

Embase

#1 'Danxiang Guanxin injection':ab,ti OR ‘danxiangguanxin’:ab,ti OR ‘Danxiang Guanxin’:ab,ti

#2 'heart infarction'/exp

#3 'Acute Coronary Syndrome'/exp

#4 'cardiac infarct':ab,ti OR 'cardiac infarction':ab,ti OR 'cardial infarct':ab,ti OR 'heart attack':ab,ti OR 'heart infarct':ab,ti OR 'heart micro infarction':ab,ti OR 'heart muscle infarction':ab,ti OR 'infarction, heart':ab,ti OR 'myocardial infarct':ab,ti OR 'myocardial infarction':ab,ti OR 'myocardium infarct':ab,ti OR 'myocardium infarction':ab,ti OR 'premonitory infarction sign':ab,ti OR 'second heart attack':ab,ti OR 'subendocardial infarction':ab,ti OR 'transmural cardiac infarction':ab,ti OR 'transmural heart infarction':ab,ti OR 'transmural infarction, heart':ab,ti OR 'heart infarction':ab,ti OR ‘Acute Coronary Syndrome’:ab,ti OR ‘ACS’:ab,ti OR ‘Acute Coronary Syndrome*’:ab,ti OR ‘NSTE-ACS’:ab,ti OR ‘Non–ST-Segment Elevation Acute Coronary Syndrome*’:ab,ti OR 'STEMI':ab,ti OR 'NSTEMI':ab,ti OR ‘ST-segment elevation myocardial infarction’:ab,ti OR ‘Non-ST-segment elevation myocardial infarction’:ab,ti OR ‘MINOCA’:ab,ti OR ‘Myocardial infarction with no obstructive coronary atherosclerosis’:ab,ti OR ‘xiongbi’:ab,ti OR ‘xiongbixintong’:ab,ti OR ‘xiongbi Xintong’:ab,ti OR ‘zhenxintong’:ab,ti OR ‘cuxintong’:ab,ti OR ‘Xintong’:ab,ti OR ‘juexintong’:ab,ti OR ‘cardiovascular shock’:ab,ti OR ‘cardiogenic stroke*’:ab,ti

#5 ‘percutaneous coronary intervention'/exp

#6 ‘percutaneous coronary intervention*':ab,ti OR 'percutaneous coronary revascularization*':ab,ti OR 'coronary atherectom*':ab,ti OR 'transluminal coronary angioplast*':ab,ti OR 'transluminal coronary balloon dilation':ab,ti OR 'coronary balloon angioplast*':ab,ti OR 'PCI':ab,ti OR 'Percutaneous Transluminal Coronary Angioplast*':ab,ti OR 'PTCA':ab,ti OR 'Rotational Atherectom*':ab,ti OR 'Directional Coronary Atherectom*':ab,ti OR 'Balloon dilation':ab,ti OR 'Artery Stenting’:ab,ti

#7 #2 OR #3

#8 #4 OR #7

#9 #5 OR #6

#10 #8 AND #9

#11 #1 AND #10

Cochrane

#1 (Danxiang Guanxin injection):ti,ab,kw OR (danxiangguanxin):ti,ab,kw OR (Danxiang Guanxin):ti,ab,kw

#2 MeSH descriptor: [Myocardial Infarction] explode all trees

#3 MeSH descriptor: [Acute Coronary Syndrome] explode all trees

#4 (myocardial infarct*):ti,ab,kw OR (heart infarct*):ti,ab,kw OR (cardiac infarct*):ti,ab,kw OR (cardiovascular stroke*):ti,ab,kw OR (cardiogenic shock):ti,ab,kw OR (heart attack*):ti,ab,kw OR (Acute Coronary Syndrome):ti,ab,kw OR (ACS):ti,ab,kw OR (Acute Coronary Syndrome*):ti,ab,kw OR (NSTE-ACS):ti,ab,kw OR (Non–ST-Segment Elevation Acute Coronary Syndrome*):ti,ab,kw OR (STEMI):ti,ab,kw OR (NSTEMI):ti,ab,kw OR (ST-segment elevation myocardial infarction):ti,ab,kw OR (Non-ST-segment elevation myocardial infarction):ti,ab,kw OR (MINOCA):ti,ab,kw OR (Myocardial infarction with no obstructive coronary atherosclerosis):ti,ab,kw OR (xiongbi):ti,ab,kw OR (xiongbixintong):ti,ab,kw OR (xiongbi Xintong):ti,ab,kw OR (zhenxintong):ti,ab,kw OR (cuxintong):ti,ab,kw OR (Xintong):ti,ab,kw OR (juexintong):ti,ab,kw OR (cardiovascular shock):ti,ab,kw OR (cardiogenic stroke*):ti,ab,kw

#5 MeSH descriptor: [Percutaneous Coronary Intervention] explode all trees

#6 (percutaneous coronary intervention):ti,ab,kw OR (percutaneous coronary intervention*):ti,ab,kw OR (percutaneous coronary revascularization*):ti,ab,kw OR (coronary atherectom*):ti,ab,kw OR (transluminal coronary angioplast*):ti,ab,kw OR (transluminal coronary balloon dilation):ti,ab,kw OR (coronary balloon angioplast*):ti,ab,kw OR (PCI):ti,ab,kw OR (Percutaneous Transluminal Coronary Angioplast*):ti,ab,kw OR (PTCA):ti,ab,kw OR (Rotational Atherectom*):ti,ab,kw OR (Directional Coronary Atherectom*):ti,ab,kw OR (Balloon dilation):ti,ab,kw OR (Artery Stenting):ti,ab,kw

#7 #2 OR #3

#8 #4 OR #7

#9 #5 OR #6

#10 #8 AND #9

#11 #1 AND #10

7.Danlou tablets

CNKI

(TKA=‘丹蒌片’ + ‘丹蒌’) and ((TKA=‘心肌梗死’ + ‘心梗’ + ‘心肌梗塞’ + ‘急性冠脉综合征’+ ‘ACS’ + ‘STEMI’ + ‘NSTEMI’ + ‘胸痹’ + ‘胸痹心痛’ + ‘真心痛’ + ‘卒心痛’ + ‘心痛’ + ‘猝心痛’ + ‘厥心痛’) and (TKA=‘PCI’ + ‘PTCA’ + ‘经皮冠状动脉介入治疗术’ + ‘经皮冠状动脉介入’ + ‘经皮冠状动脉’ + ‘冠脉介入’ + ‘经皮冠脉’ + ‘血管成形术’ + ‘支架置入术’ + ‘冠状动脉成形术’ + ‘球囊’ + ‘支架’))

WangFang Data

主题:(“丹蒌片” OR “丹蒌”) and ((主题:心肌梗死 OR 心梗 OR 心肌梗塞 OR 急性冠脉综合征 OR ACS OR STEMI OR NSTEMI OR 胸痹 OR 胸痹心痛 OR 真心痛 OR 卒心痛 OR 心痛 OR 猝心痛 OR 厥心痛) and 主题:(PCI OR PTCA OR 经皮冠状动脉介入治疗术 OR 经皮冠状动脉介入 OR 经皮冠状动脉 OR 冠脉介入 OR 经皮冠脉 OR 血管成形术 OR 支架置入术 OR 冠状动脉成形术 OR 球囊 OR 支架))

VIP

(M=(“丹蒌片” OR “丹蒌”) OR R=(“丹蒌片” OR “丹蒌”)) AND ((M=(心肌梗死 OR 心梗 OR 心肌梗塞 OR 急性冠脉综合征 OR STEMI OR NSTEMI OR 胸痹 OR 胸痹心痛 OR 真心痛 OR 卒心痛 OR 心痛 OR 猝心痛 OR 厥心痛) OR R=(心肌梗死 OR 心梗 OR 心肌梗塞 OR ACS OR STEMI OR NSTEMI OR 胸痹 OR 胸痹心痛 OR 真心痛 OR 卒心痛 OR 心痛 OR 猝心痛 OR 厥心痛)) AND (M=(PCI OR PTCA OR 经皮冠状动脉介入治疗术 OR 经皮冠状动脉介入 OR 经皮冠状动脉 OR 冠脉介入 OR 经皮冠脉 OR 血管成形术 OR 支架置入术 OR 冠状动脉成形术 OR 球囊 OR 支架) OR R=(PCI OR PTCA OR 经皮冠状动脉介入治疗术 OR 经皮冠状动脉介入 OR 经皮冠状动脉 OR 冠脉介入 OR 经皮冠脉 OR 血管成形术 OR 支架置入术 OR 冠状动脉成形术 OR 球囊 OR 支架)))

Web of Science

#1 TS=(Danlou tablet* OR Danlou)

#2 TS=(myocardial infarct* OR heart infarct* OR cardiac infarct* OR cardiovascular stroke* OR cardiogenic shock OR heart attack* OR ACS OR STEMI OR NSTEMI OR ST-segment elevation myocardial infarction OR Non-ST-segment elevation myocardial infarction OR MINOCA OR Myocardial infarction with no obstructive coronary atherosclerosis OR xiongbi OR xiongbixintong OR xiongbi Xintong OR zhenxintong OR cuxintong OR Xintong OR juexintong OR cardiovascular shock OR cardiogenic stroke*)

#3 TS=(percutaneous coronary intervention OR percutaneous coronary intervention* OR percutaneous coronary revascularization* OR coronary atherectom* OR transluminal coronary angioplast* OR transluminal coronary balloon dilation OR coronary balloon angioplast* OR PCI OR Percutaneous Transluminal Coronary Angioplast* OR PTCA OR Rotational Atherectom* OR Directional Coronary Atherectom* OR Balloon dilation OR Artery Stenting)

#4 #2 AND #3

#5 #1 AND #4

PubMed

#1 “Danlou tablet*”[Title/Abstract] OR “Danlou”[Title/Abstract]

#2 "myocardial infarction"[MeSH Terms] OR “Acute Coronary Syndrome”[MeSH Terms] OR "myocardial infarct*"[Title/Abstract] OR “Acute Coronary Syndrome”[Title/Abstract] OR “ACS”[Title/Abstract] OR “Acute Coronary Syndrome*”[Title/Abstract]OR “NSTE-ACS”[Title/Abstract] OR “Non–ST-Segment Elevation Acute Coronary Syndrome*”[Title/Abstract] OR "heart infarct*"[Title/Abstract] OR "cardiac infarct*"[Title/Abstract] OR "cardiovascular stroke*"[Title/Abstract] OR "cardiogenic shock"[Title/Abstract] OR “cardiovascular shock” [Title/Abstract] OR “cardiogenic stroke*” [Title/Abstract] OR "heart attack*"[Title/Abstract] OR "STEMI"[Title/Abstract] OR "NSTEMI"[Title/Abstract] OR “ST-segment elevation myocardial infarction”[Title/Abstract] OR “Non-ST-segment elevation myocardial infarction”[Title/Abstract] OR “MINOCA”[Title/Abstract] OR “Myocardial infarction with no obstructive coronary atherosclerosis” [Title/Abstract] OR “xiongbi” [Title/Abstract] OR “xiongbixintong” [Title/Abstract] OR “xiongbi xintong” [Title/Abstract] OR “zhenxintong” [Title/Abstract] OR “cuxintong” [Title/Abstract] OR “Xintong” [Title/Abstract] OR “juexintong” [Title/Abstract]

#3 “percutaneous coronary intervention” [MeSH Terms] OR “percutaneous coronary intervention*”[Title/Abstract] OR “percutaneous coronary revascularization*” [Title/Abstract] OR “coronary atherectom*”[Title/Abstract] OR “transluminal coronary angioplast*”[Title/Abstract] OR “transluminal coronary balloon dilation”[Title/Abstract] OR “coronary balloon angioplast*”[Title/Abstract] OR “PCI”[Title/Abstract] OR “Percutaneous Transluminal Coronary Angioplast*”[Title/Abstract] OR “PTCA”[Title/Abstract] OR “Rotational Atherectom*”[Title/Abstract] OR

“Directional Coronary Atherectom*”[Title/Abstract] OR “Balloon dilation”[Title/Abstract] OR “Artery Stenting”[Title/Abstract]

#4 #2 AND #3

#1 AND #4

Embase

#1 'Danlou tablet*':ab,ti OR ‘Danlou’:ab,ti

#2 'heart infarction'/exp

#3 'Acute Coronary Syndrome'/exp

#4 'cardiac infarct':ab,ti OR 'cardiac infarction':ab,ti OR 'cardial infarct':ab,ti OR 'heart attack':ab,ti OR 'heart infarct':ab,ti OR 'heart micro infarction':ab,ti OR 'heart muscle infarction':ab,ti OR 'infarction, heart':ab,ti OR 'myocardial infarct':ab,ti OR 'myocardial infarction':ab,ti OR 'myocardium infarct':ab,ti OR 'myocardium infarction':ab,ti OR 'premonitory infarction sign':ab,ti OR 'second heart attack':ab,ti OR 'subendocardial infarction':ab,ti OR 'transmural cardiac infarction':ab,ti OR 'transmural heart infarction':ab,ti OR 'transmural infarction, heart':ab,ti OR 'heart infarction':ab,ti OR ‘Acute Coronary Syndrome’:ab,ti OR ‘ACS’:ab,ti OR ‘Acute Coronary Syndrome*’:ab,ti OR ‘NSTE-ACS’:ab,ti OR ‘Non–ST-Segment Elevation Acute Coronary Syndrome*’:ab,ti OR 'STEMI':ab,ti OR 'NSTEMI':ab,ti OR ‘ST-segment elevation myocardial infarction’:ab,ti OR ‘Non-ST-segment elevation myocardial infarction’:ab,ti OR ‘MINOCA’:ab,ti OR ‘Myocardial infarction with no obstructive coronary atherosclerosis’:ab,ti OR ‘xiongbi’:ab,ti OR ‘xiongbixintong’:ab,ti OR ‘xiongbi Xintong’:ab,ti OR ‘zhenxintong’:ab,ti OR ‘cuxintong’:ab,ti OR ‘Xintong’:ab,ti OR ‘juexintong’:ab,ti OR ‘cardiovascular shock’:ab,ti OR ‘cardiogenic stroke*’:ab,ti

#5 ‘percutaneous coronary intervention'/exp

#6 ‘percutaneous coronary intervention*':ab,ti OR 'percutaneous coronary revascularization*':ab,ti OR 'coronary atherectom*':ab,ti OR 'transluminal coronary angioplast*':ab,ti OR 'transluminal coronary balloon dilation':ab,ti OR 'coronary balloon angioplast*':ab,ti OR 'PCI':ab,ti OR 'Percutaneous Transluminal Coronary Angioplast*':ab,ti OR 'PTCA':ab,ti OR 'Rotational Atherectom*':ab,ti OR 'Directional Coronary Atherectom*':ab,ti OR 'Balloon dilation':ab,ti OR 'Artery Stenting’:ab,ti

#7 #2 OR #3

#8 #4 OR #7

#9 #5 OR #6

#10 #8 AND #9

#11 #1 AND #10

Cochrane

#1 (Danlou tablet*):ti,ab,kw OR (Danlou):ti,ab,kw

#2 MeSH descriptor: [Myocardial Infarction] explode all trees

#3 MeSH descriptor: [Acute Coronary Syndrome] explode all trees

#4 (myocardial infarct*):ti,ab,kw OR (heart infarct*):ti,ab,kw OR (cardiac infarct*):ti,ab,kw OR (cardiovascular stroke*):ti,ab,kw OR (cardiogenic shock):ti,ab,kw OR (heart attack*):ti,ab,kw OR (Acute Coronary Syndrome):ti,ab,kw OR (ACS):ti,ab,kw OR (Acute Coronary Syndrome*):ti,ab,kw OR (NSTE-ACS):ti,ab,kw OR (Non–ST-Segment Elevation Acute Coronary Syndrome*):ti,ab,kw OR (STEMI):ti,ab,kw OR (NSTEMI):ti,ab,kw OR (ST-segment elevation myocardial infarction):ti,ab,kw OR (Non-ST-segment elevation myocardial infarction):ti,ab,kw OR (MINOCA):ti,ab,kw OR (Myocardial infarction with no obstructive coronary atherosclerosis):ti,ab,kw OR (xiongbi):ti,ab,kw OR (xiongbixintong):ti,ab,kw OR (xiongbi Xintong):ti,ab,kw OR (zhenxintong):ti,ab,kw OR (cuxintong):ti,ab,kw OR (Xintong):ti,ab,kw OR (juexintong):ti,ab,kw OR (cardiovascular shock):ti,ab,kw OR (cardiogenic stroke*):ti,ab,kw

#5 MeSH descriptor: [Percutaneous Coronary Intervention] explode all trees

#6 (percutaneous coronary intervention):ti,ab,kw OR (percutaneous coronary intervention*):ti,ab,kw OR (percutaneous coronary revascularization*):ti,ab,kw OR (coronary atherectom*):ti,ab,kw OR (transluminal coronary angioplast*):ti,ab,kw OR (transluminal coronary balloon dilation):ti,ab,kw OR (coronary balloon angioplast*):ti,ab,kw OR (PCI):ti,ab,kw OR (Percutaneous Transluminal Coronary Angioplast*):ti,ab,kw OR (PTCA):ti,ab,kw OR (Rotational Atherectom*):ti,ab,kw OR (Directional Coronary Atherectom*):ti,ab,kw OR (Balloon dilation):ti,ab,kw OR (Artery Stenting):ti,ab,kw

#7 #2 OR #3

#8 #4 OR #7

#9 #5 OR #6

#10 #8 AND #9

#11 #1 AND #10

8.Xinyuan capsules

CNKI

(TKA=‘心元胶囊’ OR ‘心元’) and ((TKA=‘心肌梗死’ + ‘心梗’ + ‘心肌梗塞’ + ‘急性冠脉综合征’+ ‘ACS’ + ‘STEMI’ + ‘NSTEMI’ + ‘胸痹’ + ‘胸痹心痛’ + ‘真心痛’ + ‘卒心痛’ + ‘心痛’ + ‘猝心痛’ + ‘厥心痛’) and (TKA=‘PCI’ + ‘PTCA’ + ‘经皮冠状动脉介入治疗术’ + ‘经皮冠状动脉介入’ + ‘经皮冠状动脉’ + ‘冠脉介入’ + ‘经皮冠脉’ + ‘血管成形术’ + ‘支架置入术’ + ‘冠状动脉成形术’ + ‘球囊’ + ‘支架’))

WangFang Data

主题:(“心元胶囊” OR “心元”) and ((主题:心肌梗死 OR 心梗 OR 心肌梗塞 OR 急性冠脉综合征 OR ACS OR STEMI OR NSTEMI OR 胸痹 OR 胸痹心痛 OR 真心痛 OR 卒心痛 OR 心痛 OR 猝心痛 OR 厥心痛) and 主题:(PCI OR PTCA OR 经皮冠状动脉介入治疗术 OR 经皮冠状动脉介入 OR 经皮冠状动脉 OR 冠脉介入 OR 经皮冠脉 OR 血管成形术 OR 支架置入术 OR 冠状动脉成形术 OR 球囊 OR 支架))

VIP

(M=(“心元胶囊” OR “心元”) OR R=(“心元胶囊” OR “心元”)) AND ((M=(心肌梗死 OR 心梗 OR 心肌梗塞 OR 急性冠脉综合征 OR STEMI OR NSTEMI OR 胸痹 OR 胸痹心痛 OR 真心痛 OR 卒心痛 OR 心痛 OR 猝心痛 OR 厥心痛) OR R=(心肌梗死 OR 心梗 OR 心肌梗塞 OR ACS OR STEMI OR NSTEMI OR 胸痹 OR 胸痹心痛 OR 真心痛 OR 卒心痛 OR 心痛 OR 猝心痛 OR 厥心痛)) AND (M=(PCI OR PTCA OR 经皮冠状动脉介入治疗术 OR 经皮冠状动脉介入 OR 经皮冠状动脉 OR 冠脉介入 OR 经皮冠脉 OR 血管成形术 OR 支架置入术 OR 冠状动脉成形术 OR 球囊 OR 支架) OR R=(PCI OR PTCA OR 经皮冠状动脉介入治疗术 OR 经皮冠状动脉介入 OR 经皮冠状动脉 OR 冠脉介入 OR 经皮冠脉 OR 血管成形术 OR 支架置入术 OR 冠状动脉成形术 OR 球囊 OR 支架)))

Web of Science

#1 TS=(Xinyuan capsule* OR Xinyuan)

#2 TS=(myocardial infarct* OR heart infarct* OR cardiac infarct* OR cardiovascular stroke* OR cardiogenic shock OR heart attack* OR ACS OR STEMI OR NSTEMI OR ST-segment elevation myocardial infarction OR Non-ST-segment elevation myocardial infarction OR MINOCA OR Myocardial infarction with no obstructive coronary atherosclerosis OR xiongbi OR xiongbixintong OR xiongbi Xintong OR zhenxintong OR cuxintong OR Xintong OR juexintong OR cardiovascular shock OR cardiogenic stroke*)

#3 TS=(percutaneous coronary intervention OR percutaneous coronary intervention* OR percutaneous coronary revascularization* OR coronary atherectom* OR transluminal coronary angioplast* OR transluminal coronary balloon dilation OR coronary balloon angioplast* OR PCI OR Percutaneous Transluminal Coronary Angioplast* OR PTCA OR Rotational Atherectom* OR Directional Coronary Atherectom* OR Balloon dilation OR Artery Stenting)

#4 #2 AND #3

#5 #1 AND #4

PubMed

#1 “Xinyuan capsule*”[Title/Abstract] OR “Xinyuan”[Title/Abstract]

#2 "myocardial infarction"[MeSH Terms] OR “Acute Coronary Syndrome”[MeSH Terms] OR "myocardial infarct*"[Title/Abstract] OR “Acute Coronary Syndrome”[Title/Abstract] OR “ACS”[Title/Abstract] OR “Acute Coronary Syndrome*”[Title/Abstract]OR “NSTE-ACS”[Title/Abstract] OR “Non–ST-Segment Elevation Acute Coronary Syndrome*”[Title/Abstract] OR "heart infarct*"[Title/Abstract] OR "cardiac infarct*"[Title/Abstract] OR "cardiovascular stroke*"[Title/Abstract] OR "cardiogenic shock"[Title/Abstract] OR “cardiovascular shock” [Title/Abstract] OR “cardiogenic stroke*” [Title/Abstract] OR "heart attack*"[Title/Abstract] OR "STEMI"[Title/Abstract] OR "NSTEMI"[Title/Abstract] OR “ST-segment elevation myocardial infarction”[Title/Abstract] OR “Non-ST-segment elevation myocardial infarction”[Title/Abstract] OR “MINOCA”[Title/Abstract] OR “Myocardial infarction with no obstructive coronary atherosclerosis” [Title/Abstract] OR “xiongbi” [Title/Abstract] OR “xiongbixintong” [Title/Abstract] OR “xiongbi xintong” [Title/Abstract] OR “zhenxintong” [Title/Abstract] OR “cuxintong” [Title/Abstract] OR “Xintong” [Title/Abstract] OR “juexintong” [Title/Abstract]

#3 “percutaneous coronary intervention” [MeSH Terms] OR “percutaneous coronary intervention*”[Title/Abstract] OR “percutaneous coronary revascularization*” [Title/Abstract] OR “coronary atherectom*”[Title/Abstract] OR “transluminal coronary angioplast*”[Title/Abstract] OR “transluminal coronary balloon dilation”[Title/Abstract] OR “coronary balloon angioplast*”[Title/Abstract] OR “PCI”[Title/Abstract] OR “Percutaneous Transluminal Coronary Angioplast*”[Title/Abstract] OR “PTCA”[Title/Abstract] OR “Rotational Atherectom*”[Title/Abstract] OR

“Directional Coronary Atherectom*”[Title/Abstract] OR “Balloon dilation”[Title/Abstract] OR “Artery Stenting”[Title/Abstract]

#4 #2 AND #3

#1 AND #4

Embase

#1 'Xinyuan capsule*':ab,ti OR ‘Xinyuan’:ab,ti

#2 'heart infarction'/exp

#3 'Acute Coronary Syndrome'/exp

#4 'cardiac infarct':ab,ti OR 'cardiac infarction':ab,ti OR 'cardial infarct':ab,ti OR 'heart attack':ab,ti OR 'heart infarct':ab,ti OR 'heart micro infarction':ab,ti OR 'heart muscle infarction':ab,ti OR 'infarction, heart':ab,ti OR 'myocardial infarct':ab,ti OR 'myocardial infarction':ab,ti OR 'myocardium infarct':ab,ti OR 'myocardium infarction':ab,ti OR 'premonitory infarction sign':ab,ti OR 'second heart attack':ab,ti OR 'subendocardial infarction':ab,ti OR 'transmural cardiac infarction':ab,ti OR 'transmural heart infarction':ab,ti OR 'transmural infarction, heart':ab,ti OR 'heart infarction':ab,ti OR ‘Acute Coronary Syndrome’:ab,ti OR ‘ACS’:ab,ti OR ‘Acute Coronary Syndrome*’:ab,ti OR ‘NSTE-ACS’:ab,ti OR ‘Non–ST-Segment Elevation Acute Coronary Syndrome*’:ab,ti OR 'STEMI':ab,ti OR 'NSTEMI':ab,ti OR ‘ST-segment elevation myocardial infarction’:ab,ti OR ‘Non-ST-segment elevation myocardial infarction’:ab,ti OR ‘MINOCA’:ab,ti OR ‘Myocardial infarction with no obstructive coronary atherosclerosis’:ab,ti OR ‘xiongbi’:ab,ti OR ‘xiongbixintong’:ab,ti OR ‘xiongbi Xintong’:ab,ti OR ‘zhenxintong’:ab,ti OR ‘cuxintong’:ab,ti OR ‘Xintong’:ab,ti OR ‘juexintong’:ab,ti OR ‘cardiovascular shock’:ab,ti OR ‘cardiogenic stroke*’:ab,ti

#5 ‘percutaneous coronary intervention'/exp

#6 ‘percutaneous coronary intervention*':ab,ti OR 'percutaneous coronary revascularization*':ab,ti OR 'coronary atherectom*':ab,ti OR 'transluminal coronary angioplast*':ab,ti OR 'transluminal coronary balloon dilation':ab,ti OR 'coronary balloon angioplast*':ab,ti OR 'PCI':ab,ti OR 'Percutaneous Transluminal Coronary Angioplast*':ab,ti OR 'PTCA':ab,ti OR 'Rotational Atherectom*':ab,ti OR 'Directional Coronary Atherectom*':ab,ti OR 'Balloon dilation':ab,ti OR 'Artery Stenting’:ab,ti

#7 #2 OR #3

#8 #4 OR #7

#9 #5 OR #6

#10 #8 AND #9

#11 #1 AND #10

Cochrane

#1 (Xinyuan capsule*):ti,ab,kw OR (Xinyuan):ti,ab,kw

#2 MeSH descriptor: [Myocardial Infarction] explode all trees

#3 MeSH descriptor: [Acute Coronary Syndrome] explode all trees

#4 (myocardial infarct*):ti,ab,kw OR (heart infarct*):ti,ab,kw OR (cardiac infarct*):ti,ab,kw OR (cardiovascular stroke*):ti,ab,kw OR (cardiogenic shock):ti,ab,kw OR (heart attack*):ti,ab,kw OR (Acute Coronary Syndrome):ti,ab,kw OR (ACS):ti,ab,kw OR (Acute Coronary Syndrome*):ti,ab,kw OR (NSTE-ACS):ti,ab,kw OR (Non–ST-Segment Elevation Acute Coronary Syndrome*):ti,ab,kw OR (STEMI):ti,ab,kw OR (NSTEMI):ti,ab,kw OR (ST-segment elevation myocardial infarction):ti,ab,kw OR (Non-ST-segment elevation myocardial infarction):ti,ab,kw OR (MINOCA):ti,ab,kw OR (Myocardial infarction with no obstructive coronary atherosclerosis):ti,ab,kw OR (xiongbi):ti,ab,kw OR (xiongbixintong):ti,ab,kw OR (xiongbi Xintong):ti,ab,kw OR (zhenxintong):ti,ab,kw OR (cuxintong):ti,ab,kw OR (Xintong):ti,ab,kw OR (juexintong):ti,ab,kw OR (cardiovascular shock):ti,ab,kw OR (cardiogenic stroke*):ti,ab,kw

#5 MeSH descriptor: [Percutaneous Coronary Intervention] explode all trees

#6 (percutaneous coronary intervention):ti,ab,kw OR (percutaneous coronary intervention*):ti,ab,kw OR (percutaneous coronary revascularization*):ti,ab,kw OR (coronary atherectom*):ti,ab,kw OR (transluminal coronary angioplast*):ti,ab,kw OR (transluminal coronary balloon dilation):ti,ab,kw OR (coronary balloon angioplast*):ti,ab,kw OR (PCI):ti,ab,kw OR (Percutaneous Transluminal Coronary Angioplast*):ti,ab,kw OR (PTCA):ti,ab,kw OR (Rotational Atherectom*):ti,ab,kw OR (Directional Coronary Atherectom*):ti,ab,kw OR (Balloon dilation):ti,ab,kw OR (Artery Stenting):ti,ab,kw

#7 #2 OR #3

#8 #4 OR #7

#9 #5 OR #6

#10 #8 AND #9

#11 #1 AND #10

9.Xinkeshu tablets

CNKI

(TKA=‘心可舒片’ + ‘心可舒’) and ((TKA=‘心肌梗死’ + ‘心梗’ + ‘心肌梗塞’ + ‘急性冠脉综合征’+ ‘ACS’ + ‘STEMI’ + ‘NSTEMI’ + ‘胸痹’ + ‘胸痹心痛’ + ‘真心痛’ + ‘卒心痛’ + ‘心痛’ + ‘猝心痛’ + ‘厥心痛’) and (TKA=‘PCI’ + ‘PTCA’ + ‘经皮冠状动脉介入治疗术’ + ‘经皮冠状动脉介入’ + ‘经皮冠状动脉’ + ‘冠脉介入’ + ‘经皮冠脉’ + ‘血管成形术’ + ‘支架置入术’ + ‘冠状动脉成形术’ + ‘球囊’ + ‘支架’))

WangFang Data

主题:(“心可舒片” OR “心可舒”) and ((主题:心肌梗死 OR 心梗 OR 心肌梗塞 OR 急性冠脉综合征 OR ACS OR STEMI OR NSTEMI OR 胸痹 OR 胸痹心痛 OR 真心痛 OR 卒心痛 OR 心痛 OR 猝心痛 OR 厥心痛) and 主题:(PCI OR PTCA OR 经皮冠状动脉介入治疗术 OR 经皮冠状动脉介入 OR 经皮冠状动脉 OR 冠脉介入 OR 经皮冠脉 OR 血管成形术 OR 支架置入术 OR 冠状动脉成形术 OR 球囊 OR 支架))

VIP

(M=(“心可舒片” OR “心可舒”) OR R=(“心可舒片” OR “心可舒”)) AND ((M=(心肌梗死 OR 心梗 OR 心肌梗塞 OR 急性冠脉综合征 OR STEMI OR NSTEMI OR 胸痹 OR 胸痹心痛 OR 真心痛 OR 卒心痛 OR 心痛 OR 猝心痛 OR 厥心痛) OR R=(心肌梗死 OR 心梗 OR 心肌梗塞 OR ACS OR STEMI OR NSTEMI OR 胸痹 OR 胸痹心痛 OR 真心痛 OR 卒心痛 OR 心痛 OR 猝心痛 OR 厥心痛)) AND (M=(PCI OR PTCA OR 经皮冠状动脉介入治疗术 OR 经皮冠状动脉介入 OR 经皮冠状动脉 OR 冠脉介入 OR 经皮冠脉 OR 血管成形术 OR 支架置入术 OR 冠状动脉成形术 OR 球囊 OR 支架) OR R=(PCI OR PTCA OR 经皮冠状动脉介入治疗术 OR 经皮冠状动脉介入 OR 经皮冠状动脉 OR 冠脉介入 OR 经皮冠脉 OR 血管成形术 OR 支架置入术 OR 冠状动脉成形术 OR 球囊 OR 支架)))

Web of Science

#1 TS=(Xinkeshu tablet* OR Xinkeshu)

#2 TS=(myocardial infarct* OR heart infarct* OR cardiac infarct* OR cardiovascular stroke* OR cardiogenic shock OR heart attack* OR ACS OR STEMI OR NSTEMI OR ST-segment elevation myocardial infarction OR Non-ST-segment elevation myocardial infarction OR MINOCA OR Myocardial infarction with no obstructive coronary atherosclerosis OR xiongbi OR xiongbixintong OR xiongbi Xintong OR zhenxintong OR cuxintong OR Xintong OR juexintong OR cardiovascular shock OR cardiogenic stroke*)

#3 TS=(percutaneous coronary intervention OR percutaneous coronary intervention* OR percutaneous coronary revascularization* OR coronary atherectom* OR transluminal coronary angioplast* OR transluminal coronary balloon dilation OR coronary balloon angioplast* OR PCI OR Percutaneous Transluminal Coronary Angioplast* OR PTCA OR Rotational Atherectom* OR Directional Coronary Atherectom* OR Balloon dilation OR Artery Stenting)

#4 #2 AND #3

#5 #1 AND #4

PubMed

#1 “Xinkeshu tablet*”[Title/Abstract] OR “Xinkeshu”[Title/Abstract]

#2 "myocardial infarction"[MeSH Terms] OR “Acute Coronary Syndrome”[MeSH Terms] OR "myocardial infarct*"[Title/Abstract] OR “Acute Coronary Syndrome”[Title/Abstract] OR “ACS”[Title/Abstract] OR “Acute Coronary Syndrome*”[Title/Abstract]OR “NSTE-ACS”[Title/Abstract] OR “Non–ST-Segment Elevation Acute Coronary Syndrome*”[Title/Abstract] OR "heart infarct*"[Title/Abstract] OR "cardiac infarct*"[Title/Abstract] OR "cardiovascular stroke*"[Title/Abstract] OR "cardiogenic shock"[Title/Abstract] OR “cardiovascular shock” [Title/Abstract] OR “cardiogenic stroke*” [Title/Abstract] OR "heart attack*"[Title/Abstract] OR "STEMI"[Title/Abstract] OR "NSTEMI"[Title/Abstract] OR “ST-segment elevation myocardial infarction”[Title/Abstract] OR “Non-ST-segment elevation myocardial infarction”[Title/Abstract] OR “MINOCA”[Title/Abstract] OR “Myocardial infarction with no obstructive coronary atherosclerosis” [Title/Abstract] OR “xiongbi” [Title/Abstract] OR “xiongbixintong” [Title/Abstract] OR “xiongbi xintong” [Title/Abstract] OR “zhenxintong” [Title/Abstract] OR “cuxintong” [Title/Abstract] OR “Xintong” [Title/Abstract] OR “juexintong” [Title/Abstract]

#3 “percutaneous coronary intervention” [MeSH Terms] OR “percutaneous coronary intervention*”[Title/Abstract] OR “percutaneous coronary revascularization*” [Title/Abstract] OR “coronary atherectom*”[Title/Abstract] OR “transluminal coronary angioplast*”[Title/Abstract] OR “transluminal coronary balloon dilation”[Title/Abstract] OR “coronary balloon angioplast*”[Title/Abstract] OR “PCI”[Title/Abstract] OR “Percutaneous Transluminal Coronary Angioplast*”[Title/Abstract] OR “PTCA”[Title/Abstract] OR “Rotational Atherectom*”[Title/Abstract] OR

“Directional Coronary Atherectom*”[Title/Abstract] OR “Balloon dilation”[Title/Abstract] OR “Artery Stenting”[Title/Abstract]

#4 #2 AND #3

#1 AND #4

Embase

#1 'Xinkeshu tablet*':ab,ti OR ‘Xinkeshu’:ab,ti

#2 'heart infarction'/exp

#3 'Acute Coronary Syndrome'/exp

#4 'cardiac infarct':ab,ti OR 'cardiac infarction':ab,ti OR 'cardial infarct':ab,ti OR 'heart attack':ab,ti OR 'heart infarct':ab,ti OR 'heart micro infarction':ab,ti OR 'heart muscle infarction':ab,ti OR 'infarction, heart':ab,ti OR 'myocardial infarct':ab,ti OR 'myocardial infarction':ab,ti OR 'myocardium infarct':ab,ti OR 'myocardium infarction':ab,ti OR 'premonitory infarction sign':ab,ti OR 'second heart attack':ab,ti OR 'subendocardial infarction':ab,ti OR 'transmural cardiac infarction':ab,ti OR 'transmural heart infarction':ab,ti OR 'transmural infarction, heart':ab,ti OR 'heart infarction':ab,ti OR ‘Acute Coronary Syndrome’:ab,ti OR ‘ACS’:ab,ti OR ‘Acute Coronary Syndrome*’:ab,ti OR ‘NSTE-ACS’:ab,ti OR ‘Non–ST-Segment Elevation Acute Coronary Syndrome*’:ab,ti OR 'STEMI':ab,ti OR 'NSTEMI':ab,ti OR ‘ST-segment elevation myocardial infarction’:ab,ti OR ‘Non-ST-segment elevation myocardial infarction’:ab,ti OR ‘MINOCA’:ab,ti OR ‘Myocardial infarction with no obstructive coronary atherosclerosis’:ab,ti OR ‘xiongbi’:ab,ti OR ‘xiongbixintong’:ab,ti OR ‘xiongbi Xintong’:ab,ti OR ‘zhenxintong’:ab,ti OR ‘cuxintong’:ab,ti OR ‘Xintong’:ab,ti OR ‘juexintong’:ab,ti OR ‘cardiovascular shock’:ab,ti OR ‘cardiogenic stroke*’:ab,ti

#5 ‘percutaneous coronary intervention'/exp

#6 ‘percutaneous coronary intervention*':ab,ti OR 'percutaneous coronary revascularization*':ab,ti OR 'coronary atherectom*':ab,ti OR 'transluminal coronary angioplast*':ab,ti OR 'transluminal coronary balloon dilation':ab,ti OR 'coronary balloon angioplast*':ab,ti OR 'PCI':ab,ti OR 'Percutaneous Transluminal Coronary Angioplast*':ab,ti OR 'PTCA':ab,ti OR 'Rotational Atherectom*':ab,ti OR 'Directional Coronary Atherectom*':ab,ti OR 'Balloon dilation':ab,ti OR 'Artery Stenting’:ab,ti

#7 #2 OR #3

#8 #4 OR #7

#9 #5 OR #6

#10 #8 AND #9

#11 #1 AND #10

Cochrane

#1 (Xinkeshu tablet*):ti,ab,kw OR (Xinkeshu):ti,ab,kw

#2 MeSH descriptor: [Myocardial Infarction] explode all trees

#3 MeSH descriptor: [Acute Coronary Syndrome] explode all trees

#4 (myocardial infarct*):ti,ab,kw OR (heart infarct*):ti,ab,kw OR (cardiac infarct*):ti,ab,kw OR (cardiovascular stroke*):ti,ab,kw OR (cardiogenic shock):ti,ab,kw OR (heart attack*):ti,ab,kw OR (Acute Coronary Syndrome):ti,ab,kw OR (ACS):ti,ab,kw OR (Acute Coronary Syndrome*):ti,ab,kw OR (NSTE-ACS):ti,ab,kw OR (Non–ST-Segment Elevation Acute Coronary Syndrome*):ti,ab,kw OR (STEMI):ti,ab,kw OR (NSTEMI):ti,ab,kw OR (ST-segment elevation myocardial infarction):ti,ab,kw OR (Non-ST-segment elevation myocardial infarction):ti,ab,kw OR (MINOCA):ti,ab,kw OR (Myocardial infarction with no obstructive coronary atherosclerosis):ti,ab,kw OR (xiongbi):ti,ab,kw OR (xiongbixintong):ti,ab,kw OR (xiongbi Xintong):ti,ab,kw OR (zhenxintong):ti,ab,kw OR (cuxintong):ti,ab,kw OR (Xintong):ti,ab,kw OR (juexintong):ti,ab,kw OR (cardiovascular shock):ti,ab,kw OR (cardiogenic stroke*):ti,ab,kw

#5 MeSH descriptor: [Percutaneous Coronary Intervention] explode all trees

#6 (percutaneous coronary intervention):ti,ab,kw OR (percutaneous coronary intervention*):ti,ab,kw OR (percutaneous coronary revascularization*):ti,ab,kw OR (coronary atherectom*):ti,ab,kw OR (transluminal coronary angioplast*):ti,ab,kw OR (transluminal coronary balloon dilation):ti,ab,kw OR (coronary balloon angioplast*):ti,ab,kw OR (PCI):ti,ab,kw OR (Percutaneous Transluminal Coronary Angioplast*):ti,ab,kw OR (PTCA):ti,ab,kw OR (Rotational Atherectom*):ti,ab,kw OR (Directional Coronary Atherectom*):ti,ab,kw OR (Balloon dilation):ti,ab,kw OR (Artery Stenting):ti,ab,kw

#7 #2 OR #3

#8 #4 OR #7

#9 #5 OR #6

#10 #8 AND #9

#11 #1 AND #10

10.Xinnaoning capsules

CNKI

(TKA=‘心脑宁胶囊’ + ‘心脑宁’) and ((TKA=‘心肌梗死’ + ‘心梗’ + ‘心肌梗塞’ + ‘急性冠脉综合征’+ ‘ACS’ + ‘STEMI’ + ‘NSTEMI’ + ‘胸痹’ + ‘胸痹心痛’ + ‘真心痛’ + ‘卒心痛’ + ‘心痛’ + ‘猝心痛’ + ‘厥心痛’) and (TKA=‘PCI’ + ‘PTCA’ + ‘经皮冠状动脉介入治疗术’ + ‘经皮冠状动脉介入’ + ‘经皮冠状动脉’ + ‘冠脉介入’ + ‘经皮冠脉’ + ‘血管成形术’ + ‘支架置入术’ + ‘冠状动脉成形术’ + ‘球囊’ + ‘支架’))

WangFang Data

主题:(“心脑宁胶囊” OR “心脑宁”) and ((主题:心肌梗死 OR 心梗 OR 心肌梗塞 OR 急性冠脉综合征 OR ACS OR STEMI OR NSTEMI OR 胸痹 OR 胸痹心痛 OR 真心痛 OR 卒心痛 OR 心痛 OR 猝心痛 OR 厥心痛) and 主题:(PCI OR PTCA OR 经皮冠状动脉介入治疗术 OR 经皮冠状动脉介入 OR 经皮冠状动脉 OR 冠脉介入 OR 经皮冠脉 OR 血管成形术 OR 支架置入术 OR 冠状动脉成形术 OR 球囊 OR 支架))

VIP

(M=(“心脑宁胶囊” OR “心脑宁”) OR R=(“心脑宁胶囊” OR “心脑宁”)) AND ((M=(心肌梗死 OR 心梗 OR 心肌梗塞 OR 急性冠脉综合征 OR STEMI OR NSTEMI OR 胸痹 OR 胸痹心痛 OR 真心痛 OR 卒心痛 OR 心痛 OR 猝心痛 OR 厥心痛) OR R=(心肌梗死 OR 心梗 OR 心肌梗塞 OR ACS OR STEMI OR NSTEMI OR 胸痹 OR 胸痹心痛 OR 真心痛 OR 卒心痛 OR 心痛 OR 猝心痛 OR 厥心痛)) AND (M=(PCI OR PTCA OR 经皮冠状动脉介入治疗术 OR 经皮冠状动脉介入 OR 经皮冠状动脉 OR 冠脉介入 OR 经皮冠脉 OR 血管成形术 OR 支架置入术 OR 冠状动脉成形术 OR 球囊 OR 支架) OR R=(PCI OR PTCA OR 经皮冠状动脉介入治疗术 OR 经皮冠状动脉介入 OR 经皮冠状动脉 OR 冠脉介入 OR 经皮冠脉 OR 血管成形术 OR 支架置入术 OR 冠状动脉成形术 OR 球囊 OR 支架)))

Web of Science

#1 TS=(Xinnaoning capsule* OR Xinnaoning)

#2 TS=(myocardial infarct* OR heart infarct* OR cardiac infarct* OR cardiovascular stroke* OR cardiogenic shock OR heart attack* OR ACS OR STEMI OR NSTEMI OR ST-segment elevation myocardial infarction OR Non-ST-segment elevation myocardial infarction OR MINOCA OR Myocardial infarction with no obstructive coronary atherosclerosis OR xiongbi OR xiongbixintong OR xiongbi Xintong OR zhenxintong OR cuxintong OR Xintong OR juexintong OR cardiovascular shock OR cardiogenic stroke*)

#3 TS=(percutaneous coronary intervention OR percutaneous coronary intervention* OR percutaneous coronary revascularization* OR coronary atherectom* OR transluminal coronary angioplast* OR transluminal coronary balloon dilation OR coronary balloon angioplast* OR PCI OR Percutaneous Transluminal Coronary Angioplast* OR PTCA OR Rotational Atherectom* OR Directional Coronary Atherectom* OR Balloon dilation OR Artery Stenting)

#4 #2 AND #3

#5 #1 AND #4

PubMed

#1 “Xinnaoning capsule*”[Title/Abstract] OR “Xinnaoning”[Title/Abstract]

#2 "myocardial infarction"[MeSH Terms] OR “Acute Coronary Syndrome”[MeSH Terms] OR "myocardial infarct*"[Title/Abstract] OR “Acute Coronary Syndrome”[Title/Abstract] OR “ACS”[Title/Abstract] OR “Acute Coronary Syndrome*”[Title/Abstract]OR “NSTE-ACS”[Title/Abstract] OR “Non–ST-Segment Elevation Acute Coronary Syndrome*”[Title/Abstract] OR "heart infarct*"[Title/Abstract] OR "cardiac infarct*"[Title/Abstract] OR "cardiovascular stroke*"[Title/Abstract] OR "cardiogenic shock"[Title/Abstract] OR “cardiovascular shock” [Title/Abstract] OR “cardiogenic stroke*” [Title/Abstract] OR "heart attack*"[Title/Abstract] OR "STEMI"[Title/Abstract] OR "NSTEMI"[Title/Abstract] OR “ST-segment elevation myocardial infarction”[Title/Abstract] OR “Non-ST-segment elevation myocardial infarction”[Title/Abstract] OR “MINOCA”[Title/Abstract] OR “Myocardial infarction with no obstructive coronary atherosclerosis” [Title/Abstract] OR “xiongbi” [Title/Abstract] OR “xiongbixintong” [Title/Abstract] OR “xiongbi xintong” [Title/Abstract] OR “zhenxintong” [Title/Abstract] OR “cuxintong” [Title/Abstract] OR “Xintong” [Title/Abstract] OR “juexintong” [Title/Abstract]

#3 “percutaneous coronary intervention” [MeSH Terms] OR “percutaneous coronary intervention*”[Title/Abstract] OR “percutaneous coronary revascularization*” [Title/Abstract] OR “coronary atherectom*”[Title/Abstract] OR “transluminal coronary angioplast*”[Title/Abstract] OR “transluminal coronary balloon dilation”[Title/Abstract] OR “coronary balloon angioplast*”[Title/Abstract] OR “PCI”[Title/Abstract] OR “Percutaneous Transluminal Coronary Angioplast*”[Title/Abstract] OR “PTCA”[Title/Abstract] OR “Rotational Atherectom*”[Title/Abstract] OR

“Directional Coronary Atherectom*”[Title/Abstract] OR “Balloon dilation”[Title/Abstract] OR “Artery Stenting”[Title/Abstract]

#4 #2 AND #3

#1 AND #4

Embase

#1 'Xinnaoning capsule*':ab,ti OR ‘Xinnaoning’:ab,ti

#2 'heart infarction'/exp

#3 'Acute Coronary Syndrome'/exp

#4 'cardiac infarct':ab,ti OR 'cardiac infarction':ab,ti OR 'cardial infarct':ab,ti OR 'heart attack':ab,ti OR 'heart infarct':ab,ti OR 'heart micro infarction':ab,ti OR 'heart muscle infarction':ab,ti OR 'infarction, heart':ab,ti OR 'myocardial infarct':ab,ti OR 'myocardial infarction':ab,ti OR 'myocardium infarct':ab,ti OR 'myocardium infarction':ab,ti OR 'premonitory infarction sign':ab,ti OR 'second heart attack':ab,ti OR 'subendocardial infarction':ab,ti OR 'transmural cardiac infarction':ab,ti OR 'transmural heart infarction':ab,ti OR 'transmural infarction, heart':ab,ti OR 'heart infarction':ab,ti OR ‘Acute Coronary Syndrome’:ab,ti OR ‘ACS’:ab,ti OR ‘Acute Coronary Syndrome*’:ab,ti OR ‘NSTE-ACS’:ab,ti OR ‘Non–ST-Segment Elevation Acute Coronary Syndrome*’:ab,ti OR 'STEMI':ab,ti OR 'NSTEMI':ab,ti OR ‘ST-segment elevation myocardial infarction’:ab,ti OR ‘Non-ST-segment elevation myocardial infarction’:ab,ti OR ‘MINOCA’:ab,ti OR ‘Myocardial infarction with no obstructive coronary atherosclerosis’:ab,ti OR ‘xiongbi’:ab,ti OR ‘xiongbixintong’:ab,ti OR ‘xiongbi Xintong’:ab,ti OR ‘zhenxintong’:ab,ti OR ‘cuxintong’:ab,ti OR ‘Xintong’:ab,ti OR ‘juexintong’:ab,ti OR ‘cardiovascular shock’:ab,ti OR ‘cardiogenic stroke*’:ab,ti

#5 ‘percutaneous coronary intervention'/exp

#6 ‘percutaneous coronary intervention*':ab,ti OR 'percutaneous coronary revascularization*':ab,ti OR 'coronary atherectom*':ab,ti OR 'transluminal coronary angioplast*':ab,ti OR 'transluminal coronary balloon dilation':ab,ti OR 'coronary balloon angioplast*':ab,ti OR 'PCI':ab,ti OR 'Percutaneous Transluminal Coronary Angioplast*':ab,ti OR 'PTCA':ab,ti OR 'Rotational Atherectom*':ab,ti OR 'Directional Coronary Atherectom*':ab,ti OR 'Balloon dilation':ab,ti OR 'Artery Stenting’:ab,ti

#7 #2 OR #3

#8 #4 OR #7

#9 #5 OR #6

#10 #8 AND #9

#11 #1 AND #10

Cochrane

#1 (Xinnaoning capsule*):ti,ab,kw OR (Xinnaoning):ti,ab,kw

#2 MeSH descriptor: [Myocardial Infarction] explode all trees

#3 MeSH descriptor: [Acute Coronary Syndrome] explode all trees

#4 (myocardial infarct*):ti,ab,kw OR (heart infarct*):ti,ab,kw OR (cardiac infarct*):ti,ab,kw OR (cardiovascular stroke*):ti,ab,kw OR (cardiogenic shock):ti,ab,kw OR (heart attack*):ti,ab,kw OR (Acute Coronary Syndrome):ti,ab,kw OR (ACS):ti,ab,kw OR (Acute Coronary Syndrome*):ti,ab,kw OR (NSTE-ACS):ti,ab,kw OR (Non–ST-Segment Elevation Acute Coronary Syndrome*):ti,ab,kw OR (STEMI):ti,ab,kw OR (NSTEMI):ti,ab,kw OR (ST-segment elevation myocardial infarction):ti,ab,kw OR (Non-ST-segment elevation myocardial infarction):ti,ab,kw OR (MINOCA):ti,ab,kw OR (Myocardial infarction with no obstructive coronary atherosclerosis):ti,ab,kw OR (xiongbi):ti,ab,kw OR (xiongbixintong):ti,ab,kw OR (xiongbi Xintong):ti,ab,kw OR (zhenxintong):ti,ab,kw OR (cuxintong):ti,ab,kw OR (Xintong):ti,ab,kw OR (juexintong):ti,ab,kw OR (cardiovascular shock):ti,ab,kw OR (cardiogenic stroke*):ti,ab,kw

#5 MeSH descriptor: [Percutaneous Coronary Intervention] explode all trees

#6 (percutaneous coronary intervention):ti,ab,kw OR (percutaneous coronary intervention*):ti,ab,kw OR (percutaneous coronary revascularization*):ti,ab,kw OR (coronary atherectom*):ti,ab,kw OR (transluminal coronary angioplast*):ti,ab,kw OR (transluminal coronary balloon dilation):ti,ab,kw OR (coronary balloon angioplast*):ti,ab,kw OR (PCI):ti,ab,kw OR (Percutaneous Transluminal Coronary Angioplast*):ti,ab,kw OR (PTCA):ti,ab,kw OR (Rotational Atherectom*):ti,ab,kw OR (Directional Coronary Atherectom*):ti,ab,kw OR (Balloon dilation):ti,ab,kw OR (Artery Stenting):ti,ab,kw

#7 #2 OR #3

#8 #4 OR #7

#9 #5 OR #6

#10 #8 AND #9

#11 #1 AND #10

11.Xinnaoqing soft capsules

CNKI

(TKA=‘心脑清软胶囊’ + ‘心脑清’) and ((TKA=‘心肌梗死’ + ‘心梗’ + ‘心肌梗塞’ + ‘急性冠脉综合征’+ ‘ACS’ + ‘STEMI’ + ‘NSTEMI’ + ‘胸痹’ + ‘胸痹心痛’ + ‘真心痛’ + ‘卒心痛’ + ‘心痛’ + ‘猝心痛’ + ‘厥心痛’) and (TKA=‘PCI’ + ‘PTCA’ + ‘经皮冠状动脉介入治疗术’ + ‘经皮冠状动脉介入’ + ‘经皮冠状动脉’ + ‘冠脉介入’ + ‘经皮冠脉’ + ‘血管成形术’ + ‘支架置入术’ + ‘冠状动脉成形术’ + ‘球囊’ + ‘支架’))

WangFang Data

主题:(“心脑清软胶囊” OR “心脑清”) and ((主题:心肌梗死 OR 心梗 OR 心肌梗塞 OR 急性冠脉综合征 OR ACS OR STEMI OR NSTEMI OR 胸痹 OR 胸痹心痛 OR 真心痛 OR 卒心痛 OR 心痛 OR 猝心痛 OR 厥心痛) and 主题:(PCI OR PTCA OR 经皮冠状动脉介入治疗术 OR 经皮冠状动脉介入 OR 经皮冠状动脉 OR 冠脉介入 OR 经皮冠脉 OR 血管成形术 OR 支架置入术 OR 冠状动脉成形术 OR 球囊 OR 支架))

VIP

(M=(“心脑清软胶囊” OR “心脑清”) OR R=(“心脑清软胶囊” OR “心脑清”)) AND ((M=(心肌梗死 OR 心梗 OR 心肌梗塞 OR 急性冠脉综合征 OR STEMI OR NSTEMI OR 胸痹 OR 胸痹心痛 OR 真心痛 OR 卒心痛 OR 心痛 OR 猝心痛 OR 厥心痛) OR R=(心肌梗死 OR 心梗 OR 心肌梗塞 OR ACS OR STEMI OR NSTEMI OR 胸痹 OR 胸痹心痛 OR 真心痛 OR 卒心痛 OR 心痛 OR 猝心痛 OR 厥心痛)) AND (M=(PCI OR PTCA OR 经皮冠状动脉介入治疗术 OR 经皮冠状动脉介入 OR 经皮冠状动脉 OR 冠脉介入 OR 经皮冠脉 OR 血管成形术 OR 支架置入术 OR 冠状动脉成形术 OR 球囊 OR 支架) OR R=(PCI OR PTCA OR 经皮冠状动脉介入治疗术 OR 经皮冠状动脉介入 OR 经皮冠状动脉 OR 冠脉介入 OR 经皮冠脉 OR 血管成形术 OR 支架置入术 OR 冠状动脉成形术 OR 球囊 OR 支架)))

Web of Science

#1 TS=(XinNaoQingRuanJiaoNang OR xinnaoqing soft capsule* OR XinNaoQing)

#2 TS=(myocardial infarct* OR heart infarct* OR cardiac infarct* OR cardiovascular stroke* OR cardiogenic shock OR heart attack* OR ACS OR STEMI OR NSTEMI OR ST-segment elevation myocardial infarction OR Non-ST-segment elevation myocardial infarction OR MINOCA OR Myocardial infarction with no obstructive coronary atherosclerosis OR xiongbi OR xiongbixintong OR xiongbi Xintong OR zhenxintong OR cuxintong OR Xintong OR juexintong OR cardiovascular shock OR cardiogenic stroke*)

#3 TS=(percutaneous coronary intervention OR percutaneous coronary intervention* OR percutaneous coronary revascularization* OR coronary atherectom* OR transluminal coronary angioplast* OR transluminal coronary balloon dilation OR coronary balloon angioplast* OR PCI OR Percutaneous Transluminal Coronary Angioplast* OR PTCA OR Rotational Atherectom* OR Directional Coronary Atherectom* OR Balloon dilation OR Artery Stenting)

#4 #2 AND #3

#5 #1 AND #4

PubMed

#1 “XinNaoQingRuanJiaoNang”[Title/Abstract] OR “XinNaoQing”[Title/Abstract] OR “xinnaoqing soft capsule*”[Title/Abstract]

#2 "myocardial infarction"[MeSH Terms] OR “Acute Coronary Syndrome”[MeSH Terms] OR "myocardial infarct*"[Title/Abstract] OR “Acute Coronary Syndrome”[Title/Abstract] OR “ACS”[Title/Abstract] OR “Acute Coronary Syndrome*”[Title/Abstract]OR “NSTE-ACS”[Title/Abstract] OR “Non–ST-Segment Elevation Acute Coronary Syndrome*”[Title/Abstract] OR "heart infarct*"[Title/Abstract] OR "cardiac infarct*"[Title/Abstract] OR "cardiovascular stroke*"[Title/Abstract] OR "cardiogenic shock"[Title/Abstract] OR “cardiovascular shock” [Title/Abstract] OR “cardiogenic stroke*” [Title/Abstract] OR "heart attack*"[Title/Abstract] OR "STEMI"[Title/Abstract] OR "NSTEMI"[Title/Abstract] OR “ST-segment elevation myocardial infarction”[Title/Abstract] OR “Non-ST-segment elevation myocardial infarction”[Title/Abstract] OR “MINOCA”[Title/Abstract] OR “Myocardial infarction with no obstructive coronary atherosclerosis” [Title/Abstract] OR “xiongbi” [Title/Abstract] OR “xiongbixintong” [Title/Abstract] OR “xiongbi xintong” [Title/Abstract] OR “zhenxintong” [Title/Abstract] OR “cuxintong” [Title/Abstract] OR “Xintong” [Title/Abstract] OR “juexintong” [Title/Abstract]

#3 “percutaneous coronary intervention” [MeSH Terms] OR “percutaneous coronary intervention*”[Title/Abstract] OR “percutaneous coronary revascularization*” [Title/Abstract] OR “coronary atherectom*”[Title/Abstract] OR “transluminal coronary angioplast*”[Title/Abstract] OR “transluminal coronary balloon dilation”[Title/Abstract] OR “coronary balloon angioplast*”[Title/Abstract] OR “PCI”[Title/Abstract] OR “Percutaneous Transluminal Coronary Angioplast*”[Title/Abstract] OR “PTCA”[Title/Abstract] OR “Rotational Atherectom*”[Title/Abstract] OR

“Directional Coronary Atherectom*”[Title/Abstract] OR “Balloon dilation”[Title/Abstract] OR “Artery Stenting”[Title/Abstract]

#4 #2 AND #3

#1 AND #4

Embase

#1 'XinNaoQingRuanJiaoNang':ab,ti OR ‘XinNaoQing’:ab,ti OR ‘xinnaoqing soft capsule*’:ab,ti

#2 'heart infarction'/exp

#3 'Acute Coronary Syndrome'/exp

#4 'cardiac infarct':ab,ti OR 'cardiac infarction':ab,ti OR 'cardial infarct':ab,ti OR 'heart attack':ab,ti OR 'heart infarct':ab,ti OR 'heart micro infarction':ab,ti OR 'heart muscle infarction':ab,ti OR 'infarction, heart':ab,ti OR 'myocardial infarct':ab,ti OR 'myocardial infarction':ab,ti OR 'myocardium infarct':ab,ti OR 'myocardium infarction':ab,ti OR 'premonitory infarction sign':ab,ti OR 'second heart attack':ab,ti OR 'subendocardial infarction':ab,ti OR 'transmural cardiac infarction':ab,ti OR 'transmural heart infarction':ab,ti OR 'transmural infarction, heart':ab,ti OR 'heart infarction':ab,ti OR ‘Acute Coronary Syndrome’:ab,ti OR ‘ACS’:ab,ti OR ‘Acute Coronary Syndrome*’:ab,ti OR ‘NSTE-ACS’:ab,ti OR ‘Non–ST-Segment Elevation Acute Coronary Syndrome*’:ab,ti OR 'STEMI':ab,ti OR 'NSTEMI':ab,ti OR ‘ST-segment elevation myocardial infarction’:ab,ti OR ‘Non-ST-segment elevation myocardial infarction’:ab,ti OR ‘MINOCA’:ab,ti OR ‘Myocardial infarction with no obstructive coronary atherosclerosis’:ab,ti OR ‘xiongbi’:ab,ti OR ‘xiongbixintong’:ab,ti OR ‘xiongbi Xintong’:ab,ti OR ‘zhenxintong’:ab,ti OR ‘cuxintong’:ab,ti OR ‘Xintong’:ab,ti OR ‘juexintong’:ab,ti OR ‘cardiovascular shock’:ab,ti OR ‘cardiogenic stroke*’:ab,ti

#5 ‘percutaneous coronary intervention'/exp

#6 ‘percutaneous coronary intervention*':ab,ti OR 'percutaneous coronary revascularization*':ab,ti OR 'coronary atherectom*':ab,ti OR 'transluminal coronary angioplast*':ab,ti OR 'transluminal coronary balloon dilation':ab,ti OR 'coronary balloon angioplast*':ab,ti OR 'PCI':ab,ti OR 'Percutaneous Transluminal Coronary Angioplast*':ab,ti OR 'PTCA':ab,ti OR 'Rotational Atherectom*':ab,ti OR 'Directional Coronary Atherectom*':ab,ti OR 'Balloon dilation':ab,ti OR 'Artery Stenting’:ab,ti

#7 #2 OR #3

#8 #4 OR #7

#9 #5 OR #6

#10 #8 AND #9

#11 #1 AND #10

Cochrane

#1 (XinNaoQingRuanJiaoNang):ti,ab,kw OR (XinNaoQing):ti,ab,kw OR (xinnaoqing soft capsule*):ti,ab,kw

#2 MeSH descriptor: [Myocardial Infarction] explode all trees

#3 MeSH descriptor: [Acute Coronary Syndrome] explode all trees

#4 (myocardial infarct*):ti,ab,kw OR (heart infarct*):ti,ab,kw OR (cardiac infarct*):ti,ab,kw OR (cardiovascular stroke*):ti,ab,kw OR (cardiogenic shock):ti,ab,kw OR (heart attack*):ti,ab,kw OR (Acute Coronary Syndrome):ti,ab,kw OR (ACS):ti,ab,kw OR (Acute Coronary Syndrome*):ti,ab,kw OR (NSTE-ACS):ti,ab,kw OR (Non–ST-Segment Elevation Acute Coronary Syndrome*):ti,ab,kw OR (STEMI):ti,ab,kw OR (NSTEMI):ti,ab,kw OR (ST-segment elevation myocardial infarction):ti,ab,kw OR (Non-ST-segment elevation myocardial infarction):ti,ab,kw OR (MINOCA):ti,ab,kw OR (Myocardial infarction with no obstructive coronary atherosclerosis):ti,ab,kw OR (xiongbi):ti,ab,kw OR (xiongbixintong):ti,ab,kw OR (xiongbi Xintong):ti,ab,kw OR (zhenxintong):ti,ab,kw OR (cuxintong):ti,ab,kw OR (Xintong):ti,ab,kw OR (juexintong):ti,ab,kw OR (cardiovascular shock):ti,ab,kw OR (cardiogenic stroke*):ti,ab,kw

#5 MeSH descriptor: [Percutaneous Coronary Intervention] explode all trees

#6 (percutaneous coronary intervention):ti,ab,kw OR (percutaneous coronary intervention*):ti,ab,kw OR (percutaneous coronary revascularization*):ti,ab,kw OR (coronary atherectom*):ti,ab,kw OR (transluminal coronary angioplast*):ti,ab,kw OR (transluminal coronary balloon dilation):ti,ab,kw OR (coronary balloon angioplast*):ti,ab,kw OR (PCI):ti,ab,kw OR (Percutaneous Transluminal Coronary Angioplast*):ti,ab,kw OR (PTCA):ti,ab,kw OR (Rotational Atherectom*):ti,ab,kw OR (Directional Coronary Atherectom*):ti,ab,kw OR (Balloon dilation):ti,ab,kw OR (Artery Stenting):ti,ab,kw

#7 #2 OR #3

#8 #4 OR #7

#9 #5 OR #6

#10 #8 AND #9

#11 #1 AND #10

12.Xinnao Shutong capsules

CNKI

(TKA=‘心脑舒通胶囊’ + ‘心脑舒通’) and ((TKA=‘心肌梗死’ + ‘心梗’ + ‘心肌梗塞’ + ‘急性冠脉综合征’+ ‘ACS’ + ‘STEMI’ + ‘NSTEMI’ + ‘胸痹’ + ‘胸痹心痛’ + ‘真心痛’ + ‘卒心痛’ + ‘心痛’ + ‘猝心痛’ + ‘厥心痛’) and (TKA=‘PCI’ + ‘PTCA’ + ‘经皮冠状动脉介入治疗术’ + ‘经皮冠状动脉介入’ + ‘经皮冠状动脉’ + ‘冠脉介入’ + ‘经皮冠脉’ + ‘血管成形术’ + ‘支架置入术’ + ‘冠状动脉成形术’ + ‘球囊’ + ‘支架’))

WangFang Data

主题:(“心脑舒通胶囊” OR “心脑舒通”) and ((主题:心肌梗死 OR 心梗 OR 心肌梗塞 OR 急性冠脉综合征 OR ACS OR STEMI OR NSTEMI OR 胸痹 OR 胸痹心痛 OR 真心痛 OR 卒心痛 OR 心痛 OR 猝心痛 OR 厥心痛) and 主题:(PCI OR PTCA OR 经皮冠状动脉介入治疗术 OR 经皮冠状动脉介入 OR 经皮冠状动脉 OR 冠脉介入 OR 经皮冠脉 OR 血管成形术 OR 支架置入术 OR 冠状动脉成形术 OR 球囊 OR 支架))

VIP

(M=(“心脑舒通胶囊” OR “心脑舒通”) OR R=(“心脑舒通胶囊” OR “心脑舒通”)) AND ((M=(心肌梗死 OR 心梗 OR 心肌梗塞 OR 急性冠脉综合征 OR STEMI OR NSTEMI OR 胸痹 OR 胸痹心痛 OR 真心痛 OR 卒心痛 OR 心痛 OR 猝心痛 OR 厥心痛) OR R=(心肌梗死 OR 心梗 OR 心肌梗塞 OR ACS OR STEMI OR NSTEMI OR 胸痹 OR 胸痹心痛 OR 真心痛 OR 卒心痛 OR 心痛 OR 猝心痛 OR 厥心痛)) AND (M=(PCI OR PTCA OR 经皮冠状动脉介入治疗术 OR 经皮冠状动脉介入 OR 经皮冠状动脉 OR 冠脉介入 OR 经皮冠脉 OR 血管成形术 OR 支架置入术 OR 冠状动脉成形术 OR 球囊 OR 支架) OR R=(PCI OR PTCA OR 经皮冠状动脉介入治疗术 OR 经皮冠状动脉介入 OR 经皮冠状动脉 OR 冠脉介入 OR 经皮冠脉 OR 血管成形术 OR 支架置入术 OR 冠状动脉成形术 OR 球囊 OR 支架)))

Web of Science

#1 TS=(Xinnaoshutong capsule* OR Xinnaoshutong OR xinnao shutong)

#2 TS=(myocardial infarct* OR heart infarct* OR cardiac infarct* OR cardiovascular stroke* OR cardiogenic shock OR heart attack* OR ACS OR STEMI OR NSTEMI OR ST-segment elevation myocardial infarction OR Non-ST-segment elevation myocardial infarction OR MINOCA OR Myocardial infarction with no obstructive coronary atherosclerosis OR xiongbi OR xiongbixintong OR xiongbi Xintong OR zhenxintong OR cuxintong OR Xintong OR juexintong OR cardiovascular shock OR cardiogenic stroke*)

#3 TS=(percutaneous coronary intervention OR percutaneous coronary intervention* OR percutaneous coronary revascularization* OR coronary atherectom* OR transluminal coronary angioplast* OR transluminal coronary balloon dilation OR coronary balloon angioplast* OR PCI OR Percutaneous Transluminal Coronary Angioplast* OR PTCA OR Rotational Atherectom* OR Directional Coronary Atherectom* OR Balloon dilation OR Artery Stenting)

#4 #2 AND #3

#5 #1 AND #4

PubMed

#1 “Xinnaoshutong capsule*”[Title/Abstract] OR “Xinnaoshutong”[Title/Abstract] OR “xinnao shutong”[Title/Abstract]

#2 "myocardial infarction"[MeSH Terms] OR “Acute Coronary Syndrome”[MeSH Terms] OR "myocardial infarct*"[Title/Abstract] OR “Acute Coronary Syndrome”[Title/Abstract] OR “ACS”[Title/Abstract] OR “Acute Coronary Syndrome*”[Title/Abstract]OR “NSTE-ACS”[Title/Abstract] OR “Non–ST-Segment Elevation Acute Coronary Syndrome*”[Title/Abstract] OR "heart infarct*"[Title/Abstract] OR "cardiac infarct*"[Title/Abstract] OR "cardiovascular stroke*"[Title/Abstract] OR "cardiogenic shock"[Title/Abstract] OR “cardiovascular shock” [Title/Abstract] OR “cardiogenic stroke*” [Title/Abstract] OR "heart attack*"[Title/Abstract] OR "STEMI"[Title/Abstract] OR "NSTEMI"[Title/Abstract] OR “ST-segment elevation myocardial infarction”[Title/Abstract] OR “Non-ST-segment elevation myocardial infarction”[Title/Abstract] OR “MINOCA”[Title/Abstract] OR “Myocardial infarction with no obstructive coronary atherosclerosis” [Title/Abstract] OR “xiongbi” [Title/Abstract] OR “xiongbixintong” [Title/Abstract] OR “xiongbi xintong” [Title/Abstract] OR “zhenxintong” [Title/Abstract] OR “cuxintong” [Title/Abstract] OR “Xintong” [Title/Abstract] OR “juexintong” [Title/Abstract]

#3 “percutaneous coronary intervention” [MeSH Terms] OR “percutaneous coronary intervention*”[Title/Abstract] OR “percutaneous coronary revascularization*” [Title/Abstract] OR “coronary atherectom*”[Title/Abstract] OR “transluminal coronary angioplast*”[Title/Abstract] OR “transluminal coronary balloon dilation”[Title/Abstract] OR “coronary balloon angioplast*”[Title/Abstract] OR “PCI”[Title/Abstract] OR “Percutaneous Transluminal Coronary Angioplast*”[Title/Abstract] OR “PTCA”[Title/Abstract] OR “Rotational Atherectom*”[Title/Abstract] OR

“Directional Coronary Atherectom*”[Title/Abstract] OR “Balloon dilation”[Title/Abstract] OR “Artery Stenting”[Title/Abstract]

#4 #2 AND #3

#1 AND #4

Embase

#1 'Xinnaoshutong capsule*':ab,ti OR ‘Xinnaoshutong’:ab,ti OR ‘xinnao shutong’:ab,ti

#2 'heart infarction'/exp

#3 'Acute Coronary Syndrome'/exp

#4 'cardiac infarct':ab,ti OR 'cardiac infarction':ab,ti OR 'cardial infarct':ab,ti OR 'heart attack':ab,ti OR 'heart infarct':ab,ti OR 'heart micro infarction':ab,ti OR 'heart muscle infarction':ab,ti OR 'infarction, heart':ab,ti OR 'myocardial infarct':ab,ti OR 'myocardial infarction':ab,ti OR 'myocardium infarct':ab,ti OR 'myocardium infarction':ab,ti OR 'premonitory infarction sign':ab,ti OR 'second heart attack':ab,ti OR 'subendocardial infarction':ab,ti OR 'transmural cardiac infarction':ab,ti OR 'transmural heart infarction':ab,ti OR 'transmural infarction, heart':ab,ti OR 'heart infarction':ab,ti OR ‘Acute Coronary Syndrome’:ab,ti OR ‘ACS’:ab,ti OR ‘Acute Coronary Syndrome*’:ab,ti OR ‘NSTE-ACS’:ab,ti OR ‘Non–ST-Segment Elevation Acute Coronary Syndrome*’:ab,ti OR 'STEMI':ab,ti OR 'NSTEMI':ab,ti OR ‘ST-segment elevation myocardial infarction’:ab,ti OR ‘Non-ST-segment elevation myocardial infarction’:ab,ti OR ‘MINOCA’:ab,ti OR ‘Myocardial infarction with no obstructive coronary atherosclerosis’:ab,ti OR ‘xiongbi’:ab,ti OR ‘xiongbixintong’:ab,ti OR ‘xiongbi Xintong’:ab,ti OR ‘zhenxintong’:ab,ti OR ‘cuxintong’:ab,ti OR ‘Xintong’:ab,ti OR ‘juexintong’:ab,ti OR ‘cardiovascular shock’:ab,ti OR ‘cardiogenic stroke*’:ab,ti

#5 ‘percutaneous coronary intervention'/exp

#6 ‘percutaneous coronary intervention*':ab,ti OR 'percutaneous coronary revascularization*':ab,ti OR 'coronary atherectom*':ab,ti OR 'transluminal coronary angioplast*':ab,ti OR 'transluminal coronary balloon dilation':ab,ti OR 'coronary balloon angioplast*':ab,ti OR 'PCI':ab,ti OR 'Percutaneous Transluminal Coronary Angioplast*':ab,ti OR 'PTCA':ab,ti OR 'Rotational Atherectom*':ab,ti OR 'Directional Coronary Atherectom*':ab,ti OR 'Balloon dilation':ab,ti OR 'Artery Stenting’:ab,ti

#7 #2 OR #3

#8 #4 OR #7

#9 #5 OR #6

#10 #8 AND #9

#11 #1 AND #10

Cochrane

#1 (Xinnaoshutong capsule*):ti,ab,kw OR (Xinnaoshutong):ti,ab,kw OR (xinnao shutong):ti,ab,kw

#2 MeSH descriptor: [Myocardial Infarction] explode all trees

#3 MeSH descriptor: [Acute Coronary Syndrome] explode all trees

#4 (myocardial infarct*):ti,ab,kw OR (heart infarct*):ti,ab,kw OR (cardiac infarct*):ti,ab,kw OR (cardiovascular stroke*):ti,ab,kw OR (cardiogenic shock):ti,ab,kw OR (heart attack*):ti,ab,kw OR (Acute Coronary Syndrome):ti,ab,kw OR (ACS):ti,ab,kw OR (Acute Coronary Syndrome*):ti,ab,kw OR (NSTE-ACS):ti,ab,kw OR (Non–ST-Segment Elevation Acute Coronary Syndrome*):ti,ab,kw OR (STEMI):ti,ab,kw OR (NSTEMI):ti,ab,kw OR (ST-segment elevation myocardial infarction):ti,ab,kw OR (Non-ST-segment elevation myocardial infarction):ti,ab,kw OR (MINOCA):ti,ab,kw OR (Myocardial infarction with no obstructive coronary atherosclerosis):ti,ab,kw OR (xiongbi):ti,ab,kw OR (xiongbixintong):ti,ab,kw OR (xiongbi Xintong):ti,ab,kw OR (zhenxintong):ti,ab,kw OR (cuxintong):ti,ab,kw OR (Xintong):ti,ab,kw OR (juexintong):ti,ab,kw OR (cardiovascular shock):ti,ab,kw OR (cardiogenic stroke*):ti,ab,kw

#5 MeSH descriptor: [Percutaneous Coronary Intervention] explode all trees

#6 (percutaneous coronary intervention):ti,ab,kw OR (percutaneous coronary intervention*):ti,ab,kw OR (percutaneous coronary revascularization*):ti,ab,kw OR (coronary atherectom*):ti,ab,kw OR (transluminal coronary angioplast*):ti,ab,kw OR (transluminal coronary balloon dilation):ti,ab,kw OR (coronary balloon angioplast*):ti,ab,kw OR (PCI):ti,ab,kw OR (Percutaneous Transluminal Coronary Angioplast*):ti,ab,kw OR (PTCA):ti,ab,kw OR (Rotational Atherectom*):ti,ab,kw OR (Directional Coronary Atherectom*):ti,ab,kw OR (Balloon dilation):ti,ab,kw OR (Artery Stenting):ti,ab,kw

#7 #2 OR #3

#8 #4 OR #7

#9 #5 OR #6

#10 #8 AND #9

#11 #1 AND #10

13.Xinshu capsules

CNKI

(TKA=‘心舒胶囊’ + ‘心舒’) and ((TKA=‘心肌梗死’ + ‘心梗’ + ‘心肌梗塞’ + ‘急性冠脉综合征’+ ‘ACS’ + ‘STEMI’ + ‘NSTEMI’ + ‘胸痹’ + ‘胸痹心痛’ + ‘真心痛’ + ‘卒心痛’ + ‘心痛’ + ‘猝心痛’ + ‘厥心痛’) and (TKA=‘PCI’ + ‘PTCA’ + ‘经皮冠状动脉介入治疗术’ + ‘经皮冠状动脉介入’ + ‘经皮冠状动脉’ + ‘冠脉介入’ + ‘经皮冠脉’ + ‘血管成形术’ + ‘支架置入术’ + ‘冠状动脉成形术’ + ‘球囊’ + ‘支架’))

WangFang Data

主题:(“心舒胶囊” OR “心舒”) and ((主题:心肌梗死 OR 心梗 OR 心肌梗塞 OR 急性冠脉综合征 OR ACS OR STEMI OR NSTEMI OR 胸痹 OR 胸痹心痛 OR 真心痛 OR 卒心痛 OR 心痛 OR 猝心痛 OR 厥心痛) and 主题:(PCI OR PTCA OR 经皮冠状动脉介入治疗术 OR 经皮冠状动脉介入 OR 经皮冠状动脉 OR 冠脉介入 OR 经皮冠脉 OR 血管成形术 OR 支架置入术 OR 冠状动脉成形术 OR 球囊 OR 支架))

VIP

(M=(“心舒胶囊” OR “心舒”) OR R=(“心舒胶囊” OR “心舒”)) AND ((M=(心肌梗死 OR 心梗 OR 心肌梗塞 OR 急性冠脉综合征 OR STEMI OR NSTEMI OR 胸痹 OR 胸痹心痛 OR 真心痛 OR 卒心痛 OR 心痛 OR 猝心痛 OR 厥心痛) OR R=(心肌梗死 OR 心梗 OR 心肌梗塞 OR ACS OR STEMI OR NSTEMI OR 胸痹 OR 胸痹心痛 OR 真心痛 OR 卒心痛 OR 心痛 OR 猝心痛 OR 厥心痛)) AND (M=(PCI OR PTCA OR 经皮冠状动脉介入治疗术 OR 经皮冠状动脉介入 OR 经皮冠状动脉 OR 冠脉介入 OR 经皮冠脉 OR 血管成形术 OR 支架置入术 OR 冠状动脉成形术 OR 球囊 OR 支架) OR R=(PCI OR PTCA OR 经皮冠状动脉介入治疗术 OR 经皮冠状动脉介入 OR 经皮冠状动脉 OR 冠脉介入 OR 经皮冠脉 OR 血管成形术 OR 支架置入术 OR 冠状动脉成形术 OR 球囊 OR 支架)))

Web of Science

#1 TS=(Xinshu capsule* OR Xinshu)

#2 TS=(myocardial infarct* OR heart infarct* OR cardiac infarct* OR cardiovascular stroke* OR cardiogenic shock OR heart attack* OR ACS OR STEMI OR NSTEMI OR ST-segment elevation myocardial infarction OR Non-ST-segment elevation myocardial infarction OR MINOCA OR Myocardial infarction with no obstructive coronary atherosclerosis OR xiongbi OR xiongbixintong OR xiongbi Xintong OR zhenxintong OR cuxintong OR Xintong OR juexintong OR cardiovascular shock OR cardiogenic stroke*)

#3 TS=(percutaneous coronary intervention OR percutaneous coronary intervention* OR percutaneous coronary revascularization* OR coronary atherectom* OR transluminal coronary angioplast* OR transluminal coronary balloon dilation OR coronary balloon angioplast* OR PCI OR Percutaneous Transluminal Coronary Angioplast* OR PTCA OR Rotational Atherectom* OR Directional Coronary Atherectom* OR Balloon dilation OR Artery Stenting)

#4 #2 AND #3

#5 #1 AND #4

PubMed

#1 “Xinshu capsule*”[Title/Abstract] OR “Xinshu”[Title/Abstract]

#2 "myocardial infarction"[MeSH Terms] OR “Acute Coronary Syndrome”[MeSH Terms] OR "myocardial infarct*"[Title/Abstract] OR “Acute Coronary Syndrome”[Title/Abstract] OR “ACS”[Title/Abstract] OR “Acute Coronary Syndrome*”[Title/Abstract]OR “NSTE-ACS”[Title/Abstract] OR “Non–ST-Segment Elevation Acute Coronary Syndrome*”[Title/Abstract] OR "heart infarct*"[Title/Abstract] OR "cardiac infarct*"[Title/Abstract] OR "cardiovascular stroke*"[Title/Abstract] OR "cardiogenic shock"[Title/Abstract] OR “cardiovascular shock” [Title/Abstract] OR “cardiogenic stroke*” [Title/Abstract] OR "heart attack*"[Title/Abstract] OR "STEMI"[Title/Abstract] OR "NSTEMI"[Title/Abstract] OR “ST-segment elevation myocardial infarction”[Title/Abstract] OR “Non-ST-segment elevation myocardial infarction”[Title/Abstract] OR “MINOCA”[Title/Abstract] OR “Myocardial infarction with no obstructive coronary atherosclerosis” [Title/Abstract] OR “xiongbi” [Title/Abstract] OR “xiongbixintong” [Title/Abstract] OR “xiongbi xintong” [Title/Abstract] OR “zhenxintong” [Title/Abstract] OR “cuxintong” [Title/Abstract] OR “Xintong” [Title/Abstract] OR “juexintong” [Title/Abstract]

#3 “percutaneous coronary intervention” [MeSH Terms] OR “percutaneous coronary intervention*”[Title/Abstract] OR “percutaneous coronary revascularization*” [Title/Abstract] OR “coronary atherectom*”[Title/Abstract] OR “transluminal coronary angioplast*”[Title/Abstract] OR “transluminal coronary balloon dilation”[Title/Abstract] OR “coronary balloon angioplast*”[Title/Abstract] OR “PCI”[Title/Abstract] OR “Percutaneous Transluminal Coronary Angioplast*”[Title/Abstract] OR “PTCA”[Title/Abstract] OR “Rotational Atherectom*”[Title/Abstract] OR

“Directional Coronary Atherectom*”[Title/Abstract] OR “Balloon dilation”[Title/Abstract] OR “Artery Stenting”[Title/Abstract]

#4 #2 AND #3

#1 AND #4

Embase

#1 'Xinshu capsule*':ab,ti OR ‘Xinshu’:ab,ti

#2 'heart infarction'/exp

#3 'Acute Coronary Syndrome'/exp

#4 'cardiac infarct':ab,ti OR 'cardiac infarction':ab,ti OR 'cardial infarct':ab,ti OR 'heart attack':ab,ti OR 'heart infarct':ab,ti OR 'heart micro infarction':ab,ti OR 'heart muscle infarction':ab,ti OR 'infarction, heart':ab,ti OR 'myocardial infarct':ab,ti OR 'myocardial infarction':ab,ti OR 'myocardium infarct':ab,ti OR 'myocardium infarction':ab,ti OR 'premonitory infarction sign':ab,ti OR 'second heart attack':ab,ti OR 'subendocardial infarction':ab,ti OR 'transmural cardiac infarction':ab,ti OR 'transmural heart infarction':ab,ti OR 'transmural infarction, heart':ab,ti OR 'heart infarction':ab,ti OR ‘Acute Coronary Syndrome’:ab,ti OR ‘ACS’:ab,ti OR ‘Acute Coronary Syndrome*’:ab,ti OR ‘NSTE-ACS’:ab,ti OR ‘Non–ST-Segment Elevation Acute Coronary Syndrome*’:ab,ti OR 'STEMI':ab,ti OR 'NSTEMI':ab,ti OR ‘ST-segment elevation myocardial infarction’:ab,ti OR ‘Non-ST-segment elevation myocardial infarction’:ab,ti OR ‘MINOCA’:ab,ti OR ‘Myocardial infarction with no obstructive coronary atherosclerosis’:ab,ti OR ‘xiongbi’:ab,ti OR ‘xiongbixintong’:ab,ti OR ‘xiongbi Xintong’:ab,ti OR ‘zhenxintong’:ab,ti OR ‘cuxintong’:ab,ti OR ‘Xintong’:ab,ti OR ‘juexintong’:ab,ti OR ‘cardiovascular shock’:ab,ti OR ‘cardiogenic stroke*’:ab,ti

#5 ‘percutaneous coronary intervention'/exp

#6 ‘percutaneous coronary intervention*':ab,ti OR 'percutaneous coronary revascularization*':ab,ti OR 'coronary atherectom*':ab,ti OR 'transluminal coronary angioplast*':ab,ti OR 'transluminal coronary balloon dilation':ab,ti OR 'coronary balloon angioplast*':ab,ti OR 'PCI':ab,ti OR 'Percutaneous Transluminal Coronary Angioplast*':ab,ti OR 'PTCA':ab,ti OR 'Rotational Atherectom*':ab,ti OR 'Directional Coronary Atherectom*':ab,ti OR 'Balloon dilation':ab,ti OR 'Artery Stenting’:ab,ti

#7 #2 OR #3

#8 #4 OR #7

#9 #5 OR #6

#10 #8 AND #9

#11 #1 AND #10

Cochrane

#1 (Xinshu capsule*):ti,ab,kw OR (Xinshu):ti,ab,kw

#2 MeSH descriptor: [Myocardial Infarction] explode all trees

#3 MeSH descriptor: [Acute Coronary Syndrome] explode all trees

#4 (myocardial infarct*):ti,ab,kw OR (heart infarct*):ti,ab,kw OR (cardiac infarct*):ti,ab,kw OR (cardiovascular stroke*):ti,ab,kw OR (cardiogenic shock):ti,ab,kw OR (heart attack*):ti,ab,kw OR (Acute Coronary Syndrome):ti,ab,kw OR (ACS):ti,ab,kw OR (Acute Coronary Syndrome*):ti,ab,kw OR (NSTE-ACS):ti,ab,kw OR (Non–ST-Segment Elevation Acute Coronary Syndrome*):ti,ab,kw OR (STEMI):ti,ab,kw OR (NSTEMI):ti,ab,kw OR (ST-segment elevation myocardial infarction):ti,ab,kw OR (Non-ST-segment elevation myocardial infarction):ti,ab,kw OR (MINOCA):ti,ab,kw OR (Myocardial infarction with no obstructive coronary atherosclerosis):ti,ab,kw OR (xiongbi):ti,ab,kw OR (xiongbixintong):ti,ab,kw OR (xiongbi Xintong):ti,ab,kw OR (zhenxintong):ti,ab,kw OR (cuxintong):ti,ab,kw OR (Xintong):ti,ab,kw OR (juexintong):ti,ab,kw OR (cardiovascular shock):ti,ab,kw OR (cardiogenic stroke*):ti,ab,kw

#5 MeSH descriptor: [Percutaneous Coronary Intervention] explode all trees

#6 (percutaneous coronary intervention):ti,ab,kw OR (percutaneous coronary intervention*):ti,ab,kw OR (percutaneous coronary revascularization*):ti,ab,kw OR (coronary atherectom*):ti,ab,kw OR (transluminal coronary angioplast*):ti,ab,kw OR (transluminal coronary balloon dilation):ti,ab,kw OR (coronary balloon angioplast*):ti,ab,kw OR (PCI):ti,ab,kw OR (Percutaneous Transluminal Coronary Angioplast*):ti,ab,kw OR (PTCA):ti,ab,kw OR (Rotational Atherectom*):ti,ab,kw OR (Directional Coronary Atherectom*):ti,ab,kw OR (Balloon dilation):ti,ab,kw OR (Artery Stenting):ti,ab,kw

#7 #2 OR #3

#8 #4 OR #7

#9 #5 OR #6

#10 #8 AND #9

#11 #1 AND #10

14.Kedaling tablets

CNKI

(TKA=‘可达灵片’ + ‘可达灵’) and ((TKA=‘心肌梗死’ + ‘心梗’ + ‘心肌梗塞’ + ‘急性冠脉综合征’+ ‘ACS’ + ‘STEMI’ + ‘NSTEMI’ + ‘胸痹’ + ‘胸痹心痛’ + ‘真心痛’ + ‘卒心痛’ + ‘心痛’ + ‘猝心痛’ + ‘厥心痛’) and (TKA=‘PCI’ + ‘PTCA’ + ‘经皮冠状动脉介入治疗术’ + ‘经皮冠状动脉介入’ + ‘经皮冠状动脉’ + ‘冠脉介入’ + ‘经皮冠脉’ + ‘血管成形术’ + ‘支架置入术’ + ‘冠状动脉成形术’ + ‘球囊’ + ‘支架’))

WangFang Data

主题:(“可达灵片” OR “可达灵”) and ((主题:心肌梗死 OR 心梗 OR 心肌梗塞 OR 急性冠脉综合征 OR ACS OR STEMI OR NSTEMI OR 胸痹 OR 胸痹心痛 OR 真心痛 OR 卒心痛 OR 心痛 OR 猝心痛 OR 厥心痛) and 主题:(PCI OR PTCA OR 经皮冠状动脉介入治疗术 OR 经皮冠状动脉介入 OR 经皮冠状动脉 OR 冠脉介入 OR 经皮冠脉 OR 血管成形术 OR 支架置入术 OR 冠状动脉成形术 OR 球囊 OR 支架))

VIP

(M=(“可达灵片” OR “可达灵”) OR R=(“可达灵片” OR “可达灵”)) AND ((M=(心肌梗死 OR 心梗 OR 心肌梗塞 OR 急性冠脉综合征 OR STEMI OR NSTEMI OR 胸痹 OR 胸痹心痛 OR 真心痛 OR 卒心痛 OR 心痛 OR 猝心痛 OR 厥心痛) OR R=(心肌梗死 OR 心梗 OR 心肌梗塞 OR ACS OR STEMI OR NSTEMI OR 胸痹 OR 胸痹心痛 OR 真心痛 OR 卒心痛 OR 心痛 OR 猝心痛 OR 厥心痛)) AND (M=(PCI OR PTCA OR 经皮冠状动脉介入治疗术 OR 经皮冠状动脉介入 OR 经皮冠状动脉 OR 冠脉介入 OR 经皮冠脉 OR 血管成形术 OR 支架置入术 OR 冠状动脉成形术 OR 球囊 OR 支架) OR R=(PCI OR PTCA OR 经皮冠状动脉介入治疗术 OR 经皮冠状动脉介入 OR 经皮冠状动脉 OR 冠脉介入 OR 经皮冠脉 OR 血管成形术 OR 支架置入术 OR 冠状动脉成形术 OR 球囊 OR 支架)))

Web of Science

#1 TS=(Kedaling tablet* OR Kedaling)

#2 TS=(myocardial infarct* OR heart infarct* OR cardiac infarct* OR cardiovascular stroke* OR cardiogenic shock OR heart attack* OR ACS OR STEMI OR NSTEMI OR ST-segment elevation myocardial infarction OR Non-ST-segment elevation myocardial infarction OR MINOCA OR Myocardial infarction with no obstructive coronary atherosclerosis OR xiongbi OR xiongbixintong OR xiongbi Xintong OR zhenxintong OR cuxintong OR Xintong OR juexintong OR cardiovascular shock OR cardiogenic stroke*)

#3 TS=(percutaneous coronary intervention OR percutaneous coronary intervention* OR percutaneous coronary revascularization* OR coronary atherectom* OR transluminal coronary angioplast* OR transluminal coronary balloon dilation OR coronary balloon angioplast* OR PCI OR Percutaneous Transluminal Coronary Angioplast* OR PTCA OR Rotational Atherectom* OR Directional Coronary Atherectom* OR Balloon dilation OR Artery Stenting)

#4 #2 AND #3

#5 #1 AND #4

PubMed

#1 “Kedaling tablet*”[Title/Abstract] OR “Kedaling”[Title/Abstract]

#2 "myocardial infarction"[MeSH Terms] OR “Acute Coronary Syndrome”[MeSH Terms] OR "myocardial infarct*"[Title/Abstract] OR “Acute Coronary Syndrome”[Title/Abstract] OR “ACS”[Title/Abstract] OR “Acute Coronary Syndrome*”[Title/Abstract]OR “NSTE-ACS”[Title/Abstract] OR “Non–ST-Segment Elevation Acute Coronary Syndrome*”[Title/Abstract] OR "heart infarct*"[Title/Abstract] OR "cardiac infarct*"[Title/Abstract] OR "cardiovascular stroke*"[Title/Abstract] OR "cardiogenic shock"[Title/Abstract] OR “cardiovascular shock” [Title/Abstract] OR “cardiogenic stroke*” [Title/Abstract] OR "heart attack*"[Title/Abstract] OR "STEMI"[Title/Abstract] OR "NSTEMI"[Title/Abstract] OR “ST-segment elevation myocardial infarction”[Title/Abstract] OR “Non-ST-segment elevation myocardial infarction”[Title/Abstract] OR “MINOCA”[Title/Abstract] OR “Myocardial infarction with no obstructive coronary atherosclerosis” [Title/Abstract] OR “xiongbi” [Title/Abstract] OR “xiongbixintong” [Title/Abstract] OR “xiongbi xintong” [Title/Abstract] OR “zhenxintong” [Title/Abstract] OR “cuxintong” [Title/Abstract] OR “Xintong” [Title/Abstract] OR “juexintong” [Title/Abstract]

#3 “percutaneous coronary intervention” [MeSH Terms] OR “percutaneous coronary intervention*”[Title/Abstract] OR “percutaneous coronary revascularization*” [Title/Abstract] OR “coronary atherectom*”[Title/Abstract] OR “transluminal coronary angioplast*”[Title/Abstract] OR “transluminal coronary balloon dilation”[Title/Abstract] OR “coronary balloon angioplast*”[Title/Abstract] OR “PCI”[Title/Abstract] OR “Percutaneous Transluminal Coronary Angioplast*”[Title/Abstract] OR “PTCA”[Title/Abstract] OR “Rotational Atherectom*”[Title/Abstract] OR

“Directional Coronary Atherectom*”[Title/Abstract] OR “Balloon dilation”[Title/Abstract] OR “Artery Stenting”[Title/Abstract]

#4 #2 AND #3

#1 AND #4

Embase

#1 'Kedaling tablet*':ab,ti OR ‘Kedaling’:ab,ti

#2 'heart infarction'/exp

#3 'Acute Coronary Syndrome'/exp

#4 'cardiac infarct':ab,ti OR 'cardiac infarction':ab,ti OR 'cardial infarct':ab,ti OR 'heart attack':ab,ti OR 'heart infarct':ab,ti OR 'heart micro infarction':ab,ti OR 'heart muscle infarction':ab,ti OR 'infarction, heart':ab,ti OR 'myocardial infarct':ab,ti OR 'myocardial infarction':ab,ti OR 'myocardium infarct':ab,ti OR 'myocardium infarction':ab,ti OR 'premonitory infarction sign':ab,ti OR 'second heart attack':ab,ti OR 'subendocardial infarction':ab,ti OR 'transmural cardiac infarction':ab,ti OR 'transmural heart infarction':ab,ti OR 'transmural infarction, heart':ab,ti OR 'heart infarction':ab,ti OR ‘Acute Coronary Syndrome’:ab,ti OR ‘ACS’:ab,ti OR ‘Acute Coronary Syndrome*’:ab,ti OR ‘NSTE-ACS’:ab,ti OR ‘Non–ST-Segment Elevation Acute Coronary Syndrome*’:ab,ti OR 'STEMI':ab,ti OR 'NSTEMI':ab,ti OR ‘ST-segment elevation myocardial infarction’:ab,ti OR ‘Non-ST-segment elevation myocardial infarction’:ab,ti OR ‘MINOCA’:ab,ti OR ‘Myocardial infarction with no obstructive coronary atherosclerosis’:ab,ti OR ‘xiongbi’:ab,ti OR ‘xiongbixintong’:ab,ti OR ‘xiongbi Xintong’:ab,ti OR ‘zhenxintong’:ab,ti OR ‘cuxintong’:ab,ti OR ‘Xintong’:ab,ti OR ‘juexintong’:ab,ti OR ‘cardiovascular shock’:ab,ti OR ‘cardiogenic stroke*’:ab,ti

#5 ‘percutaneous coronary intervention'/exp

#6 ‘percutaneous coronary intervention*':ab,ti OR 'percutaneous coronary revascularization*':ab,ti OR 'coronary atherectom*':ab,ti OR 'transluminal coronary angioplast*':ab,ti OR 'transluminal coronary balloon dilation':ab,ti OR 'coronary balloon angioplast*':ab,ti OR 'PCI':ab,ti OR 'Percutaneous Transluminal Coronary Angioplast*':ab,ti OR 'PTCA':ab,ti OR 'Rotational Atherectom*':ab,ti OR 'Directional Coronary Atherectom*':ab,ti OR 'Balloon dilation':ab,ti OR 'Artery Stenting’:ab,ti

#7 #2 OR #3

#8 #4 OR #7

#9 #5 OR #6

#10 #8 AND #9

#11 #1 AND #10

Cochrane

#1 (Kedaling tablet*):ti,ab,kw OR (Kedaling):ti,ab,kw

#2 MeSH descriptor: [Myocardial Infarction] explode all trees

#3 MeSH descriptor: [Acute Coronary Syndrome] explode all trees

#4 (myocardial infarct*):ti,ab,kw OR (heart infarct*):ti,ab,kw OR (cardiac infarct*):ti,ab,kw OR (cardiovascular stroke*):ti,ab,kw OR (cardiogenic shock):ti,ab,kw OR (heart attack*):ti,ab,kw OR (Acute Coronary Syndrome):ti,ab,kw OR (ACS):ti,ab,kw OR (Acute Coronary Syndrome*):ti,ab,kw OR (NSTE-ACS):ti,ab,kw OR (Non–ST-Segment Elevation Acute Coronary Syndrome*):ti,ab,kw OR (STEMI):ti,ab,kw OR (NSTEMI):ti,ab,kw OR (ST-segment elevation myocardial infarction):ti,ab,kw OR (Non-ST-segment elevation myocardial infarction):ti,ab,kw OR (MINOCA):ti,ab,kw OR (Myocardial infarction with no obstructive coronary atherosclerosis):ti,ab,kw OR (xiongbi):ti,ab,kw OR (xiongbixintong):ti,ab,kw OR (xiongbi Xintong):ti,ab,kw OR (zhenxintong):ti,ab,kw OR (cuxintong):ti,ab,kw OR (Xintong):ti,ab,kw OR (juexintong):ti,ab,kw OR (cardiovascular shock):ti,ab,kw OR (cardiogenic stroke*):ti,ab,kw

#5 MeSH descriptor: [Percutaneous Coronary Intervention] explode all trees

#6 (percutaneous coronary intervention):ti,ab,kw OR (percutaneous coronary intervention*):ti,ab,kw OR (percutaneous coronary revascularization*):ti,ab,kw OR (coronary atherectom*):ti,ab,kw OR (transluminal coronary angioplast*):ti,ab,kw OR (transluminal coronary balloon dilation):ti,ab,kw OR (coronary balloon angioplast*):ti,ab,kw OR (PCI):ti,ab,kw OR (Percutaneous Transluminal Coronary Angioplast*):ti,ab,kw OR (PTCA):ti,ab,kw OR (Rotational Atherectom*):ti,ab,kw OR (Directional Coronary Atherectom*):ti,ab,kw OR (Balloon dilation):ti,ab,kw OR (Artery Stenting):ti,ab,kw

#7 #2 OR #3

#8 #4 OR #7

#9 #5 OR #6

#10 #8 AND #9

#11 #1 AND #10

15.Lemai granule

CNKI

(TKA=‘乐脉颗粒’ + ‘乐脉’) and ((TKA=‘心肌梗死’ + ‘心梗’ + ‘心肌梗塞’ + ‘急性冠脉综合征’+ ‘ACS’ + ‘STEMI’ + ‘NSTEMI’ + ‘胸痹’ + ‘胸痹心痛’ + ‘真心痛’ + ‘卒心痛’ + ‘心痛’ + ‘猝心痛’ + ‘厥心痛’) and (TKA=‘PCI’ + ‘PTCA’ + ‘经皮冠状动脉介入治疗术’ + ‘经皮冠状动脉介入’ + ‘经皮冠状动脉’ + ‘冠脉介入’ + ‘经皮冠脉’ + ‘血管成形术’ + ‘支架置入术’ + ‘冠状动脉成形术’ + ‘球囊’ + ‘支架’))

WangFang Data

主题:(“乐脉颗粒” OR “乐脉”) and ((主题:心肌梗死 OR 心梗 OR 心肌梗塞 OR 急性冠脉综合征 OR ACS OR STEMI OR NSTEMI OR 胸痹 OR 胸痹心痛 OR 真心痛 OR 卒心痛 OR 心痛 OR 猝心痛 OR 厥心痛) and 主题:(PCI OR PTCA OR 经皮冠状动脉介入治疗术 OR 经皮冠状动脉介入 OR 经皮冠状动脉 OR 冠脉介入 OR 经皮冠脉 OR 血管成形术 OR 支架置入术 OR 冠状动脉成形术 OR 球囊 OR 支架))

VIP

(M=(“乐脉颗粒” OR “乐脉”) OR R=(“乐脉颗粒” OR “乐脉”)) AND ((M=(心肌梗死 OR 心梗 OR 心肌梗塞 OR 急性冠脉综合征 OR STEMI OR NSTEMI OR 胸痹 OR 胸痹心痛 OR 真心痛 OR 卒心痛 OR 心痛 OR 猝心痛 OR 厥心痛) OR R=(心肌梗死 OR 心梗 OR 心肌梗塞 OR ACS OR STEMI OR NSTEMI OR 胸痹 OR 胸痹心痛 OR 真心痛 OR 卒心痛 OR 心痛 OR 猝心痛 OR 厥心痛)) AND (M=(PCI OR PTCA OR 经皮冠状动脉介入治疗术 OR 经皮冠状动脉介入 OR 经皮冠状动脉 OR 冠脉介入 OR 经皮冠脉 OR 血管成形术 OR 支架置入术 OR 冠状动脉成形术 OR 球囊 OR 支架) OR R=(PCI OR PTCA OR 经皮冠状动脉介入治疗术 OR 经皮冠状动脉介入 OR 经皮冠状动脉 OR 冠脉介入 OR 经皮冠脉 OR 血管成形术 OR 支架置入术 OR 冠状动脉成形术 OR 球囊 OR 支架)))

Web of Science

#1 TS=(Lemai granule* OR lemai keli OR LeMaiKeLi OR Lemai)

#2 TS=(myocardial infarct* OR heart infarct* OR cardiac infarct* OR cardiovascular stroke* OR cardiogenic shock OR heart attack* OR ACS OR STEMI OR NSTEMI OR ST-segment elevation myocardial infarction OR Non-ST-segment elevation myocardial infarction OR MINOCA OR Myocardial infarction with no obstructive coronary atherosclerosis OR xiongbi OR xiongbixintong OR xiongbi Xintong OR zhenxintong OR cuxintong OR Xintong OR juexintong OR cardiovascular shock OR cardiogenic stroke*)

#3 TS=(percutaneous coronary intervention OR percutaneous coronary intervention* OR percutaneous coronary revascularization* OR coronary atherectom* OR transluminal coronary angioplast* OR transluminal coronary balloon dilation OR coronary balloon angioplast* OR PCI OR Percutaneous Transluminal Coronary Angioplast* OR PTCA OR Rotational Atherectom* OR Directional Coronary Atherectom* OR Balloon dilation OR Artery Stenting)

#4 #2 AND #3

#5 #1 AND #4

PubMed

#1 “Lemai granule*”[Title/Abstract] OR “lemai keli”[Title/Abstract] OR “LeMaiKeLi”[Title/Abstract] OR “Lemai”[Title/Abstract]

#2 "myocardial infarction"[MeSH Terms] OR “Acute Coronary Syndrome”[MeSH Terms] OR "myocardial infarct*"[Title/Abstract] OR “Acute Coronary Syndrome”[Title/Abstract] OR “ACS”[Title/Abstract] OR “Acute Coronary Syndrome*”[Title/Abstract]OR “NSTE-ACS”[Title/Abstract] OR “Non–ST-Segment Elevation Acute Coronary Syndrome*”[Title/Abstract] OR "heart infarct*"[Title/Abstract] OR "cardiac infarct*"[Title/Abstract] OR "cardiovascular stroke*"[Title/Abstract] OR "cardiogenic shock"[Title/Abstract] OR “cardiovascular shock” [Title/Abstract] OR “cardiogenic stroke*” [Title/Abstract] OR "heart attack*"[Title/Abstract] OR "STEMI"[Title/Abstract] OR "NSTEMI"[Title/Abstract] OR “ST-segment elevation myocardial infarction”[Title/Abstract] OR “Non-ST-segment elevation myocardial infarction”[Title/Abstract] OR “MINOCA”[Title/Abstract] OR “Myocardial infarction with no obstructive coronary atherosclerosis” [Title/Abstract] OR “xiongbi” [Title/Abstract] OR “xiongbixintong” [Title/Abstract] OR “xiongbi xintong” [Title/Abstract] OR “zhenxintong” [Title/Abstract] OR “cuxintong” [Title/Abstract] OR “Xintong” [Title/Abstract] OR “juexintong” [Title/Abstract]

#3 “percutaneous coronary intervention” [MeSH Terms] OR “percutaneous coronary intervention*”[Title/Abstract] OR “percutaneous coronary revascularization*” [Title/Abstract] OR “coronary atherectom*”[Title/Abstract] OR “transluminal coronary angioplast*”[Title/Abstract] OR “transluminal coronary balloon dilation”[Title/Abstract] OR “coronary balloon angioplast*”[Title/Abstract] OR “PCI”[Title/Abstract] OR “Percutaneous Transluminal Coronary Angioplast*”[Title/Abstract] OR “PTCA”[Title/Abstract] OR “Rotational Atherectom*”[Title/Abstract] OR

“Directional Coronary Atherectom*”[Title/Abstract] OR “Balloon dilation”[Title/Abstract] OR “Artery Stenting”[Title/Abstract]

#4 #2 AND #3

#1 AND #4

Embase

#1 'Lemai granule*':ab,ti OR ‘lemai keli’:ab,ti OR ‘LeMaiKeLi’:ab,ti OR ‘Lemai':ab,ti

#2 'heart infarction'/exp

#3 'Acute Coronary Syndrome'/exp

#4 'cardiac infarct':ab,ti OR 'cardiac infarction':ab,ti OR 'cardial infarct':ab,ti OR 'heart attack':ab,ti OR 'heart infarct':ab,ti OR 'heart micro infarction':ab,ti OR 'heart muscle infarction':ab,ti OR 'infarction, heart':ab,ti OR 'myocardial infarct':ab,ti OR 'myocardial infarction':ab,ti OR 'myocardium infarct':ab,ti OR 'myocardium infarction':ab,ti OR 'premonitory infarction sign':ab,ti OR 'second heart attack':ab,ti OR 'subendocardial infarction':ab,ti OR 'transmural cardiac infarction':ab,ti OR 'transmural heart infarction':ab,ti OR 'transmural infarction, heart':ab,ti OR 'heart infarction':ab,ti OR ‘Acute Coronary Syndrome’:ab,ti OR ‘ACS’:ab,ti OR ‘Acute Coronary Syndrome*’:ab,ti OR ‘NSTE-ACS’:ab,ti OR ‘Non–ST-Segment Elevation Acute Coronary Syndrome*’:ab,ti OR 'STEMI':ab,ti OR 'NSTEMI':ab,ti OR ‘ST-segment elevation myocardial infarction’:ab,ti OR ‘Non-ST-segment elevation myocardial infarction’:ab,ti OR ‘MINOCA’:ab,ti OR ‘Myocardial infarction with no obstructive coronary atherosclerosis’:ab,ti OR ‘xiongbi’:ab,ti OR ‘xiongbixintong’:ab,ti OR ‘xiongbi Xintong’:ab,ti OR ‘zhenxintong’:ab,ti OR ‘cuxintong’:ab,ti OR ‘Xintong’:ab,ti OR ‘juexintong’:ab,ti OR ‘cardiovascular shock’:ab,ti OR ‘cardiogenic stroke*’:ab,ti

#5 ‘percutaneous coronary intervention'/exp

#6 ‘percutaneous coronary intervention*':ab,ti OR 'percutaneous coronary revascularization*':ab,ti OR 'coronary atherectom*':ab,ti OR 'transluminal coronary angioplast*':ab,ti OR 'transluminal coronary balloon dilation':ab,ti OR 'coronary balloon angioplast*':ab,ti OR 'PCI':ab,ti OR 'Percutaneous Transluminal Coronary Angioplast*':ab,ti OR 'PTCA':ab,ti OR 'Rotational Atherectom*':ab,ti OR 'Directional Coronary Atherectom*':ab,ti OR 'Balloon dilation':ab,ti OR 'Artery Stenting’:ab,ti

#7 #2 OR #3

#8 #4 OR #7

#9 #5 OR #6

#10 #8 AND #9

#11 #1 AND #10

Cochrane

#1 (Lemai granule*):ti,ab,kw OR (lemai keli):ti,ab,kw OR (LeMaiKeLi):ti,ab,kw OR (Lemai):ti,ab,kw

#2 MeSH descriptor: [Myocardial Infarction] explode all trees

#3 MeSH descriptor: [Acute Coronary Syndrome] explode all trees

#4 (myocardial infarct*):ti,ab,kw OR (heart infarct*):ti,ab,kw OR (cardiac infarct*):ti,ab,kw OR (cardiovascular stroke*):ti,ab,kw OR (cardiogenic shock):ti,ab,kw OR (heart attack*):ti,ab,kw OR (Acute Coronary Syndrome):ti,ab,kw OR (ACS):ti,ab,kw OR (Acute Coronary Syndrome*):ti,ab,kw OR (NSTE-ACS):ti,ab,kw OR (Non–ST-Segment Elevation Acute Coronary Syndrome*):ti,ab,kw OR (STEMI):ti,ab,kw OR (NSTEMI):ti,ab,kw OR (ST-segment elevation myocardial infarction):ti,ab,kw OR (Non-ST-segment elevation myocardial infarction):ti,ab,kw OR (MINOCA):ti,ab,kw OR (Myocardial infarction with no obstructive coronary atherosclerosis):ti,ab,kw OR (xiongbi):ti,ab,kw OR (xiongbixintong):ti,ab,kw OR (xiongbi Xintong):ti,ab,kw OR (zhenxintong):ti,ab,kw OR (cuxintong):ti,ab,kw OR (Xintong):ti,ab,kw OR (juexintong):ti,ab,kw OR (cardiovascular shock):ti,ab,kw OR (cardiogenic stroke*):ti,ab,kw

#5 MeSH descriptor: [Percutaneous Coronary Intervention] explode all trees

#6 (percutaneous coronary intervention):ti,ab,kw OR (percutaneous coronary intervention*):ti,ab,kw OR (percutaneous coronary revascularization*):ti,ab,kw OR (coronary atherectom*):ti,ab,kw OR (transluminal coronary angioplast*):ti,ab,kw OR (transluminal coronary balloon dilation):ti,ab,kw OR (coronary balloon angioplast*):ti,ab,kw OR (PCI):ti,ab,kw OR (Percutaneous Transluminal Coronary Angioplast*):ti,ab,kw OR (PTCA):ti,ab,kw OR (Rotational Atherectom*):ti,ab,kw OR (Directional Coronary Atherectom*):ti,ab,kw OR (Balloon dilation):ti,ab,kw OR (Artery Stenting):ti,ab,kw

#7 #2 OR #3

#8 #4 OR #7

#9 #5 OR #6

#10 #8 AND #9

#11 #1 AND #10

16.Di'ao Xinxuekang soft capsules

CNKI

(TKA=‘地奥心血康胶囊’ + ‘地奥心血康软胶囊’ + ‘地奥心血康’) and ((TKA=‘心肌梗死’ + ‘心梗’ + ‘心肌梗塞’ + ‘急性冠脉综合征’+ ‘ACS’ + ‘STEMI’ + ‘NSTEMI’ + ‘胸痹’ + ‘胸痹心痛’ + ‘真心痛’ + ‘卒心痛’ + ‘心痛’ + ‘猝心痛’ + ‘厥心痛’) and (TKA=‘PCI’ + ‘PTCA’ + ‘经皮冠状动脉介入治疗术’ + ‘经皮冠状动脉介入’ + ‘经皮冠状动脉’ + ‘冠脉介入’ + ‘经皮冠脉’ + ‘血管成形术’ + ‘支架置入术’ + ‘冠状动脉成形术’ + ‘球囊’ + ‘支架’))

WangFang Data

主题:(“地奥心血康胶囊” OR “地奥心血康软胶囊” OR “地奥心血康”) and ((主题:心肌梗死 OR 心梗 OR 心肌梗塞 OR 急性冠脉综合征 OR ACS OR STEMI OR NSTEMI OR 胸痹 OR 胸痹心痛 OR 真心痛 OR 卒心痛 OR 心痛 OR 猝心痛 OR 厥心痛) and 主题:(PCI OR PTCA OR 经皮冠状动脉介入治疗术 OR 经皮冠状动脉介入 OR 经皮冠状动脉 OR 冠脉介入 OR 经皮冠脉 OR 血管成形术 OR 支架置入术 OR 冠状动脉成形术 OR 球囊 OR 支架))

VIP

(M=(“地奥心血康胶囊” OR “地奥心血康软胶囊” OR “地奥心血康”) OR R=(“地奥心血康胶囊” OR “地奥心血康软胶囊” OR “地奥心血康”)) AND ((M=(心肌梗死 OR 心梗 OR 心肌梗塞 OR 急性冠脉综合征 OR STEMI OR NSTEMI OR 胸痹 OR 胸痹心痛 OR 真心痛 OR 卒心痛 OR 心痛 OR 猝心痛 OR 厥心痛) OR R=(心肌梗死 OR 心梗 OR 心肌梗塞 OR ACS OR STEMI OR NSTEMI OR 胸痹 OR 胸痹心痛 OR 真心痛 OR 卒心痛 OR 心痛 OR 猝心痛 OR 厥心痛)) AND (M=(PCI OR PTCA OR 经皮冠状动脉介入治疗术 OR 经皮冠状动脉介入 OR 经皮冠状动脉 OR 冠脉介入 OR 经皮冠脉 OR 血管成形术 OR 支架置入术 OR 冠状动脉成形术 OR 球囊 OR 支架) OR R=(PCI OR PTCA OR 经皮冠状动脉介入治疗术 OR 经皮冠状动脉介入 OR 经皮冠状动脉 OR 冠脉介入 OR 经皮冠脉 OR 血管成形术 OR 支架置入术 OR 冠状动脉成形术 OR 球囊 OR 支架)))

Web of Science

#1 TS=(Di'ao Xinxuekang capsule OR Xinxuekang capsule OR Di'ao Xinxuekang OR Xinxuekang OR Xinxuekang soft capsule OR Di'ao Xinxuekang soft capsule)

#2 TS=(myocardial infarct* OR heart infarct* OR cardiac infarct* OR cardiovascular stroke* OR cardiogenic shock OR heart attack* OR ACS OR STEMI OR NSTEMI OR ST-segment elevation myocardial infarction OR Non-ST-segment elevation myocardial infarction OR MINOCA OR Myocardial infarction with no obstructive coronary atherosclerosis OR xiongbi OR xiongbixintong OR xiongbi Xintong OR zhenxintong OR cuxintong OR Xintong OR juexintong OR cardiovascular shock OR cardiogenic stroke*)

#3 TS=(percutaneous coronary intervention OR percutaneous coronary intervention* OR percutaneous coronary revascularization* OR coronary atherectom* OR transluminal coronary angioplast* OR transluminal coronary balloon dilation OR coronary balloon angioplast* OR PCI OR Percutaneous Transluminal Coronary Angioplast* OR PTCA OR Rotational Atherectom* OR Directional Coronary Atherectom* OR Balloon dilation OR Artery Stenting)

#4 #2 AND #3

#5 #1 AND #4

PubMed

#1 “Xinxuekang soft capsule”[Title/Abstract] OR “Di'ao Xinxuekang soft capsule”[Title/Abstract] OR “Di'ao Xinxuekang capsule”[Title/Abstract] OR “Xinxuekang capsule”[Title/Abstract] OR “Di'ao Xinxuekang”[Title/Abstract] OR “Xinxuekang”[Title/Abstract]

#2 "myocardial infarction"[MeSH Terms] OR “Acute Coronary Syndrome”[MeSH Terms] OR "myocardial infarct*"[Title/Abstract] OR “Acute Coronary Syndrome”[Title/Abstract] OR “ACS”[Title/Abstract] OR “Acute Coronary Syndrome*”[Title/Abstract]OR “NSTE-ACS”[Title/Abstract] OR “Non–ST-Segment Elevation Acute Coronary Syndrome*”[Title/Abstract] OR "heart infarct*"[Title/Abstract] OR "cardiac infarct*"[Title/Abstract] OR "cardiovascular stroke*"[Title/Abstract] OR "cardiogenic shock"[Title/Abstract] OR “cardiovascular shock” [Title/Abstract] OR “cardiogenic stroke*” [Title/Abstract] OR "heart attack*"[Title/Abstract] OR "STEMI"[Title/Abstract] OR "NSTEMI"[Title/Abstract] OR “ST-segment elevation myocardial infarction”[Title/Abstract] OR “Non-ST-segment elevation myocardial infarction”[Title/Abstract] OR “MINOCA”[Title/Abstract] OR “Myocardial infarction with no obstructive coronary atherosclerosis” [Title/Abstract] OR “xiongbi” [Title/Abstract] OR “xiongbixintong” [Title/Abstract] OR “xiongbi xintong” [Title/Abstract] OR “zhenxintong” [Title/Abstract] OR “cuxintong” [Title/Abstract] OR “Xintong” [Title/Abstract] OR “juexintong” [Title/Abstract]

#3 “percutaneous coronary intervention” [MeSH Terms] OR “percutaneous coronary intervention*”[Title/Abstract] OR “percutaneous coronary revascularization*” [Title/Abstract] OR “coronary atherectom*”[Title/Abstract] OR “transluminal coronary angioplast*”[Title/Abstract] OR “transluminal coronary balloon dilation”[Title/Abstract] OR “coronary balloon angioplast*”[Title/Abstract] OR “PCI”[Title/Abstract] OR “Percutaneous Transluminal Coronary Angioplast*”[Title/Abstract] OR “PTCA”[Title/Abstract] OR “Rotational Atherectom*”[Title/Abstract] OR

“Directional Coronary Atherectom*”[Title/Abstract] OR “Balloon dilation”[Title/Abstract] OR “Artery Stenting”[Title/Abstract]

#4 #2 AND #3

#1 AND #4

Embase

#1 'Xinxuekang soft capsule':ab,ti OR ‘Di ao Xinxuekang soft capsule’:ab,ti OR ‘Di ao Xinxuekang capsule’:ab,ti OR ‘Xinxuekang capsule':ab,ti OR ‘Di ao Xinxuekang’:ab,ti OR ‘Xinxuekang’:ab,ti

#2 'heart infarction'/exp

#3 'Acute Coronary Syndrome'/exp

#4 'cardiac infarct':ab,ti OR 'cardiac infarction':ab,ti OR 'cardial infarct':ab,ti OR 'heart attack':ab,ti OR 'heart infarct':ab,ti OR 'heart micro infarction':ab,ti OR 'heart muscle infarction':ab,ti OR 'infarction, heart':ab,ti OR 'myocardial infarct':ab,ti OR 'myocardial infarction':ab,ti OR 'myocardium infarct':ab,ti OR 'myocardium infarction':ab,ti OR 'premonitory infarction sign':ab,ti OR 'second heart attack':ab,ti OR 'subendocardial infarction':ab,ti OR 'transmural cardiac infarction':ab,ti OR 'transmural heart infarction':ab,ti OR 'transmural infarction, heart':ab,ti OR 'heart infarction':ab,ti OR ‘Acute Coronary Syndrome’:ab,ti OR ‘ACS’:ab,ti OR ‘Acute Coronary Syndrome*’:ab,ti OR ‘NSTE-ACS’:ab,ti OR ‘Non–ST-Segment Elevation Acute Coronary Syndrome*’:ab,ti OR 'STEMI':ab,ti OR 'NSTEMI':ab,ti OR ‘ST-segment elevation myocardial infarction’:ab,ti OR ‘Non-ST-segment elevation myocardial infarction’:ab,ti OR ‘MINOCA’:ab,ti OR ‘Myocardial infarction with no obstructive coronary atherosclerosis’:ab,ti OR ‘xiongbi’:ab,ti OR ‘xiongbixintong’:ab,ti OR ‘xiongbi Xintong’:ab,ti OR ‘zhenxintong’:ab,ti OR ‘cuxintong’:ab,ti OR ‘Xintong’:ab,ti OR ‘juexintong’:ab,ti OR ‘cardiovascular shock’:ab,ti OR ‘cardiogenic stroke*’:ab,ti

#5 ‘percutaneous coronary intervention'/exp

#6 ‘percutaneous coronary intervention*':ab,ti OR 'percutaneous coronary revascularization*':ab,ti OR 'coronary atherectom*':ab,ti OR 'transluminal coronary angioplast*':ab,ti OR 'transluminal coronary balloon dilation':ab,ti OR 'coronary balloon angioplast*':ab,ti OR 'PCI':ab,ti OR 'Percutaneous Transluminal Coronary Angioplast*':ab,ti OR 'PTCA':ab,ti OR 'Rotational Atherectom*':ab,ti OR 'Directional Coronary Atherectom*':ab,ti OR 'Balloon dilation':ab,ti OR 'Artery Stenting’:ab,ti

#7 #2 OR #3

#8 #4 OR #7

#9 #5 OR #6

#10 #8 AND #9

#11 #1 AND #10

Cochrane

#1 (Xinxuekang soft capsule):ti,ab,kw OR (Di'ao Xinxuekang soft capsule):ti,ab,kw OR (Di'ao Xinxuekang capsule):ti,ab,kw OR (Xinxuekang capsule):ti,ab,kw OR (Di'ao Xinxuekang):ti,ab,kw OR (Xinxuekang):ti,ab,kw

#2 MeSH descriptor: [Myocardial Infarction] explode all trees

#3 MeSH descriptor: [Acute Coronary Syndrome] explode all trees

#4 (myocardial infarct*):ti,ab,kw OR (heart infarct*):ti,ab,kw OR (cardiac infarct*):ti,ab,kw OR (cardiovascular stroke*):ti,ab,kw OR (cardiogenic shock):ti,ab,kw OR (heart attack*):ti,ab,kw OR (Acute Coronary Syndrome):ti,ab,kw OR (ACS):ti,ab,kw OR (Acute Coronary Syndrome*):ti,ab,kw OR (NSTE-ACS):ti,ab,kw OR (Non–ST-Segment Elevation Acute Coronary Syndrome*):ti,ab,kw OR (STEMI):ti,ab,kw OR (NSTEMI):ti,ab,kw OR (ST-segment elevation myocardial infarction):ti,ab,kw OR (Non-ST-segment elevation myocardial infarction):ti,ab,kw OR (MINOCA):ti,ab,kw OR (Myocardial infarction with no obstructive coronary atherosclerosis):ti,ab,kw OR (xiongbi):ti,ab,kw OR (xiongbixintong):ti,ab,kw OR (xiongbi Xintong):ti,ab,kw OR (zhenxintong):ti,ab,kw OR (cuxintong):ti,ab,kw OR (Xintong):ti,ab,kw OR (juexintong):ti,ab,kw OR (cardiovascular shock):ti,ab,kw OR (cardiogenic stroke*):ti,ab,kw

#5 MeSH descriptor: [Percutaneous Coronary Intervention] explode all trees

#6 (percutaneous coronary intervention):ti,ab,kw OR (percutaneous coronary intervention*):ti,ab,kw OR (percutaneous coronary revascularization*):ti,ab,kw OR (coronary atherectom*):ti,ab,kw OR (transluminal coronary angioplast*):ti,ab,kw OR (transluminal coronary balloon dilation):ti,ab,kw OR (coronary balloon angioplast*):ti,ab,kw OR (PCI):ti,ab,kw OR (Percutaneous Transluminal Coronary Angioplast*):ti,ab,kw OR (PTCA):ti,ab,kw OR (Rotational Atherectom*):ti,ab,kw OR (Directional Coronary Atherectom*):ti,ab,kw OR (Balloon dilation):ti,ab,kw OR (Artery Stenting):ti,ab,kw

#7 #2 OR #3

#8 #4 OR #7

#9 #5 OR #6

#10 #8 AND #9

#11 #1 AND #10

17.Xuesaitong capsules

CNKI

(TKA=‘血府逐瘀胶囊’ + ‘血府逐瘀软胶囊’ + ‘血府逐瘀颗粒’ + ‘血府逐瘀’) and ((TKA=‘心肌梗死’ + ‘心梗’ + ‘心肌梗塞’ + ‘急性冠脉综合征’+ ‘ACS’ + ‘STEMI’ + ‘NSTEMI’ + ‘胸痹’ + ‘胸痹心痛’ + ‘真心痛’ + ‘卒心痛’ + ‘心痛’ + ‘猝心痛’ + ‘厥心痛’) and (TKA=‘PCI’ + ‘PTCA’ + ‘经皮冠状动脉介入治疗术’ + ‘经皮冠状动脉介入’ + ‘经皮冠状动脉’ + ‘冠脉介入’ + ‘经皮冠脉’ + ‘血管成形术’ + ‘支架置入术’ + ‘冠状动脉成形术’ + ‘球囊’ + ‘支架’))

WangFang Data

主题:(“血府逐瘀胶囊” OR “血府逐瘀软胶囊” OR “血府逐瘀颗粒” OR “血府逐瘀”) and ((主题:心肌梗死 OR 心梗 OR 心肌梗塞 OR 急性冠脉综合征 OR ACS OR STEMI OR NSTEMI OR 胸痹 OR 胸痹心痛 OR 真心痛 OR 卒心痛 OR 心痛 OR 猝心痛 OR 厥心痛) and 主题:(PCI OR PTCA OR 经皮冠状动脉介入治疗术 OR 经皮冠状动脉介入 OR 经皮冠状动脉 OR 冠脉介入 OR 经皮冠脉 OR 血管成形术 OR 支架置入术 OR 冠状动脉成形术 OR 球囊 OR 支架))

VIP

(M=(“血府逐瘀胶囊” OR “血府逐瘀软胶囊” OR “血府逐瘀颗粒” OR “血府逐瘀”) OR R=(“血府逐瘀胶囊” OR “血府逐瘀软胶囊” OR “血府逐瘀颗粒” OR “血府逐瘀”)) AND ((M=(心肌梗死 OR 心梗 OR 心肌梗塞 OR 急性冠脉综合征 OR STEMI OR NSTEMI OR 胸痹 OR 胸痹心痛 OR 真心痛 OR 卒心痛 OR 心痛 OR 猝心痛 OR 厥心痛) OR R=(心肌梗死 OR 心梗 OR 心肌梗塞 OR ACS OR STEMI OR NSTEMI OR 胸痹 OR 胸痹心痛 OR 真心痛 OR 卒心痛 OR 心痛 OR 猝心痛 OR 厥心痛)) AND (M=(PCI OR PTCA OR 经皮冠状动脉介入治疗术 OR 经皮冠状动脉介入 OR 经皮冠状动脉 OR 冠脉介入 OR 经皮冠脉 OR 血管成形术 OR 支架置入术 OR 冠状动脉成形术 OR 球囊 OR 支架) OR R=(PCI OR PTCA OR 经皮冠状动脉介入治疗术 OR 经皮冠状动脉介入 OR 经皮冠状动脉 OR 冠脉介入 OR 经皮冠脉 OR 血管成形术 OR 支架置入术 OR 冠状动脉成形术 OR 球囊 OR 支架)))

Web of Science

#1 TS=(xuefu zhuyu capsule* OR xuefu zhuyu soft capsule* OR xuefu zhuyu granule*)

#2 TS=(myocardial infarct* OR heart infarct* OR cardiac infarct* OR cardiovascular stroke* OR cardiogenic shock OR heart attack* OR ACS OR STEMI OR NSTEMI OR ST-segment elevation myocardial infarction OR Non-ST-segment elevation myocardial infarction OR MINOCA OR Myocardial infarction with no obstructive coronary atherosclerosis OR xiongbi OR xiongbixintong OR xiongbi Xintong OR zhenxintong OR cuxintong OR Xintong OR juexintong OR cardiovascular shock OR cardiogenic stroke*)

#3 TS=(percutaneous coronary intervention OR percutaneous coronary intervention* OR percutaneous coronary revascularization* OR coronary atherectom* OR transluminal coronary angioplast* OR transluminal coronary balloon dilation OR coronary balloon angioplast* OR PCI OR Percutaneous Transluminal Coronary Angioplast* OR PTCA OR Rotational Atherectom* OR Directional Coronary Atherectom* OR Balloon dilation OR Artery Stenting)

#4 #2 AND #3

#5 #1 AND #4

PubMed

#1 “xuefu zhuyu capsule*”[Title/Abstract] OR “xuefu zhuyu soft capsule*”[Title/Abstract] OR “xuefu zhuyu granule*”[Title/Abstract]

#2 "myocardial infarction"[MeSH Terms] OR “Acute Coronary Syndrome”[MeSH Terms] OR "myocardial infarct*"[Title/Abstract] OR “Acute Coronary Syndrome”[Title/Abstract] OR “ACS”[Title/Abstract] OR “Acute Coronary Syndrome*”[Title/Abstract]OR “NSTE-ACS”[Title/Abstract] OR “Non–ST-Segment Elevation Acute Coronary Syndrome*”[Title/Abstract] OR "heart infarct*"[Title/Abstract] OR "cardiac infarct*"[Title/Abstract] OR "cardiovascular stroke*"[Title/Abstract] OR "cardiogenic shock"[Title/Abstract] OR “cardiovascular shock” [Title/Abstract] OR “cardiogenic stroke*” [Title/Abstract] OR "heart attack*"[Title/Abstract] OR "STEMI"[Title/Abstract] OR "NSTEMI"[Title/Abstract] OR “ST-segment elevation myocardial infarction”[Title/Abstract] OR “Non-ST-segment elevation myocardial infarction”[Title/Abstract] OR “MINOCA”[Title/Abstract] OR “Myocardial infarction with no obstructive coronary atherosclerosis” [Title/Abstract] OR “xiongbi” [Title/Abstract] OR “xiongbixintong” [Title/Abstract] OR “xiongbi xintong” [Title/Abstract] OR “zhenxintong” [Title/Abstract] OR “cuxintong” [Title/Abstract] OR “Xintong” [Title/Abstract] OR “juexintong” [Title/Abstract]

#3 “percutaneous coronary intervention” [MeSH Terms] OR “percutaneous coronary intervention*”[Title/Abstract] OR “percutaneous coronary revascularization*” [Title/Abstract] OR “coronary atherectom*”[Title/Abstract] OR “transluminal coronary angioplast*”[Title/Abstract] OR “transluminal coronary balloon dilation”[Title/Abstract] OR “coronary balloon angioplast*”[Title/Abstract] OR “PCI”[Title/Abstract] OR “Percutaneous Transluminal Coronary Angioplast*”[Title/Abstract] OR “PTCA”[Title/Abstract] OR “Rotational Atherectom*”[Title/Abstract] OR

“Directional Coronary Atherectom*”[Title/Abstract] OR “Balloon dilation”[Title/Abstract] OR “Artery Stenting”[Title/Abstract]

#4 #2 AND #3

#1 AND #4

Embase

#1 'xuefu zhuyu capsule*':ab,ti OR ‘xuefu zhuyu soft capsule*’:ab,ti OR ‘xuefu zhuyu granule*’:ab,ti

#2 'heart infarction'/exp

#3 'Acute Coronary Syndrome'/exp

#4 'cardiac infarct':ab,ti OR 'cardiac infarction':ab,ti OR 'cardial infarct':ab,ti OR 'heart attack':ab,ti OR 'heart infarct':ab,ti OR 'heart micro infarction':ab,ti OR 'heart muscle infarction':ab,ti OR 'infarction, heart':ab,ti OR 'myocardial infarct':ab,ti OR 'myocardial infarction':ab,ti OR 'myocardium infarct':ab,ti OR 'myocardium infarction':ab,ti OR 'premonitory infarction sign':ab,ti OR 'second heart attack':ab,ti OR 'subendocardial infarction':ab,ti OR 'transmural cardiac infarction':ab,ti OR 'transmural heart infarction':ab,ti OR 'transmural infarction, heart':ab,ti OR 'heart infarction':ab,ti OR ‘Acute Coronary Syndrome’:ab,ti OR ‘ACS’:ab,ti OR ‘Acute Coronary Syndrome*’:ab,ti OR ‘NSTE-ACS’:ab,ti OR ‘Non–ST-Segment Elevation Acute Coronary Syndrome*’:ab,ti OR 'STEMI':ab,ti OR 'NSTEMI':ab,ti OR ‘ST-segment elevation myocardial infarction’:ab,ti OR ‘Non-ST-segment elevation myocardial infarction’:ab,ti OR ‘MINOCA’:ab,ti OR ‘Myocardial infarction with no obstructive coronary atherosclerosis’:ab,ti OR ‘xiongbi’:ab,ti OR ‘xiongbixintong’:ab,ti OR ‘xiongbi Xintong’:ab,ti OR ‘zhenxintong’:ab,ti OR ‘cuxintong’:ab,ti OR ‘Xintong’:ab,ti OR ‘juexintong’:ab,ti OR ‘cardiovascular shock’:ab,ti OR ‘cardiogenic stroke*’:ab,ti

#5 ‘percutaneous coronary intervention'/exp

#6 ‘percutaneous coronary intervention*':ab,ti OR 'percutaneous coronary revascularization*':ab,ti OR 'coronary atherectom*':ab,ti OR 'transluminal coronary angioplast*':ab,ti OR 'transluminal coronary balloon dilation':ab,ti OR 'coronary balloon angioplast*':ab,ti OR 'PCI':ab,ti OR 'Percutaneous Transluminal Coronary Angioplast*':ab,ti OR 'PTCA':ab,ti OR 'Rotational Atherectom*':ab,ti OR 'Directional Coronary Atherectom*':ab,ti OR 'Balloon dilation':ab,ti OR 'Artery Stenting’:ab,ti

#7 #2 OR #3

#8 #4 OR #7

#9 #5 OR #6

#10 #8 AND #9

#11 #1 AND #10

Cochrane

#1 (xuefu zhuyu capsule*):ti,ab,kw OR (xuefu zhuyu soft capsule*):ti,ab,kw OR (xuefu zhuyu granule*):ti,ab,kw

#2 MeSH descriptor: [Myocardial Infarction] explode all trees

#3 MeSH descriptor: [Acute Coronary Syndrome] explode all trees

#4 (myocardial infarct*):ti,ab,kw OR (heart infarct*):ti,ab,kw OR (cardiac infarct*):ti,ab,kw OR (cardiovascular stroke*):ti,ab,kw OR (cardiogenic shock):ti,ab,kw OR (heart attack*):ti,ab,kw OR (Acute Coronary Syndrome):ti,ab,kw OR (ACS):ti,ab,kw OR (Acute Coronary Syndrome*):ti,ab,kw OR (NSTE-ACS):ti,ab,kw OR (Non–ST-Segment Elevation Acute Coronary Syndrome*):ti,ab,kw OR (STEMI):ti,ab,kw OR (NSTEMI):ti,ab,kw OR (ST-segment elevation myocardial infarction):ti,ab,kw OR (Non-ST-segment elevation myocardial infarction):ti,ab,kw OR (MINOCA):ti,ab,kw OR (Myocardial infarction with no obstructive coronary atherosclerosis):ti,ab,kw OR (xiongbi):ti,ab,kw OR (xiongbixintong):ti,ab,kw OR (xiongbi Xintong):ti,ab,kw OR (zhenxintong):ti,ab,kw OR (cuxintong):ti,ab,kw OR (Xintong):ti,ab,kw OR (juexintong):ti,ab,kw OR (cardiovascular shock):ti,ab,kw OR (cardiogenic stroke*):ti,ab,kw

#5 MeSH descriptor: [Percutaneous Coronary Intervention] explode all trees

#6 (percutaneous coronary intervention):ti,ab,kw OR (percutaneous coronary intervention*):ti,ab,kw OR (percutaneous coronary revascularization*):ti,ab,kw OR (coronary atherectom*):ti,ab,kw OR (transluminal coronary angioplast*):ti,ab,kw OR (transluminal coronary balloon dilation):ti,ab,kw OR (coronary balloon angioplast*):ti,ab,kw OR (PCI):ti,ab,kw OR (Percutaneous Transluminal Coronary Angioplast*):ti,ab,kw OR (PTCA):ti,ab,kw OR (Rotational Atherectom*):ti,ab,kw OR (Directional Coronary Atherectom*):ti,ab,kw OR (Balloon dilation):ti,ab,kw OR (Artery Stenting):ti,ab,kw

#7 #2 OR #3

#8 #4 OR #7

#9 #5 OR #6

#10 #8 AND #9

#11 #1 AND #10

18.Xueshuan Xinmaining tablets

CNKI

(TKA=‘血栓心脉宁片’ + ‘血栓心脉宁’) and ((TKA=‘心肌梗死’ + ‘心梗’ + ‘心肌梗塞’ + ‘急性冠脉综合征’+ ‘ACS’ + ‘STEMI’ + ‘NSTEMI’ + ‘胸痹’ + ‘胸痹心痛’ + ‘真心痛’ + ‘卒心痛’ + ‘心痛’ + ‘猝心痛’ + ‘厥心痛’) and (TKA=‘PCI’ + ‘PTCA’ + ‘经皮冠状动脉介入治疗术’ + ‘经皮冠状动脉介入’ + ‘经皮冠状动脉’ + ‘冠脉介入’ + ‘经皮冠脉’ + ‘血管成形术’ + ‘支架置入术’ + ‘冠状动脉成形术’ + ‘球囊’ + ‘支架’))

WangFang Data

主题:(“血栓心脉宁片” OR “血栓心脉宁”) and ((主题:心肌梗死 OR 心梗 OR 心肌梗塞 OR 急性冠脉综合征 OR ACS OR STEMI OR NSTEMI OR 胸痹 OR 胸痹心痛 OR 真心痛 OR 卒心痛 OR 心痛 OR 猝心痛 OR 厥心痛) and 主题:(PCI OR PTCA OR 经皮冠状动脉介入治疗术 OR 经皮冠状动脉介入 OR 经皮冠状动脉 OR 冠脉介入 OR 经皮冠脉 OR 血管成形术 OR 支架置入术 OR 冠状动脉成形术 OR 球囊 OR 支架))

VIP

(M=(“血栓心脉宁片” OR “血栓心脉宁”) OR R=(“血栓心脉宁片” OR “血栓心脉宁”)) AND ((M=(心肌梗死 OR 心梗 OR 心肌梗塞 OR 急性冠脉综合征 OR STEMI OR NSTEMI OR 胸痹 OR 胸痹心痛 OR 真心痛 OR 卒心痛 OR 心痛 OR 猝心痛 OR 厥心痛) OR R=(心肌梗死 OR 心梗 OR 心肌梗塞 OR ACS OR STEMI OR NSTEMI OR 胸痹 OR 胸痹心痛 OR 真心痛 OR 卒心痛 OR 心痛 OR 猝心痛 OR 厥心痛)) AND (M=(PCI OR PTCA OR 经皮冠状动脉介入治疗术 OR 经皮冠状动脉介入 OR 经皮冠状动脉 OR 冠脉介入 OR 经皮冠脉 OR 血管成形术 OR 支架置入术 OR 冠状动脉成形术 OR 球囊 OR 支架) OR R=(PCI OR PTCA OR 经皮冠状动脉介入治疗术 OR 经皮冠状动脉介入 OR 经皮冠状动脉 OR 冠脉介入 OR 经皮冠脉 OR 血管成形术 OR 支架置入术 OR 冠状动脉成形术 OR 球囊 OR 支架)))

Web of Science

#1 TS=(Xueshuan Xinmaining Tablet* OR Xueshuan Xinmaining)

#2 TS=(myocardial infarct* OR heart infarct* OR cardiac infarct* OR cardiovascular stroke* OR cardiogenic shock OR heart attack* OR ACS OR STEMI OR NSTEMI OR ST-segment elevation myocardial infarction OR Non-ST-segment elevation myocardial infarction OR MINOCA OR Myocardial infarction with no obstructive coronary atherosclerosis OR xiongbi OR xiongbixintong OR xiongbi Xintong OR zhenxintong OR cuxintong OR Xintong OR juexintong OR cardiovascular shock OR cardiogenic stroke*)

#3 TS=(percutaneous coronary intervention OR percutaneous coronary intervention* OR percutaneous coronary revascularization* OR coronary atherectom* OR transluminal coronary angioplast* OR transluminal coronary balloon dilation OR coronary balloon angioplast* OR PCI OR Percutaneous Transluminal Coronary Angioplast* OR PTCA OR Rotational Atherectom* OR Directional Coronary Atherectom* OR Balloon dilation OR Artery Stenting)

#4 #2 AND #3

#5 #1 AND #4

PubMed

#1 “Xueshuan Xinmaining Tablet*”[Title/Abstract] OR “Xueshuan Xinmaining”[Title/Abstract]

#2 "myocardial infarction"[MeSH Terms] OR “Acute Coronary Syndrome”[MeSH Terms] OR "myocardial infarct*"[Title/Abstract] OR “Acute Coronary Syndrome”[Title/Abstract] OR “ACS”[Title/Abstract] OR “Acute Coronary Syndrome*”[Title/Abstract]OR “NSTE-ACS”[Title/Abstract] OR “Non–ST-Segment Elevation Acute Coronary Syndrome*”[Title/Abstract] OR "heart infarct*"[Title/Abstract] OR "cardiac infarct*"[Title/Abstract] OR "cardiovascular stroke*"[Title/Abstract] OR "cardiogenic shock"[Title/Abstract] OR “cardiovascular shock” [Title/Abstract] OR “cardiogenic stroke*” [Title/Abstract] OR "heart attack*"[Title/Abstract] OR "STEMI"[Title/Abstract] OR "NSTEMI"[Title/Abstract] OR “ST-segment elevation myocardial infarction”[Title/Abstract] OR “Non-ST-segment elevation myocardial infarction”[Title/Abstract] OR “MINOCA”[Title/Abstract] OR “Myocardial infarction with no obstructive coronary atherosclerosis” [Title/Abstract] OR “xiongbi” [Title/Abstract] OR “xiongbixintong” [Title/Abstract] OR “xiongbi xintong” [Title/Abstract] OR “zhenxintong” [Title/Abstract] OR “cuxintong” [Title/Abstract] OR “Xintong” [Title/Abstract] OR “juexintong” [Title/Abstract]

#3 “percutaneous coronary intervention” [MeSH Terms] OR “percutaneous coronary intervention*”[Title/Abstract] OR “percutaneous coronary revascularization*” [Title/Abstract] OR “coronary atherectom*”[Title/Abstract] OR “transluminal coronary angioplast*”[Title/Abstract] OR “transluminal coronary balloon dilation”[Title/Abstract] OR “coronary balloon angioplast*”[Title/Abstract] OR “PCI”[Title/Abstract] OR “Percutaneous Transluminal Coronary Angioplast*”[Title/Abstract] OR “PTCA”[Title/Abstract] OR “Rotational Atherectom*”[Title/Abstract] OR

“Directional Coronary Atherectom*”[Title/Abstract] OR “Balloon dilation”[Title/Abstract] OR “Artery Stenting”[Title/Abstract]

#4 #2 AND #3

#1 AND #4

Embase

#1 'Xueshuan Xinmaining Tablet*':ab,ti OR ‘Xueshuan Xinmaining’:ab,ti

#2 'heart infarction'/exp

#3 'Acute Coronary Syndrome'/exp

#4 'cardiac infarct':ab,ti OR 'cardiac infarction':ab,ti OR 'cardial infarct':ab,ti OR 'heart attack':ab,ti OR 'heart infarct':ab,ti OR 'heart micro infarction':ab,ti OR 'heart muscle infarction':ab,ti OR 'infarction, heart':ab,ti OR 'myocardial infarct':ab,ti OR 'myocardial infarction':ab,ti OR 'myocardium infarct':ab,ti OR 'myocardium infarction':ab,ti OR 'premonitory infarction sign':ab,ti OR 'second heart attack':ab,ti OR 'subendocardial infarction':ab,ti OR 'transmural cardiac infarction':ab,ti OR 'transmural heart infarction':ab,ti OR 'transmural infarction, heart':ab,ti OR 'heart infarction':ab,ti OR ‘Acute Coronary Syndrome’:ab,ti OR ‘ACS’:ab,ti OR ‘Acute Coronary Syndrome*’:ab,ti OR ‘NSTE-ACS’:ab,ti OR ‘Non–ST-Segment Elevation Acute Coronary Syndrome*’:ab,ti OR 'STEMI':ab,ti OR 'NSTEMI':ab,ti OR ‘ST-segment elevation myocardial infarction’:ab,ti OR ‘Non-ST-segment elevation myocardial infarction’:ab,ti OR ‘MINOCA’:ab,ti OR ‘Myocardial infarction with no obstructive coronary atherosclerosis’:ab,ti OR ‘xiongbi’:ab,ti OR ‘xiongbixintong’:ab,ti OR ‘xiongbi Xintong’:ab,ti OR ‘zhenxintong’:ab,ti OR ‘cuxintong’:ab,ti OR ‘Xintong’:ab,ti OR ‘juexintong’:ab,ti OR ‘cardiovascular shock’:ab,ti OR ‘cardiogenic stroke*’:ab,ti

#5 ‘percutaneous coronary intervention'/exp

#6 ‘percutaneous coronary intervention*':ab,ti OR 'percutaneous coronary revascularization*':ab,ti OR 'coronary atherectom*':ab,ti OR 'transluminal coronary angioplast*':ab,ti OR 'transluminal coronary balloon dilation':ab,ti OR 'coronary balloon angioplast*':ab,ti OR 'PCI':ab,ti OR 'Percutaneous Transluminal Coronary Angioplast*':ab,ti OR 'PTCA':ab,ti OR 'Rotational Atherectom*':ab,ti OR 'Directional Coronary Atherectom*':ab,ti OR 'Balloon dilation':ab,ti OR 'Artery Stenting’:ab,ti

#7 #2 OR #3

#8 #4 OR #7

#9 #5 OR #6

#10 #8 AND #9

#11 #1 AND #10

Cochrane

#1 (Xueshuan Xinmaining Tablet*):ti,ab,kw OR (Xueshuan Xinmaining):ti,ab,kw

#2 MeSH descriptor: [Myocardial Infarction] explode all trees

#3 MeSH descriptor: [Acute Coronary Syndrome] explode all trees

#4 (myocardial infarct*):ti,ab,kw OR (heart infarct*):ti,ab,kw OR (cardiac infarct*):ti,ab,kw OR (cardiovascular stroke*):ti,ab,kw OR (cardiogenic shock):ti,ab,kw OR (heart attack*):ti,ab,kw OR (Acute Coronary Syndrome):ti,ab,kw OR (ACS):ti,ab,kw OR (Acute Coronary Syndrome*):ti,ab,kw OR (NSTE-ACS):ti,ab,kw OR (Non–ST-Segment Elevation Acute Coronary Syndrome*):ti,ab,kw OR (STEMI):ti,ab,kw OR (NSTEMI):ti,ab,kw OR (ST-segment elevation myocardial infarction):ti,ab,kw OR (Non-ST-segment elevation myocardial infarction):ti,ab,kw OR (MINOCA):ti,ab,kw OR (Myocardial infarction with no obstructive coronary atherosclerosis):ti,ab,kw OR (xiongbi):ti,ab,kw OR (xiongbixintong):ti,ab,kw OR (xiongbi Xintong):ti,ab,kw OR (zhenxintong):ti,ab,kw OR (cuxintong):ti,ab,kw OR (Xintong):ti,ab,kw OR (juexintong):ti,ab,kw OR (cardiovascular shock):ti,ab,kw OR (cardiogenic stroke*):ti,ab,kw

#5 MeSH descriptor: [Percutaneous Coronary Intervention] explode all trees

#6 (percutaneous coronary intervention):ti,ab,kw OR (percutaneous coronary intervention*):ti,ab,kw OR (percutaneous coronary revascularization*):ti,ab,kw OR (coronary atherectom*):ti,ab,kw OR (transluminal coronary angioplast*):ti,ab,kw OR (transluminal coronary balloon dilation):ti,ab,kw OR (coronary balloon angioplast*):ti,ab,kw OR (PCI):ti,ab,kw OR (Percutaneous Transluminal Coronary Angioplast*):ti,ab,kw OR (PTCA):ti,ab,kw OR (Rotational Atherectom*):ti,ab,kw OR (Directional Coronary Atherectom*):ti,ab,kw OR (Balloon dilation):ti,ab,kw OR (Artery Stenting):ti,ab,kw

#7 #2 OR #3

#8 #4 OR #7

#9 #5 OR #6

#10 #8 AND #9

#11 #1 AND #10

19.Dengzhan Shengmai capsules

CNKI

(TKA=‘灯盏生脉胶囊’ + ‘灯盏生脉’) and ((TKA=‘心肌梗死’ + ‘心梗’ + ‘心肌梗塞’ + ‘急性冠脉综合征’+ ‘ACS’ + ‘STEMI’ + ‘NSTEMI’ + ‘胸痹’ + ‘胸痹心痛’ + ‘真心痛’ + ‘卒心痛’ + ‘心痛’ + ‘猝心痛’ + ‘厥心痛’) and (TKA=‘PCI’ + ‘PTCA’ + ‘经皮冠状动脉介入治疗术’ + ‘经皮冠状动脉介入’ + ‘经皮冠状动脉’ + ‘冠脉介入’ + ‘经皮冠脉’ + ‘血管成形术’ + ‘支架置入术’ + ‘冠状动脉成形术’ + ‘球囊’ + ‘支架’))

WangFang Data

主题:(“灯盏生脉胶囊” OR “灯盏生脉”) and ((主题:心肌梗死 OR 心梗 OR 心肌梗塞 OR 急性冠脉综合征 OR ACS OR STEMI OR NSTEMI OR 胸痹 OR 胸痹心痛 OR 真心痛 OR 卒心痛 OR 心痛 OR 猝心痛 OR 厥心痛) and 主题:(PCI OR PTCA OR 经皮冠状动脉介入治疗术 OR 经皮冠状动脉介入 OR 经皮冠状动脉 OR 冠脉介入 OR 经皮冠脉 OR 血管成形术 OR 支架置入术 OR 冠状动脉成形术 OR 球囊 OR 支架))

VIP

(M=(“灯盏生脉胶囊” OR “灯盏生脉”) OR R=(“灯盏生脉胶囊” OR “灯盏生脉”)) AND ((M=(心肌梗死 OR 心梗 OR 心肌梗塞 OR 急性冠脉综合征 OR STEMI OR NSTEMI OR 胸痹 OR 胸痹心痛 OR 真心痛 OR 卒心痛 OR 心痛 OR 猝心痛 OR 厥心痛) OR R=(心肌梗死 OR 心梗 OR 心肌梗塞 OR ACS OR STEMI OR NSTEMI OR 胸痹 OR 胸痹心痛 OR 真心痛 OR 卒心痛 OR 心痛 OR 猝心痛 OR 厥心痛)) AND (M=(PCI OR PTCA OR 经皮冠状动脉介入治疗术 OR 经皮冠状动脉介入 OR 经皮冠状动脉 OR 冠脉介入 OR 经皮冠脉 OR 血管成形术 OR 支架置入术 OR 冠状动脉成形术 OR 球囊 OR 支架) OR R=(PCI OR PTCA OR 经皮冠状动脉介入治疗术 OR 经皮冠状动脉介入 OR 经皮冠状动脉 OR 冠脉介入 OR 经皮冠脉 OR 血管成形术 OR 支架置入术 OR 冠状动脉成形术 OR 球囊 OR 支架)))

Web of Science

#1 TS=(Dengzhanshengmai capsule* OR Deng-Zhan-Sheng-Mai capsule* OR Dengzhanshengmai)

#2 TS=(myocardial infarct* OR heart infarct* OR cardiac infarct* OR cardiovascular stroke* OR cardiogenic shock OR heart attack* OR ACS OR STEMI OR NSTEMI OR ST-segment elevation myocardial infarction OR Non-ST-segment elevation myocardial infarction OR MINOCA OR Myocardial infarction with no obstructive coronary atherosclerosis OR xiongbi OR xiongbixintong OR xiongbi Xintong OR zhenxintong OR cuxintong OR Xintong OR juexintong OR cardiovascular shock OR cardiogenic stroke*)

#3 TS=(percutaneous coronary intervention OR percutaneous coronary intervention* OR percutaneous coronary revascularization* OR coronary atherectom* OR transluminal coronary angioplast* OR transluminal coronary balloon dilation OR coronary balloon angioplast* OR PCI OR Percutaneous Transluminal Coronary Angioplast* OR PTCA OR Rotational Atherectom* OR Directional Coronary Atherectom* OR Balloon dilation OR Artery Stenting)

#4 #2 AND #3

#5 #1 AND #4

PubMed

#1 “Dengzhanshengmai capsule*”[Title/Abstract] OR “Deng-Zhan-Sheng-Mai capsule*”[Title/Abstract] OR “Dengzhanshengmai”[Title/Abstract]

#2 "myocardial infarction"[MeSH Terms] OR “Acute Coronary Syndrome”[MeSH Terms] OR "myocardial infarct*"[Title/Abstract] OR “Acute Coronary Syndrome”[Title/Abstract] OR “ACS”[Title/Abstract] OR “Acute Coronary Syndrome*”[Title/Abstract]OR “NSTE-ACS”[Title/Abstract] OR “Non–ST-Segment Elevation Acute Coronary Syndrome*”[Title/Abstract] OR "heart infarct*"[Title/Abstract] OR "cardiac infarct*"[Title/Abstract] OR "cardiovascular stroke*"[Title/Abstract] OR "cardiogenic shock"[Title/Abstract] OR “cardiovascular shock” [Title/Abstract] OR “cardiogenic stroke*” [Title/Abstract] OR "heart attack*"[Title/Abstract] OR "STEMI"[Title/Abstract] OR "NSTEMI"[Title/Abstract] OR “ST-segment elevation myocardial infarction”[Title/Abstract] OR “Non-ST-segment elevation myocardial infarction”[Title/Abstract] OR “MINOCA”[Title/Abstract] OR “Myocardial infarction with no obstructive coronary atherosclerosis” [Title/Abstract] OR “xiongbi” [Title/Abstract] OR “xiongbixintong” [Title/Abstract] OR “xiongbi xintong” [Title/Abstract] OR “zhenxintong” [Title/Abstract] OR “cuxintong” [Title/Abstract] OR “Xintong” [Title/Abstract] OR “juexintong” [Title/Abstract]

#3 “percutaneous coronary intervention” [MeSH Terms] OR “percutaneous coronary intervention*”[Title/Abstract] OR “percutaneous coronary revascularization*” [Title/Abstract] OR “coronary atherectom*”[Title/Abstract] OR “transluminal coronary angioplast*”[Title/Abstract] OR “transluminal coronary balloon dilation”[Title/Abstract] OR “coronary balloon angioplast*”[Title/Abstract] OR “PCI”[Title/Abstract] OR “Percutaneous Transluminal Coronary Angioplast*”[Title/Abstract] OR “PTCA”[Title/Abstract] OR “Rotational Atherectom*”[Title/Abstract] OR

“Directional Coronary Atherectom*”[Title/Abstract] OR “Balloon dilation”[Title/Abstract] OR “Artery Stenting”[Title/Abstract]

#4 #2 AND #3

#1 AND #4

Embase

#1 'Dengzhanshengmai':ab,ti OR ‘Deng-Zhan-Sheng-Mai capsule*’:ab,ti OR ‘Dengzhanshengmai capsule*’:ab,ti

#2 'heart infarction'/exp

#3 'Acute Coronary Syndrome'/exp

#4 'cardiac infarct':ab,ti OR 'cardiac infarction':ab,ti OR 'cardial infarct':ab,ti OR 'heart attack':ab,ti OR 'heart infarct':ab,ti OR 'heart micro infarction':ab,ti OR 'heart muscle infarction':ab,ti OR 'infarction, heart':ab,ti OR 'myocardial infarct':ab,ti OR 'myocardial infarction':ab,ti OR 'myocardium infarct':ab,ti OR 'myocardium infarction':ab,ti OR 'premonitory infarction sign':ab,ti OR 'second heart attack':ab,ti OR 'subendocardial infarction':ab,ti OR 'transmural cardiac infarction':ab,ti OR 'transmural heart infarction':ab,ti OR 'transmural infarction, heart':ab,ti OR 'heart infarction':ab,ti OR ‘Acute Coronary Syndrome’:ab,ti OR ‘ACS’:ab,ti OR ‘Acute Coronary Syndrome*’:ab,ti OR ‘NSTE-ACS’:ab,ti OR ‘Non–ST-Segment Elevation Acute Coronary Syndrome*’:ab,ti OR 'STEMI':ab,ti OR 'NSTEMI':ab,ti OR ‘ST-segment elevation myocardial infarction’:ab,ti OR ‘Non-ST-segment elevation myocardial infarction’:ab,ti OR ‘MINOCA’:ab,ti OR ‘Myocardial infarction with no obstructive coronary atherosclerosis’:ab,ti OR ‘xiongbi’:ab,ti OR ‘xiongbixintong’:ab,ti OR ‘xiongbi Xintong’:ab,ti OR ‘zhenxintong’:ab,ti OR ‘cuxintong’:ab,ti OR ‘Xintong’:ab,ti OR ‘juexintong’:ab,ti OR ‘cardiovascular shock’:ab,ti OR ‘cardiogenic stroke*’:ab,ti

#5 ‘percutaneous coronary intervention'/exp

#6 ‘percutaneous coronary intervention*':ab,ti OR 'percutaneous coronary revascularization*':ab,ti OR 'coronary atherectom*':ab,ti OR 'transluminal coronary angioplast*':ab,ti OR 'transluminal coronary balloon dilation':ab,ti OR 'coronary balloon angioplast*':ab,ti OR 'PCI':ab,ti OR 'Percutaneous Transluminal Coronary Angioplast*':ab,ti OR 'PTCA':ab,ti OR 'Rotational Atherectom*':ab,ti OR 'Directional Coronary Atherectom*':ab,ti OR 'Balloon dilation':ab,ti OR 'Artery Stenting’:ab,ti

#7 #2 OR #3

#8 #4 OR #7

#9 #5 OR #6

#10 #8 AND #9

#11 #1 AND #10

Cochrane

#1 (Dengzhanshengmai capsule*):ti,ab,kw OR (Deng-Zhan-Sheng-Mai capsule*):ti,ab,kw OR (Dengzhanshengmai):ti,ab,kw

#2 MeSH descriptor: [Myocardial Infarction] explode all trees

#3 MeSH descriptor: [Acute Coronary Syndrome] explode all trees

#4 (myocardial infarct*):ti,ab,kw OR (heart infarct*):ti,ab,kw OR (cardiac infarct*):ti,ab,kw OR (cardiovascular stroke*):ti,ab,kw OR (cardiogenic shock):ti,ab,kw OR (heart attack*):ti,ab,kw OR (Acute Coronary Syndrome):ti,ab,kw OR (ACS):ti,ab,kw OR (Acute Coronary Syndrome*):ti,ab,kw OR (NSTE-ACS):ti,ab,kw OR (Non–ST-Segment Elevation Acute Coronary Syndrome*):ti,ab,kw OR (STEMI):ti,ab,kw OR (NSTEMI):ti,ab,kw OR (ST-segment elevation myocardial infarction):ti,ab,kw OR (Non-ST-segment elevation myocardial infarction):ti,ab,kw OR (MINOCA):ti,ab,kw OR (Myocardial infarction with no obstructive coronary atherosclerosis):ti,ab,kw OR (xiongbi):ti,ab,kw OR (xiongbixintong):ti,ab,kw OR (xiongbi Xintong):ti,ab,kw OR (zhenxintong):ti,ab,kw OR (cuxintong):ti,ab,kw OR (Xintong):ti,ab,kw OR (juexintong):ti,ab,kw OR (cardiovascular shock):ti,ab,kw OR (cardiogenic stroke*):ti,ab,kw

#5 MeSH descriptor: [Percutaneous Coronary Intervention] explode all trees

#6 (percutaneous coronary intervention):ti,ab,kw OR (percutaneous coronary intervention*):ti,ab,kw OR (percutaneous coronary revascularization*):ti,ab,kw OR (coronary atherectom*):ti,ab,kw OR (transluminal coronary angioplast*):ti,ab,kw OR (transluminal coronary balloon dilation):ti,ab,kw OR (coronary balloon angioplast*):ti,ab,kw OR (PCI):ti,ab,kw OR (Percutaneous Transluminal Coronary Angioplast*):ti,ab,kw OR (PTCA):ti,ab,kw OR (Rotational Atherectom*):ti,ab,kw OR (Directional Coronary Atherectom*):ti,ab,kw OR (Balloon dilation):ti,ab,kw OR (Artery Stenting):ti,ab,kw

#7 #2 OR #3

#8 #4 OR #7

#9 #5 OR #6

#10 #8 AND #9

#11 #1 AND #10

20.Erigeron breviscapus injection

CNKI

(TKA=‘灯盏细辛注射液’ + ‘灯盏细辛’) and ((TKA=‘心肌梗死’ + ‘心梗’ + ‘心肌梗塞’ + ‘急性冠脉综合征’+ ‘ACS’ + ‘STEMI’ + ‘NSTEMI’ + ‘胸痹’ + ‘胸痹心痛’ + ‘真心痛’ + ‘卒心痛’ + ‘心痛’ + ‘猝心痛’ + ‘厥心痛’) and (TKA=‘PCI’ + ‘PTCA’ + ‘经皮冠状动脉介入治疗术’ + ‘经皮冠状动脉介入’ + ‘经皮冠状动脉’ + ‘冠脉介入’ + ‘经皮冠脉’ + ‘血管成形术’ + ‘支架置入术’ + ‘冠状动脉成形术’ + ‘球囊’ + ‘支架’))

WangFang Data

主题:(“灯盏细辛注射液” OR “灯盏细辛”) and ((主题:心肌梗死 OR 心梗 OR 心肌梗塞 OR 急性冠脉综合征 OR ACS OR STEMI OR NSTEMI OR 胸痹 OR 胸痹心痛 OR 真心痛 OR 卒心痛 OR 心痛 OR 猝心痛 OR 厥心痛) and 主题:(PCI OR PTCA OR 经皮冠状动脉介入治疗术 OR 经皮冠状动脉介入 OR 经皮冠状动脉 OR 冠脉介入 OR 经皮冠脉 OR 血管成形术 OR 支架置入术 OR 冠状动脉成形术 OR 球囊 OR 支架))

VIP

(M=(“灯盏细辛注射液” OR “灯盏细辛”) OR R=(“灯盏细辛注射液” OR “灯盏细辛”)) AND ((M=(心肌梗死 OR 心梗 OR 心肌梗塞 OR 急性冠脉综合征 OR STEMI OR NSTEMI OR 胸痹 OR 胸痹心痛 OR 真心痛 OR 卒心痛 OR 心痛 OR 猝心痛 OR 厥心痛) OR R=(心肌梗死 OR 心梗 OR 心肌梗塞 OR ACS OR STEMI OR NSTEMI OR 胸痹 OR 胸痹心痛 OR 真心痛 OR 卒心痛 OR 心痛 OR 猝心痛 OR 厥心痛)) AND (M=(PCI OR PTCA OR 经皮冠状动脉介入治疗术 OR 经皮冠状动脉介入 OR 经皮冠状动脉 OR 冠脉介入 OR 经皮冠脉 OR 血管成形术 OR 支架置入术 OR 冠状动脉成形术 OR 球囊 OR 支架) OR R=(PCI OR PTCA OR 经皮冠状动脉介入治疗术 OR 经皮冠状动脉介入 OR 经皮冠状动脉 OR 冠脉介入 OR 经皮冠脉 OR 血管成形术 OR 支架置入术 OR 冠状动脉成形术 OR 球囊 OR 支架)))

Web of Science

#1 TS=(Dengzhanxixin injection OR Erigeron breviscapus injection OR Dengzhanxixin OR Erigeron breviscapus)

#2 TS=(myocardial infarct* OR heart infarct* OR cardiac infarct* OR cardiovascular stroke* OR cardiogenic shock OR heart attack* OR ACS OR STEMI OR NSTEMI OR ST-segment elevation myocardial infarction OR Non-ST-segment elevation myocardial infarction OR MINOCA OR Myocardial infarction with no obstructive coronary atherosclerosis OR xiongbi OR xiongbixintong OR xiongbi Xintong OR zhenxintong OR cuxintong OR Xintong OR juexintong OR cardiovascular shock OR cardiogenic stroke*)

#3 TS=(percutaneous coronary intervention OR percutaneous coronary intervention* OR percutaneous coronary revascularization* OR coronary atherectom* OR transluminal coronary angioplast* OR transluminal coronary balloon dilation OR coronary balloon angioplast* OR PCI OR Percutaneous Transluminal Coronary Angioplast* OR PTCA OR Rotational Atherectom* OR Directional Coronary Atherectom* OR Balloon dilation OR Artery Stenting)

#4 #2 AND #3

#5 #1 AND #4

PubMed

#1 “Dengzhanxixin injection”[Title/Abstract] OR “Erigeron breviscapus injection”[Title/Abstract] OR “Dengzhanxixin”[Title/Abstract] OR “Erigeron breviscapus”[Title/Abstract]

#2 "myocardial infarction"[MeSH Terms] OR “Acute Coronary Syndrome”[MeSH Terms] OR "myocardial infarct*"[Title/Abstract] OR “Acute Coronary Syndrome”[Title/Abstract] OR “ACS”[Title/Abstract] OR “Acute Coronary Syndrome*”[Title/Abstract]OR “NSTE-ACS”[Title/Abstract] OR “Non–ST-Segment Elevation Acute Coronary Syndrome*”[Title/Abstract] OR "heart infarct*"[Title/Abstract] OR "cardiac infarct*"[Title/Abstract] OR "cardiovascular stroke*"[Title/Abstract] OR "cardiogenic shock"[Title/Abstract] OR “cardiovascular shock” [Title/Abstract] OR “cardiogenic stroke*” [Title/Abstract] OR "heart attack*"[Title/Abstract] OR "STEMI"[Title/Abstract] OR "NSTEMI"[Title/Abstract] OR “ST-segment elevation myocardial infarction”[Title/Abstract] OR “Non-ST-segment elevation myocardial infarction”[Title/Abstract] OR “MINOCA”[Title/Abstract] OR “Myocardial infarction with no obstructive coronary atherosclerosis” [Title/Abstract] OR “xiongbi” [Title/Abstract] OR “xiongbixintong” [Title/Abstract] OR “xiongbi xintong” [Title/Abstract] OR “zhenxintong” [Title/Abstract] OR “cuxintong” [Title/Abstract] OR “Xintong” [Title/Abstract] OR “juexintong” [Title/Abstract]

#3 “percutaneous coronary intervention” [MeSH Terms] OR “percutaneous coronary intervention*”[Title/Abstract] OR “percutaneous coronary revascularization*” [Title/Abstract] OR “coronary atherectom*”[Title/Abstract] OR “transluminal coronary angioplast*”[Title/Abstract] OR “transluminal coronary balloon dilation”[Title/Abstract] OR “coronary balloon angioplast*”[Title/Abstract] OR “PCI”[Title/Abstract] OR “Percutaneous Transluminal Coronary Angioplast*”[Title/Abstract] OR “PTCA”[Title/Abstract] OR “Rotational Atherectom*”[Title/Abstract] OR

“Directional Coronary Atherectom*”[Title/Abstract] OR “Balloon dilation”[Title/Abstract] OR “Artery Stenting”[Title/Abstract]

#4 #2 AND #3

#1 AND #4

Embase

#1 'Dengzhanxixin injection':ab,ti OR ‘Erigeron breviscapus injection’:ab,ti OR ‘Dengzhanxixin’:ab,ti OR ‘Erigeron breviscapus':ab,ti

#2 'heart infarction'/exp

#3 'Acute Coronary Syndrome'/exp

#4 'cardiac infarct':ab,ti OR 'cardiac infarction':ab,ti OR 'cardial infarct':ab,ti OR 'heart attack':ab,ti OR 'heart infarct':ab,ti OR 'heart micro infarction':ab,ti OR 'heart muscle infarction':ab,ti OR 'infarction, heart':ab,ti OR 'myocardial infarct':ab,ti OR 'myocardial infarction':ab,ti OR 'myocardium infarct':ab,ti OR 'myocardium infarction':ab,ti OR 'premonitory infarction sign':ab,ti OR 'second heart attack':ab,ti OR 'subendocardial infarction':ab,ti OR 'transmural cardiac infarction':ab,ti OR 'transmural heart infarction':ab,ti OR 'transmural infarction, heart':ab,ti OR 'heart infarction':ab,ti OR ‘Acute Coronary Syndrome’:ab,ti OR ‘ACS’:ab,ti OR ‘Acute Coronary Syndrome*’:ab,ti OR ‘NSTE-ACS’:ab,ti OR ‘Non–ST-Segment Elevation Acute Coronary Syndrome*’:ab,ti OR 'STEMI':ab,ti OR 'NSTEMI':ab,ti OR ‘ST-segment elevation myocardial infarction’:ab,ti OR ‘Non-ST-segment elevation myocardial infarction’:ab,ti OR ‘MINOCA’:ab,ti OR ‘Myocardial infarction with no obstructive coronary atherosclerosis’:ab,ti OR ‘xiongbi’:ab,ti OR ‘xiongbixintong’:ab,ti OR ‘xiongbi Xintong’:ab,ti OR ‘zhenxintong’:ab,ti OR ‘cuxintong’:ab,ti OR ‘Xintong’:ab,ti OR ‘juexintong’:ab,ti OR ‘cardiovascular shock’:ab,ti OR ‘cardiogenic stroke*’:ab,ti

#5 ‘percutaneous coronary intervention'/exp

#6 ‘percutaneous coronary intervention*':ab,ti OR 'percutaneous coronary revascularization*':ab,ti OR 'coronary atherectom*':ab,ti OR 'transluminal coronary angioplast*':ab,ti OR 'transluminal coronary balloon dilation':ab,ti OR 'coronary balloon angioplast*':ab,ti OR 'PCI':ab,ti OR 'Percutaneous Transluminal Coronary Angioplast*':ab,ti OR 'PTCA':ab,ti OR 'Rotational Atherectom*':ab,ti OR 'Directional Coronary Atherectom*':ab,ti OR 'Balloon dilation':ab,ti OR 'Artery Stenting’:ab,ti

#7 #2 OR #3

#8 #4 OR #7

#9 #5 OR #6

#10 #8 AND #9

#11 #1 AND #10

Cochrane

#1 (Dengzhanxixin injection):ti,ab,kw OR (Erigeron breviscapus injection):ti,ab,kw OR (Dengzhanxixin):ti,ab,kw OR (Erigeron breviscapus):ti,ab,kw

#2 MeSH descriptor: [Myocardial Infarction] explode all trees

#3 MeSH descriptor: [Acute Coronary Syndrome] explode all trees

#4 (myocardial infarct*):ti,ab,kw OR (heart infarct*):ti,ab,kw OR (cardiac infarct*):ti,ab,kw OR (cardiovascular stroke*):ti,ab,kw OR (cardiogenic shock):ti,ab,kw OR (heart attack*):ti,ab,kw OR (Acute Coronary Syndrome):ti,ab,kw OR (ACS):ti,ab,kw OR (Acute Coronary Syndrome*):ti,ab,kw OR (NSTE-ACS):ti,ab,kw OR (Non–ST-Segment Elevation Acute Coronary Syndrome*):ti,ab,kw OR (STEMI):ti,ab,kw OR (NSTEMI):ti,ab,kw OR (ST-segment elevation myocardial infarction):ti,ab,kw OR (Non-ST-segment elevation myocardial infarction):ti,ab,kw OR (MINOCA):ti,ab,kw OR (Myocardial infarction with no obstructive coronary atherosclerosis):ti,ab,kw OR (xiongbi):ti,ab,kw OR (xiongbixintong):ti,ab,kw OR (xiongbi Xintong):ti,ab,kw OR (zhenxintong):ti,ab,kw OR (cuxintong):ti,ab,kw OR (Xintong):ti,ab,kw OR (juexintong):ti,ab,kw OR (cardiovascular shock):ti,ab,kw OR (cardiogenic stroke*):ti,ab,kw

#5 MeSH descriptor: [Percutaneous Coronary Intervention] explode all trees

#6 (percutaneous coronary intervention):ti,ab,kw OR (percutaneous coronary intervention*):ti,ab,kw OR (percutaneous coronary revascularization*):ti,ab,kw OR (coronary atherectom*):ti,ab,kw OR (transluminal coronary angioplast*):ti,ab,kw OR (transluminal coronary balloon dilation):ti,ab,kw OR (coronary balloon angioplast*):ti,ab,kw OR (PCI):ti,ab,kw OR (Percutaneous Transluminal Coronary Angioplast*):ti,ab,kw OR (PTCA):ti,ab,kw OR (Rotational Atherectom*):ti,ab,kw OR (Directional Coronary Atherectom*):ti,ab,kw OR (Balloon dilation):ti,ab,kw OR (Artery Stenting):ti,ab,kw

#7 #2 OR #3

#8 #4 OR #7

#9 #5 OR #6

#10 #8 AND #9

#11 #1 AND #10

21.Erigeron breviscapus granule

CNKI

(TKA=‘灯盏细辛颗粒’ + ‘灯盏细辛’) and ((TKA=‘心肌梗死’ + ‘心梗’ + ‘心肌梗塞’ + ‘急性冠脉综合征’+ ‘ACS’ + ‘STEMI’ + ‘NSTEMI’ + ‘胸痹’ + ‘胸痹心痛’ + ‘真心痛’ + ‘卒心痛’ + ‘心痛’ + ‘猝心痛’ + ‘厥心痛’) and (TKA=‘PCI’ + ‘PTCA’ + ‘经皮冠状动脉介入治疗术’ + ‘经皮冠状动脉介入’ + ‘经皮冠状动脉’ + ‘冠脉介入’ + ‘经皮冠脉’ + ‘血管成形术’ + ‘支架置入术’ + ‘冠状动脉成形术’ + ‘球囊’ + ‘支架’))

WangFang Data

主题:(“灯盏细辛颗粒” OR “灯盏细辛”) and ((主题:心肌梗死 OR 心梗 OR 心肌梗塞 OR 急性冠脉综合征 OR ACS OR STEMI OR NSTEMI OR 胸痹 OR 胸痹心痛 OR 真心痛 OR 卒心痛 OR 心痛 OR 猝心痛 OR 厥心痛) and 主题:(PCI OR PTCA OR 经皮冠状动脉介入治疗术 OR 经皮冠状动脉介入 OR 经皮冠状动脉 OR 冠脉介入 OR 经皮冠脉 OR 血管成形术 OR 支架置入术 OR 冠状动脉成形术 OR 球囊 OR 支架))

VIP

(M=(“灯盏细辛颗粒” OR “灯盏细辛”) OR R=(“灯盏细辛颗粒” OR “灯盏细辛”)) AND ((M=(心肌梗死 OR 心梗 OR 心肌梗塞 OR 急性冠脉综合征 OR STEMI OR NSTEMI OR 胸痹 OR 胸痹心痛 OR 真心痛 OR 卒心痛 OR 心痛 OR 猝心痛 OR 厥心痛) OR R=(心肌梗死 OR 心梗 OR 心肌梗塞 OR ACS OR STEMI OR NSTEMI OR 胸痹 OR 胸痹心痛 OR 真心痛 OR 卒心痛 OR 心痛 OR 猝心痛 OR 厥心痛)) AND (M=(PCI OR PTCA OR 经皮冠状动脉介入治疗术 OR 经皮冠状动脉介入 OR 经皮冠状动脉 OR 冠脉介入 OR 经皮冠脉 OR 血管成形术 OR 支架置入术 OR 冠状动脉成形术 OR 球囊 OR 支架) OR R=(PCI OR PTCA OR 经皮冠状动脉介入治疗术 OR 经皮冠状动脉介入 OR 经皮冠状动脉 OR 冠脉介入 OR 经皮冠脉 OR 血管成形术 OR 支架置入术 OR 冠状动脉成形术 OR 球囊 OR 支架)))

Web of Science

#1 TS=(dengzhanxixin granule OR Erigeron breviscapus granule OR dengzhanxixin OR Erigeron breviscapus)

#2 TS=(myocardial infarct* OR heart infarct* OR cardiac infarct* OR cardiovascular stroke* OR cardiogenic shock OR heart attack* OR ACS OR STEMI OR NSTEMI OR ST-segment elevation myocardial infarction OR Non-ST-segment elevation myocardial infarction OR MINOCA OR Myocardial infarction with no obstructive coronary atherosclerosis OR xiongbi OR xiongbixintong OR xiongbi Xintong OR zhenxintong OR cuxintong OR Xintong OR juexintong OR cardiovascular shock OR cardiogenic stroke*)

#3 TS=(percutaneous coronary intervention OR percutaneous coronary intervention* OR percutaneous coronary revascularization* OR coronary atherectom* OR transluminal coronary angioplast* OR transluminal coronary balloon dilation OR coronary balloon angioplast* OR PCI OR Percutaneous Transluminal Coronary Angioplast* OR PTCA OR Rotational Atherectom* OR Directional Coronary Atherectom* OR Balloon dilation OR Artery Stenting)

#4 #2 AND #3

#5 #1 AND #4

PubMed

#1 “dengzhanxixin granule”[Title/Abstract] OR “Erigeron breviscapus granule”[Title/Abstract] OR “dengzhanxixin”[Title/Abstract] OR “Erigeron breviscapus”[Title/Abstract]

#2 "myocardial infarction"[MeSH Terms] OR “Acute Coronary Syndrome”[MeSH Terms] OR "myocardial infarct*"[Title/Abstract] OR “Acute Coronary Syndrome”[Title/Abstract] OR “ACS”[Title/Abstract] OR “Acute Coronary Syndrome*”[Title/Abstract]OR “NSTE-ACS”[Title/Abstract] OR “Non–ST-Segment Elevation Acute Coronary Syndrome*”[Title/Abstract] OR "heart infarct*"[Title/Abstract] OR "cardiac infarct*"[Title/Abstract] OR "cardiovascular stroke*"[Title/Abstract] OR "cardiogenic shock"[Title/Abstract] OR “cardiovascular shock” [Title/Abstract] OR “cardiogenic stroke*” [Title/Abstract] OR "heart attack*"[Title/Abstract] OR "STEMI"[Title/Abstract] OR "NSTEMI"[Title/Abstract] OR “ST-segment elevation myocardial infarction”[Title/Abstract] OR “Non-ST-segment elevation myocardial infarction”[Title/Abstract] OR “MINOCA”[Title/Abstract] OR “Myocardial infarction with no obstructive coronary atherosclerosis” [Title/Abstract] OR “xiongbi” [Title/Abstract] OR “xiongbixintong” [Title/Abstract] OR “xiongbi xintong” [Title/Abstract] OR “zhenxintong” [Title/Abstract] OR “cuxintong” [Title/Abstract] OR “Xintong” [Title/Abstract] OR “juexintong” [Title/Abstract]

#3 “percutaneous coronary intervention” [MeSH Terms] OR “percutaneous coronary intervention*”[Title/Abstract] OR “percutaneous coronary revascularization*” [Title/Abstract] OR “coronary atherectom*”[Title/Abstract] OR “transluminal coronary angioplast*”[Title/Abstract] OR “transluminal coronary balloon dilation”[Title/Abstract] OR “coronary balloon angioplast*”[Title/Abstract] OR “PCI”[Title/Abstract] OR “Percutaneous Transluminal Coronary Angioplast*”[Title/Abstract] OR “PTCA”[Title/Abstract] OR “Rotational Atherectom*”[Title/Abstract] OR

“Directional Coronary Atherectom*”[Title/Abstract] OR “Balloon dilation”[Title/Abstract] OR “Artery Stenting”[Title/Abstract]

#4 #2 AND #3

#1 AND #4

Embase

#1 'dengzhanxixin granule':ab,ti OR ‘Erigeron breviscapus granule’:ab,ti OR ‘dengzhanxixin’:ab,ti OR ‘Erigeron breviscapus':ab,ti

#2 'heart infarction'/exp

#3 'Acute Coronary Syndrome'/exp

#4 'cardiac infarct':ab,ti OR 'cardiac infarction':ab,ti OR 'cardial infarct':ab,ti OR 'heart attack':ab,ti OR 'heart infarct':ab,ti OR 'heart micro infarction':ab,ti OR 'heart muscle infarction':ab,ti OR 'infarction, heart':ab,ti OR 'myocardial infarct':ab,ti OR 'myocardial infarction':ab,ti OR 'myocardium infarct':ab,ti OR 'myocardium infarction':ab,ti OR 'premonitory infarction sign':ab,ti OR 'second heart attack':ab,ti OR 'subendocardial infarction':ab,ti OR 'transmural cardiac infarction':ab,ti OR 'transmural heart infarction':ab,ti OR 'transmural infarction, heart':ab,ti OR 'heart infarction':ab,ti OR ‘Acute Coronary Syndrome’:ab,ti OR ‘ACS’:ab,ti OR ‘Acute Coronary Syndrome*’:ab,ti OR ‘NSTE-ACS’:ab,ti OR ‘Non–ST-Segment Elevation Acute Coronary Syndrome*’:ab,ti OR 'STEMI':ab,ti OR 'NSTEMI':ab,ti OR ‘ST-segment elevation myocardial infarction’:ab,ti OR ‘Non-ST-segment elevation myocardial infarction’:ab,ti OR ‘MINOCA’:ab,ti OR ‘Myocardial infarction with no obstructive coronary atherosclerosis’:ab,ti OR ‘xiongbi’:ab,ti OR ‘xiongbixintong’:ab,ti OR ‘xiongbi Xintong’:ab,ti OR ‘zhenxintong’:ab,ti OR ‘cuxintong’:ab,ti OR ‘Xintong’:ab,ti OR ‘juexintong’:ab,ti OR ‘cardiovascular shock’:ab,ti OR ‘cardiogenic stroke*’:ab,ti

#5 ‘percutaneous coronary intervention'/exp

#6 ‘percutaneous coronary intervention*':ab,ti OR 'percutaneous coronary revascularization*':ab,ti OR 'coronary atherectom*':ab,ti OR 'transluminal coronary angioplast*':ab,ti OR 'transluminal coronary balloon dilation':ab,ti OR 'coronary balloon angioplast*':ab,ti OR 'PCI':ab,ti OR 'Percutaneous Transluminal Coronary Angioplast*':ab,ti OR 'PTCA':ab,ti OR 'Rotational Atherectom*':ab,ti OR 'Directional Coronary Atherectom*':ab,ti OR 'Balloon dilation':ab,ti OR 'Artery Stenting’:ab,ti

#7 #2 OR #3

#8 #4 OR #7

#9 #5 OR #6

#10 #8 AND #9

#11 #1 AND #10

Cochrane

#1 (dengzhanxixin granule):ti,ab,kw OR (Erigeron breviscapus granule):ti,ab,kw OR (dengzhanxixin):ti,ab,kw OR (Erigeron breviscapus):ti,ab,kw

#2 MeSH descriptor: [Myocardial Infarction] explode all trees

#3 MeSH descriptor: [Acute Coronary Syndrome] explode all trees

#4 (myocardial infarct*):ti,ab,kw OR (heart infarct*):ti,ab,kw OR (cardiac infarct*):ti,ab,kw OR (cardiovascular stroke*):ti,ab,kw OR (cardiogenic shock):ti,ab,kw OR (heart attack*):ti,ab,kw OR (Acute Coronary Syndrome):ti,ab,kw OR (ACS):ti,ab,kw OR (Acute Coronary Syndrome*):ti,ab,kw OR (NSTE-ACS):ti,ab,kw OR (Non–ST-Segment Elevation Acute Coronary Syndrome*):ti,ab,kw OR (STEMI):ti,ab,kw OR (NSTEMI):ti,ab,kw OR (ST-segment elevation myocardial infarction):ti,ab,kw OR (Non-ST-segment elevation myocardial infarction):ti,ab,kw OR (MINOCA):ti,ab,kw OR (Myocardial infarction with no obstructive coronary atherosclerosis):ti,ab,kw OR (xiongbi):ti,ab,kw OR (xiongbixintong):ti,ab,kw OR (xiongbi Xintong):ti,ab,kw OR (zhenxintong):ti,ab,kw OR (cuxintong):ti,ab,kw OR (Xintong):ti,ab,kw OR (juexintong):ti,ab,kw OR (cardiovascular shock):ti,ab,kw OR (cardiogenic stroke*):ti,ab,kw

#5 MeSH descriptor: [Percutaneous Coronary Intervention] explode all trees

#6 (percutaneous coronary intervention):ti,ab,kw OR (percutaneous coronary intervention*):ti,ab,kw OR (percutaneous coronary revascularization*):ti,ab,kw OR (coronary atherectom*):ti,ab,kw OR (transluminal coronary angioplast*):ti,ab,kw OR (transluminal coronary balloon dilation):ti,ab,kw OR (coronary balloon angioplast*):ti,ab,kw OR (PCI):ti,ab,kw OR (Percutaneous Transluminal Coronary Angioplast*):ti,ab,kw OR (PTCA):ti,ab,kw OR (Rotational Atherectom*):ti,ab,kw OR (Directional Coronary Atherectom*):ti,ab,kw OR (Balloon dilation):ti,ab,kw OR (Artery Stenting):ti,ab,kw

#7 #2 OR #3

#8 #4 OR #7

#9 #5 OR #6

#10 #8 AND #9

#11 #1 AND #10

22.Qishen Yiqi dropping pills

CNKI

(TKA=‘芪参益气滴丸’ + ‘芪参益气’) and ((TKA=‘心肌梗死’ + ‘心梗’ + ‘心肌梗塞’ + ‘急性冠脉综合征’+ ‘ACS’ + ‘STEMI’ + ‘NSTEMI’ + ‘胸痹’ + ‘胸痹心痛’ + ‘真心痛’ + ‘卒心痛’ + ‘心痛’ + ‘猝心痛’ + ‘厥心痛’) and (TKA=‘PCI’ + ‘PTCA’ + ‘经皮冠状动脉介入治疗术’ + ‘经皮冠状动脉介入’ + ‘经皮冠状动脉’ + ‘冠脉介入’ + ‘经皮冠脉’ + ‘血管成形术’ + ‘支架置入术’ + ‘冠状动脉成形术’ + ‘球囊’ + ‘支架’))

WangFang Data

主题:(“芪参益气滴丸” OR “芪参益气”) and ((主题:心肌梗死 OR 心梗 OR 心肌梗塞 OR 急性冠脉综合征 OR ACS OR STEMI OR NSTEMI OR 胸痹 OR 胸痹心痛 OR 真心痛 OR 卒心痛 OR 心痛 OR 猝心痛 OR 厥心痛) and 主题:(PCI OR PTCA OR 经皮冠状动脉介入治疗术 OR 经皮冠状动脉介入 OR 经皮冠状动脉 OR 冠脉介入 OR 经皮冠脉 OR 血管成形术 OR 支架置入术 OR 冠状动脉成形术 OR 球囊 OR 支架))

VIP

(M=(“芪参益气滴丸” OR “芪参益气”) OR R=(“芪参益气滴丸” OR “芪参益气”)) AND ((M=(心肌梗死 OR 心梗 OR 心肌梗塞 OR 急性冠脉综合征 OR STEMI OR NSTEMI OR 胸痹 OR 胸痹心痛 OR 真心痛 OR 卒心痛 OR 心痛 OR 猝心痛 OR 厥心痛) OR R=(心肌梗死 OR 心梗 OR 心肌梗塞 OR ACS OR STEMI OR NSTEMI OR 胸痹 OR 胸痹心痛 OR 真心痛 OR 卒心痛 OR 心痛 OR 猝心痛 OR 厥心痛)) AND (M=(PCI OR PTCA OR 经皮冠状动脉介入治疗术 OR 经皮冠状动脉介入 OR 经皮冠状动脉 OR 冠脉介入 OR 经皮冠脉 OR 血管成形术 OR 支架置入术 OR 冠状动脉成形术 OR 球囊 OR 支架) OR R=(PCI OR PTCA OR 经皮冠状动脉介入治疗术 OR 经皮冠状动脉介入 OR 经皮冠状动脉 OR 冠脉介入 OR 经皮冠脉 OR 血管成形术 OR 支架置入术 OR 冠状动脉成形术 OR 球囊 OR 支架)))

Web of Science

#1 TS=(Qishen Yiqi dropping pill* OR Qishen Yiqi diwan OR Qishen Yiqi)

#2 TS=(myocardial infarct* OR heart infarct* OR cardiac infarct* OR cardiovascular stroke* OR cardiogenic shock OR heart attack* OR ACS OR STEMI OR NSTEMI OR ST-segment elevation myocardial infarction OR Non-ST-segment elevation myocardial infarction OR MINOCA OR Myocardial infarction with no obstructive coronary atherosclerosis OR xiongbi OR xiongbixintong OR xiongbi Xintong OR zhenxintong OR cuxintong OR Xintong OR juexintong OR cardiovascular shock OR cardiogenic stroke*)

#3 TS=(percutaneous coronary intervention OR percutaneous coronary intervention* OR percutaneous coronary revascularization* OR coronary atherectom* OR transluminal coronary angioplast* OR transluminal coronary balloon dilation OR coronary balloon angioplast* OR PCI OR Percutaneous Transluminal Coronary Angioplast* OR PTCA OR Rotational Atherectom* OR Directional Coronary Atherectom* OR Balloon dilation OR Artery Stenting)

#4 #2 AND #3

#5 #1 AND #4

PubMed

#1 “Qishen Yiqi dropping pill*”[Title/Abstract] OR “Qishen Yiqi diwan”[Title/Abstract] OR “Qishen Yiqi”[Title/Abstract]

#2 "myocardial infarction"[MeSH Terms] OR “Acute Coronary Syndrome”[MeSH Terms] OR "myocardial infarct*"[Title/Abstract] OR “Acute Coronary Syndrome”[Title/Abstract] OR “ACS”[Title/Abstract] OR “Acute Coronary Syndrome*”[Title/Abstract]OR “NSTE-ACS”[Title/Abstract] OR “Non–ST-Segment Elevation Acute Coronary Syndrome*”[Title/Abstract] OR "heart infarct*"[Title/Abstract] OR "cardiac infarct*"[Title/Abstract] OR "cardiovascular stroke*"[Title/Abstract] OR "cardiogenic shock"[Title/Abstract] OR “cardiovascular shock” [Title/Abstract] OR “cardiogenic stroke*” [Title/Abstract] OR "heart attack*"[Title/Abstract] OR "STEMI"[Title/Abstract] OR "NSTEMI"[Title/Abstract] OR “ST-segment elevation myocardial infarction”[Title/Abstract] OR “Non-ST-segment elevation myocardial infarction”[Title/Abstract] OR “MINOCA”[Title/Abstract] OR “Myocardial infarction with no obstructive coronary atherosclerosis” [Title/Abstract] OR “xiongbi” [Title/Abstract] OR “xiongbixintong” [Title/Abstract] OR “xiongbi xintong” [Title/Abstract] OR “zhenxintong” [Title/Abstract] OR “cuxintong” [Title/Abstract] OR “Xintong” [Title/Abstract] OR “juexintong” [Title/Abstract]

#3 “percutaneous coronary intervention” [MeSH Terms] OR “percutaneous coronary intervention*”[Title/Abstract] OR “percutaneous coronary revascularization*” [Title/Abstract] OR “coronary atherectom*”[Title/Abstract] OR “transluminal coronary angioplast*”[Title/Abstract] OR “transluminal coronary balloon dilation”[Title/Abstract] OR “coronary balloon angioplast*”[Title/Abstract] OR “PCI”[Title/Abstract] OR “Percutaneous Transluminal Coronary Angioplast*”[Title/Abstract] OR “PTCA”[Title/Abstract] OR “Rotational Atherectom*”[Title/Abstract] OR

“Directional Coronary Atherectom*”[Title/Abstract] OR “Balloon dilation”[Title/Abstract] OR “Artery Stenting”[Title/Abstract]

#4 #2 AND #3

#1 AND #4

Embase

#1 'Qishen Yiqi dropping pill*':ab,ti OR ‘Qishen Yiqi diwan’:ab,ti OR ‘Qishen Yiqi’:ab,ti

#2 'heart infarction'/exp

#3 'Acute Coronary Syndrome'/exp

#4 'cardiac infarct':ab,ti OR 'cardiac infarction':ab,ti OR 'cardial infarct':ab,ti OR 'heart attack':ab,ti OR 'heart infarct':ab,ti OR 'heart micro infarction':ab,ti OR 'heart muscle infarction':ab,ti OR 'infarction, heart':ab,ti OR 'myocardial infarct':ab,ti OR 'myocardial infarction':ab,ti OR 'myocardium infarct':ab,ti OR 'myocardium infarction':ab,ti OR 'premonitory infarction sign':ab,ti OR 'second heart attack':ab,ti OR 'subendocardial infarction':ab,ti OR 'transmural cardiac infarction':ab,ti OR 'transmural heart infarction':ab,ti OR 'transmural infarction, heart':ab,ti OR 'heart infarction':ab,ti OR ‘Acute Coronary Syndrome’:ab,ti OR ‘ACS’:ab,ti OR ‘Acute Coronary Syndrome*’:ab,ti OR ‘NSTE-ACS’:ab,ti OR ‘Non–ST-Segment Elevation Acute Coronary Syndrome*’:ab,ti OR 'STEMI':ab,ti OR 'NSTEMI':ab,ti OR ‘ST-segment elevation myocardial infarction’:ab,ti OR ‘Non-ST-segment elevation myocardial infarction’:ab,ti OR ‘MINOCA’:ab,ti OR ‘Myocardial infarction with no obstructive coronary atherosclerosis’:ab,ti OR ‘xiongbi’:ab,ti OR ‘xiongbixintong’:ab,ti OR ‘xiongbi Xintong’:ab,ti OR ‘zhenxintong’:ab,ti OR ‘cuxintong’:ab,ti OR ‘Xintong’:ab,ti OR ‘juexintong’:ab,ti OR ‘cardiovascular shock’:ab,ti OR ‘cardiogenic stroke*’:ab,ti

#5 ‘percutaneous coronary intervention'/exp

#6 ‘percutaneous coronary intervention*':ab,ti OR 'percutaneous coronary revascularization*':ab,ti OR 'coronary atherectom*':ab,ti OR 'transluminal coronary angioplast*':ab,ti OR 'transluminal coronary balloon dilation':ab,ti OR 'coronary balloon angioplast*':ab,ti OR 'PCI':ab,ti OR 'Percutaneous Transluminal Coronary Angioplast*':ab,ti OR 'PTCA':ab,ti OR 'Rotational Atherectom*':ab,ti OR 'Directional Coronary Atherectom*':ab,ti OR 'Balloon dilation':ab,ti OR 'Artery Stenting’:ab,ti

#7 #2 OR #3

#8 #4 OR #7

#9 #5 OR #6

#10 #8 AND #9

#11 #1 AND #10

Cochrane

#1 (Qishen Yiqi dropping pill*):ti,ab,kw OR (Qishen Yiqi diwan):ti,ab,kw OR (Qishen Yiqi):ti,ab,kw

#2 MeSH descriptor: [Myocardial Infarction] explode all trees

#3 MeSH descriptor: [Acute Coronary Syndrome] explode all trees

#4 (myocardial infarct*):ti,ab,kw OR (heart infarct*):ti,ab,kw OR (cardiac infarct*):ti,ab,kw OR (cardiovascular stroke*):ti,ab,kw OR (cardiogenic shock):ti,ab,kw OR (heart attack*):ti,ab,kw OR (Acute Coronary Syndrome):ti,ab,kw OR (ACS):ti,ab,kw OR (Acute Coronary Syndrome*):ti,ab,kw OR (NSTE-ACS):ti,ab,kw OR (Non–ST-Segment Elevation Acute Coronary Syndrome*):ti,ab,kw OR (STEMI):ti,ab,kw OR (NSTEMI):ti,ab,kw OR (ST-segment elevation myocardial infarction):ti,ab,kw OR (Non-ST-segment elevation myocardial infarction):ti,ab,kw OR (MINOCA):ti,ab,kw OR (Myocardial infarction with no obstructive coronary atherosclerosis):ti,ab,kw OR (xiongbi):ti,ab,kw OR (xiongbixintong):ti,ab,kw OR (xiongbi Xintong):ti,ab,kw OR (zhenxintong):ti,ab,kw OR (cuxintong):ti,ab,kw OR (Xintong):ti,ab,kw OR (juexintong):ti,ab,kw OR (cardiovascular shock):ti,ab,kw OR (cardiogenic stroke*):ti,ab,kw

#5 MeSH descriptor: [Percutaneous Coronary Intervention] explode all trees

#6 (percutaneous coronary intervention):ti,ab,kw OR (percutaneous coronary intervention*):ti,ab,kw OR (percutaneous coronary revascularization*):ti,ab,kw OR (coronary atherectom*):ti,ab,kw OR (transluminal coronary angioplast*):ti,ab,kw OR (transluminal coronary balloon dilation):ti,ab,kw OR (coronary balloon angioplast*):ti,ab,kw OR (PCI):ti,ab,kw OR (Percutaneous Transluminal Coronary Angioplast*):ti,ab,kw OR (PTCA):ti,ab,kw OR (Rotational Atherectom*):ti,ab,kw OR (Directional Coronary Atherectom*):ti,ab,kw OR (Balloon dilation):ti,ab,kw OR (Artery Stenting):ti,ab,kw

#7 #2 OR #3

#8 #4 OR #7

#9 #5 OR #6

#10 #8 AND #9

#11 #1 AND #10

23.Ixeris sonchifolia extract Injection

CNKI

(TKA=‘苦碟子注射液’ + ‘苦碟子’) and ((TKA=‘心肌梗死’ + ‘心梗’ + ‘心肌梗塞’ + ‘急性冠脉综合征’+ ‘ACS’ + ‘STEMI’ + ‘NSTEMI’ + ‘胸痹’ + ‘胸痹心痛’ + ‘真心痛’ + ‘卒心痛’ + ‘心痛’ + ‘猝心痛’ + ‘厥心痛’ ) and (TKA=‘PCI’ + ‘PTCA’ + ‘经皮冠状动脉介入治疗术’ + ‘经皮冠状动脉介入’ + ‘经皮冠状动脉’ + ‘冠脉介入’ + ‘经皮冠脉’ + ‘血管成形术’ + ‘支架置入术’ + ‘冠状动脉成形术’ + ‘球囊’ + ‘支架’))

WangFang Data

主题:(“苦碟子注射液” OR “苦碟子”) and ((主题:心肌梗死 OR 心梗 OR 心肌梗塞 OR 急性冠脉综合征 OR ACS OR STEMI OR NSTEMI OR 胸痹 OR 胸痹心痛 OR 真心痛 OR 卒心痛 OR 心痛 OR 猝心痛 OR 厥心痛) and 主题:(PCI OR PTCA OR 经皮冠状动脉介入治疗术 OR 经皮冠状动脉介入 OR 经皮冠状动脉 OR 冠脉介入 OR 经皮冠脉 OR 血管成形术 OR 支架置入术 OR 冠状动脉成形术 OR 球囊 OR 支架))

VIP

(M=(“苦碟子注射液” OR “苦碟子”) OR R=(“苦碟子注射液” OR “苦碟子”)) AND ((M=(心肌梗死 OR 心梗 OR 心肌梗塞 OR 急性冠脉综合征 OR STEMI OR NSTEMI OR 胸痹 OR 胸痹心痛 OR 真心痛 OR 卒心痛 OR 心痛 OR 猝心痛 OR 厥心痛) OR R=(心肌梗死 OR 心梗 OR 心肌梗塞 OR ACS OR STEMI OR NSTEMI OR 胸痹 OR 胸痹心痛 OR 真心痛 OR 卒心痛 OR 心痛 OR 猝心痛 OR 厥心痛)) AND (M=(PCI OR PTCA OR 经皮冠状动脉介入治疗术 OR 经皮冠状动脉介入 OR 经皮冠状动脉 OR 冠脉介入 OR 经皮冠脉 OR 血管成形术 OR 支架置入术 OR 冠状动脉成形术 OR 球囊 OR 支架) OR R=(PCI OR PTCA OR 经皮冠状动脉介入治疗术 OR 经皮冠状动脉介入 OR 经皮冠状动脉 OR 冠脉介入 OR 经皮冠脉 OR 血管成形术 OR 支架置入术 OR 冠状动脉成形术 OR 球囊 OR 支架)))

Web of Science

#1 TS=(Kudiezi injection OR Ixeris sonchifolia extract injection OR Kudiezi OR Ixeris sonchifolia extract)

#2 TS=(myocardial infarct* OR heart infarct* OR cardiac infarct* OR cardiovascular stroke* OR cardiogenic shock OR heart attack* OR ACS OR STEMI OR NSTEMI OR ST-segment elevation myocardial infarction OR Non-ST-segment elevation myocardial infarction OR MINOCA OR Myocardial infarction with no obstructive coronary atherosclerosis OR xiongbi OR xiongbixintong OR xiongbi Xintong OR zhenxintong OR cuxintong OR Xintong OR juexintong OR cardiovascular shock OR cardiogenic stroke*)

#3 TS=(percutaneous coronary intervention OR percutaneous coronary intervention* OR percutaneous coronary revascularization* OR coronary atherectom* OR transluminal coronary angioplast* OR transluminal coronary balloon dilation OR coronary balloon angioplast* OR PCI OR Percutaneous Transluminal Coronary Angioplast* OR PTCA OR Rotational Atherectom* OR Directional Coronary Atherectom* OR Balloon dilation OR Artery Stenting)

#4 #2 AND #3

#5 #1 AND #4

PubMed

#1 “Kudiezi injection”[Title/Abstract] OR “Ixeris sonchifolia extract injection”[Title/Abstract] OR “Kudiezi”[Title/Abstract] OR “Ixeris sonchifolia extract”[Title/Abstract]

#2 "myocardial infarction"[MeSH Terms] OR “Acute Coronary Syndrome”[MeSH Terms] OR "myocardial infarct*"[Title/Abstract] OR “Acute Coronary Syndrome”[Title/Abstract] OR “ACS”[Title/Abstract] OR “Acute Coronary Syndrome*”[Title/Abstract]OR “NSTE-ACS”[Title/Abstract] OR “Non–ST-Segment Elevation Acute Coronary Syndrome*”[Title/Abstract] OR "heart infarct*"[Title/Abstract] OR "cardiac infarct*"[Title/Abstract] OR "cardiovascular stroke*"[Title/Abstract] OR "cardiogenic shock"[Title/Abstract] OR “cardiovascular shock” [Title/Abstract] OR “cardiogenic stroke*” [Title/Abstract] OR "heart attack*"[Title/Abstract] OR "STEMI"[Title/Abstract] OR "NSTEMI"[Title/Abstract] OR “ST-segment elevation myocardial infarction”[Title/Abstract] OR “Non-ST-segment elevation myocardial infarction”[Title/Abstract] OR “MINOCA”[Title/Abstract] OR “Myocardial infarction with no obstructive coronary atherosclerosis” [Title/Abstract] OR “xiongbi” [Title/Abstract] OR “xiongbixintong” [Title/Abstract] OR “xiongbi xintong” [Title/Abstract] OR “zhenxintong” [Title/Abstract] OR “cuxintong” [Title/Abstract] OR “Xintong” [Title/Abstract] OR “juexintong” [Title/Abstract]

#3 “percutaneous coronary intervention” [MeSH Terms] OR “percutaneous coronary intervention*”[Title/Abstract] OR “percutaneous coronary revascularization*” [Title/Abstract] OR “coronary atherectom*”[Title/Abstract] OR “transluminal coronary angioplast*”[Title/Abstract] OR “transluminal coronary balloon dilation”[Title/Abstract] OR “coronary balloon angioplast*”[Title/Abstract] OR “PCI”[Title/Abstract] OR “Percutaneous Transluminal Coronary Angioplast*”[Title/Abstract] OR “PTCA”[Title/Abstract] OR “Rotational Atherectom*”[Title/Abstract] OR

“Directional Coronary Atherectom*”[Title/Abstract] OR “Balloon dilation”[Title/Abstract] OR “Artery Stenting”[Title/Abstract]

#4 #2 AND #3

#1 AND #4

Embase

#1 'Kudiezi injection':ab,ti OR ‘Ixeris sonchifolia extract injection’:ab,ti OR ‘Kudiezi’:ab,ti OR ‘Ixeris sonchifolia extract':ab,ti

#2 'heart infarction'/exp

#3 'Acute Coronary Syndrome'/exp

#4 'cardiac infarct':ab,ti OR 'cardiac infarction':ab,ti OR 'cardial infarct':ab,ti OR 'heart attack':ab,ti OR 'heart infarct':ab,ti OR 'heart micro infarction':ab,ti OR 'heart muscle infarction':ab,ti OR 'infarction, heart':ab,ti OR 'myocardial infarct':ab,ti OR 'myocardial infarction':ab,ti OR 'myocardium infarct':ab,ti OR 'myocardium infarction':ab,ti OR 'premonitory infarction sign':ab,ti OR 'second heart attack':ab,ti OR 'subendocardial infarction':ab,ti OR 'transmural cardiac infarction':ab,ti OR 'transmural heart infarction':ab,ti OR 'transmural infarction, heart':ab,ti OR 'heart infarction':ab,ti OR ‘Acute Coronary Syndrome’:ab,ti OR ‘ACS’:ab,ti OR ‘Acute Coronary Syndrome*’:ab,ti OR ‘NSTE-ACS’:ab,ti OR ‘Non–ST-Segment Elevation Acute Coronary Syndrome*’:ab,ti OR 'STEMI':ab,ti OR 'NSTEMI':ab,ti OR ‘ST-segment elevation myocardial infarction’:ab,ti OR ‘Non-ST-segment elevation myocardial infarction’:ab,ti OR ‘MINOCA’:ab,ti OR ‘Myocardial infarction with no obstructive coronary atherosclerosis’:ab,ti OR ‘xiongbi’:ab,ti OR ‘xiongbixintong’:ab,ti OR ‘xiongbi Xintong’:ab,ti OR ‘zhenxintong’:ab,ti OR ‘cuxintong’:ab,ti OR ‘Xintong’:ab,ti OR ‘juexintong’:ab,ti OR ‘cardiovascular shock’:ab,ti OR ‘cardiogenic stroke*’:ab,ti

#5 ‘percutaneous coronary intervention'/exp

#6 ‘percutaneous coronary intervention*':ab,ti OR 'percutaneous coronary revascularization*':ab,ti OR 'coronary atherectom*':ab,ti OR 'transluminal coronary angioplast*':ab,ti OR 'transluminal coronary balloon dilation':ab,ti OR 'coronary balloon angioplast*':ab,ti OR 'PCI':ab,ti OR 'Percutaneous Transluminal Coronary Angioplast*':ab,ti OR 'PTCA':ab,ti OR 'Rotational Atherectom*':ab,ti OR 'Directional Coronary Atherectom*':ab,ti OR 'Balloon dilation':ab,ti OR 'Artery Stenting’:ab,ti

#7 #2 OR #3

#8 #4 OR #7

#9 #5 OR #6

#10 #8 AND #9

#11 #1 AND #10

Cochrane

#1 (Kudiezi injection):ti,ab,kw OR (Kudiezi injection):ti,ab,kw OR (Kudiezi):ti,ab,kw OR (Ixeris sonchifolia extract ):ti,ab,kw

#2 MeSH descriptor: [Myocardial Infarction] explode all trees

#3 MeSH descriptor: [Acute Coronary Syndrome] explode all trees

#4 (myocardial infarct*):ti,ab,kw OR (heart infarct*):ti,ab,kw OR (cardiac infarct*):ti,ab,kw OR (cardiovascular stroke*):ti,ab,kw OR (cardiogenic shock):ti,ab,kw OR (heart attack*):ti,ab,kw OR (Acute Coronary Syndrome):ti,ab,kw OR (ACS):ti,ab,kw OR (Acute Coronary Syndrome*):ti,ab,kw OR (NSTE-ACS):ti,ab,kw OR (Non–ST-Segment Elevation Acute Coronary Syndrome*):ti,ab,kw OR (STEMI):ti,ab,kw OR (NSTEMI):ti,ab,kw OR (ST-segment elevation myocardial infarction):ti,ab,kw OR (Non-ST-segment elevation myocardial infarction):ti,ab,kw OR (MINOCA):ti,ab,kw OR (Myocardial infarction with no obstructive coronary atherosclerosis):ti,ab,kw OR (xiongbi):ti,ab,kw OR (xiongbixintong):ti,ab,kw OR (xiongbi Xintong):ti,ab,kw OR (zhenxintong):ti,ab,kw OR (cuxintong):ti,ab,kw OR (Xintong):ti,ab,kw OR (juexintong):ti,ab,kw OR (cardiovascular shock):ti,ab,kw OR (cardiogenic stroke*):ti,ab,kw

#5 MeSH descriptor: [Percutaneous Coronary Intervention] explode all trees

#6 (percutaneous coronary intervention):ti,ab,kw OR (percutaneous coronary intervention*):ti,ab,kw OR (percutaneous coronary revascularization*):ti,ab,kw OR (coronary atherectom*):ti,ab,kw OR (transluminal coronary angioplast*):ti,ab,kw OR (transluminal coronary balloon dilation):ti,ab,kw OR (coronary balloon angioplast*):ti,ab,kw OR (PCI):ti,ab,kw OR (Percutaneous Transluminal Coronary Angioplast*):ti,ab,kw OR (PTCA):ti,ab,kw OR (Rotational Atherectom*):ti,ab,kw OR (Directional Coronary Atherectom*):ti,ab,kw OR (Balloon dilation):ti,ab,kw OR (Artery Stenting):ti,ab,kw

#7 #2 OR #3

#8 #4 OR #7

#9 #5 OR #6

#10 #8 AND #9

#11 #1 AND #10

24.Salvianolate Injection

CNKI

(TKA=‘注射用丹参多酚酸盐’ + ‘丹参多酚酸盐’) and ((TKA=‘心肌梗死’ + ‘心梗’ + ‘心肌梗塞’ + ‘急性冠脉综合征’+ ‘ACS’ + ‘STEMI’ + ‘NSTEMI’ + ‘胸痹’ + ‘胸痹心痛’ + ‘真心痛’ + ‘卒心痛’ + ‘心痛’ + ‘猝心痛’ + ‘厥心痛’) and (TKA=‘PCI’ + ‘PTCA’ + ‘经皮冠状动脉介入治疗术’ + ‘经皮冠状动脉介入’ + ‘经皮冠状动脉’ + ‘冠脉介入’ + ‘经皮冠脉’ + ‘血管成形术’ + ‘支架置入术’ + ‘冠状动脉成形术’ + ‘球囊’ + ‘支架’))

WangFang Data

主题:(“注射用丹参多酚酸盐” OR “丹参多酚酸盐”) and ((主题:心肌梗死 OR 心梗 OR 心肌梗塞 OR 急性冠脉综合征 OR ACS OR STEMI OR NSTEMI OR 胸痹 OR 胸痹心痛 OR 真心痛 OR 卒心痛 OR 心痛 OR 猝心痛 OR 厥心痛) and 主题:(PCI OR PTCA OR 经皮冠状动脉介入治疗术 OR 经皮冠状动脉介入 OR 经皮冠状动脉 OR 冠脉介入 OR 经皮冠脉 OR 血管成形术 OR 支架置入术 OR 冠状动脉成形术 OR 球囊 OR 支架))

VIP

(M=(“注射用丹参多酚酸盐” OR “丹参多酚酸盐”) OR R=(“注射用丹参多酚酸盐” OR “丹参多酚酸盐”)) AND ((M=(心肌梗死 OR 心梗 OR 心肌梗塞 OR 急性冠脉综合征 OR STEMI OR NSTEMI OR 胸痹 OR 胸痹心痛 OR 真心痛 OR 卒心痛 OR 心痛 OR 猝心痛 OR 厥心痛) OR R=(心肌梗死 OR 心梗 OR 心肌梗塞 OR ACS OR STEMI OR NSTEMI OR 胸痹 OR 胸痹心痛 OR 真心痛 OR 卒心痛 OR 心痛 OR 猝心痛 OR 厥心痛)) AND (M=(PCI OR PTCA OR 经皮冠状动脉介入治疗术 OR 经皮冠状动脉介入 OR 经皮冠状动脉 OR 冠脉介入 OR 经皮冠脉 OR 血管成形术 OR 支架置入术 OR 冠状动脉成形术 OR 球囊 OR 支架) OR R=(PCI OR PTCA OR 经皮冠状动脉介入治疗术 OR 经皮冠状动脉介入 OR 经皮冠状动脉 OR 冠脉介入 OR 经皮冠脉 OR 血管成形术 OR 支架置入术 OR 冠状动脉成形术 OR 球囊 OR 支架)))

Web of Science

#1 TS=(Salvianolate injection OR salvianolic acids for injection OR Salvianolate OR salvianolic acids)

#2 TS=(myocardial infarct* OR heart infarct* OR cardiac infarct* OR cardiovascular stroke* OR cardiogenic shock OR heart attack* OR ACS OR STEMI OR NSTEMI OR ST-segment elevation myocardial infarction OR Non-ST-segment elevation myocardial infarction OR MINOCA OR Myocardial infarction with no obstructive coronary atherosclerosis OR xiongbi OR xiongbixintong OR xiongbi Xintong OR zhenxintong OR cuxintong OR Xintong OR juexintong OR cardiovascular shock OR cardiogenic stroke*)

#3 TS=(percutaneous coronary intervention OR percutaneous coronary intervention* OR percutaneous coronary revascularization* OR coronary atherectom* OR transluminal coronary angioplast* OR transluminal coronary balloon dilation OR coronary balloon angioplast* OR PCI OR Percutaneous Transluminal Coronary Angioplast* OR PTCA OR Rotational Atherectom* OR Directional Coronary Atherectom* OR Balloon dilation OR Artery Stenting)

#4 #2 AND #3

#5 #1 AND #4

PubMed

#1 “Salvianolate injection”[Title/Abstract] OR “salvianolic acids for injection”[Title/Abstract] OR “Salvianolate”[Title/Abstract] OR “salvianolic acids”[Title/Abstract]

#2 "myocardial infarction"[MeSH Terms] OR “Acute Coronary Syndrome”[MeSH Terms] OR "myocardial infarct*"[Title/Abstract] OR “Acute Coronary Syndrome”[Title/Abstract] OR “ACS”[Title/Abstract] OR “Acute Coronary Syndrome*”[Title/Abstract]OR “NSTE-ACS”[Title/Abstract] OR “Non–ST-Segment Elevation Acute Coronary Syndrome*”[Title/Abstract] OR "heart infarct*"[Title/Abstract] OR "cardiac infarct*"[Title/Abstract] OR "cardiovascular stroke*"[Title/Abstract] OR "cardiogenic shock"[Title/Abstract] OR “cardiovascular shock” [Title/Abstract] OR “cardiogenic stroke*” [Title/Abstract] OR "heart attack*"[Title/Abstract] OR "STEMI"[Title/Abstract] OR "NSTEMI"[Title/Abstract] OR “ST-segment elevation myocardial infarction”[Title/Abstract] OR “Non-ST-segment elevation myocardial infarction”[Title/Abstract] OR “MINOCA”[Title/Abstract] OR “Myocardial infarction with no obstructive coronary atherosclerosis” [Title/Abstract] OR “xiongbi” [Title/Abstract] OR “xiongbixintong” [Title/Abstract] OR “xiongbi xintong” [Title/Abstract] OR “zhenxintong” [Title/Abstract] OR “cuxintong” [Title/Abstract] OR “Xintong” [Title/Abstract] OR “juexintong” [Title/Abstract]

#3 “percutaneous coronary intervention” [MeSH Terms] OR “percutaneous coronary intervention*”[Title/Abstract] OR “percutaneous coronary revascularization*” [Title/Abstract] OR “coronary atherectom*”[Title/Abstract] OR “transluminal coronary angioplast*”[Title/Abstract] OR “transluminal coronary balloon dilation”[Title/Abstract] OR “coronary balloon angioplast*”[Title/Abstract] OR “PCI”[Title/Abstract] OR “Percutaneous Transluminal Coronary Angioplast*”[Title/Abstract] OR “PTCA”[Title/Abstract] OR “Rotational Atherectom*”[Title/Abstract] OR

“Directional Coronary Atherectom*”[Title/Abstract] OR “Balloon dilation”[Title/Abstract] OR “Artery Stenting”[Title/Abstract]

#4 #2 AND #3

#1 AND #4

Embase

#1 'Salvianolate injection':ab,ti OR ‘salvianolic acids for injection’:ab,ti OR ‘Salvianolate’:ab,ti OR ‘salvianolic acids':ab,ti

#2 'heart infarction'/exp

#3 'Acute Coronary Syndrome'/exp

#4 'cardiac infarct':ab,ti OR 'cardiac infarction':ab,ti OR 'cardial infarct':ab,ti OR 'heart attack':ab,ti OR 'heart infarct':ab,ti OR 'heart micro infarction':ab,ti OR 'heart muscle infarction':ab,ti OR 'infarction, heart':ab,ti OR 'myocardial infarct':ab,ti OR 'myocardial infarction':ab,ti OR 'myocardium infarct':ab,ti OR 'myocardium infarction':ab,ti OR 'premonitory infarction sign':ab,ti OR 'second heart attack':ab,ti OR 'subendocardial infarction':ab,ti OR 'transmural cardiac infarction':ab,ti OR 'transmural heart infarction':ab,ti OR 'transmural infarction, heart':ab,ti OR 'heart infarction':ab,ti OR ‘Acute Coronary Syndrome’:ab,ti OR ‘ACS’:ab,ti OR ‘Acute Coronary Syndrome*’:ab,ti OR ‘NSTE-ACS’:ab,ti OR ‘Non–ST-Segment Elevation Acute Coronary Syndrome*’:ab,ti OR 'STEMI':ab,ti OR 'NSTEMI':ab,ti OR ‘ST-segment elevation myocardial infarction’:ab,ti OR ‘Non-ST-segment elevation myocardial infarction’:ab,ti OR ‘MINOCA’:ab,ti OR ‘Myocardial infarction with no obstructive coronary atherosclerosis’:ab,ti OR ‘xiongbi’:ab,ti OR ‘xiongbixintong’:ab,ti OR ‘xiongbi Xintong’:ab,ti OR ‘zhenxintong’:ab,ti OR ‘cuxintong’:ab,ti OR ‘Xintong’:ab,ti OR ‘juexintong’:ab,ti OR ‘cardiovascular shock’:ab,ti OR ‘cardiogenic stroke*’:ab,ti

#5 ‘percutaneous coronary intervention'/exp

#6 ‘percutaneous coronary intervention*':ab,ti OR 'percutaneous coronary revascularization*':ab,ti OR 'coronary atherectom*':ab,ti OR 'transluminal coronary angioplast*':ab,ti OR 'transluminal coronary balloon dilation':ab,ti OR 'coronary balloon angioplast*':ab,ti OR 'PCI':ab,ti OR 'Percutaneous Transluminal Coronary Angioplast*':ab,ti OR 'PTCA':ab,ti OR 'Rotational Atherectom*':ab,ti OR 'Directional Coronary Atherectom*':ab,ti OR 'Balloon dilation':ab,ti OR 'Artery Stenting’:ab,ti

#7 #2 OR #3

#8 #4 OR #7

#9 #5 OR #6

#10 #8 AND #9

#11 #1 AND #10

Cochrane

#1 (Salvianolate injection):ti,ab,kw OR (salvianolic acids for injection):ti,ab,kw OR (Salvianolate):ti,ab,kw OR (salvianolic acids):ti,ab,kw

#2 MeSH descriptor: [Myocardial Infarction] explode all trees

#3 MeSH descriptor: [Acute Coronary Syndrome] explode all trees

#4 (myocardial infarct*):ti,ab,kw OR (heart infarct*):ti,ab,kw OR (cardiac infarct*):ti,ab,kw OR (cardiovascular stroke*):ti,ab,kw OR (cardiogenic shock):ti,ab,kw OR (heart attack*):ti,ab,kw OR (Acute Coronary Syndrome):ti,ab,kw OR (ACS):ti,ab,kw OR (Acute Coronary Syndrome*):ti,ab,kw OR (NSTE-ACS):ti,ab,kw OR (Non–ST-Segment Elevation Acute Coronary Syndrome*):ti,ab,kw OR (STEMI):ti,ab,kw OR (NSTEMI):ti,ab,kw OR (ST-segment elevation myocardial infarction):ti,ab,kw OR (Non-ST-segment elevation myocardial infarction):ti,ab,kw OR (MINOCA):ti,ab,kw OR (Myocardial infarction with no obstructive coronary atherosclerosis):ti,ab,kw OR (xiongbi):ti,ab,kw OR (xiongbixintong):ti,ab,kw OR (xiongbi Xintong):ti,ab,kw OR (zhenxintong):ti,ab,kw OR (cuxintong):ti,ab,kw OR (Xintong):ti,ab,kw OR (juexintong):ti,ab,kw OR (cardiovascular shock):ti,ab,kw OR (cardiogenic stroke*):ti,ab,kw

#5 MeSH descriptor: [Percutaneous Coronary Intervention] explode all trees

#6 (percutaneous coronary intervention):ti,ab,kw OR (percutaneous coronary intervention*):ti,ab,kw OR (percutaneous coronary revascularization*):ti,ab,kw OR (coronary atherectom*):ti,ab,kw OR (transluminal coronary angioplast*):ti,ab,kw OR (transluminal coronary balloon dilation):ti,ab,kw OR (coronary balloon angioplast*):ti,ab,kw OR (PCI):ti,ab,kw OR (Percutaneous Transluminal Coronary Angioplast*):ti,ab,kw OR (PTCA):ti,ab,kw OR (Rotational Atherectom*):ti,ab,kw OR (Directional Coronary Atherectom*):ti,ab,kw OR (Balloon dilation):ti,ab,kw OR (Artery Stenting):ti,ab,kw

#7 #2 OR #3

#8 #4 OR #7

#9 #5 OR #6

#10 #8 AND #9

#11 #1 AND #10

25 breviscapine Injection

CNKI

(TKA=‘注射用灯盏花素’ + ‘灯盏花素’) and ((TKA=‘心肌梗死’ + ‘心梗’ + ‘心肌梗塞’ + ‘急性冠脉综合征’+ ‘ACS’ + ‘STEMI’ + ‘NSTEMI’ + ‘胸痹’ + ‘胸痹心痛’ + ‘真心痛’ + ‘卒心痛’ + ‘心痛’ + ‘猝心痛’ + ‘厥心痛’) and (TKA=‘PCI’ + ‘PTCA’ + ‘经皮冠状动脉介入治疗术’ + ‘经皮冠状动脉介入’ + ‘经皮冠状动脉’ + ‘冠脉介入’ + ‘经皮冠脉’ + ‘血管成形术’ + ‘支架置入术’ + ‘冠状动脉成形术’ + ‘球囊’ + ‘支架’))

WangFang Data

主题:(“注射用灯盏花素” OR “灯盏花素”) and ((主题:心肌梗死 OR 心梗 OR 心肌梗塞 OR 急性冠脉综合征 OR ACS OR STEMI OR NSTEMI OR 胸痹 OR 胸痹心痛 OR 真心痛 OR 卒心痛 OR 心痛 OR 猝心痛 OR 厥心痛) and 主题:(PCI OR PTCA OR 经皮冠状动脉介入治疗术 OR 经皮冠状动脉介入 OR 经皮冠状动脉 OR 冠脉介入 OR 经皮冠脉 OR 血管成形术 OR 支架置入术 OR 冠状动脉成形术 OR 球囊 OR 支架))

VIP

(M=(“注射用灯盏花素” OR “灯盏花素”) OR R=(“注射用灯盏花素” OR “灯盏花素”)) AND ((M=(心肌梗死 OR 心梗 OR 心肌梗塞 OR 急性冠脉综合征 OR STEMI OR NSTEMI OR 胸痹 OR 胸痹心痛 OR 真心痛 OR 卒心痛 OR 心痛 OR 猝心痛 OR 厥心痛) OR R=(心肌梗死 OR 心梗 OR 心肌梗塞 OR ACS OR STEMI OR NSTEMI OR 胸痹 OR 胸痹心痛 OR 真心痛 OR 卒心痛 OR 心痛 OR 猝心痛 OR 厥心痛)) AND (M=(PCI OR PTCA OR 经皮冠状动脉介入治疗术 OR 经皮冠状动脉介入 OR 经皮冠状动脉 OR 冠脉介入 OR 经皮冠脉 OR 血管成形术 OR 支架置入术 OR 冠状动脉成形术 OR 球囊 OR 支架) OR R=(PCI OR PTCA OR 经皮冠状动脉介入治疗术 OR 经皮冠状动脉介入 OR 经皮冠状动脉 OR 冠脉介入 OR 经皮冠脉 OR 血管成形术 OR 支架置入术 OR 冠状动脉成形术 OR 球囊 OR 支架)))

Web of Science

#1 TS=breviscapine

#2 TS=(myocardial infarct* OR heart infarct* OR cardiac infarct* OR cardiovascular stroke* OR cardiogenic shock OR heart attack* OR ACS OR STEMI OR NSTEMI OR ST-segment elevation myocardial infarction OR Non-ST-segment elevation myocardial infarction OR MINOCA OR Myocardial infarction with no obstructive coronary atherosclerosis OR xiongbi OR xiongbixintong OR xiongbi Xintong OR zhenxintong OR cuxintong OR Xintong OR juexintong OR cardiovascular shock OR cardiogenic stroke*)

#3 TS=(percutaneous coronary intervention OR percutaneous coronary intervention* OR percutaneous coronary revascularization* OR coronary atherectom* OR transluminal coronary angioplast* OR transluminal coronary balloon dilation OR coronary balloon angioplast* OR PCI OR Percutaneous Transluminal Coronary Angioplast* OR PTCA OR Rotational Atherectom* OR Directional Coronary Atherectom* OR Balloon dilation OR Artery Stenting)

#4 #2 AND #3

#5 #1 AND #4

PubMed

#1 “breviscapine”[Title/Abstract]

#2 "myocardial infarction"[MeSH Terms] OR “Acute Coronary Syndrome”[MeSH Terms] OR "myocardial infarct*"[Title/Abstract] OR “Acute Coronary Syndrome”[Title/Abstract] OR “ACS”[Title/Abstract] OR “Acute Coronary Syndrome*”[Title/Abstract]OR “NSTE-ACS”[Title/Abstract] OR “Non–ST-Segment Elevation Acute Coronary Syndrome*”[Title/Abstract] OR "heart infarct*"[Title/Abstract] OR "cardiac infarct*"[Title/Abstract] OR "cardiovascular stroke*"[Title/Abstract] OR "cardiogenic shock"[Title/Abstract] OR “cardiovascular shock” [Title/Abstract] OR “cardiogenic stroke*” [Title/Abstract] OR "heart attack*"[Title/Abstract] OR "STEMI"[Title/Abstract] OR "NSTEMI"[Title/Abstract] OR “ST-segment elevation myocardial infarction”[Title/Abstract] OR “Non-ST-segment elevation myocardial infarction”[Title/Abstract] OR “MINOCA”[Title/Abstract] OR “Myocardial infarction with no obstructive coronary atherosclerosis” [Title/Abstract] OR “xiongbi” [Title/Abstract] OR “xiongbixintong” [Title/Abstract] OR “xiongbi xintong” [Title/Abstract] OR “zhenxintong” [Title/Abstract] OR “cuxintong” [Title/Abstract] OR “Xintong” [Title/Abstract] OR “juexintong” [Title/Abstract]

#3 “percutaneous coronary intervention” [MeSH Terms] OR “percutaneous coronary intervention*”[Title/Abstract] OR “percutaneous coronary revascularization*” [Title/Abstract] OR “coronary atherectom*”[Title/Abstract] OR “transluminal coronary angioplast*”[Title/Abstract] OR “transluminal coronary balloon dilation”[Title/Abstract] OR “coronary balloon angioplast*”[Title/Abstract] OR “PCI”[Title/Abstract] OR “Percutaneous Transluminal Coronary Angioplast*”[Title/Abstract] OR “PTCA”[Title/Abstract] OR “Rotational Atherectom*”[Title/Abstract] OR

“Directional Coronary Atherectom*”[Title/Abstract] OR “Balloon dilation”[Title/Abstract] OR “Artery Stenting”[Title/Abstract]

#4 #2 AND #3

#1 AND #4

Embase

#1 'breviscapine':ab,ti

#2 'heart infarction'/exp

#3 'Acute Coronary Syndrome'/exp

#4 'cardiac infarct':ab,ti OR 'cardiac infarction':ab,ti OR 'cardial infarct':ab,ti OR 'heart attack':ab,ti OR 'heart infarct':ab,ti OR 'heart micro infarction':ab,ti OR 'heart muscle infarction':ab,ti OR 'infarction, heart':ab,ti OR 'myocardial infarct':ab,ti OR 'myocardial infarction':ab,ti OR 'myocardium infarct':ab,ti OR 'myocardium infarction':ab,ti OR 'premonitory infarction sign':ab,ti OR 'second heart attack':ab,ti OR 'subendocardial infarction':ab,ti OR 'transmural cardiac infarction':ab,ti OR 'transmural heart infarction':ab,ti OR 'transmural infarction, heart':ab,ti OR 'heart infarction':ab,ti OR ‘Acute Coronary Syndrome’:ab,ti OR ‘ACS’:ab,ti OR ‘Acute Coronary Syndrome*’:ab,ti OR ‘NSTE-ACS’:ab,ti OR ‘Non–ST-Segment Elevation Acute Coronary Syndrome*’:ab,ti OR 'STEMI':ab,ti OR 'NSTEMI':ab,ti OR ‘ST-segment elevation myocardial infarction’:ab,ti OR ‘Non-ST-segment elevation myocardial infarction’:ab,ti OR ‘MINOCA’:ab,ti OR ‘Myocardial infarction with no obstructive coronary atherosclerosis’:ab,ti OR ‘xiongbi’:ab,ti OR ‘xiongbixintong’:ab,ti OR ‘xiongbi Xintong’:ab,ti OR ‘zhenxintong’:ab,ti OR ‘cuxintong’:ab,ti OR ‘Xintong’:ab,ti OR ‘juexintong’:ab,ti OR ‘cardiovascular shock’:ab,ti OR ‘cardiogenic stroke*’:ab,ti

#5 ‘percutaneous coronary intervention'/exp

#6 ‘percutaneous coronary intervention*':ab,ti OR 'percutaneous coronary revascularization*':ab,ti OR 'coronary atherectom*':ab,ti OR 'transluminal coronary angioplast*':ab,ti OR 'transluminal coronary balloon dilation':ab,ti OR 'coronary balloon angioplast*':ab,ti OR 'PCI':ab,ti OR 'Percutaneous Transluminal Coronary Angioplast*':ab,ti OR 'PTCA':ab,ti OR 'Rotational Atherectom*':ab,ti OR 'Directional Coronary Atherectom*':ab,ti OR 'Balloon dilation':ab,ti OR 'Artery Stenting’:ab,ti

#7 #2 OR #3

#8 #4 OR #7

#9 #5 OR #6

#10 #8 AND #9

#11 #1 AND #10

Cochrane

#1 (breviscapine):ti,ab,kw

#2 MeSH descriptor: [Myocardial Infarction] explode all trees

#3 MeSH descriptor: [Acute Coronary Syndrome] explode all trees

#4 (myocardial infarct*):ti,ab,kw OR (heart infarct*):ti,ab,kw OR (cardiac infarct*):ti,ab,kw OR (cardiovascular stroke*):ti,ab,kw OR (cardiogenic shock):ti,ab,kw OR (heart attack*):ti,ab,kw OR (Acute Coronary Syndrome):ti,ab,kw OR (ACS):ti,ab,kw OR (Acute Coronary Syndrome*):ti,ab,kw OR (NSTE-ACS):ti,ab,kw OR (Non–ST-Segment Elevation Acute Coronary Syndrome*):ti,ab,kw OR (STEMI):ti,ab,kw OR (NSTEMI):ti,ab,kw OR (ST-segment elevation myocardial infarction):ti,ab,kw OR (Non-ST-segment elevation myocardial infarction):ti,ab,kw OR (MINOCA):ti,ab,kw OR (Myocardial infarction with no obstructive coronary atherosclerosis):ti,ab,kw OR (xiongbi):ti,ab,kw OR (xiongbixintong):ti,ab,kw OR (xiongbi Xintong):ti,ab,kw OR (zhenxintong):ti,ab,kw OR (cuxintong):ti,ab,kw OR (Xintong):ti,ab,kw OR (juexintong):ti,ab,kw OR (cardiovascular shock):ti,ab,kw OR (cardiogenic stroke*):ti,ab,kw

#5 MeSH descriptor: [Percutaneous Coronary Intervention] explode all trees

#6 (percutaneous coronary intervention):ti,ab,kw OR (percutaneous coronary intervention*):ti,ab,kw OR (percutaneous coronary revascularization*):ti,ab,kw OR (coronary atherectom*):ti,ab,kw OR (transluminal coronary angioplast*):ti,ab,kw OR (transluminal coronary balloon dilation):ti,ab,kw OR (coronary balloon angioplast*):ti,ab,kw OR (PCI):ti,ab,kw OR (Percutaneous Transluminal Coronary Angioplast*):ti,ab,kw OR (PTCA):ti,ab,kw OR (Rotational Atherectom*):ti,ab,kw OR (Directional Coronary Atherectom*):ti,ab,kw OR (Balloon dilation):ti,ab,kw OR (Artery Stenting):ti,ab,kw

#7 #2 OR #3

#8 #4 OR #7

#9 #5 OR #6

#10 #8 AND #9

#11 #1 AND #10

26.Safflower yellow pigment for Injection

CNKI

(TKA=‘注射用红花黄色素’ + ‘红花黄色素’) and ((TKA=‘心肌梗死’ + ‘心梗’ + ‘心肌梗塞’ + ‘急性冠脉综合征’+ ‘ACS’ + ‘STEMI’ + ‘NSTEMI’ + ‘胸痹’ + ‘胸痹心痛’ + ‘真心痛’ + ‘卒心痛’ + ‘心痛’ + ‘猝心痛’ + ‘厥心痛’) and (TKA=‘PCI’ + ‘PTCA’ + ‘经皮冠状动脉介入治疗术’ + ‘经皮冠状动脉介入’ + ‘经皮冠状动脉’ + ‘冠脉介入’ + ‘经皮冠脉’ + ‘血管成形术’ + ‘支架置入术’ + ‘冠状动脉成形术’ + ‘球囊’ + ‘支架’))

WangFang Data

主题:(“注射用红花黄色素” OR “红花黄色素”) and ((主题:心肌梗死 OR 心梗 OR 心肌梗塞 OR 急性冠脉综合征 OR ACS OR STEMI OR NSTEMI OR 胸痹 OR 胸痹心痛 OR 真心痛 OR 卒心痛 OR 心痛 OR 猝心痛 OR 厥心痛) and 主题:(PCI OR PTCA OR 经皮冠状动脉介入治疗术 OR 经皮冠状动脉介入 OR 经皮冠状动脉 OR 冠脉介入 OR 经皮冠脉 OR 血管成形术 OR 支架置入术 OR 冠状动脉成形术 OR 球囊 OR 支架))

VIP

(M=(“注射用红花黄色素” OR “红花黄色素”) OR R=(“注射用红花黄色素” OR “红花黄色素”)) AND ((M=(心肌梗死 OR 心梗 OR 心肌梗塞 OR 急性冠脉综合征 OR STEMI OR NSTEMI OR 胸痹 OR 胸痹心痛 OR 真心痛 OR 卒心痛 OR 心痛 OR 猝心痛 OR 厥心痛) OR R=(心肌梗死 OR 心梗 OR 心肌梗塞 OR ACS OR STEMI OR NSTEMI OR 胸痹 OR 胸痹心痛 OR 真心痛 OR 卒心痛 OR 心痛 OR 猝心痛 OR 厥心痛)) AND (M=(PCI OR PTCA OR 经皮冠状动脉介入治疗术 OR 经皮冠状动脉介入 OR 经皮冠状动脉 OR 冠脉介入 OR 经皮冠脉 OR 血管成形术 OR 支架置入术 OR 冠状动脉成形术 OR 球囊 OR 支架) OR R=(PCI OR PTCA OR 经皮冠状动脉介入治疗术 OR 经皮冠状动脉介入 OR 经皮冠状动脉 OR 冠脉介入 OR 经皮冠脉 OR 血管成形术 OR 支架置入术 OR 冠状动脉成形术 OR 球囊 OR 支架)))

Web of Science

#1 TS=(Safflower yellow pigment for injection OR Safflower yellow for injection OR Safflower yellow pigment OR Safflower yellow)

#2 TS=(myocardial infarct* OR heart infarct* OR cardiac infarct* OR cardiovascular stroke* OR cardiogenic shock OR heart attack* OR ACS OR STEMI OR NSTEMI OR ST-segment elevation myocardial infarction OR Non-ST-segment elevation myocardial infarction OR MINOCA OR Myocardial infarction with no obstructive coronary atherosclerosis OR xiongbi OR xiongbixintong OR xiongbi Xintong OR zhenxintong OR cuxintong OR Xintong OR juexintong OR cardiovascular shock OR cardiogenic stroke*)

#3 TS=(percutaneous coronary intervention OR percutaneous coronary intervention* OR percutaneous coronary revascularization* OR coronary atherectom* OR transluminal coronary angioplast* OR transluminal coronary balloon dilation OR coronary balloon angioplast* OR PCI OR Percutaneous Transluminal Coronary Angioplast* OR PTCA OR Rotational Atherectom* OR Directional Coronary Atherectom* OR Balloon dilation OR Artery Stenting)

#4 #2 AND #3

#5 #1 AND #4

PubMed

#1 “Safflower yellow pigment for injection”[Title/Abstract] OR “Safflower yellow for injection”[Title/Abstract] OR “Safflower yellow pigment”[Title/Abstract] OR “Safflower yellow”[Title/Abstract]

#2 "myocardial infarction"[MeSH Terms] OR “Acute Coronary Syndrome”[MeSH Terms] OR "myocardial infarct*"[Title/Abstract] OR “Acute Coronary Syndrome”[Title/Abstract] OR “ACS”[Title/Abstract] OR “Acute Coronary Syndrome*”[Title/Abstract]OR “NSTE-ACS”[Title/Abstract] OR “Non–ST-Segment Elevation Acute Coronary Syndrome*”[Title/Abstract] OR "heart infarct*"[Title/Abstract] OR "cardiac infarct*"[Title/Abstract] OR "cardiovascular stroke*"[Title/Abstract] OR "cardiogenic shock"[Title/Abstract] OR “cardiovascular shock” [Title/Abstract] OR “cardiogenic stroke*” [Title/Abstract] OR "heart attack*"[Title/Abstract] OR "STEMI"[Title/Abstract] OR "NSTEMI"[Title/Abstract] OR “ST-segment elevation myocardial infarction”[Title/Abstract] OR “Non-ST-segment elevation myocardial infarction”[Title/Abstract] OR “MINOCA”[Title/Abstract] OR “Myocardial infarction with no obstructive coronary atherosclerosis” [Title/Abstract] OR “xiongbi” [Title/Abstract] OR “xiongbixintong” [Title/Abstract] OR “xiongbi xintong” [Title/Abstract] OR “zhenxintong” [Title/Abstract] OR “cuxintong” [Title/Abstract] OR “Xintong” [Title/Abstract] OR “juexintong” [Title/Abstract]

#3 “percutaneous coronary intervention” [MeSH Terms] OR “percutaneous coronary intervention*”[Title/Abstract] OR “percutaneous coronary revascularization*” [Title/Abstract] OR “coronary atherectom*”[Title/Abstract] OR “transluminal coronary angioplast*”[Title/Abstract] OR “transluminal coronary balloon dilation”[Title/Abstract] OR “coronary balloon angioplast*”[Title/Abstract] OR “PCI”[Title/Abstract] OR “Percutaneous Transluminal Coronary Angioplast*”[Title/Abstract] OR “PTCA”[Title/Abstract] OR “Rotational Atherectom*”[Title/Abstract] OR

“Directional Coronary Atherectom*”[Title/Abstract] OR “Balloon dilation”[Title/Abstract] OR “Artery Stenting”[Title/Abstract]

#4 #2 AND #3

#1 AND #4

Embase

#1 'Safflower yellow pigment for injection':ab,ti OR ‘Safflower yellow for injection’:ab,ti OR ‘Safflower yellow pigment’:ab,ti OR ‘Safflower yellow':ab,ti

#2 'heart infarction'/exp

#3 'Acute Coronary Syndrome'/exp

#4 'cardiac infarct':ab,ti OR 'cardiac infarction':ab,ti OR 'cardial infarct':ab,ti OR 'heart attack':ab,ti OR 'heart infarct':ab,ti OR 'heart micro infarction':ab,ti OR 'heart muscle infarction':ab,ti OR 'infarction, heart':ab,ti OR 'myocardial infarct':ab,ti OR 'myocardial infarction':ab,ti OR 'myocardium infarct':ab,ti OR 'myocardium infarction':ab,ti OR 'premonitory infarction sign':ab,ti OR 'second heart attack':ab,ti OR 'subendocardial infarction':ab,ti OR 'transmural cardiac infarction':ab,ti OR 'transmural heart infarction':ab,ti OR 'transmural infarction, heart':ab,ti OR 'heart infarction':ab,ti OR ‘Acute Coronary Syndrome’:ab,ti OR ‘ACS’:ab,ti OR ‘Acute Coronary Syndrome*’:ab,ti OR ‘NSTE-ACS’:ab,ti OR ‘Non–ST-Segment Elevation Acute Coronary Syndrome*’:ab,ti OR 'STEMI':ab,ti OR 'NSTEMI':ab,ti OR ‘ST-segment elevation myocardial infarction’:ab,ti OR ‘Non-ST-segment elevation myocardial infarction’:ab,ti OR ‘MINOCA’:ab,ti OR ‘Myocardial infarction with no obstructive coronary atherosclerosis’:ab,ti OR ‘xiongbi’:ab,ti OR ‘xiongbixintong’:ab,ti OR ‘xiongbi Xintong’:ab,ti OR ‘zhenxintong’:ab,ti OR ‘cuxintong’:ab,ti OR ‘Xintong’:ab,ti OR ‘juexintong’:ab,ti OR ‘cardiovascular shock’:ab,ti OR ‘cardiogenic stroke*’:ab,ti

#5 ‘percutaneous coronary intervention'/exp

#6 ‘percutaneous coronary intervention*':ab,ti OR 'percutaneous coronary revascularization*':ab,ti OR 'coronary atherectom*':ab,ti OR 'transluminal coronary angioplast*':ab,ti OR 'transluminal coronary balloon dilation':ab,ti OR 'coronary balloon angioplast*':ab,ti OR 'PCI':ab,ti OR 'Percutaneous Transluminal Coronary Angioplast*':ab,ti OR 'PTCA':ab,ti OR 'Rotational Atherectom*':ab,ti OR 'Directional Coronary Atherectom*':ab,ti OR 'Balloon dilation':ab,ti OR 'Artery Stenting’:ab,ti

#7 #2 OR #3

#8 #4 OR #7

#9 #5 OR #6

#10 #8 AND #9

#11 #1 AND #10

Cochrane

#1 (Safflower yellow pigment for injection):ti,ab,kw OR (Safflower yellow for injection):ti,ab,kw OR (Safflower yellow pigment):ti,ab,kw OR (Safflower yellow):ti,ab,kw

#2 MeSH descriptor: [Myocardial Infarction] explode all trees

#3 MeSH descriptor: [Acute Coronary Syndrome] explode all trees

#4 (myocardial infarct*):ti,ab,kw OR (heart infarct*):ti,ab,kw OR (cardiac infarct*):ti,ab,kw OR (cardiovascular stroke*):ti,ab,kw OR (cardiogenic shock):ti,ab,kw OR (heart attack*):ti,ab,kw OR (Acute Coronary Syndrome):ti,ab,kw OR (ACS):ti,ab,kw OR (Acute Coronary Syndrome*):ti,ab,kw OR (NSTE-ACS):ti,ab,kw OR (Non–ST-Segment Elevation Acute Coronary Syndrome*):ti,ab,kw OR (STEMI):ti,ab,kw OR (NSTEMI):ti,ab,kw OR (ST-segment elevation myocardial infarction):ti,ab,kw OR (Non-ST-segment elevation myocardial infarction):ti,ab,kw OR (MINOCA):ti,ab,kw OR (Myocardial infarction with no obstructive coronary atherosclerosis):ti,ab,kw OR (xiongbi):ti,ab,kw OR (xiongbixintong):ti,ab,kw OR (xiongbi Xintong):ti,ab,kw OR (zhenxintong):ti,ab,kw OR (cuxintong):ti,ab,kw OR (Xintong):ti,ab,kw OR (juexintong):ti,ab,kw OR (cardiovascular shock):ti,ab,kw OR (cardiogenic stroke*):ti,ab,kw

#5 MeSH descriptor: [Percutaneous Coronary Intervention] explode all trees

#6 (percutaneous coronary intervention):ti,ab,kw OR (percutaneous coronary intervention*):ti,ab,kw OR (percutaneous coronary revascularization*):ti,ab,kw OR (coronary atherectom*):ti,ab,kw OR (transluminal coronary angioplast*):ti,ab,kw OR (transluminal coronary balloon dilation):ti,ab,kw OR (coronary balloon angioplast*):ti,ab,kw OR (PCI):ti,ab,kw OR (Percutaneous Transluminal Coronary Angioplast*):ti,ab,kw OR (PTCA):ti,ab,kw OR (Rotational Atherectom*):ti,ab,kw OR (Directional Coronary Atherectom*):ti,ab,kw OR (Balloon dilation):ti,ab,kw OR (Artery Stenting):ti,ab,kw

#7 #2 OR #3

#8 #4 OR #7

#9 #5 OR #6

#10 #8 AND #9

#11 #1 AND #10

27.Xiangdan Injection

CNKI

(TKA=‘香丹注射液’ + ‘香丹’) and ((TKA=‘心肌梗死’ + ‘心梗’ + ‘心肌梗塞’ + ‘急性冠脉综合征’+ ‘ACS’ + ‘STEMI’ + ‘NSTEMI’ + ‘胸痹’ + ‘胸痹心痛’ + ‘真心痛’ + ‘卒心痛’ + ‘心痛’ + ‘猝心痛’ + ‘厥心痛’) and (TKA=‘PCI’ + ‘PTCA’ + ‘经皮冠状动脉介入治疗术’ + ‘经皮冠状动脉介入’ + ‘经皮冠状动脉’ + ‘冠脉介入’ + ‘经皮冠脉’ + ‘血管成形术’ + ‘支架置入术’ + ‘冠状动脉成形术’ + ‘球囊’ + ‘支架’))

WangFang Data

主题:(“香丹注射液” OR “香丹”) and ((主题:心肌梗死 OR 心梗 OR 心肌梗塞 OR 急性冠脉综合征 OR ACS OR STEMI OR NSTEMI OR 胸痹 OR 胸痹心痛 OR 真心痛 OR 卒心痛 OR 心痛 OR 猝心痛 OR 厥心痛) and 主题:(PCI OR PTCA OR 经皮冠状动脉介入治疗术 OR 经皮冠状动脉介入 OR 经皮冠状动脉 OR 冠脉介入 OR 经皮冠脉 OR 血管成形术 OR 支架置入术 OR 冠状动脉成形术 OR 球囊 OR 支架))

VIP

(M=(“香丹注射液” OR “香丹”) OR R=(“香丹注射液” OR “香丹”)) AND ((M=(心肌梗死 OR 心梗 OR 心肌梗塞 OR 急性冠脉综合征 OR STEMI OR NSTEMI OR 胸痹 OR 胸痹心痛 OR 真心痛 OR 卒心痛 OR 心痛 OR 猝心痛 OR 厥心痛) OR R=(心肌梗死 OR 心梗 OR 心肌梗塞 OR ACS OR STEMI OR NSTEMI OR 胸痹 OR 胸痹心痛 OR 真心痛 OR 卒心痛 OR 心痛 OR 猝心痛 OR 厥心痛)) AND (M=(PCI OR PTCA OR 经皮冠状动脉介入治疗术 OR 经皮冠状动脉介入 OR 经皮冠状动脉 OR 冠脉介入 OR 经皮冠脉 OR 血管成形术 OR 支架置入术 OR 冠状动脉成形术 OR 球囊 OR 支架) OR R=(PCI OR PTCA OR 经皮冠状动脉介入治疗术 OR 经皮冠状动脉介入 OR 经皮冠状动脉 OR 冠脉介入 OR 经皮冠脉 OR 血管成形术 OR 支架置入术 OR 冠状动脉成形术 OR 球囊 OR 支架)))

Web of Science

#1 TS=(Xiangdan injection OR xiangdan)

#2 TS=(myocardial infarct* OR heart infarct* OR cardiac infarct* OR cardiovascular stroke* OR cardiogenic shock OR heart attack* OR ACS OR STEMI OR NSTEMI OR ST-segment elevation myocardial infarction OR Non-ST-segment elevation myocardial infarction OR MINOCA OR Myocardial infarction with no obstructive coronary atherosclerosis OR xiongbi OR xiongbixintong OR xiongbi Xintong OR zhenxintong OR cuxintong OR Xintong OR juexintong OR cardiovascular shock OR cardiogenic stroke*)

#3 TS=(percutaneous coronary intervention OR percutaneous coronary intervention* OR percutaneous coronary revascularization* OR coronary atherectom* OR transluminal coronary angioplast* OR transluminal coronary balloon dilation OR coronary balloon angioplast* OR PCI OR Percutaneous Transluminal Coronary Angioplast* OR PTCA OR Rotational Atherectom* OR Directional Coronary Atherectom* OR Balloon dilation OR Artery Stenting)

#4 #2 AND #3

#5 #1 AND #4

PubMed

#1 “Xiangdan injection”[Title/Abstract] OR “xiangdan”[Title/Abstract]

#2 "myocardial infarction"[MeSH Terms] OR “Acute Coronary Syndrome”[MeSH Terms] OR "myocardial infarct*"[Title/Abstract] OR “Acute Coronary Syndrome”[Title/Abstract] OR “ACS”[Title/Abstract] OR “Acute Coronary Syndrome*”[Title/Abstract]OR “NSTE-ACS”[Title/Abstract] OR “Non–ST-Segment Elevation Acute Coronary Syndrome*”[Title/Abstract] OR "heart infarct*"[Title/Abstract] OR "cardiac infarct*"[Title/Abstract] OR "cardiovascular stroke*"[Title/Abstract] OR "cardiogenic shock"[Title/Abstract] OR “cardiovascular shock” [Title/Abstract] OR “cardiogenic stroke*” [Title/Abstract] OR "heart attack*"[Title/Abstract] OR "STEMI"[Title/Abstract] OR "NSTEMI"[Title/Abstract] OR “ST-segment elevation myocardial infarction”[Title/Abstract] OR “Non-ST-segment elevation myocardial infarction”[Title/Abstract] OR “MINOCA”[Title/Abstract] OR “Myocardial infarction with no obstructive coronary atherosclerosis” [Title/Abstract] OR “xiongbi” [Title/Abstract] OR “xiongbixintong” [Title/Abstract] OR “xiongbi xintong” [Title/Abstract] OR “zhenxintong” [Title/Abstract] OR “cuxintong” [Title/Abstract] OR “Xintong” [Title/Abstract] OR “juexintong” [Title/Abstract]

#3 “percutaneous coronary intervention” [MeSH Terms] OR “percutaneous coronary intervention*”[Title/Abstract] OR “percutaneous coronary revascularization*” [Title/Abstract] OR “coronary atherectom*”[Title/Abstract] OR “transluminal coronary angioplast*”[Title/Abstract] OR “transluminal coronary balloon dilation”[Title/Abstract] OR “coronary balloon angioplast*”[Title/Abstract] OR “PCI”[Title/Abstract] OR “Percutaneous Transluminal Coronary Angioplast*”[Title/Abstract] OR “PTCA”[Title/Abstract] OR “Rotational Atherectom*”[Title/Abstract] OR

“Directional Coronary Atherectom*”[Title/Abstract] OR “Balloon dilation”[Title/Abstract] OR “Artery Stenting”[Title/Abstract]

#4 #2 AND #3

#1 AND #4

Embase

#1 'Xiangdan injection':ab,ti OR ‘xiangdan’:ab,ti

#2 'heart infarction'/exp

#3 'Acute Coronary Syndrome'/exp

#4 'cardiac infarct':ab,ti OR 'cardiac infarction':ab,ti OR 'cardial infarct':ab,ti OR 'heart attack':ab,ti OR 'heart infarct':ab,ti OR 'heart micro infarction':ab,ti OR 'heart muscle infarction':ab,ti OR 'infarction, heart':ab,ti OR 'myocardial infarct':ab,ti OR 'myocardial infarction':ab,ti OR 'myocardium infarct':ab,ti OR 'myocardium infarction':ab,ti OR 'premonitory infarction sign':ab,ti OR 'second heart attack':ab,ti OR 'subendocardial infarction':ab,ti OR 'transmural cardiac infarction':ab,ti OR 'transmural heart infarction':ab,ti OR 'transmural infarction, heart':ab,ti OR 'heart infarction':ab,ti OR ‘Acute Coronary Syndrome’:ab,ti OR ‘ACS’:ab,ti OR ‘Acute Coronary Syndrome*’:ab,ti OR ‘NSTE-ACS’:ab,ti OR ‘Non–ST-Segment Elevation Acute Coronary Syndrome*’:ab,ti OR 'STEMI':ab,ti OR 'NSTEMI':ab,ti OR ‘ST-segment elevation myocardial infarction’:ab,ti OR ‘Non-ST-segment elevation myocardial infarction’:ab,ti OR ‘MINOCA’:ab,ti OR ‘Myocardial infarction with no obstructive coronary atherosclerosis’:ab,ti OR ‘xiongbi’:ab,ti OR ‘xiongbixintong’:ab,ti OR ‘xiongbi Xintong’:ab,ti OR ‘zhenxintong’:ab,ti OR ‘cuxintong’:ab,ti OR ‘Xintong’:ab,ti OR ‘juexintong’:ab,ti OR ‘cardiovascular shock’:ab,ti OR ‘cardiogenic stroke*’:ab,ti

#5 ‘percutaneous coronary intervention'/exp

#6 ‘percutaneous coronary intervention*':ab,ti OR 'percutaneous coronary revascularization*':ab,ti OR 'coronary atherectom*':ab,ti OR 'transluminal coronary angioplast*':ab,ti OR 'transluminal coronary balloon dilation':ab,ti OR 'coronary balloon angioplast*':ab,ti OR 'PCI':ab,ti OR 'Percutaneous Transluminal Coronary Angioplast*':ab,ti OR 'PTCA':ab,ti OR 'Rotational Atherectom*':ab,ti OR 'Directional Coronary Atherectom*':ab,ti OR 'Balloon dilation':ab,ti OR 'Artery Stenting’:ab,ti

#7 #2 OR #3

#8 #4 OR #7

#9 #5 OR #6

#10 #8 AND #9

#11 #1 AND #10

Cochrane

#1 (Xiangdan injection):ti,ab,kw OR (xiangdan):ti,ab,kw

#2 MeSH descriptor: [Myocardial Infarction] explode all trees

#3 MeSH descriptor: [Acute Coronary Syndrome] explode all trees

#4 (myocardial infarct*):ti,ab,kw OR (heart infarct*):ti,ab,kw OR (cardiac infarct*):ti,ab,kw OR (cardiovascular stroke*):ti,ab,kw OR (cardiogenic shock):ti,ab,kw OR (heart attack*):ti,ab,kw OR (Acute Coronary Syndrome):ti,ab,kw OR (ACS):ti,ab,kw OR (Acute Coronary Syndrome*):ti,ab,kw OR (NSTE-ACS):ti,ab,kw OR (Non–ST-Segment Elevation Acute Coronary Syndrome*):ti,ab,kw OR (STEMI):ti,ab,kw OR (NSTEMI):ti,ab,kw OR (ST-segment elevation myocardial infarction):ti,ab,kw OR (Non-ST-segment elevation myocardial infarction):ti,ab,kw OR (MINOCA):ti,ab,kw OR (Myocardial infarction with no obstructive coronary atherosclerosis):ti,ab,kw OR (xiongbi):ti,ab,kw OR (xiongbixintong):ti,ab,kw OR (xiongbi Xintong):ti,ab,kw OR (zhenxintong):ti,ab,kw OR (cuxintong):ti,ab,kw OR (Xintong):ti,ab,kw OR (juexintong):ti,ab,kw OR (cardiovascular shock):ti,ab,kw OR (cardiogenic stroke*):ti,ab,kw

#5 MeSH descriptor: [Percutaneous Coronary Intervention] explode all trees

#6 (percutaneous coronary intervention):ti,ab,kw OR (percutaneous coronary intervention*):ti,ab,kw OR (percutaneous coronary revascularization*):ti,ab,kw OR (coronary atherectom*):ti,ab,kw OR (transluminal coronary angioplast*):ti,ab,kw OR (transluminal coronary balloon dilation):ti,ab,kw OR (coronary balloon angioplast*):ti,ab,kw OR (PCI):ti,ab,kw OR (Percutaneous Transluminal Coronary Angioplast*):ti,ab,kw OR (PTCA):ti,ab,kw OR (Rotational Atherectom*):ti,ab,kw OR (Directional Coronary Atherectom*):ti,ab,kw OR (Balloon dilation):ti,ab,kw OR (Artery Stenting):ti,ab,kw

#7 #2 OR #3

#8 #4 OR #7

#9 #5 OR #6

#10 #8 AND #9

#11 #1 AND #10

28 Compound Danshen tablets

CNKI

(TKA=‘复方丹参片’ + ‘复方丹参’) and ((TKA=‘心肌梗死’ + ‘心梗’ + ‘心肌梗塞’ + ‘急性冠脉综合征’+ ‘ACS’ + ‘STEMI’ + ‘NSTEMI’ + ‘胸痹’ + ‘胸痹心痛’ + ‘真心痛’ + ‘卒心痛’ + ‘心痛’ + ‘猝心痛’ + ‘厥心痛’) and (TKA=‘PCI’ + ‘PTCA’ + ‘经皮冠状动脉介入治疗术’ + ‘经皮冠状动脉介入’ + ‘经皮冠状动脉’ + ‘冠脉介入’ + ‘经皮冠脉’ + ‘血管成形术’ + ‘支架置入术’ + ‘冠状动脉成形术’ + ‘球囊’ + ‘支架’))

WangFang Data

主题:(“复方丹参片” OR “复方丹参”) and ((主题:心肌梗死 OR 心梗 OR 心肌梗塞 OR 急性冠脉综合征 OR ACS OR STEMI OR NSTEMI OR 胸痹 OR 胸痹心痛 OR 真心痛 OR 卒心痛 OR 心痛 OR 猝心痛 OR 厥心痛) and 主题:(PCI OR PTCA OR 经皮冠状动脉介入治疗术 OR 经皮冠状动脉介入 OR 经皮冠状动脉 OR 冠脉介入 OR 经皮冠脉 OR 血管成形术 OR 支架置入术 OR 冠状动脉成形术 OR 球囊 OR 支架))

VIP

(M=(“复方丹参片” OR “复方丹参”) OR R=(“复方丹参片” OR “复方丹参”)) AND ((M=(心肌梗死 OR 心梗 OR 心肌梗塞 OR 急性冠脉综合征 OR STEMI OR NSTEMI OR 胸痹 OR 胸痹心痛 OR 真心痛 OR 卒心痛 OR 心痛 OR 猝心痛 OR 厥心痛) OR R=(心肌梗死 OR 心梗 OR 心肌梗塞 OR ACS OR STEMI OR NSTEMI OR 胸痹 OR 胸痹心痛 OR 真心痛 OR 卒心痛 OR 心痛 OR 猝心痛 OR 厥心痛)) AND (M=(PCI OR PTCA OR 经皮冠状动脉介入治疗术 OR 经皮冠状动脉介入 OR 经皮冠状动脉 OR 冠脉介入 OR 经皮冠脉 OR 血管成形术 OR 支架置入术 OR 冠状动脉成形术 OR 球囊 OR 支架) OR R=(PCI OR PTCA OR 经皮冠状动脉介入治疗术 OR 经皮冠状动脉介入 OR 经皮冠状动脉 OR 冠脉介入 OR 经皮冠脉 OR 血管成形术 OR 支架置入术 OR 冠状动脉成形术 OR 球囊 OR 支架)))

Web of Science

#1 TS=(Compound Danshen tablet* OR Compound Danshen)

#2 TS=(myocardial infarct* OR heart infarct* OR cardiac infarct* OR cardiovascular stroke* OR cardiogenic shock OR heart attack* OR ACS OR STEMI OR NSTEMI OR ST-segment elevation myocardial infarction OR Non-ST-segment elevation myocardial infarction OR MINOCA OR Myocardial infarction with no obstructive coronary atherosclerosis OR xiongbi OR xiongbixintong OR xiongbi Xintong OR zhenxintong OR cuxintong OR Xintong OR juexintong OR cardiovascular shock OR cardiogenic stroke*)

#3 TS=(percutaneous coronary intervention OR percutaneous coronary intervention* OR percutaneous coronary revascularization* OR coronary atherectom* OR transluminal coronary angioplast* OR transluminal coronary balloon dilation OR coronary balloon angioplast* OR PCI OR Percutaneous Transluminal Coronary Angioplast* OR PTCA OR Rotational Atherectom* OR Directional Coronary Atherectom* OR Balloon dilation OR Artery Stenting)

#4 #2 AND #3

#5 #1 AND #4

PubMed

#1 “Compound Danshen tablet*”[Title/Abstract] OR “Compound Danshen”[Title/Abstract]

#2 "myocardial infarction"[MeSH Terms] OR “Acute Coronary Syndrome”[MeSH Terms] OR "myocardial infarct*"[Title/Abstract] OR “Acute Coronary Syndrome”[Title/Abstract] OR “ACS”[Title/Abstract] OR “Acute Coronary Syndrome*”[Title/Abstract]OR “NSTE-ACS”[Title/Abstract] OR “Non–ST-Segment Elevation Acute Coronary Syndrome*”[Title/Abstract] OR "heart infarct*"[Title/Abstract] OR "cardiac infarct*"[Title/Abstract] OR "cardiovascular stroke*"[Title/Abstract] OR "cardiogenic shock"[Title/Abstract] OR “cardiovascular shock” [Title/Abstract] OR “cardiogenic stroke*” [Title/Abstract] OR "heart attack*"[Title/Abstract] OR "STEMI"[Title/Abstract] OR "NSTEMI"[Title/Abstract] OR “ST-segment elevation myocardial infarction”[Title/Abstract] OR “Non-ST-segment elevation myocardial infarction”[Title/Abstract] OR “MINOCA”[Title/Abstract] OR “Myocardial infarction with no obstructive coronary atherosclerosis” [Title/Abstract] OR “xiongbi” [Title/Abstract] OR “xiongbixintong” [Title/Abstract] OR “xiongbi xintong” [Title/Abstract] OR “zhenxintong” [Title/Abstract] OR “cuxintong” [Title/Abstract] OR “Xintong” [Title/Abstract] OR “juexintong” [Title/Abstract]

#3 “percutaneous coronary intervention” [MeSH Terms] OR “percutaneous coronary intervention*”[Title/Abstract] OR “percutaneous coronary revascularization*” [Title/Abstract] OR “coronary atherectom*”[Title/Abstract] OR “transluminal coronary angioplast*”[Title/Abstract] OR “transluminal coronary balloon dilation”[Title/Abstract] OR “coronary balloon angioplast*”[Title/Abstract] OR “PCI”[Title/Abstract] OR “Percutaneous Transluminal Coronary Angioplast*”[Title/Abstract] OR “PTCA”[Title/Abstract] OR “Rotational Atherectom*”[Title/Abstract] OR

“Directional Coronary Atherectom*”[Title/Abstract] OR “Balloon dilation”[Title/Abstract] OR “Artery Stenting”[Title/Abstract]

#4 #2 AND #3

#1 AND #4

Embase

#1 'Compound Danshen tablet*':ab,ti OR ‘Compound Danshen’:ab,ti

#2 'heart infarction'/exp

#3 'Acute Coronary Syndrome'/exp

#4 'cardiac infarct':ab,ti OR 'cardiac infarction':ab,ti OR 'cardial infarct':ab,ti OR 'heart attack':ab,ti OR 'heart infarct':ab,ti OR 'heart micro infarction':ab,ti OR 'heart muscle infarction':ab,ti OR 'infarction, heart':ab,ti OR 'myocardial infarct':ab,ti OR 'myocardial infarction':ab,ti OR 'myocardium infarct':ab,ti OR 'myocardium infarction':ab,ti OR 'premonitory infarction sign':ab,ti OR 'second heart attack':ab,ti OR 'subendocardial infarction':ab,ti OR 'transmural cardiac infarction':ab,ti OR 'transmural heart infarction':ab,ti OR 'transmural infarction, heart':ab,ti OR 'heart infarction':ab,ti OR ‘Acute Coronary Syndrome’:ab,ti OR ‘ACS’:ab,ti OR ‘Acute Coronary Syndrome*’:ab,ti OR ‘NSTE-ACS’:ab,ti OR ‘Non–ST-Segment Elevation Acute Coronary Syndrome*’:ab,ti OR 'STEMI':ab,ti OR 'NSTEMI':ab,ti OR ‘ST-segment elevation myocardial infarction’:ab,ti OR ‘Non-ST-segment elevation myocardial infarction’:ab,ti OR ‘MINOCA’:ab,ti OR ‘Myocardial infarction with no obstructive coronary atherosclerosis’:ab,ti OR ‘xiongbi’:ab,ti OR ‘xiongbixintong’:ab,ti OR ‘xiongbi Xintong’:ab,ti OR ‘zhenxintong’:ab,ti OR ‘cuxintong’:ab,ti OR ‘Xintong’:ab,ti OR ‘juexintong’:ab,ti OR ‘cardiovascular shock’:ab,ti OR ‘cardiogenic stroke*’:ab,ti

#5 ‘percutaneous coronary intervention'/exp

#6 ‘percutaneous coronary intervention*':ab,ti OR 'percutaneous coronary revascularization*':ab,ti OR 'coronary atherectom*':ab,ti OR 'transluminal coronary angioplast*':ab,ti OR 'transluminal coronary balloon dilation':ab,ti OR 'coronary balloon angioplast*':ab,ti OR 'PCI':ab,ti OR 'Percutaneous Transluminal Coronary Angioplast*':ab,ti OR 'PTCA':ab,ti OR 'Rotational Atherectom*':ab,ti OR 'Directional Coronary Atherectom*':ab,ti OR 'Balloon dilation':ab,ti OR 'Artery Stenting’:ab,ti

#7 #2 OR #3

#8 #4 OR #7

#9 #5 OR #6

#10 #8 AND #9

#11 #1 AND #10

Cochrane

#1 (Compound Danshen tablet*):ti,ab,kw OR (Compound Danshen):ti,ab,kw

#2 MeSH descriptor: [Myocardial Infarction] explode all trees

#3 MeSH descriptor: [Acute Coronary Syndrome] explode all trees

#4 (myocardial infarct*):ti,ab,kw OR (heart infarct*):ti,ab,kw OR (cardiac infarct*):ti,ab,kw OR (cardiovascular stroke*):ti,ab,kw OR (cardiogenic shock):ti,ab,kw OR (heart attack*):ti,ab,kw OR (Acute Coronary Syndrome):ti,ab,kw OR (ACS):ti,ab,kw OR (Acute Coronary Syndrome*):ti,ab,kw OR (NSTE-ACS):ti,ab,kw OR (Non–ST-Segment Elevation Acute Coronary Syndrome*):ti,ab,kw OR (STEMI):ti,ab,kw OR (NSTEMI):ti,ab,kw OR (ST-segment elevation myocardial infarction):ti,ab,kw OR (Non-ST-segment elevation myocardial infarction):ti,ab,kw OR (MINOCA):ti,ab,kw OR (Myocardial infarction with no obstructive coronary atherosclerosis):ti,ab,kw OR (xiongbi):ti,ab,kw OR (xiongbixintong):ti,ab,kw OR (xiongbi Xintong):ti,ab,kw OR (zhenxintong):ti,ab,kw OR (cuxintong):ti,ab,kw OR (Xintong):ti,ab,kw OR (juexintong):ti,ab,kw OR (cardiovascular shock):ti,ab,kw OR (cardiogenic stroke*):ti,ab,kw

#5 MeSH descriptor: [Percutaneous Coronary Intervention] explode all trees

#6 (percutaneous coronary intervention):ti,ab,kw OR (percutaneous coronary intervention*):ti,ab,kw OR (percutaneous coronary revascularization*):ti,ab,kw OR (coronary atherectom*):ti,ab,kw OR (transluminal coronary angioplast*):ti,ab,kw OR (transluminal coronary balloon dilation):ti,ab,kw OR (coronary balloon angioplast*):ti,ab,kw OR (PCI):ti,ab,kw OR (Percutaneous Transluminal Coronary Angioplast*):ti,ab,kw OR (PTCA):ti,ab,kw OR (Rotational Atherectom*):ti,ab,kw OR (Directional Coronary Atherectom*):ti,ab,kw OR (Balloon dilation):ti,ab,kw OR (Artery Stenting):ti,ab,kw

#7 #2 OR #3

#8 #4 OR #7

#9 #5 OR #6

#10 #8 AND #9

#11 #1 AND #10

29.Cmpound Danshen dripping pills

CNKI

(TKA=‘复方丹参滴丸’ + ‘复方丹参’) and ((TKA=‘心肌梗死’ + ‘心梗’ + ‘心肌梗塞’ + ‘急性冠脉综合征’+ ‘ACS’ + ‘STEMI’ + ‘NSTEMI’ + ‘胸痹’ + ‘胸痹心痛’ + ‘真心痛’ + ‘卒心痛’ + ‘心痛’ + ‘猝心痛’ + ‘厥心痛’ + ) and (TKA=‘PCI’ + ‘PTCA’ + ‘经皮冠状动脉介入治疗术’ + ‘经皮冠状动脉介入’ + ‘经皮冠状动脉’ + ‘冠脉介入’ + ‘经皮冠脉’ + ‘血管成形术’ + ‘支架置入术’ + ‘冠状动脉成形术’ + ‘球囊’ + ‘支架’))

WangFang Data

主题:(“复方丹参滴丸” OR “复方丹参”) and ((主题:心肌梗死 OR 心梗 OR 心肌梗塞 OR 急性冠脉综合征 OR ACS OR STEMI OR NSTEMI OR 胸痹 OR 胸痹心痛 OR 真心痛 OR 卒心痛 OR 心痛 OR 猝心痛 OR 厥心痛) and 主题:(PCI OR PTCA OR 经皮冠状动脉介入治疗术 OR 经皮冠状动脉介入 OR 经皮冠状动脉 OR 冠脉介入 OR 经皮冠脉 OR 血管成形术 OR 支架置入术 OR 冠状动脉成形术 OR 球囊 OR 支架))

VIP

(M=(“复方丹参滴丸” OR “复方丹参”) OR R=(“复方丹参滴丸” OR “复方丹参”)) AND ((M=(心肌梗死 OR 心梗 OR 心肌梗塞 OR 急性冠脉综合征 OR STEMI OR NSTEMI OR 胸痹 OR 胸痹心痛 OR 真心痛 OR 卒心痛 OR 心痛 OR 猝心痛 OR 厥心痛) OR R=(心肌梗死 OR 心梗 OR 心肌梗塞 OR ACS OR STEMI OR NSTEMI OR 胸痹 OR 胸痹心痛 OR 真心痛 OR 卒心痛 OR 心痛 OR 猝心痛 OR 厥心痛)) AND (M=(PCI OR PTCA OR 经皮冠状动脉介入治疗术 OR 经皮冠状动脉介入 OR 经皮冠状动脉 OR 冠脉介入 OR 经皮冠脉 OR 血管成形术 OR 支架置入术 OR 冠状动脉成形术 OR 球囊 OR 支架) OR R=(PCI OR PTCA OR 经皮冠状动脉介入治疗术 OR 经皮冠状动脉介入 OR 经皮冠状动脉 OR 冠脉介入 OR 经皮冠脉 OR 血管成形术 OR 支架置入术 OR 冠状动脉成形术 OR 球囊 OR 支架)))

Web of Science

#1 TS=(compound Danshen dripping pill* OR Compound Danshen)

#2 TS=(myocardial infarct* OR heart infarct* OR cardiac infarct* OR cardiovascular stroke* OR cardiogenic shock OR heart attack* OR ACS OR STEMI OR NSTEMI OR ST-segment elevation myocardial infarction OR Non-ST-segment elevation myocardial infarction OR MINOCA OR Myocardial infarction with no obstructive coronary atherosclerosis OR xiongbi OR xiongbixintong OR xiongbi Xintong OR zhenxintong OR cuxintong OR Xintong OR juexintong OR cardiovascular shock OR cardiogenic stroke*)

#3 TS=(percutaneous coronary intervention OR percutaneous coronary intervention* OR percutaneous coronary revascularization* OR coronary atherectom* OR transluminal coronary angioplast* OR transluminal coronary balloon dilation OR coronary balloon angioplast* OR PCI OR Percutaneous Transluminal Coronary Angioplast* OR PTCA OR Rotational Atherectom* OR Directional Coronary Atherectom* OR Balloon dilation OR Artery Stenting)

#4 #2 AND #3

#5 #1 AND #4

PubMed

#1 “Compound Danshen dripping pill*”[Title/Abstract] OR “Compound Danshen”[Title/Abstract]

#2 "myocardial infarction"[MeSH Terms] OR “Acute Coronary Syndrome”[MeSH Terms] OR "myocardial infarct*"[Title/Abstract] OR “Acute Coronary Syndrome”[Title/Abstract] OR “ACS”[Title/Abstract] OR “Acute Coronary Syndrome*”[Title/Abstract]OR “NSTE-ACS”[Title/Abstract] OR “Non–ST-Segment Elevation Acute Coronary Syndrome*”[Title/Abstract] OR "heart infarct*"[Title/Abstract] OR "cardiac infarct*"[Title/Abstract] OR "cardiovascular stroke*"[Title/Abstract] OR "cardiogenic shock"[Title/Abstract] OR “cardiovascular shock” [Title/Abstract] OR “cardiogenic stroke*” [Title/Abstract] OR "heart attack*"[Title/Abstract] OR "STEMI"[Title/Abstract] OR "NSTEMI"[Title/Abstract] OR “ST-segment elevation myocardial infarction”[Title/Abstract] OR “Non-ST-segment elevation myocardial infarction”[Title/Abstract] OR “MINOCA”[Title/Abstract] OR “Myocardial infarction with no obstructive coronary atherosclerosis” [Title/Abstract] OR “xiongbi” [Title/Abstract] OR “xiongbixintong” [Title/Abstract] OR “xiongbi xintong” [Title/Abstract] OR “zhenxintong” [Title/Abstract] OR “cuxintong” [Title/Abstract] OR “Xintong” [Title/Abstract] OR “juexintong” [Title/Abstract]

#3 “percutaneous coronary intervention” [MeSH Terms] OR “percutaneous coronary intervention*”[Title/Abstract] OR “percutaneous coronary revascularization*” [Title/Abstract] OR “coronary atherectom*”[Title/Abstract] OR “transluminal coronary angioplast*”[Title/Abstract] OR “transluminal coronary balloon dilation”[Title/Abstract] OR “coronary balloon angioplast*”[Title/Abstract] OR “PCI”[Title/Abstract] OR “Percutaneous Transluminal Coronary Angioplast*”[Title/Abstract] OR “PTCA”[Title/Abstract] OR “Rotational Atherectom*”[Title/Abstract] OR

“Directional Coronary Atherectom*”[Title/Abstract] OR “Balloon dilation”[Title/Abstract] OR “Artery Stenting”[Title/Abstract]

#4 #2 AND #3

#1 AND #4

Embase

#1 'Compound Danshen dripping pill*':ab,ti OR ‘Compound Danshen’:ab,ti

#2 'heart infarction'/exp

#3 'Acute Coronary Syndrome'/exp

#4 'cardiac infarct':ab,ti OR 'cardiac infarction':ab,ti OR 'cardial infarct':ab,ti OR 'heart attack':ab,ti OR 'heart infarct':ab,ti OR 'heart micro infarction':ab,ti OR 'heart muscle infarction':ab,ti OR 'infarction, heart':ab,ti OR 'myocardial infarct':ab,ti OR 'myocardial infarction':ab,ti OR 'myocardium infarct':ab,ti OR 'myocardium infarction':ab,ti OR 'premonitory infarction sign':ab,ti OR 'second heart attack':ab,ti OR 'subendocardial infarction':ab,ti OR 'transmural cardiac infarction':ab,ti OR 'transmural heart infarction':ab,ti OR 'transmural infarction, heart':ab,ti OR 'heart infarction':ab,ti OR ‘Acute Coronary Syndrome’:ab,ti OR ‘ACS’:ab,ti OR ‘Acute Coronary Syndrome*’:ab,ti OR ‘NSTE-ACS’:ab,ti OR ‘Non–ST-Segment Elevation Acute Coronary Syndrome*’:ab,ti OR 'STEMI':ab,ti OR 'NSTEMI':ab,ti OR ‘ST-segment elevation myocardial infarction’:ab,ti OR ‘Non-ST-segment elevation myocardial infarction’:ab,ti OR ‘MINOCA’:ab,ti OR ‘Myocardial infarction with no obstructive coronary atherosclerosis’:ab,ti OR ‘xiongbi’:ab,ti OR ‘xiongbixintong’:ab,ti OR ‘xiongbi Xintong’:ab,ti OR ‘zhenxintong’:ab,ti OR ‘cuxintong’:ab,ti OR ‘Xintong’:ab,ti OR ‘juexintong’:ab,ti OR ‘cardiovascular shock’:ab,ti OR ‘cardiogenic stroke*’:ab,ti

#5 ‘percutaneous coronary intervention'/exp

#6 ‘percutaneous coronary intervention*':ab,ti OR 'percutaneous coronary revascularization*':ab,ti OR 'coronary atherectom*':ab,ti OR 'transluminal coronary angioplast*':ab,ti OR 'transluminal coronary balloon dilation':ab,ti OR 'coronary balloon angioplast*':ab,ti OR 'PCI':ab,ti OR 'Percutaneous Transluminal Coronary Angioplast*':ab,ti OR 'PTCA':ab,ti OR 'Rotational Atherectom*':ab,ti OR 'Directional Coronary Atherectom*':ab,ti OR 'Balloon dilation':ab,ti OR 'Artery Stenting’:ab,ti

#7 #2 OR #3

#8 #4 OR #7

#9 #5 OR #6

#10 #8 AND #9

#11 #1 AND #10

Cochrane

#1 (Compound Danshen dripping pill*):ti,ab,kw OR (Compound Danshen):ti,ab,kw

#2 MeSH descriptor: [Myocardial Infarction] explode all trees

#3 MeSH descriptor: [Acute Coronary Syndrome] explode all trees

#4 (myocardial infarct*):ti,ab,kw OR (heart infarct*):ti,ab,kw OR (cardiac infarct*):ti,ab,kw OR (cardiovascular stroke*):ti,ab,kw OR (cardiogenic shock):ti,ab,kw OR (heart attack*):ti,ab,kw OR (Acute Coronary Syndrome):ti,ab,kw OR (ACS):ti,ab,kw OR (Acute Coronary Syndrome*):ti,ab,kw OR (NSTE-ACS):ti,ab,kw OR (Non–ST-Segment Elevation Acute Coronary Syndrome*):ti,ab,kw OR (STEMI):ti,ab,kw OR (NSTEMI):ti,ab,kw OR (ST-segment elevation myocardial infarction):ti,ab,kw OR (Non-ST-segment elevation myocardial infarction):ti,ab,kw OR (MINOCA):ti,ab,kw OR (Myocardial infarction with no obstructive coronary atherosclerosis):ti,ab,kw OR (xiongbi):ti,ab,kw OR (xiongbixintong):ti,ab,kw OR (xiongbi Xintong):ti,ab,kw OR (zhenxintong):ti,ab,kw OR (cuxintong):ti,ab,kw OR (Xintong):ti,ab,kw OR (juexintong):ti,ab,kw OR (cardiovascular shock):ti,ab,kw OR (cardiogenic stroke*):ti,ab,kw

#5 MeSH descriptor: [Percutaneous Coronary Intervention] explode all trees

#6 (percutaneous coronary intervention):ti,ab,kw OR (percutaneous coronary intervention*):ti,ab,kw OR (percutaneous coronary revascularization*):ti,ab,kw OR (coronary atherectom*):ti,ab,kw OR (transluminal coronary angioplast*):ti,ab,kw OR (transluminal coronary balloon dilation):ti,ab,kw OR (coronary balloon angioplast*):ti,ab,kw OR (PCI):ti,ab,kw OR (Percutaneous Transluminal Coronary Angioplast*):ti,ab,kw OR (PTCA):ti,ab,kw OR (Rotational Atherectom*):ti,ab,kw OR (Directional Coronary Atherectom*):ti,ab,kw OR (Balloon dilation):ti,ab,kw OR (Artery Stenting):ti,ab,kw

#7 #2 OR #3

#8 #4 OR #7

#9 #5 OR #6

#10 #8 AND #9

#11 #1 AND #10

30.Compound Xueshuantong capsules

CNKI

(TKA=‘复方血栓通胶囊’ + ‘复方血栓通’) and ((TKA=‘心肌梗死’ + ‘心梗’ + ‘心肌梗塞’ + ‘急性冠脉综合征’+ ‘ACS’ + ‘STEMI’ + ‘NSTEMI’ + ‘胸痹’ + ‘胸痹心痛’ + ‘真心痛’ + ‘卒心痛’ + ‘心痛’ + ‘猝心痛’ + ‘厥心痛’ + ) and (TKA=‘PCI’ + ‘PTCA’ + ‘经皮冠状动脉介入治疗术’ + ‘经皮冠状动脉介入’ + ‘经皮冠状动脉’ + ‘冠脉介入’ + ‘经皮冠脉’ + ‘血管成形术’ + ‘支架置入术’ + ‘冠状动脉成形术’ + ‘球囊’ + ‘支架’))

WangFang Data

主题:(“复方血栓通胶囊” OR “复方血栓通”) and ((主题:心肌梗死 OR 心梗 OR 心肌梗塞 OR 急性冠脉综合征 OR ACS OR STEMI OR NSTEMI OR 胸痹 OR 胸痹心痛 OR 真心痛 OR 卒心痛 OR 心痛 OR 猝心痛 OR 厥心痛) and 主题:(PCI OR PTCA OR 经皮冠状动脉介入治疗术 OR 经皮冠状动脉介入 OR 经皮冠状动脉 OR 冠脉介入 OR 经皮冠脉 OR 血管成形术 OR 支架置入术 OR 冠状动脉成形术 OR 球囊 OR 支架))

VIP

(M=(“复方血栓通胶囊” OR “复方血栓通”) OR R=(“复方血栓通胶囊” OR “复方血栓通”)) AND ((M=(心肌梗死 OR 心梗 OR 心肌梗塞 OR 急性冠脉综合征 OR STEMI OR NSTEMI OR 胸痹 OR 胸痹心痛 OR 真心痛 OR 卒心痛 OR 心痛 OR 猝心痛 OR 厥心痛) OR R=(心肌梗死 OR 心梗 OR 心肌梗塞 OR ACS OR STEMI OR NSTEMI OR 胸痹 OR 胸痹心痛 OR 真心痛 OR 卒心痛 OR 心痛 OR 猝心痛 OR 厥心痛)) AND (M=(PCI OR PTCA OR 经皮冠状动脉介入治疗术 OR 经皮冠状动脉介入 OR 经皮冠状动脉 OR 冠脉介入 OR 经皮冠脉 OR 血管成形术 OR 支架置入术 OR 冠状动脉成形术 OR 球囊 OR 支架) OR R=(PCI OR PTCA OR 经皮冠状动脉介入治疗术 OR 经皮冠状动脉介入 OR 经皮冠状动脉 OR 冠脉介入 OR 经皮冠脉 OR 血管成形术 OR 支架置入术 OR 冠状动脉成形术 OR 球囊 OR 支架)))

Web of Science

#1 TS=(Compound Xueshuantong Capsule* OR Compound Xueshuantong)

#2 TS=(myocardial infarct* OR heart infarct* OR cardiac infarct* OR cardiovascular stroke* OR cardiogenic shock OR heart attack* OR ACS OR STEMI OR NSTEMI OR ST-segment elevation myocardial infarction OR Non-ST-segment elevation myocardial infarction OR MINOCA OR Myocardial infarction with no obstructive coronary atherosclerosis OR xiongbi OR xiongbixintong OR xiongbi Xintong OR zhenxintong OR cuxintong OR Xintong OR juexintong OR cardiovascular shock OR cardiogenic stroke*)

#3 TS=(percutaneous coronary intervention OR percutaneous coronary intervention* OR percutaneous coronary revascularization* OR coronary atherectom* OR transluminal coronary angioplast* OR transluminal coronary balloon dilation OR coronary balloon angioplast* OR PCI OR Percutaneous Transluminal Coronary Angioplast* OR PTCA OR Rotational Atherectom* OR Directional Coronary Atherectom* OR Balloon dilation OR Artery Stenting)

#4 #2 AND #3

#5 #1 AND #4

PubMed

#1 “Compound Xueshuantong Capsule*”[Title/Abstract] OR “Compound Xueshuantong”[Title/Abstract]

#2 "myocardial infarction"[MeSH Terms] OR “Acute Coronary Syndrome”[MeSH Terms] OR "myocardial infarct*"[Title/Abstract] OR “Acute Coronary Syndrome”[Title/Abstract] OR “ACS”[Title/Abstract] OR “Acute Coronary Syndrome*”[Title/Abstract]OR “NSTE-ACS”[Title/Abstract] OR “Non–ST-Segment Elevation Acute Coronary Syndrome*”[Title/Abstract] OR "heart infarct*"[Title/Abstract] OR "cardiac infarct*"[Title/Abstract] OR "cardiovascular stroke*"[Title/Abstract] OR "cardiogenic shock"[Title/Abstract] OR “cardiovascular shock” [Title/Abstract] OR “cardiogenic stroke*” [Title/Abstract] OR "heart attack*"[Title/Abstract] OR "STEMI"[Title/Abstract] OR "NSTEMI"[Title/Abstract] OR “ST-segment elevation myocardial infarction”[Title/Abstract] OR “Non-ST-segment elevation myocardial infarction”[Title/Abstract] OR “MINOCA”[Title/Abstract] OR “Myocardial infarction with no obstructive coronary atherosclerosis” [Title/Abstract] OR “xiongbi” [Title/Abstract] OR “xiongbixintong” [Title/Abstract] OR “xiongbi xintong” [Title/Abstract] OR “zhenxintong” [Title/Abstract] OR “cuxintong” [Title/Abstract] OR “Xintong” [Title/Abstract] OR “juexintong” [Title/Abstract]

#3 “percutaneous coronary intervention” [MeSH Terms] OR “percutaneous coronary intervention*”[Title/Abstract] OR “percutaneous coronary revascularization*” [Title/Abstract] OR “coronary atherectom*”[Title/Abstract] OR “transluminal coronary angioplast*”[Title/Abstract] OR “transluminal coronary balloon dilation”[Title/Abstract] OR “coronary balloon angioplast*”[Title/Abstract] OR “PCI”[Title/Abstract] OR “Percutaneous Transluminal Coronary Angioplast*”[Title/Abstract] OR “PTCA”[Title/Abstract] OR “Rotational Atherectom*”[Title/Abstract] OR

“Directional Coronary Atherectom*”[Title/Abstract] OR “Balloon dilation”[Title/Abstract] OR “Artery Stenting”[Title/Abstract]

#4 #2 AND #3

#1 AND #4

Embase

#1 'Compound Xueshuantong Capsule*':ab,ti OR ‘Compound Xueshuantong’:ab,ti

#2 'heart infarction'/exp

#3 'Acute Coronary Syndrome'/exp

#4 'cardiac infarct':ab,ti OR 'cardiac infarction':ab,ti OR 'cardial infarct':ab,ti OR 'heart attack':ab,ti OR 'heart infarct':ab,ti OR 'heart micro infarction':ab,ti OR 'heart muscle infarction':ab,ti OR 'infarction, heart':ab,ti OR 'myocardial infarct':ab,ti OR 'myocardial infarction':ab,ti OR 'myocardium infarct':ab,ti OR 'myocardium infarction':ab,ti OR 'premonitory infarction sign':ab,ti OR 'second heart attack':ab,ti OR 'subendocardial infarction':ab,ti OR 'transmural cardiac infarction':ab,ti OR 'transmural heart infarction':ab,ti OR 'transmural infarction, heart':ab,ti OR 'heart infarction':ab,ti OR ‘Acute Coronary Syndrome’:ab,ti OR ‘ACS’:ab,ti OR ‘Acute Coronary Syndrome*’:ab,ti OR ‘NSTE-ACS’:ab,ti OR ‘Non–ST-Segment Elevation Acute Coronary Syndrome*’:ab,ti OR 'STEMI':ab,ti OR 'NSTEMI':ab,ti OR ‘ST-segment elevation myocardial infarction’:ab,ti OR ‘Non-ST-segment elevation myocardial infarction’:ab,ti OR ‘MINOCA’:ab,ti OR ‘Myocardial infarction with no obstructive coronary atherosclerosis’:ab,ti OR ‘xiongbi’:ab,ti OR ‘xiongbixintong’:ab,ti OR ‘xiongbi Xintong’:ab,ti OR ‘zhenxintong’:ab,ti OR ‘cuxintong’:ab,ti OR ‘Xintong’:ab,ti OR ‘juexintong’:ab,ti OR ‘cardiovascular shock’:ab,ti OR ‘cardiogenic stroke*’:ab,ti

#5 ‘percutaneous coronary intervention'/exp

#6 ‘percutaneous coronary intervention*':ab,ti OR 'percutaneous coronary revascularization*':ab,ti OR 'coronary atherectom*':ab,ti OR 'transluminal coronary angioplast*':ab,ti OR 'transluminal coronary balloon dilation':ab,ti OR 'coronary balloon angioplast*':ab,ti OR 'PCI':ab,ti OR 'Percutaneous Transluminal Coronary Angioplast*':ab,ti OR 'PTCA':ab,ti OR 'Rotational Atherectom*':ab,ti OR 'Directional Coronary Atherectom*':ab,ti OR 'Balloon dilation':ab,ti OR 'Artery Stenting’:ab,ti

#7 #2 OR #3

#8 #4 OR #7

#9 #5 OR #6

#10 #8 AND #9

#11 #1 AND #10

Cochrane

#1 (Compound Xueshuantong Capsule*):ti,ab,kw OR (Compound Xueshuantong):ti,ab,kw

#2 MeSH descriptor: [Myocardial Infarction] explode all trees

#3 MeSH descriptor: [Acute Coronary Syndrome] explode all trees

#4 (myocardial infarct*):ti,ab,kw OR (heart infarct*):ti,ab,kw OR (cardiac infarct*):ti,ab,kw OR (cardiovascular stroke*):ti,ab,kw OR (cardiogenic shock):ti,ab,kw OR (heart attack*):ti,ab,kw OR (Acute Coronary Syndrome):ti,ab,kw OR (ACS):ti,ab,kw OR (Acute Coronary Syndrome*):ti,ab,kw OR (NSTE-ACS):ti,ab,kw OR (Non–ST-Segment Elevation Acute Coronary Syndrome*):ti,ab,kw OR (STEMI):ti,ab,kw OR (NSTEMI):ti,ab,kw OR (ST-segment elevation myocardial infarction):ti,ab,kw OR (Non-ST-segment elevation myocardial infarction):ti,ab,kw OR (MINOCA):ti,ab,kw OR (Myocardial infarction with no obstructive coronary atherosclerosis):ti,ab,kw OR (xiongbi):ti,ab,kw OR (xiongbixintong):ti,ab,kw OR (xiongbi Xintong):ti,ab,kw OR (zhenxintong):ti,ab,kw OR (cuxintong):ti,ab,kw OR (Xintong):ti,ab,kw OR (juexintong):ti,ab,kw OR (cardiovascular shock):ti,ab,kw OR (cardiogenic stroke*):ti,ab,kw

#5 MeSH descriptor: [Percutaneous Coronary Intervention] explode all trees

#6 (percutaneous coronary intervention):ti,ab,kw OR (percutaneous coronary intervention*):ti,ab,kw OR (percutaneous coronary revascularization*):ti,ab,kw OR (coronary atherectom*):ti,ab,kw OR (transluminal coronary angioplast*):ti,ab,kw OR (transluminal coronary balloon dilation):ti,ab,kw OR (coronary balloon angioplast*):ti,ab,kw OR (PCI):ti,ab,kw OR (Percutaneous Transluminal Coronary Angioplast*):ti,ab,kw OR (PTCA):ti,ab,kw OR (Rotational Atherectom*):ti,ab,kw OR (Directional Coronary Atherectom*):ti,ab,kw OR (Balloon dilation):ti,ab,kw OR (Artery Stenting):ti,ab,kw

#7 #2 OR #3

#8 #4 OR #7

#9 #5 OR #6

#10 #8 AND #9

#11 #1 AND #10

31.Yangxinshi tablets

CNKI

(TKA=‘养心氏片’ + ‘养心氏’) and ((TKA=‘心肌梗死’ + ‘心梗’ + ‘心肌梗塞’ + ‘急性冠脉综合征’+ ‘ACS’ + ‘STEMI’ + ‘NSTEMI’ + ‘胸痹’ + ‘胸痹心痛’ + ‘真心痛’ + ‘卒心痛’ + ‘心痛’ + ‘猝心痛’ + ‘厥心痛’ + ) and (TKA=‘PCI’ + ‘PTCA’ + ‘经皮冠状动脉介入治疗术’ + ‘经皮冠状动脉介入’ + ‘经皮冠状动脉’ + ‘冠脉介入’ + ‘经皮冠脉’ + ‘血管成形术’ + ‘支架置入术’ + ‘冠状动脉成形术’ + ‘球囊’ + ‘支架’))

WangFang Data

主题:(“养心氏片” OR “养心氏”) and ((主题:心肌梗死 OR 心梗 OR 心肌梗塞 OR 急性冠脉综合征 OR ACS OR STEMI OR NSTEMI OR 胸痹 OR 胸痹心痛 OR 真心痛 OR 卒心痛 OR 心痛 OR 猝心痛 OR 厥心痛) and 主题:(PCI OR PTCA OR 经皮冠状动脉介入治疗术 OR 经皮冠状动脉介入 OR 经皮冠状动脉 OR 冠脉介入 OR 经皮冠脉 OR 血管成形术 OR 支架置入术 OR 冠状动脉成形术 OR 球囊 OR 支架))

VIP

(M=(“养心氏片” OR “养心氏”) OR R=(“养心氏片” OR “养心氏”)) AND ((M=(心肌梗死 OR 心梗 OR 心肌梗塞 OR 急性冠脉综合征 OR STEMI OR NSTEMI OR 胸痹 OR 胸痹心痛 OR 真心痛 OR 卒心痛 OR 心痛 OR 猝心痛 OR 厥心痛) OR R=(心肌梗死 OR 心梗 OR 心肌梗塞 OR ACS OR STEMI OR NSTEMI OR 胸痹 OR 胸痹心痛 OR 真心痛 OR 卒心痛 OR 心痛 OR 猝心痛 OR 厥心痛)) AND (M=(PCI OR PTCA OR 经皮冠状动脉介入治疗术 OR 经皮冠状动脉介入 OR 经皮冠状动脉 OR 冠脉介入 OR 经皮冠脉 OR 血管成形术 OR 支架置入术 OR 冠状动脉成形术 OR 球囊 OR 支架) OR R=(PCI OR PTCA OR 经皮冠状动脉介入治疗术 OR 经皮冠状动脉介入 OR 经皮冠状动脉 OR 冠脉介入 OR 经皮冠脉 OR 血管成形术 OR 支架置入术 OR 冠状动脉成形术 OR 球囊 OR 支架)))

Web of Science

#1 TS=(yangxinshi tablet* OR yangxinshi)

#2 TS=(myocardial infarct* OR heart infarct* OR cardiac infarct* OR cardiovascular stroke* OR cardiogenic shock OR heart attack* OR ACS OR STEMI OR NSTEMI OR ST-segment elevation myocardial infarction OR Non-ST-segment elevation myocardial infarction OR MINOCA OR Myocardial infarction with no obstructive coronary atherosclerosis OR xiongbi OR xiongbixintong OR xiongbi Xintong OR zhenxintong OR cuxintong OR Xintong OR juexintong OR cardiovascular shock OR cardiogenic stroke*)

#3 TS=(percutaneous coronary intervention OR percutaneous coronary intervention* OR percutaneous coronary revascularization* OR coronary atherectom* OR transluminal coronary angioplast* OR transluminal coronary balloon dilation OR coronary balloon angioplast* OR PCI OR Percutaneous Transluminal Coronary Angioplast* OR PTCA OR Rotational Atherectom* OR Directional Coronary Atherectom* OR Balloon dilation OR Artery Stenting)

#4 #2 AND #3

#5 #1 AND #4

PubMed

#1 “yangxinshi tablet*”[Title/Abstract] OR “yangxinshi”[Title/Abstract]

#2 "myocardial infarction"[MeSH Terms] OR “Acute Coronary Syndrome”[MeSH Terms] OR "myocardial infarct*"[Title/Abstract] OR “Acute Coronary Syndrome”[Title/Abstract] OR “ACS”[Title/Abstract] OR “Acute Coronary Syndrome*”[Title/Abstract]OR “NSTE-ACS”[Title/Abstract] OR “Non–ST-Segment Elevation Acute Coronary Syndrome*”[Title/Abstract] OR "heart infarct*"[Title/Abstract] OR "cardiac infarct*"[Title/Abstract] OR "cardiovascular stroke*"[Title/Abstract] OR "cardiogenic shock"[Title/Abstract] OR “cardiovascular shock” [Title/Abstract] OR “cardiogenic stroke*” [Title/Abstract] OR "heart attack*"[Title/Abstract] OR "STEMI"[Title/Abstract] OR "NSTEMI"[Title/Abstract] OR “ST-segment elevation myocardial infarction”[Title/Abstract] OR “Non-ST-segment elevation myocardial infarction”[Title/Abstract] OR “MINOCA”[Title/Abstract] OR “Myocardial infarction with no obstructive coronary atherosclerosis” [Title/Abstract] OR “xiongbi” [Title/Abstract] OR “xiongbixintong” [Title/Abstract] OR “xiongbi xintong” [Title/Abstract] OR “zhenxintong” [Title/Abstract] OR “cuxintong” [Title/Abstract] OR “Xintong” [Title/Abstract] OR “juexintong” [Title/Abstract]

#3 “percutaneous coronary intervention” [MeSH Terms] OR “percutaneous coronary intervention*”[Title/Abstract] OR “percutaneous coronary revascularization*” [Title/Abstract] OR “coronary atherectom*”[Title/Abstract] OR “transluminal coronary angioplast*”[Title/Abstract] OR “transluminal coronary balloon dilation”[Title/Abstract] OR “coronary balloon angioplast*”[Title/Abstract] OR “PCI”[Title/Abstract] OR “Percutaneous Transluminal Coronary Angioplast*”[Title/Abstract] OR “PTCA”[Title/Abstract] OR “Rotational Atherectom*”[Title/Abstract] OR

“Directional Coronary Atherectom*”[Title/Abstract] OR “Balloon dilation”[Title/Abstract] OR “Artery Stenting”[Title/Abstract]

#4 #2 AND #3

#1 AND #4

Embase

#1 'yangxinshi tablet*':ab,ti OR ‘yangxinshi’:ab,ti

#2 'heart infarction'/exp

#3 'Acute Coronary Syndrome'/exp

#4 'cardiac infarct':ab,ti OR 'cardiac infarction':ab,ti OR 'cardial infarct':ab,ti OR 'heart attack':ab,ti OR 'heart infarct':ab,ti OR 'heart micro infarction':ab,ti OR 'heart muscle infarction':ab,ti OR 'infarction, heart':ab,ti OR 'myocardial infarct':ab,ti OR 'myocardial infarction':ab,ti OR 'myocardium infarct':ab,ti OR 'myocardium infarction':ab,ti OR 'premonitory infarction sign':ab,ti OR 'second heart attack':ab,ti OR 'subendocardial infarction':ab,ti OR 'transmural cardiac infarction':ab,ti OR 'transmural heart infarction':ab,ti OR 'transmural infarction, heart':ab,ti OR 'heart infarction':ab,ti OR ‘Acute Coronary Syndrome’:ab,ti OR ‘ACS’:ab,ti OR ‘Acute Coronary Syndrome*’:ab,ti OR ‘NSTE-ACS’:ab,ti OR ‘Non–ST-Segment Elevation Acute Coronary Syndrome*’:ab,ti OR 'STEMI':ab,ti OR 'NSTEMI':ab,ti OR ‘ST-segment elevation myocardial infarction’:ab,ti OR ‘Non-ST-segment elevation myocardial infarction’:ab,ti OR ‘MINOCA’:ab,ti OR ‘Myocardial infarction with no obstructive coronary atherosclerosis’:ab,ti OR ‘xiongbi’:ab,ti OR ‘xiongbixintong’:ab,ti OR ‘xiongbi Xintong’:ab,ti OR ‘zhenxintong’:ab,ti OR ‘cuxintong’:ab,ti OR ‘Xintong’:ab,ti OR ‘juexintong’:ab,ti OR ‘cardiovascular shock’:ab,ti OR ‘cardiogenic stroke*’:ab,ti

#5 ‘percutaneous coronary intervention'/exp

#6 ‘percutaneous coronary intervention*':ab,ti OR 'percutaneous coronary revascularization*':ab,ti OR 'coronary atherectom*':ab,ti OR 'transluminal coronary angioplast*':ab,ti OR 'transluminal coronary balloon dilation':ab,ti OR 'coronary balloon angioplast*':ab,ti OR 'PCI':ab,ti OR 'Percutaneous Transluminal Coronary Angioplast*':ab,ti OR 'PTCA':ab,ti OR 'Rotational Atherectom*':ab,ti OR 'Directional Coronary Atherectom*':ab,ti OR 'Balloon dilation':ab,ti OR 'Artery Stenting’:ab,ti

#7 #2 OR #3

#8 #4 OR #7

#9 #5 OR #6

#10 #8 AND #9

#11 #1 AND #10

Cochrane

#1 (yangxinshi tablet*):ti,ab,kw OR (yangxinshi):ti,ab,kw

#2 MeSH descriptor: [Myocardial Infarction] explode all trees

#3 MeSH descriptor: [Acute Coronary Syndrome] explode all trees

#4 (myocardial infarct*):ti,ab,kw OR (heart infarct*):ti,ab,kw OR (cardiac infarct*):ti,ab,kw OR (cardiovascular stroke*):ti,ab,kw OR (cardiogenic shock):ti,ab,kw OR (heart attack*):ti,ab,kw OR (Acute Coronary Syndrome):ti,ab,kw OR (ACS):ti,ab,kw OR (Acute Coronary Syndrome*):ti,ab,kw OR (NSTE-ACS):ti,ab,kw OR (Non–ST-Segment Elevation Acute Coronary Syndrome*):ti,ab,kw OR (STEMI):ti,ab,kw OR (NSTEMI):ti,ab,kw OR (ST-segment elevation myocardial infarction):ti,ab,kw OR (Non-ST-segment elevation myocardial infarction):ti,ab,kw OR (MINOCA):ti,ab,kw OR (Myocardial infarction with no obstructive coronary atherosclerosis):ti,ab,kw OR (xiongbi):ti,ab,kw OR (xiongbixintong):ti,ab,kw OR (xiongbi Xintong):ti,ab,kw OR (zhenxintong):ti,ab,kw OR (cuxintong):ti,ab,kw OR (Xintong):ti,ab,kw OR (juexintong):ti,ab,kw OR (cardiovascular shock):ti,ab,kw OR (cardiogenic stroke*):ti,ab,kw

#5 MeSH descriptor: [Percutaneous Coronary Intervention] explode all trees

#6 (percutaneous coronary intervention):ti,ab,kw OR (percutaneous coronary intervention*):ti,ab,kw OR (percutaneous coronary revascularization*):ti,ab,kw OR (coronary atherectom*):ti,ab,kw OR (transluminal coronary angioplast*):ti,ab,kw OR (transluminal coronary balloon dilation):ti,ab,kw OR (coronary balloon angioplast*):ti,ab,kw OR (PCI):ti,ab,kw OR (Percutaneous Transluminal Coronary Angioplast*):ti,ab,kw OR (PTCA):ti,ab,kw OR (Rotational Atherectom*):ti,ab,kw OR (Directional Coronary Atherectom*):ti,ab,kw OR (Balloon dilation):ti,ab,kw OR (Artery Stenting):ti,ab,kw

#7 #2 OR #3

#8 #4 OR #7

#9 #5 OR #6

#10 #8 AND #9

#11 #1 AND #10

32.Guanxin Danshen dropping pills

CNKI

(TKA=‘冠心丹参滴丸’ + ‘冠心丹参’) and ((TKA=‘心肌梗死’ + ‘心梗’ + ‘心肌梗塞’ + ‘急性冠脉综合征’+ ‘ACS’ + ‘STEMI’ + ‘NSTEMI’ + ‘胸痹’ + ‘胸痹心痛’ + ‘真心痛’ + ‘卒心痛’ + ‘心痛’ + ‘猝心痛’ + ‘厥心痛’) and (TKA=‘PCI’ + ‘PTCA’ + ‘经皮冠状动脉介入治疗术’ + ‘经皮冠状动脉介入’ + ‘经皮冠状动脉’ + ‘冠脉介入’ + ‘经皮冠脉’ + ‘血管成形术’ + ‘支架置入术’ + ‘冠状动脉成形术’ + ‘球囊’ + ‘支架’))

WangFang Data

主题:(“冠心丹参滴丸” OR “冠心丹参”) and ((主题:心肌梗死 OR 心梗 OR 心肌梗塞 OR 急性冠脉综合征 OR ACS OR STEMI OR NSTEMI OR 胸痹 OR 胸痹心痛 OR 真心痛 OR 卒心痛 OR 心痛 OR 猝心痛 OR 厥心痛) and 主题:(PCI OR PTCA OR 经皮冠状动脉介入治疗术 OR 经皮冠状动脉介入 OR 经皮冠状动脉 OR 冠脉介入 OR 经皮冠脉 OR 血管成形术 OR 支架置入术 OR 冠状动脉成形术 OR 球囊 OR 支架))

VIP

(M=(“冠心丹参滴丸” OR “冠心丹参”) OR R=(“冠心丹参滴丸” OR “冠心丹参”)) AND ((M=(心肌梗死 OR 心梗 OR 心肌梗塞 OR 急性冠脉综合征 OR STEMI OR NSTEMI OR 胸痹 OR 胸痹心痛 OR 真心痛 OR 卒心痛 OR 心痛 OR 猝心痛 OR 厥心痛) OR R=(心肌梗死 OR 心梗 OR 心肌梗塞 OR ACS OR STEMI OR NSTEMI OR 胸痹 OR 胸痹心痛 OR 真心痛 OR 卒心痛 OR 心痛 OR 猝心痛 OR 厥心痛)) AND (M=(PCI OR PTCA OR 经皮冠状动脉介入治疗术 OR 经皮冠状动脉介入 OR 经皮冠状动脉 OR 冠脉介入 OR 经皮冠脉 OR 血管成形术 OR 支架置入术 OR 冠状动脉成形术 OR 球囊 OR 支架) OR R=(PCI OR PTCA OR 经皮冠状动脉介入治疗术 OR 经皮冠状动脉介入 OR 经皮冠状动脉 OR 冠脉介入 OR 经皮冠脉 OR 血管成形术 OR 支架置入术 OR 冠状动脉成形术 OR 球囊 OR 支架)))

Web of Science

#1 TS=(Guanxin Danshen dropping pill* OR Guanxin Danshen)

#2 TS=(myocardial infarct* OR heart infarct* OR cardiac infarct* OR cardiovascular stroke* OR cardiogenic shock OR heart attack* OR ACS OR STEMI OR NSTEMI OR ST-segment elevation myocardial infarction OR Non-ST-segment elevation myocardial infarction OR MINOCA OR Myocardial infarction with no obstructive coronary atherosclerosis OR xiongbi OR xiongbixintong OR xiongbi Xintong OR zhenxintong OR cuxintong OR Xintong OR juexintong OR cardiovascular shock OR cardiogenic stroke*)

#3 TS=(percutaneous coronary intervention OR percutaneous coronary intervention* OR percutaneous coronary revascularization* OR coronary atherectom* OR transluminal coronary angioplast* OR transluminal coronary balloon dilation OR coronary balloon angioplast* OR PCI OR Percutaneous Transluminal Coronary Angioplast* OR PTCA OR Rotational Atherectom* OR Directional Coronary Atherectom* OR Balloon dilation OR Artery Stenting)

#4 #2 AND #3

#5 #1 AND #4

PubMed

#1 “Guanxin Danshen dropping pill*”[Title/Abstract] OR “Guanxin Danshen”[Title/Abstract]

#2 "myocardial infarction"[MeSH Terms] OR “Acute Coronary Syndrome”[MeSH Terms] OR "myocardial infarct*"[Title/Abstract] OR “Acute Coronary Syndrome”[Title/Abstract] OR “ACS”[Title/Abstract] OR “Acute Coronary Syndrome*”[Title/Abstract]OR “NSTE-ACS”[Title/Abstract] OR “Non–ST-Segment Elevation Acute Coronary Syndrome*”[Title/Abstract] OR "heart infarct*"[Title/Abstract] OR "cardiac infarct*"[Title/Abstract] OR "cardiovascular stroke*"[Title/Abstract] OR "cardiogenic shock"[Title/Abstract] OR “cardiovascular shock” [Title/Abstract] OR “cardiogenic stroke*” [Title/Abstract] OR "heart attack*"[Title/Abstract] OR "STEMI"[Title/Abstract] OR "NSTEMI"[Title/Abstract] OR “ST-segment elevation myocardial infarction”[Title/Abstract] OR “Non-ST-segment elevation myocardial infarction”[Title/Abstract] OR “MINOCA”[Title/Abstract] OR “Myocardial infarction with no obstructive coronary atherosclerosis” [Title/Abstract] OR “xiongbi” [Title/Abstract] OR “xiongbixintong” [Title/Abstract] OR “xiongbi xintong” [Title/Abstract] OR “zhenxintong” [Title/Abstract] OR “cuxintong” [Title/Abstract] OR “Xintong” [Title/Abstract] OR “juexintong” [Title/Abstract]

#3 “percutaneous coronary intervention” [MeSH Terms] OR “percutaneous coronary intervention*”[Title/Abstract] OR “percutaneous coronary revascularization*” [Title/Abstract] OR “coronary atherectom*”[Title/Abstract] OR “transluminal coronary angioplast*”[Title/Abstract] OR “transluminal coronary balloon dilation”[Title/Abstract] OR “coronary balloon angioplast*”[Title/Abstract] OR “PCI”[Title/Abstract] OR “Percutaneous Transluminal Coronary Angioplast*”[Title/Abstract] OR “PTCA”[Title/Abstract] OR “Rotational Atherectom*”[Title/Abstract] OR

“Directional Coronary Atherectom*”[Title/Abstract] OR “Balloon dilation”[Title/Abstract] OR “Artery Stenting”[Title/Abstract]

#4 #2 AND #3

#1 AND #4

Embase

#1 'Guanxin Danshen dropping pill*':ab,ti OR ‘Guanxin Danshen’:ab,ti

#2 'heart infarction'/exp

#3 'Acute Coronary Syndrome'/exp

#4 'cardiac infarct':ab,ti OR 'cardiac infarction':ab,ti OR 'cardial infarct':ab,ti OR 'heart attack':ab,ti OR 'heart infarct':ab,ti OR 'heart micro infarction':ab,ti OR 'heart muscle infarction':ab,ti OR 'infarction, heart':ab,ti OR 'myocardial infarct':ab,ti OR 'myocardial infarction':ab,ti OR 'myocardium infarct':ab,ti OR 'myocardium infarction':ab,ti OR 'premonitory infarction sign':ab,ti OR 'second heart attack':ab,ti OR 'subendocardial infarction':ab,ti OR 'transmural cardiac infarction':ab,ti OR 'transmural heart infarction':ab,ti OR 'transmural infarction, heart':ab,ti OR 'heart infarction':ab,ti OR ‘Acute Coronary Syndrome’:ab,ti OR ‘ACS’:ab,ti OR ‘Acute Coronary Syndrome*’:ab,ti OR ‘NSTE-ACS’:ab,ti OR ‘Non–ST-Segment Elevation Acute Coronary Syndrome*’:ab,ti OR 'STEMI':ab,ti OR 'NSTEMI':ab,ti OR ‘ST-segment elevation myocardial infarction’:ab,ti OR ‘Non-ST-segment elevation myocardial infarction’:ab,ti OR ‘MINOCA’:ab,ti OR ‘Myocardial infarction with no obstructive coronary atherosclerosis’:ab,ti OR ‘xiongbi’:ab,ti OR ‘xiongbixintong’:ab,ti OR ‘xiongbi Xintong’:ab,ti OR ‘zhenxintong’:ab,ti OR ‘cuxintong’:ab,ti OR ‘Xintong’:ab,ti OR ‘juexintong’:ab,ti OR ‘cardiovascular shock’:ab,ti OR ‘cardiogenic stroke*’:ab,ti

#5 ‘percutaneous coronary intervention'/exp

#6 ‘percutaneous coronary intervention*':ab,ti OR 'percutaneous coronary revascularization*':ab,ti OR 'coronary atherectom*':ab,ti OR 'transluminal coronary angioplast*':ab,ti OR 'transluminal coronary balloon dilation':ab,ti OR 'coronary balloon angioplast*':ab,ti OR 'PCI':ab,ti OR 'Percutaneous Transluminal Coronary Angioplast*':ab,ti OR 'PTCA':ab,ti OR 'Rotational Atherectom*':ab,ti OR 'Directional Coronary Atherectom*':ab,ti OR 'Balloon dilation':ab,ti OR 'Artery Stenting’:ab,ti

#7 #2 OR #3

#8 #4 OR #7

#9 #5 OR #6

#10 #8 AND #9

#11 #1 AND #10

Cochrane

#1 (Guanxin Danshen dropping pill*):ti,ab,kw OR (Guanxin Danshen):ti,ab,kw

#2 MeSH descriptor: [Myocardial Infarction] explode all trees

#3 MeSH descriptor: [Acute Coronary Syndrome] explode all trees

#4 (myocardial infarct*):ti,ab,kw OR (heart infarct*):ti,ab,kw OR (cardiac infarct*):ti,ab,kw OR (cardiovascular stroke*):ti,ab,kw OR (cardiogenic shock):ti,ab,kw OR (heart attack*):ti,ab,kw OR (Acute Coronary Syndrome):ti,ab,kw OR (ACS):ti,ab,kw OR (Acute Coronary Syndrome*):ti,ab,kw OR (NSTE-ACS):ti,ab,kw OR (Non–ST-Segment Elevation Acute Coronary Syndrome*):ti,ab,kw OR (STEMI):ti,ab,kw OR (NSTEMI):ti,ab,kw OR (ST-segment elevation myocardial infarction):ti,ab,kw OR (Non-ST-segment elevation myocardial infarction):ti,ab,kw OR (MINOCA):ti,ab,kw OR (Myocardial infarction with no obstructive coronary atherosclerosis):ti,ab,kw OR (xiongbi):ti,ab,kw OR (xiongbixintong):ti,ab,kw OR (xiongbi Xintong):ti,ab,kw OR (zhenxintong):ti,ab,kw OR (cuxintong):ti,ab,kw OR (Xintong):ti,ab,kw OR (juexintong):ti,ab,kw OR (cardiovascular shock):ti,ab,kw OR (cardiogenic stroke*):ti,ab,kw

#5 MeSH descriptor: [Percutaneous Coronary Intervention] explode all trees

#6 (percutaneous coronary intervention):ti,ab,kw OR (percutaneous coronary intervention*):ti,ab,kw OR (percutaneous coronary revascularization*):ti,ab,kw OR (coronary atherectom*):ti,ab,kw OR (transluminal coronary angioplast*):ti,ab,kw OR (transluminal coronary balloon dilation):ti,ab,kw OR (coronary balloon angioplast*):ti,ab,kw OR (PCI):ti,ab,kw OR (Percutaneous Transluminal Coronary Angioplast*):ti,ab,kw OR (PTCA):ti,ab,kw OR (Rotational Atherectom*):ti,ab,kw OR (Directional Coronary Atherectom*):ti,ab,kw OR (Balloon dilation):ti,ab,kw OR (Artery Stenting):ti,ab,kw

#7 #2 OR #3

#8 #4 OR #7

#9 #5 OR #6

#10 #8 AND #9

#11 #1 AND #10

33.guanxinshutong capsules

CNKI

(TKA=‘冠心舒通胶囊’ + ‘冠心舒通’) and ((TKA=‘心肌梗死’ + ‘心梗’ + ‘心肌梗塞’ + ‘急性冠脉综合征’+ ‘ACS’ + ‘STEMI’ + ‘NSTEMI’ + ‘胸痹’ + ‘胸痹心痛’ + ‘真心痛’ + ‘卒心痛’ + ‘心痛’ + ‘猝心痛’ + ‘厥心痛’) and (TKA=‘PCI’ + ‘PTCA’ + ‘经皮冠状动脉介入治疗术’ + ‘经皮冠状动脉介入’ + ‘经皮冠状动脉’ + ‘冠脉介入’ + ‘经皮冠脉’ + ‘血管成形术’ + ‘支架置入术’ + ‘冠状动脉成形术’ + ‘球囊’ + ‘支架’))

WangFang Data

主题:(“冠心舒通胶囊” OR “冠心舒通”) and ((主题:心肌梗死 OR 心梗 OR 心肌梗塞 OR 急性冠脉综合征 OR ACS OR STEMI OR NSTEMI OR 胸痹 OR 胸痹心痛 OR 真心痛 OR 卒心痛 OR 心痛 OR 猝心痛 OR 厥心痛) and 主题:(PCI OR PTCA OR 经皮冠状动脉介入治疗术 OR 经皮冠状动脉介入 OR 经皮冠状动脉 OR 冠脉介入 OR 经皮冠脉 OR 血管成形术 OR 支架置入术 OR 冠状动脉成形术 OR 球囊 OR 支架))

VIP

(M=(“冠心舒通胶囊” OR “冠心舒通”) OR R=(“冠心舒通胶囊” OR “冠心舒通”)) AND ((M=(心肌梗死 OR 心梗 OR 心肌梗塞 OR 急性冠脉综合征 OR STEMI OR NSTEMI OR 胸痹 OR 胸痹心痛 OR 真心痛 OR 卒心痛 OR 心痛 OR 猝心痛 OR 厥心痛) OR R=(心肌梗死 OR 心梗 OR 心肌梗塞 OR ACS OR STEMI OR NSTEMI OR 胸痹 OR 胸痹心痛 OR 真心痛 OR 卒心痛 OR 心痛 OR 猝心痛 OR 厥心痛)) AND (M=(PCI OR PTCA OR 经皮冠状动脉介入治疗术 OR 经皮冠状动脉介入 OR 经皮冠状动脉 OR 冠脉介入 OR 经皮冠脉 OR 血管成形术 OR 支架置入术 OR 冠状动脉成形术 OR 球囊 OR 支架) OR R=(PCI OR PTCA OR 经皮冠状动脉介入治疗术 OR 经皮冠状动脉介入 OR 经皮冠状动脉 OR 冠脉介入 OR 经皮冠脉 OR 血管成形术 OR 支架置入术 OR 冠状动脉成形术 OR 球囊 OR 支架)))

Web of Science

#1 TS=(guanxinshutong capsule* OR guanxin shutong capsule* OR guanxinshutong OR guanxin shutong)

#2 TS=(myocardial infarct* OR heart infarct* OR cardiac infarct* OR cardiovascular stroke* OR cardiogenic shock OR heart attack* OR ACS OR STEMI OR NSTEMI OR ST-segment elevation myocardial infarction OR Non-ST-segment elevation myocardial infarction OR MINOCA OR Myocardial infarction with no obstructive coronary atherosclerosis OR xiongbi OR xiongbixintong OR xiongbi Xintong OR zhenxintong OR cuxintong OR Xintong OR juexintong OR cardiovascular shock OR cardiogenic stroke*)

#3 TS=(percutaneous coronary intervention OR percutaneous coronary intervention* OR percutaneous coronary revascularization* OR coronary atherectom* OR transluminal coronary angioplast* OR transluminal coronary balloon dilation OR coronary balloon angioplast* OR PCI OR Percutaneous Transluminal Coronary Angioplast* OR PTCA OR Rotational Atherectom* OR Directional Coronary Atherectom* OR Balloon dilation OR Artery Stenting)

#4 #2 AND #3

#5 #1 AND #4

PubMed

#1 “guanxinshutong capsule*”[Title/Abstract] OR “guanxin shutong capsule*”[Title/Abstract] OR “guanxinshutong”[Title/Abstract] OR “guanxin shutong”[Title/Abstract]

#2 "myocardial infarction"[MeSH Terms] OR “Acute Coronary Syndrome”[MeSH Terms] OR "myocardial infarct*"[Title/Abstract] OR “Acute Coronary Syndrome”[Title/Abstract] OR “ACS”[Title/Abstract] OR “Acute Coronary Syndrome*”[Title/Abstract]OR “NSTE-ACS”[Title/Abstract] OR “Non–ST-Segment Elevation Acute Coronary Syndrome*”[Title/Abstract] OR "heart infarct*"[Title/Abstract] OR "cardiac infarct*"[Title/Abstract] OR "cardiovascular stroke*"[Title/Abstract] OR "cardiogenic shock"[Title/Abstract] OR “cardiovascular shock” [Title/Abstract] OR “cardiogenic stroke*” [Title/Abstract] OR "heart attack*"[Title/Abstract] OR "STEMI"[Title/Abstract] OR "NSTEMI"[Title/Abstract] OR “ST-segment elevation myocardial infarction”[Title/Abstract] OR “Non-ST-segment elevation myocardial infarction”[Title/Abstract] OR “MINOCA”[Title/Abstract] OR “Myocardial infarction with no obstructive coronary atherosclerosis” [Title/Abstract] OR “xiongbi” [Title/Abstract] OR “xiongbixintong” [Title/Abstract] OR “xiongbi xintong” [Title/Abstract] OR “zhenxintong” [Title/Abstract] OR “cuxintong” [Title/Abstract] OR “Xintong” [Title/Abstract] OR “juexintong” [Title/Abstract]

#3 “percutaneous coronary intervention” [MeSH Terms] OR “percutaneous coronary intervention*”[Title/Abstract] OR “percutaneous coronary revascularization*” [Title/Abstract] OR “coronary atherectom*”[Title/Abstract] OR “transluminal coronary angioplast*”[Title/Abstract] OR “transluminal coronary balloon dilation”[Title/Abstract] OR “coronary balloon angioplast*”[Title/Abstract] OR “PCI”[Title/Abstract] OR “Percutaneous Transluminal Coronary Angioplast*”[Title/Abstract] OR “PTCA”[Title/Abstract] OR “Rotational Atherectom*”[Title/Abstract] OR

“Directional Coronary Atherectom*”[Title/Abstract] OR “Balloon dilation”[Title/Abstract] OR “Artery Stenting”[Title/Abstract]

#4 #2 AND #3

#1 AND #4

Embase

#1 'guanxinshutong capsule*':ab,ti OR ‘guanxin shutong capsule*’:ab,ti OR ‘guanxinshutong’:ab,ti OR ‘guanxin shutong':ab,ti

#2 'heart infarction'/exp

#3 'Acute Coronary Syndrome'/exp

#4 'cardiac infarct':ab,ti OR 'cardiac infarction':ab,ti OR 'cardial infarct':ab,ti OR 'heart attack':ab,ti OR 'heart infarct':ab,ti OR 'heart micro infarction':ab,ti OR 'heart muscle infarction':ab,ti OR 'infarction, heart':ab,ti OR 'myocardial infarct':ab,ti OR 'myocardial infarction':ab,ti OR 'myocardium infarct':ab,ti OR 'myocardium infarction':ab,ti OR 'premonitory infarction sign':ab,ti OR 'second heart attack':ab,ti OR 'subendocardial infarction':ab,ti OR 'transmural cardiac infarction':ab,ti OR 'transmural heart infarction':ab,ti OR 'transmural infarction, heart':ab,ti OR 'heart infarction':ab,ti OR ‘Acute Coronary Syndrome’:ab,ti OR ‘ACS’:ab,ti OR ‘Acute Coronary Syndrome*’:ab,ti OR ‘NSTE-ACS’:ab,ti OR ‘Non–ST-Segment Elevation Acute Coronary Syndrome*’:ab,ti OR 'STEMI':ab,ti OR 'NSTEMI':ab,ti OR ‘ST-segment elevation myocardial infarction’:ab,ti OR ‘Non-ST-segment elevation myocardial infarction’:ab,ti OR ‘MINOCA’:ab,ti OR ‘Myocardial infarction with no obstructive coronary atherosclerosis’:ab,ti OR ‘xiongbi’:ab,ti OR ‘xiongbixintong’:ab,ti OR ‘xiongbi Xintong’:ab,ti OR ‘zhenxintong’:ab,ti OR ‘cuxintong’:ab,ti OR ‘Xintong’:ab,ti OR ‘juexintong’:ab,ti OR ‘cardiovascular shock’:ab,ti OR ‘cardiogenic stroke*’:ab,ti

#5 ‘percutaneous coronary intervention'/exp

#6 ‘percutaneous coronary intervention*':ab,ti OR 'percutaneous coronary revascularization*':ab,ti OR 'coronary atherectom*':ab,ti OR 'transluminal coronary angioplast*':ab,ti OR 'transluminal coronary balloon dilation':ab,ti OR 'coronary balloon angioplast*':ab,ti OR 'PCI':ab,ti OR 'Percutaneous Transluminal Coronary Angioplast*':ab,ti OR 'PTCA':ab,ti OR 'Rotational Atherectom*':ab,ti OR 'Directional Coronary Atherectom*':ab,ti OR 'Balloon dilation':ab,ti OR 'Artery Stenting’:ab,ti

#7 #2 OR #3

#8 #4 OR #7

#9 #5 OR #6

#10 #8 AND #9

#11 #1 AND #10

Cochrane

#1 (guanxinshutong capsule*):ti,ab,kw OR (guanxin shutong capsule*):ti,ab,kw OR (guanxinshutong):ti,ab,kw OR (guanxin shutong):ti,ab,kw

#2 MeSH descriptor: [Myocardial Infarction] explode all trees

#3 MeSH descriptor: [Acute Coronary Syndrome] explode all trees

#4 (myocardial infarct*):ti,ab,kw OR (heart infarct*):ti,ab,kw OR (cardiac infarct*):ti,ab,kw OR (cardiovascular stroke*):ti,ab,kw OR (cardiogenic shock):ti,ab,kw OR (heart attack*):ti,ab,kw OR (Acute Coronary Syndrome):ti,ab,kw OR (ACS):ti,ab,kw OR (Acute Coronary Syndrome*):ti,ab,kw OR (NSTE-ACS):ti,ab,kw OR (Non–ST-Segment Elevation Acute Coronary Syndrome*):ti,ab,kw OR (STEMI):ti,ab,kw OR (NSTEMI):ti,ab,kw OR (ST-segment elevation myocardial infarction):ti,ab,kw OR (Non-ST-segment elevation myocardial infarction):ti,ab,kw OR (MINOCA):ti,ab,kw OR (Myocardial infarction with no obstructive coronary atherosclerosis):ti,ab,kw OR (xiongbi):ti,ab,kw OR (xiongbixintong):ti,ab,kw OR (xiongbi Xintong):ti,ab,kw OR (zhenxintong):ti,ab,kw OR (cuxintong):ti,ab,kw OR (Xintong):ti,ab,kw OR (juexintong):ti,ab,kw OR (cardiovascular shock):ti,ab,kw OR (cardiogenic stroke*):ti,ab,kw

#5 MeSH descriptor: [Percutaneous Coronary Intervention] explode all trees

#6 (percutaneous coronary intervention):ti,ab,kw OR (percutaneous coronary intervention*):ti,ab,kw OR (percutaneous coronary revascularization*):ti,ab,kw OR (coronary atherectom*):ti,ab,kw OR (transluminal coronary angioplast*):ti,ab,kw OR (transluminal coronary balloon dilation):ti,ab,kw OR (coronary balloon angioplast*):ti,ab,kw OR (PCI):ti,ab,kw OR (Percutaneous Transluminal Coronary Angioplast*):ti,ab,kw OR (PTCA):ti,ab,kw OR (Rotational Atherectom*):ti,ab,kw OR (Directional Coronary Atherectom*):ti,ab,kw OR (Balloon dilation):ti,ab,kw OR (Artery Stenting):ti,ab,kw

#7 #2 OR #3

#8 #4 OR #7

#9 #5 OR #6

#10 #8 AND #9

#11 #1 AND #10

34.Guanxinjing capsules

CNKI

(TKA=‘冠心静胶囊’ + ‘冠心静’) and ((TKA=‘心肌梗死’ + ‘心梗’ + ‘心肌梗塞’ + ‘急性冠脉综合征’+ ‘ACS’ + ‘STEMI’ + ‘NSTEMI’ + ‘胸痹’ + ‘胸痹心痛’ + ‘真心痛’ + ‘卒心痛’ + ‘心痛’ + ‘猝心痛’ + ‘厥心痛’) and (TKA=‘PCI’ + ‘PTCA’ + ‘经皮冠状动脉介入治疗术’ + ‘经皮冠状动脉介入’ + ‘经皮冠状动脉’ + ‘冠脉介入’ + ‘经皮冠脉’ + ‘血管成形术’ + ‘支架置入术’ + ‘冠状动脉成形术’ + ‘球囊’ + ‘支架’))

WangFang Data

主题:(“冠心静胶囊” OR “冠心静”) and ((主题:心肌梗死 OR 心梗 OR 心肌梗塞 OR 急性冠脉综合征 OR ACS OR STEMI OR NSTEMI OR 胸痹 OR 胸痹心痛 OR 真心痛 OR 卒心痛 OR 心痛 OR 猝心痛 OR 厥心痛) and 主题:(PCI OR PTCA OR 经皮冠状动脉介入治疗术 OR 经皮冠状动脉介入 OR 经皮冠状动脉 OR 冠脉介入 OR 经皮冠脉 OR 血管成形术 OR 支架置入术 OR 冠状动脉成形术 OR 球囊 OR 支架))

VIP

(M=(“冠心静胶囊” OR “冠心静”) OR R=(“冠心静胶囊” OR “冠心静”)) AND ((M=(心肌梗死 OR 心梗 OR 心肌梗塞 OR 急性冠脉综合征 OR STEMI OR NSTEMI OR 胸痹 OR 胸痹心痛 OR 真心痛 OR 卒心痛 OR 心痛 OR 猝心痛 OR 厥心痛) OR R=(心肌梗死 OR 心梗 OR 心肌梗塞 OR ACS OR STEMI OR NSTEMI OR 胸痹 OR 胸痹心痛 OR 真心痛 OR 卒心痛 OR 心痛 OR 猝心痛 OR 厥心痛)) AND (M=(PCI OR PTCA OR 经皮冠状动脉介入治疗术 OR 经皮冠状动脉介入 OR 经皮冠状动脉 OR 冠脉介入 OR 经皮冠脉 OR 血管成形术 OR 支架置入术 OR 冠状动脉成形术 OR 球囊 OR 支架) OR R=(PCI OR PTCA OR 经皮冠状动脉介入治疗术 OR 经皮冠状动脉介入 OR 经皮冠状动脉 OR 冠脉介入 OR 经皮冠脉 OR 血管成形术 OR 支架置入术 OR 冠状动脉成形术 OR 球囊 OR 支架)))

Web of Science

#1 TS=(guanxinjing OR guan-xin-jing capsule* OR guanxinjing capsule*)

#2 TS=(myocardial infarct* OR heart infarct* OR cardiac infarct* OR cardiovascular stroke* OR cardiogenic shock OR heart attack* OR ACS OR STEMI OR NSTEMI OR ST-segment elevation myocardial infarction OR Non-ST-segment elevation myocardial infarction OR MINOCA OR Myocardial infarction with no obstructive coronary atherosclerosis OR xiongbi OR xiongbixintong OR xiongbi Xintong OR zhenxintong OR cuxintong OR Xintong OR juexintong OR cardiovascular shock OR cardiogenic stroke*)

#3 TS=(percutaneous coronary intervention OR percutaneous coronary intervention* OR percutaneous coronary revascularization* OR coronary atherectom* OR transluminal coronary angioplast* OR transluminal coronary balloon dilation OR coronary balloon angioplast* OR PCI OR Percutaneous Transluminal Coronary Angioplast* OR PTCA OR Rotational Atherectom* OR Directional Coronary Atherectom* OR Balloon dilation OR Artery Stenting)

#4 #2 AND #3

#5 #1 AND #4

PubMed

#1 “guanxinjing”[Title/Abstract] OR “guan-xin-jing capsule*”[Title/Abstract] OR “guanxinjing capsule*”[Title/Abstract]

#2 "myocardial infarction"[MeSH Terms] OR “Acute Coronary Syndrome”[MeSH Terms] OR "myocardial infarct*"[Title/Abstract] OR “Acute Coronary Syndrome”[Title/Abstract] OR “ACS”[Title/Abstract] OR “Acute Coronary Syndrome*”[Title/Abstract]OR “NSTE-ACS”[Title/Abstract] OR “Non–ST-Segment Elevation Acute Coronary Syndrome*”[Title/Abstract] OR "heart infarct*"[Title/Abstract] OR "cardiac infarct*"[Title/Abstract] OR "cardiovascular stroke*"[Title/Abstract] OR "cardiogenic shock"[Title/Abstract] OR “cardiovascular shock” [Title/Abstract] OR “cardiogenic stroke*” [Title/Abstract] OR "heart attack*"[Title/Abstract] OR "STEMI"[Title/Abstract] OR "NSTEMI"[Title/Abstract] OR “ST-segment elevation myocardial infarction”[Title/Abstract] OR “Non-ST-segment elevation myocardial infarction”[Title/Abstract] OR “MINOCA”[Title/Abstract] OR “Myocardial infarction with no obstructive coronary atherosclerosis” [Title/Abstract] OR “xiongbi” [Title/Abstract] OR “xiongbixintong” [Title/Abstract] OR “xiongbi xintong” [Title/Abstract] OR “zhenxintong” [Title/Abstract] OR “cuxintong” [Title/Abstract] OR “Xintong” [Title/Abstract] OR “juexintong” [Title/Abstract]

#3 “percutaneous coronary intervention” [MeSH Terms] OR “percutaneous coronary intervention*”[Title/Abstract] OR “percutaneous coronary revascularization*” [Title/Abstract] OR “coronary atherectom*”[Title/Abstract] OR “transluminal coronary angioplast*”[Title/Abstract] OR “transluminal coronary balloon dilation”[Title/Abstract] OR “coronary balloon angioplast*”[Title/Abstract] OR “PCI”[Title/Abstract] OR “Percutaneous Transluminal Coronary Angioplast*”[Title/Abstract] OR “PTCA”[Title/Abstract] OR “Rotational Atherectom*”[Title/Abstract] OR

“Directional Coronary Atherectom*”[Title/Abstract] OR “Balloon dilation”[Title/Abstract] OR “Artery Stenting”[Title/Abstract]

#4 #2 AND #3

#1 AND #4

Embase

#1 'guanxinjing':ab,ti OR ‘guan-xin-jing capsule*’:ab,ti OR ‘guanxinjing capsule*’:ab,ti

#2 'heart infarction'/exp

#3 'Acute Coronary Syndrome'/exp

#4 'cardiac infarct':ab,ti OR 'cardiac infarction':ab,ti OR 'cardial infarct':ab,ti OR 'heart attack':ab,ti OR 'heart infarct':ab,ti OR 'heart micro infarction':ab,ti OR 'heart muscle infarction':ab,ti OR 'infarction, heart':ab,ti OR 'myocardial infarct':ab,ti OR 'myocardial infarction':ab,ti OR 'myocardium infarct':ab,ti OR 'myocardium infarction':ab,ti OR 'premonitory infarction sign':ab,ti OR 'second heart attack':ab,ti OR 'subendocardial infarction':ab,ti OR 'transmural cardiac infarction':ab,ti OR 'transmural heart infarction':ab,ti OR 'transmural infarction, heart':ab,ti OR 'heart infarction':ab,ti OR ‘Acute Coronary Syndrome’:ab,ti OR ‘ACS’:ab,ti OR ‘Acute Coronary Syndrome*’:ab,ti OR ‘NSTE-ACS’:ab,ti OR ‘Non–ST-Segment Elevation Acute Coronary Syndrome*’:ab,ti OR 'STEMI':ab,ti OR 'NSTEMI':ab,ti OR ‘ST-segment elevation myocardial infarction’:ab,ti OR ‘Non-ST-segment elevation myocardial infarction’:ab,ti OR ‘MINOCA’:ab,ti OR ‘Myocardial infarction with no obstructive coronary atherosclerosis’:ab,ti OR ‘xiongbi’:ab,ti OR ‘xiongbixintong’:ab,ti OR ‘xiongbi Xintong’:ab,ti OR ‘zhenxintong’:ab,ti OR ‘cuxintong’:ab,ti OR ‘Xintong’:ab,ti OR ‘juexintong’:ab,ti OR ‘cardiovascular shock’:ab,ti OR ‘cardiogenic stroke*’:ab,ti

#5 ‘percutaneous coronary intervention'/exp

#6 ‘percutaneous coronary intervention*':ab,ti OR 'percutaneous coronary revascularization*':ab,ti OR 'coronary atherectom*':ab,ti OR 'transluminal coronary angioplast*':ab,ti OR 'transluminal coronary balloon dilation':ab,ti OR 'coronary balloon angioplast*':ab,ti OR 'PCI':ab,ti OR 'Percutaneous Transluminal Coronary Angioplast*':ab,ti OR 'PTCA':ab,ti OR 'Rotational Atherectom*':ab,ti OR 'Directional Coronary Atherectom*':ab,ti OR 'Balloon dilation':ab,ti OR 'Artery Stenting’:ab,ti

#7 #2 OR #3

#8 #4 OR #7

#9 #5 OR #6

#10 #8 AND #9

#11 #1 AND #10

Cochrane

#1 (guanxinjing):ti,ab,kw OR (guan-xin-jing capsule*):ti,ab,kw OR (guanxinjing capsule*):ti,ab,kw

#2 MeSH descriptor: [Myocardial Infarction] explode all trees

#3 MeSH descriptor: [Acute Coronary Syndrome] explode all trees

#4 (myocardial infarct*):ti,ab,kw OR (heart infarct*):ti,ab,kw OR (cardiac infarct*):ti,ab,kw OR (cardiovascular stroke*):ti,ab,kw OR (cardiogenic shock):ti,ab,kw OR (heart attack*):ti,ab,kw OR (Acute Coronary Syndrome):ti,ab,kw OR (ACS):ti,ab,kw OR (Acute Coronary Syndrome*):ti,ab,kw OR (NSTE-ACS):ti,ab,kw OR (Non–ST-Segment Elevation Acute Coronary Syndrome*):ti,ab,kw OR (STEMI):ti,ab,kw OR (NSTEMI):ti,ab,kw OR (ST-segment elevation myocardial infarction):ti,ab,kw OR (Non-ST-segment elevation myocardial infarction):ti,ab,kw OR (MINOCA):ti,ab,kw OR (Myocardial infarction with no obstructive coronary atherosclerosis):ti,ab,kw OR (xiongbi):ti,ab,kw OR (xiongbixintong):ti,ab,kw OR (xiongbi Xintong):ti,ab,kw OR (zhenxintong):ti,ab,kw OR (cuxintong):ti,ab,kw OR (Xintong):ti,ab,kw OR (juexintong):ti,ab,kw OR (cardiovascular shock):ti,ab,kw OR (cardiogenic stroke*):ti,ab,kw

#5 MeSH descriptor: [Percutaneous Coronary Intervention] explode all trees

#6 (percutaneous coronary intervention):ti,ab,kw OR (percutaneous coronary intervention*):ti,ab,kw OR (percutaneous coronary revascularization*):ti,ab,kw OR (coronary atherectom*):ti,ab,kw OR (transluminal coronary angioplast*):ti,ab,kw OR (transluminal coronary balloon dilation):ti,ab,kw OR (coronary balloon angioplast*):ti,ab,kw OR (PCI):ti,ab,kw OR (Percutaneous Transluminal Coronary Angioplast*):ti,ab,kw OR (PTCA):ti,ab,kw OR (Rotational Atherectom*):ti,ab,kw OR (Directional Coronary Atherectom*):ti,ab,kw OR (Balloon dilation):ti,ab,kw OR (Artery Stenting):ti,ab,kw

#7 #2 OR #3

#8 #4 OR #7

#9 #5 OR #6

#10 #8 AND #9

#11 #1 AND #10

35.Suxiao Jiuxin pills

CNKI

(TKA=‘速效救心丸’ + ‘速效救心’) and ((TKA=‘心肌梗死’ + ‘心梗’ + ‘心肌梗塞’ + ‘急性冠脉综合征’+ ‘ACS’ + ‘STEMI’ + ‘NSTEMI’ + ‘胸痹’ + ‘胸痹心痛’ + ‘真心痛’ + ‘卒心痛’ + ‘心痛’ + ‘猝心痛’ + ‘厥心痛’) and (TKA=‘PCI’ + ‘PTCA’ + ‘经皮冠状动脉介入治疗术’ + ‘经皮冠状动脉介入’ + ‘经皮冠状动脉’ + ‘冠脉介入’ + ‘经皮冠脉’ + ‘血管成形术’ + ‘支架置入术’ + ‘冠状动脉成形术’ + ‘球囊’ + ‘支架’))

WangFang Data

主题:(“速效救心丸” OR “速效救心”) and ((主题:心肌梗死 OR 心梗 OR 心肌梗塞 OR 急性冠脉综合征 OR ACS OR STEMI OR NSTEMI OR 胸痹 OR 胸痹心痛 OR 真心痛 OR 卒心痛 OR 心痛 OR 猝心痛 OR 厥心痛) and 主题:(PCI OR PTCA OR 经皮冠状动脉介入治疗术 OR 经皮冠状动脉介入 OR 经皮冠状动脉 OR 冠脉介入 OR 经皮冠脉 OR 血管成形术 OR 支架置入术 OR 冠状动脉成形术 OR 球囊 OR 支架))

VIP

(M=(“速效救心丸” OR “速效救心”) OR R=(“速效救心丸” OR “速效救心”)) AND ((M=(心肌梗死 OR 心梗 OR 心肌梗塞 OR 急性冠脉综合征 OR STEMI OR NSTEMI OR 胸痹 OR 胸痹心痛 OR 真心痛 OR 卒心痛 OR 心痛 OR 猝心痛 OR 厥心痛) OR R=(心肌梗死 OR 心梗 OR 心肌梗塞 OR ACS OR STEMI OR NSTEMI OR 胸痹 OR 胸痹心痛 OR 真心痛 OR 卒心痛 OR 心痛 OR 猝心痛 OR 厥心痛)) AND (M=(PCI OR PTCA OR 经皮冠状动脉介入治疗术 OR 经皮冠状动脉介入 OR 经皮冠状动脉 OR 冠脉介入 OR 经皮冠脉 OR 血管成形术 OR 支架置入术 OR 冠状动脉成形术 OR 球囊 OR 支架) OR R=(PCI OR PTCA OR 经皮冠状动脉介入治疗术 OR 经皮冠状动脉介入 OR 经皮冠状动脉 OR 冠脉介入 OR 经皮冠脉 OR 血管成形术 OR 支架置入术 OR 冠状动脉成形术 OR 球囊 OR 支架)))

Web of Science

#1 TS=(Suxiao Jiuxin pill* OR Suxiao Jiuxin Wan OR suxiaojiuxin OR suxiao jiuxin)

#2 TS=(myocardial infarct* OR heart infarct* OR cardiac infarct* OR cardiovascular stroke* OR cardiogenic shock OR heart attack* OR ACS OR STEMI OR NSTEMI OR ST-segment elevation myocardial infarction OR Non-ST-segment elevation myocardial infarction OR MINOCA OR Myocardial infarction with no obstructive coronary atherosclerosis OR xiongbi OR xiongbixintong OR xiongbi Xintong OR zhenxintong OR cuxintong OR Xintong OR juexintong OR cardiovascular shock OR cardiogenic stroke*)

#3 TS=(percutaneous coronary intervention OR percutaneous coronary intervention* OR percutaneous coronary revascularization* OR coronary atherectom* OR transluminal coronary angioplast* OR transluminal coronary balloon dilation OR coronary balloon angioplast* OR PCI OR Percutaneous Transluminal Coronary Angioplast* OR PTCA OR Rotational Atherectom* OR Directional Coronary Atherectom* OR Balloon dilation OR Artery Stenting)

#4 #2 AND #3

#5 #1 AND #4

PubMed

#1 “Suxiao Jiuxin pill*”[Title/Abstract] OR “Suxiao Jiuxin Wan”[Title/Abstract] OR “suxiaojiuxin”[Title/Abstract] OR “suxiao jiuxin”[Title/Abstract]

#2 "myocardial infarction"[MeSH Terms] OR “Acute Coronary Syndrome”[MeSH Terms] OR "myocardial infarct*"[Title/Abstract] OR “Acute Coronary Syndrome”[Title/Abstract] OR “ACS”[Title/Abstract] OR “Acute Coronary Syndrome*”[Title/Abstract]OR “NSTE-ACS”[Title/Abstract] OR “Non–ST-Segment Elevation Acute Coronary Syndrome*”[Title/Abstract] OR "heart infarct*"[Title/Abstract] OR "cardiac infarct*"[Title/Abstract] OR "cardiovascular stroke*"[Title/Abstract] OR "cardiogenic shock"[Title/Abstract] OR “cardiovascular shock” [Title/Abstract] OR “cardiogenic stroke*” [Title/Abstract] OR "heart attack*"[Title/Abstract] OR "STEMI"[Title/Abstract] OR "NSTEMI"[Title/Abstract] OR “ST-segment elevation myocardial infarction”[Title/Abstract] OR “Non-ST-segment elevation myocardial infarction”[Title/Abstract] OR “MINOCA”[Title/Abstract] OR “Myocardial infarction with no obstructive coronary atherosclerosis” [Title/Abstract] OR “xiongbi” [Title/Abstract] OR “xiongbixintong” [Title/Abstract] OR “xiongbi xintong” [Title/Abstract] OR “zhenxintong” [Title/Abstract] OR “cuxintong” [Title/Abstract] OR “Xintong” [Title/Abstract] OR “juexintong” [Title/Abstract]

#3 “percutaneous coronary intervention” [MeSH Terms] OR “percutaneous coronary intervention*”[Title/Abstract] OR “percutaneous coronary revascularization*” [Title/Abstract] OR “coronary atherectom*”[Title/Abstract] OR “transluminal coronary angioplast*”[Title/Abstract] OR “transluminal coronary balloon dilation”[Title/Abstract] OR “coronary balloon angioplast*”[Title/Abstract] OR “PCI”[Title/Abstract] OR “Percutaneous Transluminal Coronary Angioplast*”[Title/Abstract] OR “PTCA”[Title/Abstract] OR “Rotational Atherectom*”[Title/Abstract] OR

“Directional Coronary Atherectom*”[Title/Abstract] OR “Balloon dilation”[Title/Abstract] OR “Artery Stenting”[Title/Abstract]

#4 #2 AND #3

#1 AND #4

Embase

#1 'Suxiao Jiuxin pill*':ab,ti OR ‘Suxiao Jiuxin Wan’:ab,ti OR ‘suxiaojiuxin’:ab,ti OR ‘suxiao jiuxin':ab,ti

#2 'heart infarction'/exp

#3 'Acute Coronary Syndrome'/exp

#4 'cardiac infarct':ab,ti OR 'cardiac infarction':ab,ti OR 'cardial infarct':ab,ti OR 'heart attack':ab,ti OR 'heart infarct':ab,ti OR 'heart micro infarction':ab,ti OR 'heart muscle infarction':ab,ti OR 'infarction, heart':ab,ti OR 'myocardial infarct':ab,ti OR 'myocardial infarction':ab,ti OR 'myocardium infarct':ab,ti OR 'myocardium infarction':ab,ti OR 'premonitory infarction sign':ab,ti OR 'second heart attack':ab,ti OR 'subendocardial infarction':ab,ti OR 'transmural cardiac infarction':ab,ti OR 'transmural heart infarction':ab,ti OR 'transmural infarction, heart':ab,ti OR 'heart infarction':ab,ti OR ‘Acute Coronary Syndrome’:ab,ti OR ‘ACS’:ab,ti OR ‘Acute Coronary Syndrome*’:ab,ti OR ‘NSTE-ACS’:ab,ti OR ‘Non–ST-Segment Elevation Acute Coronary Syndrome*’:ab,ti OR 'STEMI':ab,ti OR 'NSTEMI':ab,ti OR ‘ST-segment elevation myocardial infarction’:ab,ti OR ‘Non-ST-segment elevation myocardial infarction’:ab,ti OR ‘MINOCA’:ab,ti OR ‘Myocardial infarction with no obstructive coronary atherosclerosis’:ab,ti OR ‘xiongbi’:ab,ti OR ‘xiongbixintong’:ab,ti OR ‘xiongbi Xintong’:ab,ti OR ‘zhenxintong’:ab,ti OR ‘cuxintong’:ab,ti OR ‘Xintong’:ab,ti OR ‘juexintong’:ab,ti OR ‘cardiovascular shock’:ab,ti OR ‘cardiogenic stroke*’:ab,ti

#5 ‘percutaneous coronary intervention'/exp

#6 ‘percutaneous coronary intervention*':ab,ti OR 'percutaneous coronary revascularization*':ab,ti OR 'coronary atherectom*':ab,ti OR 'transluminal coronary angioplast*':ab,ti OR 'transluminal coronary balloon dilation':ab,ti OR 'coronary balloon angioplast*':ab,ti OR 'PCI':ab,ti OR 'Percutaneous Transluminal Coronary Angioplast*':ab,ti OR 'PTCA':ab,ti OR 'Rotational Atherectom*':ab,ti OR 'Directional Coronary Atherectom*':ab,ti OR 'Balloon dilation':ab,ti OR 'Artery Stenting’:ab,ti

#7 #2 OR #3

#8 #4 OR #7

#9 #5 OR #6

#10 #8 AND #9

#11 #1 AND #10

Cochrane

#1 (Suxiao Jiuxin pill*):ti,ab,kw OR (Suxiao Jiuxin Wan):ti,ab,kw OR (suxiaojiuxin):ti,ab,kw OR (suxiao jiuxin):ti,ab,kw

#2 MeSH descriptor: [Myocardial Infarction] explode all trees

#3 MeSH descriptor: [Acute Coronary Syndrome] explode all trees

#4 (myocardial infarct*):ti,ab,kw OR (heart infarct*):ti,ab,kw OR (cardiac infarct*):ti,ab,kw OR (cardiovascular stroke*):ti,ab,kw OR (cardiogenic shock):ti,ab,kw OR (heart attack*):ti,ab,kw OR (Acute Coronary Syndrome):ti,ab,kw OR (ACS):ti,ab,kw OR (Acute Coronary Syndrome*):ti,ab,kw OR (NSTE-ACS):ti,ab,kw OR (Non–ST-Segment Elevation Acute Coronary Syndrome*):ti,ab,kw OR (STEMI):ti,ab,kw OR (NSTEMI):ti,ab,kw OR (ST-segment elevation myocardial infarction):ti,ab,kw OR (Non-ST-segment elevation myocardial infarction):ti,ab,kw OR (MINOCA):ti,ab,kw OR (Myocardial infarction with no obstructive coronary atherosclerosis):ti,ab,kw OR (xiongbi):ti,ab,kw OR (xiongbixintong):ti,ab,kw OR (xiongbi Xintong):ti,ab,kw OR (zhenxintong):ti,ab,kw OR (cuxintong):ti,ab,kw OR (Xintong):ti,ab,kw OR (juexintong):ti,ab,kw OR (cardiovascular shock):ti,ab,kw OR (cardiogenic stroke*):ti,ab,kw

#5 MeSH descriptor: [Percutaneous Coronary Intervention] explode all trees

#6 (percutaneous coronary intervention):ti,ab,kw OR (percutaneous coronary intervention*):ti,ab,kw OR (percutaneous coronary revascularization*):ti,ab,kw OR (coronary atherectom*):ti,ab,kw OR (transluminal coronary angioplast*):ti,ab,kw OR (transluminal coronary balloon dilation):ti,ab,kw OR (coronary balloon angioplast*):ti,ab,kw OR (PCI):ti,ab,kw OR (Percutaneous Transluminal Coronary Angioplast*):ti,ab,kw OR (PTCA):ti,ab,kw OR (Rotational Atherectom*):ti,ab,kw OR (Directional Coronary Atherectom*):ti,ab,kw OR (Balloon dilation):ti,ab,kw OR (Artery Stenting):ti,ab,kw

#7 #2 OR #3

#8 #4 OR #7

#9 #5 OR #6

#10 #8 AND #9

#11 #1 AND #10

36.Naoxintong Capsules

CNKI

(TKA=‘脑心通胶囊’ + ‘脑心通’) and ((TKA=‘心肌梗死’ + ‘心梗’ + ‘心肌梗塞’ + ‘急性冠脉综合征’+ ‘ACS’ + ‘STEMI’ + ‘NSTEMI’ + ‘胸痹’ + ‘胸痹心痛’ + ‘真心痛’ + ‘卒心痛’ + ‘心痛’ + ‘猝心痛’ + ‘厥心痛’) and (TKA=‘PCI’ + ‘PTCA’ + ‘经皮冠状动脉介入治疗术’ + ‘经皮冠状动脉介入’ + ‘经皮冠状动脉’ + ‘冠脉介入’ + ‘经皮冠脉’ + ‘血管成形术’ + ‘支架置入术’ + ‘冠状动脉成形术’ + ‘球囊’ + ‘支架’))

WangFang Data

主题:(“脑心通胶囊” OR “脑心通”) and ((主题:心肌梗死 OR 心梗 OR 心肌梗塞 OR 急性冠脉综合征 OR ACS OR STEMI OR NSTEMI OR 胸痹 OR 胸痹心痛 OR 真心痛 OR 卒心痛 OR 心痛 OR 猝心痛 OR 厥心痛) and 主题:(PCI OR PTCA OR 经皮冠状动脉介入治疗术 OR 经皮冠状动脉介入 OR 经皮冠状动脉 OR 冠脉介入 OR 经皮冠脉 OR 血管成形术 OR 支架置入术 OR 冠状动脉成形术 OR 球囊 OR 支架))

VIP

(M=(“脑心通胶囊” OR “脑心通”) OR R=(“脑心通胶囊” OR “脑心通”)) AND ((M=(心肌梗死 OR 心梗 OR 心肌梗塞 OR 急性冠脉综合征 OR STEMI OR NSTEMI OR 胸痹 OR 胸痹心痛 OR 真心痛 OR 卒心痛 OR 心痛 OR 猝心痛 OR 厥心痛) OR R=(心肌梗死 OR 心梗 OR 心肌梗塞 OR ACS OR STEMI OR NSTEMI OR 胸痹 OR 胸痹心痛 OR 真心痛 OR 卒心痛 OR 心痛 OR 猝心痛 OR 厥心痛)) AND (M=(PCI OR PTCA OR 经皮冠状动脉介入治疗术 OR 经皮冠状动脉介入 OR 经皮冠状动脉 OR 冠脉介入 OR 经皮冠脉 OR 血管成形术 OR 支架置入术 OR 冠状动脉成形术 OR 球囊 OR 支架) OR R=(PCI OR PTCA OR 经皮冠状动脉介入治疗术 OR 经皮冠状动脉介入 OR 经皮冠状动脉 OR 冠脉介入 OR 经皮冠脉 OR 血管成形术 OR 支架置入术 OR 冠状动脉成形术 OR 球囊 OR 支架)))

Web of Science

#1 TS=(Naoxintong Capsule* OR naoxintong)

#2 TS=(myocardial infarct* OR heart infarct* OR cardiac infarct* OR cardiovascular stroke* OR cardiogenic shock OR heart attack* OR ACS OR STEMI OR NSTEMI OR ST-segment elevation myocardial infarction OR Non-ST-segment elevation myocardial infarction OR MINOCA OR Myocardial infarction with no obstructive coronary atherosclerosis OR xiongbi OR xiongbixintong OR xiongbi Xintong OR zhenxintong OR cuxintong OR Xintong OR juexintong OR cardiovascular shock OR cardiogenic stroke*)

#3 TS=(percutaneous coronary intervention OR percutaneous coronary intervention* OR percutaneous coronary revascularization* OR coronary atherectom* OR transluminal coronary angioplast* OR transluminal coronary balloon dilation OR coronary balloon angioplast* OR PCI OR Percutaneous Transluminal Coronary Angioplast* OR PTCA OR Rotational Atherectom* OR Directional Coronary Atherectom* OR Balloon dilation OR Artery Stenting)

#4 #2 AND #3

#5 #1 AND #4

PubMed

#1 “Naoxintong Capsule*”[Title/Abstract] OR “naoxintong”[Title/Abstract]

#2 "myocardial infarction"[MeSH Terms] OR “Acute Coronary Syndrome”[MeSH Terms] OR "myocardial infarct*"[Title/Abstract] OR “Acute Coronary Syndrome”[Title/Abstract] OR “ACS”[Title/Abstract] OR “Acute Coronary Syndrome*”[Title/Abstract]OR “NSTE-ACS”[Title/Abstract] OR “Non–ST-Segment Elevation Acute Coronary Syndrome*”[Title/Abstract] OR "heart infarct*"[Title/Abstract] OR "cardiac infarct*"[Title/Abstract] OR "cardiovascular stroke*"[Title/Abstract] OR "cardiogenic shock"[Title/Abstract] OR “cardiovascular shock” [Title/Abstract] OR “cardiogenic stroke*” [Title/Abstract] OR "heart attack*"[Title/Abstract] OR "STEMI"[Title/Abstract] OR "NSTEMI"[Title/Abstract] OR “ST-segment elevation myocardial infarction”[Title/Abstract] OR “Non-ST-segment elevation myocardial infarction”[Title/Abstract] OR “MINOCA”[Title/Abstract] OR “Myocardial infarction with no obstructive coronary atherosclerosis” [Title/Abstract] OR “xiongbi” [Title/Abstract] OR “xiongbixintong” [Title/Abstract] OR “xiongbi xintong” [Title/Abstract] OR “zhenxintong” [Title/Abstract] OR “cuxintong” [Title/Abstract] OR “Xintong” [Title/Abstract] OR “juexintong” [Title/Abstract]

#3 “percutaneous coronary intervention” [MeSH Terms] OR “percutaneous coronary intervention*”[Title/Abstract] OR “percutaneous coronary revascularization*” [Title/Abstract] OR “coronary atherectom*”[Title/Abstract] OR “transluminal coronary angioplast*”[Title/Abstract] OR “transluminal coronary balloon dilation”[Title/Abstract] OR “coronary balloon angioplast*”[Title/Abstract] OR “PCI”[Title/Abstract] OR “Percutaneous Transluminal Coronary Angioplast*”[Title/Abstract] OR “PTCA”[Title/Abstract] OR “Rotational Atherectom*”[Title/Abstract] OR

“Directional Coronary Atherectom*”[Title/Abstract] OR “Balloon dilation”[Title/Abstract] OR “Artery Stenting”[Title/Abstract]

#4 #2 AND #3

#1 AND #4

Embase

#1 'Naoxintong Capsule*':ab,ti OR ‘naoxintong’:ab,ti

#2 'heart infarction'/exp

#3 'Acute Coronary Syndrome'/exp

#4 'cardiac infarct':ab,ti OR 'cardiac infarction':ab,ti OR 'cardial infarct':ab,ti OR 'heart attack':ab,ti OR 'heart infarct':ab,ti OR 'heart micro infarction':ab,ti OR 'heart muscle infarction':ab,ti OR 'infarction, heart':ab,ti OR 'myocardial infarct':ab,ti OR 'myocardial infarction':ab,ti OR 'myocardium infarct':ab,ti OR 'myocardium infarction':ab,ti OR 'premonitory infarction sign':ab,ti OR 'second heart attack':ab,ti OR 'subendocardial infarction':ab,ti OR 'transmural cardiac infarction':ab,ti OR 'transmural heart infarction':ab,ti OR 'transmural infarction, heart':ab,ti OR 'heart infarction':ab,ti OR ‘Acute Coronary Syndrome’:ab,ti OR ‘ACS’:ab,ti OR ‘Acute Coronary Syndrome*’:ab,ti OR ‘NSTE-ACS’:ab,ti OR ‘Non–ST-Segment Elevation Acute Coronary Syndrome*’:ab,ti OR 'STEMI':ab,ti OR 'NSTEMI':ab,ti OR ‘ST-segment elevation myocardial infarction’:ab,ti OR ‘Non-ST-segment elevation myocardial infarction’:ab,ti OR ‘MINOCA’:ab,ti OR ‘Myocardial infarction with no obstructive coronary atherosclerosis’:ab,ti OR ‘xiongbi’:ab,ti OR ‘xiongbixintong’:ab,ti OR ‘xiongbi Xintong’:ab,ti OR ‘zhenxintong’:ab,ti OR ‘cuxintong’:ab,ti OR ‘Xintong’:ab,ti OR ‘juexintong’:ab,ti OR ‘cardiovascular shock’:ab,ti OR ‘cardiogenic stroke*’:ab,ti

#5 ‘percutaneous coronary intervention'/exp

#6 ‘percutaneous coronary intervention*':ab,ti OR 'percutaneous coronary revascularization*':ab,ti OR 'coronary atherectom*':ab,ti OR 'transluminal coronary angioplast*':ab,ti OR 'transluminal coronary balloon dilation':ab,ti OR 'coronary balloon angioplast*':ab,ti OR 'PCI':ab,ti OR 'Percutaneous Transluminal Coronary Angioplast*':ab,ti OR 'PTCA':ab,ti OR 'Rotational Atherectom*':ab,ti OR 'Directional Coronary Atherectom*':ab,ti OR 'Balloon dilation':ab,ti OR 'Artery Stenting’:ab,ti

#7 #2 OR #3

#8 #4 OR #7

#9 #5 OR #6

#10 #8 AND #9

#11 #1 AND #10

Cochrane

#1 (Naoxintong Capsule*):ti,ab,kw OR (naoxintong):ti,ab,kw

#2 MeSH descriptor: [Myocardial Infarction] explode all trees

#3 MeSH descriptor: [Acute Coronary Syndrome] explode all trees

#4 (myocardial infarct*):ti,ab,kw OR (heart infarct*):ti,ab,kw OR (cardiac infarct*):ti,ab,kw OR (cardiovascular stroke*):ti,ab,kw OR (cardiogenic shock):ti,ab,kw OR (heart attack*):ti,ab,kw OR (Acute Coronary Syndrome):ti,ab,kw OR (ACS):ti,ab,kw OR (Acute Coronary Syndrome*):ti,ab,kw OR (NSTE-ACS):ti,ab,kw OR (Non–ST-Segment Elevation Acute Coronary Syndrome*):ti,ab,kw OR (STEMI):ti,ab,kw OR (NSTEMI):ti,ab,kw OR (ST-segment elevation myocardial infarction):ti,ab,kw OR (Non-ST-segment elevation myocardial infarction):ti,ab,kw OR (MINOCA):ti,ab,kw OR (Myocardial infarction with no obstructive coronary atherosclerosis):ti,ab,kw OR (xiongbi):ti,ab,kw OR (xiongbixintong):ti,ab,kw OR (xiongbi Xintong):ti,ab,kw OR (zhenxintong):ti,ab,kw OR (cuxintong):ti,ab,kw OR (Xintong):ti,ab,kw OR (juexintong):ti,ab,kw OR (cardiovascular shock):ti,ab,kw OR (cardiogenic stroke*):ti,ab,kw

#5 MeSH descriptor: [Percutaneous Coronary Intervention] explode all trees

#6 (percutaneous coronary intervention):ti,ab,kw OR (percutaneous coronary intervention*):ti,ab,kw OR (percutaneous coronary revascularization*):ti,ab,kw OR (coronary atherectom*):ti,ab,kw OR (transluminal coronary angioplast*):ti,ab,kw OR (transluminal coronary balloon dilation):ti,ab,kw OR (coronary balloon angioplast*):ti,ab,kw OR (PCI):ti,ab,kw OR (Percutaneous Transluminal Coronary Angioplast*):ti,ab,kw OR (PTCA):ti,ab,kw OR (Rotational Atherectom*):ti,ab,kw OR (Directional Coronary Atherectom*):ti,ab,kw OR (Balloon dilation):ti,ab,kw OR (Artery Stenting):ti,ab,kw

#7 #2 OR #3

#8 #4 OR #7

#9 #5 OR #6

#10 #8 AND #9

#11 #1 AND #10

37.Naoxinqing tablets

CNKI

(TKA=‘脑心清片’ + ‘脑心清’) and ((TKA=‘心肌梗死’ + ‘心梗’ + ‘心肌梗塞’ + ‘急性冠脉综合征’+ ‘ACS’ + ‘STEMI’ + ‘NSTEMI’ + ‘胸痹’ + ‘胸痹心痛’ + ‘真心痛’ + ‘卒心痛’ + ‘心痛’ + ‘猝心痛’ + ‘厥心痛’) and (TKA=‘PCI’ + ‘PTCA’ + ‘经皮冠状动脉介入治疗术’ + ‘经皮冠状动脉介入’ + ‘经皮冠状动脉’ + ‘冠脉介入’ + ‘经皮冠脉’ + ‘血管成形术’ + ‘支架置入术’ + ‘冠状动脉成形术’ + ‘球囊’ + ‘支架’))

WangFang Data

主题:(“脑心清片” OR “脑心清”) and ((主题:心肌梗死 OR 心梗 OR 心肌梗塞 OR 急性冠脉综合征 OR ACS OR STEMI OR NSTEMI OR 胸痹 OR 胸痹心痛 OR 真心痛 OR 卒心痛 OR 心痛 OR 猝心痛 OR 厥心痛) and 主题:(PCI OR PTCA OR 经皮冠状动脉介入治疗术 OR 经皮冠状动脉介入 OR 经皮冠状动脉 OR 冠脉介入 OR 经皮冠脉 OR 血管成形术 OR 支架置入术 OR 冠状动脉成形术 OR 球囊 OR 支架))

VIP

(M=(“脑心清片” OR “脑心清”) OR R=(“脑心清片” OR “脑心清”)) AND ((M=(心肌梗死 OR 心梗 OR 心肌梗塞 OR 急性冠脉综合征 OR STEMI OR NSTEMI OR 胸痹 OR 胸痹心痛 OR 真心痛 OR 卒心痛 OR 心痛 OR 猝心痛 OR 厥心痛) OR R=(心肌梗死 OR 心梗 OR 心肌梗塞 OR ACS OR STEMI OR NSTEMI OR 胸痹 OR 胸痹心痛 OR 真心痛 OR 卒心痛 OR 心痛 OR 猝心痛 OR 厥心痛)) AND (M=(PCI OR PTCA OR 经皮冠状动脉介入治疗术 OR 经皮冠状动脉介入 OR 经皮冠状动脉 OR 冠脉介入 OR 经皮冠脉 OR 血管成形术 OR 支架置入术 OR 冠状动脉成形术 OR 球囊 OR 支架) OR R=(PCI OR PTCA OR 经皮冠状动脉介入治疗术 OR 经皮冠状动脉介入 OR 经皮冠状动脉 OR 冠脉介入 OR 经皮冠脉 OR 血管成形术 OR 支架置入术 OR 冠状动脉成形术 OR 球囊 OR 支架)))

Web of Science

#1 TS=(Naoxinqing tablet* OR naoxinqing)

#2 TS=(myocardial infarct* OR heart infarct* OR cardiac infarct* OR cardiovascular stroke* OR cardiogenic shock OR heart attack* OR ACS OR STEMI OR NSTEMI OR ST-segment elevation myocardial infarction OR Non-ST-segment elevation myocardial infarction OR MINOCA OR Myocardial infarction with no obstructive coronary atherosclerosis OR xiongbi OR xiongbixintong OR xiongbi Xintong OR zhenxintong OR cuxintong OR Xintong OR juexintong OR cardiovascular shock OR cardiogenic stroke*)

#3 TS=(percutaneous coronary intervention OR percutaneous coronary intervention* OR percutaneous coronary revascularization* OR coronary atherectom* OR transluminal coronary angioplast* OR transluminal coronary balloon dilation OR coronary balloon angioplast* OR PCI OR Percutaneous Transluminal Coronary Angioplast* OR PTCA OR Rotational Atherectom* OR Directional Coronary Atherectom* OR Balloon dilation OR Artery Stenting)

#4 #2 AND #3

#5 #1 AND #4

PubMed

#1 “Naoxinqing tablet*”[Title/Abstract] OR “naoxinqing”[Title/Abstract]

#2 "myocardial infarction"[MeSH Terms] OR “Acute Coronary Syndrome”[MeSH Terms] OR "myocardial infarct*"[Title/Abstract] OR “Acute Coronary Syndrome”[Title/Abstract] OR “ACS”[Title/Abstract] OR “Acute Coronary Syndrome*”[Title/Abstract]OR “NSTE-ACS”[Title/Abstract] OR “Non–ST-Segment Elevation Acute Coronary Syndrome*”[Title/Abstract] OR "heart infarct*"[Title/Abstract] OR "cardiac infarct*"[Title/Abstract] OR "cardiovascular stroke*"[Title/Abstract] OR "cardiogenic shock"[Title/Abstract] OR “cardiovascular shock” [Title/Abstract] OR “cardiogenic stroke*” [Title/Abstract] OR "heart attack*"[Title/Abstract] OR "STEMI"[Title/Abstract] OR "NSTEMI"[Title/Abstract] OR “ST-segment elevation myocardial infarction”[Title/Abstract] OR “Non-ST-segment elevation myocardial infarction”[Title/Abstract] OR “MINOCA”[Title/Abstract] OR “Myocardial infarction with no obstructive coronary atherosclerosis” [Title/Abstract] OR “xiongbi” [Title/Abstract] OR “xiongbixintong” [Title/Abstract] OR “xiongbi xintong” [Title/Abstract] OR “zhenxintong” [Title/Abstract] OR “cuxintong” [Title/Abstract] OR “Xintong” [Title/Abstract] OR “juexintong” [Title/Abstract]

#3 “percutaneous coronary intervention” [MeSH Terms] OR “percutaneous coronary intervention*”[Title/Abstract] OR “percutaneous coronary revascularization*” [Title/Abstract] OR “coronary atherectom*”[Title/Abstract] OR “transluminal coronary angioplast*”[Title/Abstract] OR “transluminal coronary balloon dilation”[Title/Abstract] OR “coronary balloon angioplast*”[Title/Abstract] OR “PCI”[Title/Abstract] OR “Percutaneous Transluminal Coronary Angioplast*”[Title/Abstract] OR “PTCA”[Title/Abstract] OR “Rotational Atherectom*”[Title/Abstract] OR

“Directional Coronary Atherectom*”[Title/Abstract] OR “Balloon dilation”[Title/Abstract] OR “Artery Stenting”[Title/Abstract]

#4 #2 AND #3

#1 AND #4

Embase

#1 'Naoxinqing tablet*':ab,ti OR ‘naoxinqing’:ab,ti

#2 'heart infarction'/exp

#3 'Acute Coronary Syndrome'/exp

#4 'cardiac infarct':ab,ti OR 'cardiac infarction':ab,ti OR 'cardial infarct':ab,ti OR 'heart attack':ab,ti OR 'heart infarct':ab,ti OR 'heart micro infarction':ab,ti OR 'heart muscle infarction':ab,ti OR 'infarction, heart':ab,ti OR 'myocardial infarct':ab,ti OR 'myocardial infarction':ab,ti OR 'myocardium infarct':ab,ti OR 'myocardium infarction':ab,ti OR 'premonitory infarction sign':ab,ti OR 'second heart attack':ab,ti OR 'subendocardial infarction':ab,ti OR 'transmural cardiac infarction':ab,ti OR 'transmural heart infarction':ab,ti OR 'transmural infarction, heart':ab,ti OR 'heart infarction':ab,ti OR ‘Acute Coronary Syndrome’:ab,ti OR ‘ACS’:ab,ti OR ‘Acute Coronary Syndrome*’:ab,ti OR ‘NSTE-ACS’:ab,ti OR ‘Non–ST-Segment Elevation Acute Coronary Syndrome*’:ab,ti OR 'STEMI':ab,ti OR 'NSTEMI':ab,ti OR ‘ST-segment elevation myocardial infarction’:ab,ti OR ‘Non-ST-segment elevation myocardial infarction’:ab,ti OR ‘MINOCA’:ab,ti OR ‘Myocardial infarction with no obstructive coronary atherosclerosis’:ab,ti OR ‘xiongbi’:ab,ti OR ‘xiongbixintong’:ab,ti OR ‘xiongbi Xintong’:ab,ti OR ‘zhenxintong’:ab,ti OR ‘cuxintong’:ab,ti OR ‘Xintong’:ab,ti OR ‘juexintong’:ab,ti OR ‘cardiovascular shock’:ab,ti OR ‘cardiogenic stroke*’:ab,ti

#5 ‘percutaneous coronary intervention'/exp

#6 ‘percutaneous coronary intervention*':ab,ti OR 'percutaneous coronary revascularization*':ab,ti OR 'coronary atherectom*':ab,ti OR 'transluminal coronary angioplast*':ab,ti OR 'transluminal coronary balloon dilation':ab,ti OR 'coronary balloon angioplast*':ab,ti OR 'PCI':ab,ti OR 'Percutaneous Transluminal Coronary Angioplast*':ab,ti OR 'PTCA':ab,ti OR 'Rotational Atherectom*':ab,ti OR 'Directional Coronary Atherectom*':ab,ti OR 'Balloon dilation':ab,ti OR 'Artery Stenting’:ab,ti

#7 #2 OR #3

#8 #4 OR #7

#9 #5 OR #6

#10 #8 AND #9

#11 #1 AND #10

Cochrane

#1 (Naoxinqing tablet*):ti,ab,kw OR (naoxinqing):ti,ab,kw

#2 MeSH descriptor: [Myocardial Infarction] explode all trees

#3 MeSH descriptor: [Acute Coronary Syndrome] explode all trees

#4 (myocardial infarct*):ti,ab,kw OR (heart infarct*):ti,ab,kw OR (cardiac infarct*):ti,ab,kw OR (cardiovascular stroke*):ti,ab,kw OR (cardiogenic shock):ti,ab,kw OR (heart attack*):ti,ab,kw OR (Acute Coronary Syndrome):ti,ab,kw OR (ACS):ti,ab,kw OR (Acute Coronary Syndrome*):ti,ab,kw OR (NSTE-ACS):ti,ab,kw OR (Non–ST-Segment Elevation Acute Coronary Syndrome*):ti,ab,kw OR (STEMI):ti,ab,kw OR (NSTEMI):ti,ab,kw OR (ST-segment elevation myocardial infarction):ti,ab,kw OR (Non-ST-segment elevation myocardial infarction):ti,ab,kw OR (MINOCA):ti,ab,kw OR (Myocardial infarction with no obstructive coronary atherosclerosis):ti,ab,kw OR (xiongbi):ti,ab,kw OR (xiongbixintong):ti,ab,kw OR (xiongbi Xintong):ti,ab,kw OR (zhenxintong):ti,ab,kw OR (cuxintong):ti,ab,kw OR (Xintong):ti,ab,kw OR (juexintong):ti,ab,kw OR (cardiovascular shock):ti,ab,kw OR (cardiogenic stroke*):ti,ab,kw

#5 MeSH descriptor: [Percutaneous Coronary Intervention] explode all trees

#6 (percutaneous coronary intervention):ti,ab,kw OR (percutaneous coronary intervention*):ti,ab,kw OR (percutaneous coronary revascularization*):ti,ab,kw OR (coronary atherectom*):ti,ab,kw OR (transluminal coronary angioplast*):ti,ab,kw OR (transluminal coronary balloon dilation):ti,ab,kw OR (coronary balloon angioplast*):ti,ab,kw OR (PCI):ti,ab,kw OR (Percutaneous Transluminal Coronary Angioplast*):ti,ab,kw OR (PTCA):ti,ab,kw OR (Rotational Atherectom*):ti,ab,kw OR (Directional Coronary Atherectom*):ti,ab,kw OR (Balloon dilation):ti,ab,kw OR (Artery Stenting):ti,ab,kw

#7 #2 OR #3

#8 #4 OR #7

#9 #5 OR #6

#10 #8 AND #9

#11 #1 AND #10

38.Yixinshu capsules

CNKI

(TKA=‘益心舒胶囊’ + ‘益心舒’) and ((TKA=‘心肌梗死’ + ‘心梗’ + ‘心肌梗塞’ + ‘急性冠脉综合征’+ ‘ACS’ + ‘STEMI’ + ‘NSTEMI’ + ‘胸痹’ + ‘胸痹心痛’ + ‘真心痛’ + ‘卒心痛’ + ‘心痛’ + ‘猝心痛’ + ‘厥心痛’) and (TKA=‘PCI’ + ‘PTCA’ + ‘经皮冠状动脉介入治疗术’ + ‘经皮冠状动脉介入’ + ‘经皮冠状动脉’ + ‘冠脉介入’ + ‘经皮冠脉’ + ‘血管成形术’ + ‘支架置入术’ + ‘冠状动脉成形术’ + ‘球囊’ + ‘支架’))

WangFang Data

主题:(“益心舒胶囊” OR “益心舒”) and ((主题:心肌梗死 OR 心梗 OR 心肌梗塞 OR 急性冠脉综合征 OR ACS OR STEMI OR NSTEMI OR 胸痹 OR 胸痹心痛 OR 真心痛 OR 卒心痛 OR 心痛 OR 猝心痛 OR 厥心痛) and 主题:(PCI OR PTCA OR 经皮冠状动脉介入治疗术 OR 经皮冠状动脉介入 OR 经皮冠状动脉 OR 冠脉介入 OR 经皮冠脉 OR 血管成形术 OR 支架置入术 OR 冠状动脉成形术 OR 球囊 OR 支架))

VIP

(M=(“益心舒胶囊” OR “益心舒”) OR R=(“益心舒胶囊” OR “益心舒”)) AND ((M=(心肌梗死 OR 心梗 OR 心肌梗塞 OR 急性冠脉综合征 OR STEMI OR NSTEMI OR 胸痹 OR 胸痹心痛 OR 真心痛 OR 卒心痛 OR 心痛 OR 猝心痛 OR 厥心痛) OR R=(心肌梗死 OR 心梗 OR 心肌梗塞 OR ACS OR STEMI OR NSTEMI OR 胸痹 OR 胸痹心痛 OR 真心痛 OR 卒心痛 OR 心痛 OR 猝心痛 OR 厥心痛)) AND (M=(PCI OR PTCA OR 经皮冠状动脉介入治疗术 OR 经皮冠状动脉介入 OR 经皮冠状动脉 OR 冠脉介入 OR 经皮冠脉 OR 血管成形术 OR 支架置入术 OR 冠状动脉成形术 OR 球囊 OR 支架) OR R=(PCI OR PTCA OR 经皮冠状动脉介入治疗术 OR 经皮冠状动脉介入 OR 经皮冠状动脉 OR 冠脉介入 OR 经皮冠脉 OR 血管成形术 OR 支架置入术 OR 冠状动脉成形术 OR 球囊 OR 支架)))

Web of Science

#1 TS=(Yixinshu capsule* OR yixinshu)

#2 TS=(myocardial infarct* OR heart infarct* OR cardiac infarct* OR cardiovascular stroke* OR cardiogenic shock OR heart attack* OR ACS OR STEMI OR NSTEMI OR ST-segment elevation myocardial infarction OR Non-ST-segment elevation myocardial infarction OR MINOCA OR Myocardial infarction with no obstructive coronary atherosclerosis OR xiongbi OR xiongbixintong OR xiongbi Xintong OR zhenxintong OR cuxintong OR Xintong OR juexintong OR cardiovascular shock OR cardiogenic stroke*)

#3 TS=(percutaneous coronary intervention OR percutaneous coronary intervention* OR percutaneous coronary revascularization* OR coronary atherectom* OR transluminal coronary angioplast* OR transluminal coronary balloon dilation OR coronary balloon angioplast* OR PCI OR Percutaneous Transluminal Coronary Angioplast* OR PTCA OR Rotational Atherectom* OR Directional Coronary Atherectom* OR Balloon dilation OR Artery Stenting)

#4 #2 AND #3

#5 #1 AND #4

PubMed

#1 “Yixinshu capsule*”[Title/Abstract] OR “Yixinshu”[Title/Abstract]

#2 "myocardial infarction"[MeSH Terms] OR “Acute Coronary Syndrome”[MeSH Terms] OR "myocardial infarct*"[Title/Abstract] OR “Acute Coronary Syndrome”[Title/Abstract] OR “ACS”[Title/Abstract] OR “Acute Coronary Syndrome*”[Title/Abstract]OR “NSTE-ACS”[Title/Abstract] OR “Non–ST-Segment Elevation Acute Coronary Syndrome*”[Title/Abstract] OR "heart infarct*"[Title/Abstract] OR "cardiac infarct*"[Title/Abstract] OR "cardiovascular stroke*"[Title/Abstract] OR "cardiogenic shock"[Title/Abstract] OR “cardiovascular shock” [Title/Abstract] OR “cardiogenic stroke*” [Title/Abstract] OR "heart attack*"[Title/Abstract] OR "STEMI"[Title/Abstract] OR "NSTEMI"[Title/Abstract] OR “ST-segment elevation myocardial infarction”[Title/Abstract] OR “Non-ST-segment elevation myocardial infarction”[Title/Abstract] OR “MINOCA”[Title/Abstract] OR “Myocardial infarction with no obstructive coronary atherosclerosis” [Title/Abstract] OR “xiongbi” [Title/Abstract] OR “xiongbixintong” [Title/Abstract] OR “xiongbi xintong” [Title/Abstract] OR “zhenxintong” [Title/Abstract] OR “cuxintong” [Title/Abstract] OR “Xintong” [Title/Abstract] OR “juexintong” [Title/Abstract]

#3 “percutaneous coronary intervention” [MeSH Terms] OR “percutaneous coronary intervention*”[Title/Abstract] OR “percutaneous coronary revascularization*” [Title/Abstract] OR “coronary atherectom*”[Title/Abstract] OR “transluminal coronary angioplast*”[Title/Abstract] OR “transluminal coronary balloon dilation”[Title/Abstract] OR “coronary balloon angioplast*”[Title/Abstract] OR “PCI”[Title/Abstract] OR “Percutaneous Transluminal Coronary Angioplast*”[Title/Abstract] OR “PTCA”[Title/Abstract] OR “Rotational Atherectom*”[Title/Abstract] OR

“Directional Coronary Atherectom*”[Title/Abstract] OR “Balloon dilation”[Title/Abstract] OR “Artery Stenting”[Title/Abstract]

#4 #2 AND #3

#1 AND #4

Embase

#1 'Yixinshu capsule*':ab,ti OR ‘Yixinshu’:ab,ti

#2 'heart infarction'/exp

#3 'Acute Coronary Syndrome'/exp

#4 'cardiac infarct':ab,ti OR 'cardiac infarction':ab,ti OR 'cardial infarct':ab,ti OR 'heart attack':ab,ti OR 'heart infarct':ab,ti OR 'heart micro infarction':ab,ti OR 'heart muscle infarction':ab,ti OR 'infarction, heart':ab,ti OR 'myocardial infarct':ab,ti OR 'myocardial infarction':ab,ti OR 'myocardium infarct':ab,ti OR 'myocardium infarction':ab,ti OR 'premonitory infarction sign':ab,ti OR 'second heart attack':ab,ti OR 'subendocardial infarction':ab,ti OR 'transmural cardiac infarction':ab,ti OR 'transmural heart infarction':ab,ti OR 'transmural infarction, heart':ab,ti OR 'heart infarction':ab,ti OR ‘Acute Coronary Syndrome’:ab,ti OR ‘ACS’:ab,ti OR ‘Acute Coronary Syndrome*’:ab,ti OR ‘NSTE-ACS’:ab,ti OR ‘Non–ST-Segment Elevation Acute Coronary Syndrome*’:ab,ti OR 'STEMI':ab,ti OR 'NSTEMI':ab,ti OR ‘ST-segment elevation myocardial infarction’:ab,ti OR ‘Non-ST-segment elevation myocardial infarction’:ab,ti OR ‘MINOCA’:ab,ti OR ‘Myocardial infarction with no obstructive coronary atherosclerosis’:ab,ti OR ‘xiongbi’:ab,ti OR ‘xiongbixintong’:ab,ti OR ‘xiongbi Xintong’:ab,ti OR ‘zhenxintong’:ab,ti OR ‘cuxintong’:ab,ti OR ‘Xintong’:ab,ti OR ‘juexintong’:ab,ti OR ‘cardiovascular shock’:ab,ti OR ‘cardiogenic stroke*’:ab,ti

#5 ‘percutaneous coronary intervention'/exp

#6 ‘percutaneous coronary intervention*':ab,ti OR 'percutaneous coronary revascularization*':ab,ti OR 'coronary atherectom*':ab,ti OR 'transluminal coronary angioplast*':ab,ti OR 'transluminal coronary balloon dilation':ab,ti OR 'coronary balloon angioplast*':ab,ti OR 'PCI':ab,ti OR 'Percutaneous Transluminal Coronary Angioplast*':ab,ti OR 'PTCA':ab,ti OR 'Rotational Atherectom*':ab,ti OR 'Directional Coronary Atherectom*':ab,ti OR 'Balloon dilation':ab,ti OR 'Artery Stenting’:ab,ti

#7 #2 OR #3

#8 #4 OR #7

#9 #5 OR #6

#10 #8 AND #9

#11 #1 AND #10

Cochrane

#1 (Yixinshu capsule*):ti,ab,kw OR (Yixinshu):ti,ab,kw

#2 MeSH descriptor: [Myocardial Infarction] explode all trees

#3 MeSH descriptor: [Acute Coronary Syndrome] explode all trees

#4 (myocardial infarct*):ti,ab,kw OR (heart infarct*):ti,ab,kw OR (cardiac infarct*):ti,ab,kw OR (cardiovascular stroke*):ti,ab,kw OR (cardiogenic shock):ti,ab,kw OR (heart attack*):ti,ab,kw OR (Acute Coronary Syndrome):ti,ab,kw OR (ACS):ti,ab,kw OR (Acute Coronary Syndrome*):ti,ab,kw OR (NSTE-ACS):ti,ab,kw OR (Non–ST-Segment Elevation Acute Coronary Syndrome*):ti,ab,kw OR (STEMI):ti,ab,kw OR (NSTEMI):ti,ab,kw OR (ST-segment elevation myocardial infarction):ti,ab,kw OR (Non-ST-segment elevation myocardial infarction):ti,ab,kw OR (MINOCA):ti,ab,kw OR (Myocardial infarction with no obstructive coronary atherosclerosis):ti,ab,kw OR (xiongbi):ti,ab,kw OR (xiongbixintong):ti,ab,kw OR (xiongbi Xintong):ti,ab,kw OR (zhenxintong):ti,ab,kw OR (cuxintong):ti,ab,kw OR (Xintong):ti,ab,kw OR (juexintong):ti,ab,kw OR (cardiovascular shock):ti,ab,kw OR (cardiogenic stroke*):ti,ab,kw

#5 MeSH descriptor: [Percutaneous Coronary Intervention] explode all trees

#6 (percutaneous coronary intervention):ti,ab,kw OR (percutaneous coronary intervention*):ti,ab,kw OR (percutaneous coronary revascularization*):ti,ab,kw OR (coronary atherectom*):ti,ab,kw OR (transluminal coronary angioplast*):ti,ab,kw OR (transluminal coronary balloon dilation):ti,ab,kw OR (coronary balloon angioplast*):ti,ab,kw OR (PCI):ti,ab,kw OR (Percutaneous Transluminal Coronary Angioplast*):ti,ab,kw OR (PTCA):ti,ab,kw OR (Rotational Atherectom*):ti,ab,kw OR (Directional Coronary Atherectom*):ti,ab,kw OR (Balloon dilation):ti,ab,kw OR (Artery Stenting):ti,ab,kw

#7 #2 OR #3

#8 #4 OR #7

#9 #5 OR #6

#10 #8 AND #9

#11 #1 AND #10

39.Nuodikang capsules

CNKI

(TKA=‘诺迪康胶囊’ + ‘诺迪康’) and ((TKA=‘心肌梗死’ + ‘心梗’ + ‘心肌梗塞’ + ‘急性冠脉综合征’+ ‘ACS’ + ‘STEMI’ + ‘NSTEMI’ + ‘胸痹’ + ‘胸痹心痛’ + ‘真心痛’ + ‘卒心痛’ + ‘心痛’ + ‘猝心痛’ + ‘厥心痛’) and (TKA=‘PCI’ + ‘PTCA’ + ‘经皮冠状动脉介入治疗术’ + ‘经皮冠状动脉介入’ + ‘经皮冠状动脉’ + ‘冠脉介入’ + ‘经皮冠脉’ + ‘血管成形术’ + ‘支架置入术’ + ‘冠状动脉成形术’ + ‘球囊’ + ‘支架’))

WangFang Data

主题:(“诺迪康胶囊” OR “诺迪康”) and ((主题:心肌梗死 OR 心梗 OR 心肌梗塞 OR 急性冠脉综合征 OR ACS OR STEMI OR NSTEMI OR 胸痹 OR 胸痹心痛 OR 真心痛 OR 卒心痛 OR 心痛 OR 猝心痛 OR 厥心痛) and 主题:(PCI OR PTCA OR 经皮冠状动脉介入治疗术 OR 经皮冠状动脉介入 OR 经皮冠状动脉 OR 冠脉介入 OR 经皮冠脉 OR 血管成形术 OR 支架置入术 OR 冠状动脉成形术 OR 球囊 OR 支架))

VIP

(M=(“诺迪康胶囊” OR “诺迪康”) OR R=(“诺迪康胶囊” OR “诺迪康”)) AND ((M=(心肌梗死 OR 心梗 OR 心肌梗塞 OR 急性冠脉综合征 OR STEMI OR NSTEMI OR 胸痹 OR 胸痹心痛 OR 真心痛 OR 卒心痛 OR 心痛 OR 猝心痛 OR 厥心痛) OR R=(心肌梗死 OR 心梗 OR 心肌梗塞 OR ACS OR STEMI OR NSTEMI OR 胸痹 OR 胸痹心痛 OR 真心痛 OR 卒心痛 OR 心痛 OR 猝心痛 OR 厥心痛)) AND (M=(PCI OR PTCA OR 经皮冠状动脉介入治疗术 OR 经皮冠状动脉介入 OR 经皮冠状动脉 OR 冠脉介入 OR 经皮冠脉 OR 血管成形术 OR 支架置入术 OR 冠状动脉成形术 OR 球囊 OR 支架) OR R=(PCI OR PTCA OR 经皮冠状动脉介入治疗术 OR 经皮冠状动脉介入 OR 经皮冠状动脉 OR 冠脉介入 OR 经皮冠脉 OR 血管成形术 OR 支架置入术 OR 冠状动脉成形术 OR 球囊 OR 支架)))

Web of Science

#1 TS=(NuoDiKangJiaoNang OR nuodikang capsule* OR nuodikang)

#2 TS=(myocardial infarct* OR heart infarct* OR cardiac infarct* OR cardiovascular stroke* OR cardiogenic shock OR heart attack* OR ACS OR STEMI OR NSTEMI OR ST-segment elevation myocardial infarction OR Non-ST-segment elevation myocardial infarction OR MINOCA OR Myocardial infarction with no obstructive coronary atherosclerosis OR xiongbi OR xiongbixintong OR xiongbi Xintong OR zhenxintong OR cuxintong OR Xintong OR juexintong OR cardiovascular shock OR cardiogenic stroke*)

#3 TS=(percutaneous coronary intervention OR percutaneous coronary intervention* OR percutaneous coronary revascularization* OR coronary atherectom* OR transluminal coronary angioplast* OR transluminal coronary balloon dilation OR coronary balloon angioplast* OR PCI OR Percutaneous Transluminal Coronary Angioplast* OR PTCA OR Rotational Atherectom* OR Directional Coronary Atherectom* OR Balloon dilation OR Artery Stenting)

#4 #2 AND #3

#5 #1 AND #4

PubMed

#1 “NuoDiKangJiaoNang”[Title/Abstract] OR “nuodikang capsule*”[Title/Abstract] OR “nudikang”[Title/Abstract]

#2 "myocardial infarction"[MeSH Terms] OR “Acute Coronary Syndrome”[MeSH Terms] OR "myocardial infarct*"[Title/Abstract] OR “Acute Coronary Syndrome”[Title/Abstract] OR “ACS”[Title/Abstract] OR “Acute Coronary Syndrome*”[Title/Abstract]OR “NSTE-ACS”[Title/Abstract] OR “Non–ST-Segment Elevation Acute Coronary Syndrome*”[Title/Abstract] OR "heart infarct*"[Title/Abstract] OR "cardiac infarct*"[Title/Abstract] OR "cardiovascular stroke*"[Title/Abstract] OR "cardiogenic shock"[Title/Abstract] OR “cardiovascular shock” [Title/Abstract] OR “cardiogenic stroke*” [Title/Abstract] OR "heart attack*"[Title/Abstract] OR "STEMI"[Title/Abstract] OR "NSTEMI"[Title/Abstract] OR “ST-segment elevation myocardial infarction”[Title/Abstract] OR “Non-ST-segment elevation myocardial infarction”[Title/Abstract] OR “MINOCA”[Title/Abstract] OR “Myocardial infarction with no obstructive coronary atherosclerosis” [Title/Abstract] OR “xiongbi” [Title/Abstract] OR “xiongbixintong” [Title/Abstract] OR “xiongbi xintong” [Title/Abstract] OR “zhenxintong” [Title/Abstract] OR “cuxintong” [Title/Abstract] OR “Xintong” [Title/Abstract] OR “juexintong” [Title/Abstract]

#3 “percutaneous coronary intervention” [MeSH Terms] OR “percutaneous coronary intervention*”[Title/Abstract] OR “percutaneous coronary revascularization*” [Title/Abstract] OR “coronary atherectom*”[Title/Abstract] OR “transluminal coronary angioplast*”[Title/Abstract] OR “transluminal coronary balloon dilation”[Title/Abstract] OR “coronary balloon angioplast*”[Title/Abstract] OR “PCI”[Title/Abstract] OR “Percutaneous Transluminal Coronary Angioplast*”[Title/Abstract] OR “PTCA”[Title/Abstract] OR “Rotational Atherectom*”[Title/Abstract] OR

“Directional Coronary Atherectom*”[Title/Abstract] OR “Balloon dilation”[Title/Abstract] OR “Artery Stenting”[Title/Abstract]

#4 #2 AND #3

#1 AND #4

Embase

#1 'NuoDiKangJiaoNang':ab,ti OR ‘nuodikang capsule*’:ab,ti OR ‘nudikang':ab,ti

#2 'heart infarction'/exp

#3 'Acute Coronary Syndrome'/exp

#4 'cardiac infarct':ab,ti OR 'cardiac infarction':ab,ti OR 'cardial infarct':ab,ti OR 'heart attack':ab,ti OR 'heart infarct':ab,ti OR 'heart micro infarction':ab,ti OR 'heart muscle infarction':ab,ti OR 'infarction, heart':ab,ti OR 'myocardial infarct':ab,ti OR 'myocardial infarction':ab,ti OR 'myocardium infarct':ab,ti OR 'myocardium infarction':ab,ti OR 'premonitory infarction sign':ab,ti OR 'second heart attack':ab,ti OR 'subendocardial infarction':ab,ti OR 'transmural cardiac infarction':ab,ti OR 'transmural heart infarction':ab,ti OR 'transmural infarction, heart':ab,ti OR 'heart infarction':ab,ti OR ‘Acute Coronary Syndrome’:ab,ti OR ‘ACS’:ab,ti OR ‘Acute Coronary Syndrome*’:ab,ti OR ‘NSTE-ACS’:ab,ti OR ‘Non–ST-Segment Elevation Acute Coronary Syndrome*’:ab,ti OR 'STEMI':ab,ti OR 'NSTEMI':ab,ti OR ‘ST-segment elevation myocardial infarction’:ab,ti OR ‘Non-ST-segment elevation myocardial infarction’:ab,ti OR ‘MINOCA’:ab,ti OR ‘Myocardial infarction with no obstructive coronary atherosclerosis’:ab,ti OR ‘xiongbi’:ab,ti OR ‘xiongbixintong’:ab,ti OR ‘xiongbi Xintong’:ab,ti OR ‘zhenxintong’:ab,ti OR ‘cuxintong’:ab,ti OR ‘Xintong’:ab,ti OR ‘juexintong’:ab,ti OR ‘cardiovascular shock’:ab,ti OR ‘cardiogenic stroke*’:ab,ti

#5 ‘percutaneous coronary intervention'/exp

#6 ‘percutaneous coronary intervention*':ab,ti OR 'percutaneous coronary revascularization*':ab,ti OR 'coronary atherectom*':ab,ti OR 'transluminal coronary angioplast*':ab,ti OR 'transluminal coronary balloon dilation':ab,ti OR 'coronary balloon angioplast*':ab,ti OR 'PCI':ab,ti OR 'Percutaneous Transluminal Coronary Angioplast*':ab,ti OR 'PTCA':ab,ti OR 'Rotational Atherectom*':ab,ti OR 'Directional Coronary Atherectom*':ab,ti OR 'Balloon dilation':ab,ti OR 'Artery Stenting’:ab,ti

#7 #2 OR #3

#8 #4 OR #7

#9 #5 OR #6

#10 #8 AND #9

#11 #1 AND #10

Cochrane

#1 (NuoDiKangJiaoNang):ti,ab,kw OR (nuodikang capsule*):ti,ab,kw OR (nudikang):ti,ab,kw

#2 MeSH descriptor: [Myocardial Infarction] explode all trees

#3 MeSH descriptor: [Acute Coronary Syndrome] explode all trees

#4 (myocardial infarct*):ti,ab,kw OR (heart infarct*):ti,ab,kw OR (cardiac infarct*):ti,ab,kw OR (cardiovascular stroke*):ti,ab,kw OR (cardiogenic shock):ti,ab,kw OR (heart attack*):ti,ab,kw OR (Acute Coronary Syndrome):ti,ab,kw OR (ACS):ti,ab,kw OR (Acute Coronary Syndrome*):ti,ab,kw OR (NSTE-ACS):ti,ab,kw OR (Non–ST-Segment Elevation Acute Coronary Syndrome*):ti,ab,kw OR (STEMI):ti,ab,kw OR (NSTEMI):ti,ab,kw OR (ST-segment elevation myocardial infarction):ti,ab,kw OR (Non-ST-segment elevation myocardial infarction):ti,ab,kw OR (MINOCA):ti,ab,kw OR (Myocardial infarction with no obstructive coronary atherosclerosis):ti,ab,kw OR (xiongbi):ti,ab,kw OR (xiongbixintong):ti,ab,kw OR (xiongbi Xintong):ti,ab,kw OR (zhenxintong):ti,ab,kw OR (cuxintong):ti,ab,kw OR (Xintong):ti,ab,kw OR (juexintong):ti,ab,kw OR (cardiovascular shock):ti,ab,kw OR (cardiogenic stroke*):ti,ab,kw

#5 MeSH descriptor: [Percutaneous Coronary Intervention] explode all trees

#6 (percutaneous coronary intervention):ti,ab,kw OR (percutaneous coronary intervention*):ti,ab,kw OR (percutaneous coronary revascularization*):ti,ab,kw OR (coronary atherectom*):ti,ab,kw OR (transluminal coronary angioplast*):ti,ab,kw OR (transluminal coronary balloon dilation):ti,ab,kw OR (coronary balloon angioplast*):ti,ab,kw OR (PCI):ti,ab,kw OR (Percutaneous Transluminal Coronary Angioplast*):ti,ab,kw OR (PTCA):ti,ab,kw OR (Rotational Atherectom*):ti,ab,kw OR (Directional Coronary Atherectom*):ti,ab,kw OR (Balloon dilation):ti,ab,kw OR (Artery Stenting):ti,ab,kw

#7 #2 OR #3

#8 #4 OR #7

#9 #5 OR #6

#10 #8 AND #9

#11 #1 AND #10

40.Tongxinluo capsules

CNKI

(TKA=‘通心络胶囊’ + ‘通心络’) and ((TKA=‘心肌梗死’ + ‘心梗’ + ‘心肌梗塞’ + ‘急性冠脉综合征’+ ‘ACS’ + ‘STEMI’ + ‘NSTEMI’ + ‘胸痹’ + ‘胸痹心痛’ + ‘真心痛’ + ‘卒心痛’ + ‘心痛’ + ‘猝心痛’ + ‘厥心痛’) and (TKA=‘PCI’ + ‘PTCA’ + ‘经皮冠状动脉介入治疗术’ + ‘经皮冠状动脉介入’ + ‘经皮冠状动脉’ + ‘冠脉介入’ + ‘经皮冠脉’ + ‘血管成形术’ + ‘支架置入术’ + ‘冠状动脉成形术’ + ‘球囊’ + ‘支架’))

WangFang Data

主题:(“通心络胶囊” OR “通心络”) and ((主题:心肌梗死 OR 心梗 OR 心肌梗塞 OR 急性冠脉综合征 OR ACS OR STEMI OR NSTEMI OR 胸痹 OR 胸痹心痛 OR 真心痛 OR 卒心痛 OR 心痛 OR 猝心痛 OR 厥心痛) and 主题:(PCI OR PTCA OR 经皮冠状动脉介入治疗术 OR 经皮冠状动脉介入 OR 经皮冠状动脉 OR 冠脉介入 OR 经皮冠脉 OR 血管成形术 OR 支架置入术 OR 冠状动脉成形术 OR 球囊 OR 支架))

VIP

(M=(“通心络胶囊” OR “通心络”) OR R=(“通心络胶囊” OR “通心络”)) AND ((M=(心肌梗死 OR 心梗 OR 心肌梗塞 OR 急性冠脉综合征 OR STEMI OR NSTEMI OR 胸痹 OR 胸痹心痛 OR 真心痛 OR 卒心痛 OR 心痛 OR 猝心痛 OR 厥心痛) OR R=(心肌梗死 OR 心梗 OR 心肌梗塞 OR ACS OR STEMI OR NSTEMI OR 胸痹 OR 胸痹心痛 OR 真心痛 OR 卒心痛 OR 心痛 OR 猝心痛 OR 厥心痛)) AND (M=(PCI OR PTCA OR 经皮冠状动脉介入治疗术 OR 经皮冠状动脉介入 OR 经皮冠状动脉 OR 冠脉介入 OR 经皮冠脉 OR 血管成形术 OR 支架置入术 OR 冠状动脉成形术 OR 球囊 OR 支架) OR R=(PCI OR PTCA OR 经皮冠状动脉介入治疗术 OR 经皮冠状动脉介入 OR 经皮冠状动脉 OR 冠脉介入 OR 经皮冠脉 OR 血管成形术 OR 支架置入术 OR 冠状动脉成形术 OR 球囊 OR 支架)))

Web of Science

#1 TS=(Tongxinluo capsule* OR tongxinluo)

#2 TS=(myocardial infarct* OR heart infarct* OR cardiac infarct* OR cardiovascular stroke* OR cardiogenic shock OR heart attack* OR ACS OR STEMI OR NSTEMI OR ST-segment elevation myocardial infarction OR Non-ST-segment elevation myocardial infarction OR MINOCA OR Myocardial infarction with no obstructive coronary atherosclerosis OR xiongbi OR xiongbixintong OR xiongbi Xintong OR zhenxintong OR cuxintong OR Xintong OR juexintong OR cardiovascular shock OR cardiogenic stroke*)

#3 TS=(percutaneous coronary intervention OR percutaneous coronary intervention* OR percutaneous coronary revascularization* OR coronary atherectom* OR transluminal coronary angioplast* OR transluminal coronary balloon dilation OR coronary balloon angioplast* OR PCI OR Percutaneous Transluminal Coronary Angioplast* OR PTCA OR Rotational Atherectom* OR Directional Coronary Atherectom* OR Balloon dilation OR Artery Stenting)

#4 #2 AND #3

#5 #1 AND #4

PubMed

#1 “Tongxinluo capsule*”[Title/Abstract] OR “tongxinluo”[Title/Abstract]

#2 "myocardial infarction"[MeSH Terms] OR “Acute Coronary Syndrome”[MeSH Terms] OR "myocardial infarct*"[Title/Abstract] OR “Acute Coronary Syndrome”[Title/Abstract] OR “ACS”[Title/Abstract] OR “Acute Coronary Syndrome*”[Title/Abstract]OR “NSTE-ACS”[Title/Abstract] OR “Non–ST-Segment Elevation Acute Coronary Syndrome*”[Title/Abstract] OR "heart infarct*"[Title/Abstract] OR "cardiac infarct*"[Title/Abstract] OR "cardiovascular stroke*"[Title/Abstract] OR "cardiogenic shock"[Title/Abstract] OR “cardiovascular shock” [Title/Abstract] OR “cardiogenic stroke*” [Title/Abstract] OR "heart attack*"[Title/Abstract] OR "STEMI"[Title/Abstract] OR "NSTEMI"[Title/Abstract] OR “ST-segment elevation myocardial infarction”[Title/Abstract] OR “Non-ST-segment elevation myocardial infarction”[Title/Abstract] OR “MINOCA”[Title/Abstract] OR “Myocardial infarction with no obstructive coronary atherosclerosis” [Title/Abstract] OR “xiongbi” [Title/Abstract] OR “xiongbixintong” [Title/Abstract] OR “xiongbi xintong” [Title/Abstract] OR “zhenxintong” [Title/Abstract] OR “cuxintong” [Title/Abstract] OR “Xintong” [Title/Abstract] OR “juexintong” [Title/Abstract]

#3 “percutaneous coronary intervention” [MeSH Terms] OR “percutaneous coronary intervention*”[Title/Abstract] OR “percutaneous coronary revascularization*” [Title/Abstract] OR “coronary atherectom*”[Title/Abstract] OR “transluminal coronary angioplast*”[Title/Abstract] OR “transluminal coronary balloon dilation”[Title/Abstract] OR “coronary balloon angioplast*”[Title/Abstract] OR “PCI”[Title/Abstract] OR “Percutaneous Transluminal Coronary Angioplast*”[Title/Abstract] OR “PTCA”[Title/Abstract] OR “Rotational Atherectom*”[Title/Abstract] OR

“Directional Coronary Atherectom*”[Title/Abstract] OR “Balloon dilation”[Title/Abstract] OR “Artery Stenting”[Title/Abstract]

#4 #2 AND #3

#1 AND #4

Embase

#1 'Tongxinluo capsule*':ab,ti OR ‘tongxinluo’:ab,ti

#2 'heart infarction'/exp

#3 'Acute Coronary Syndrome'/exp

#4 'cardiac infarct':ab,ti OR 'cardiac infarction':ab,ti OR 'cardial infarct':ab,ti OR 'heart attack':ab,ti OR 'heart infarct':ab,ti OR 'heart micro infarction':ab,ti OR 'heart muscle infarction':ab,ti OR 'infarction, heart':ab,ti OR 'myocardial infarct':ab,ti OR 'myocardial infarction':ab,ti OR 'myocardium infarct':ab,ti OR 'myocardium infarction':ab,ti OR 'premonitory infarction sign':ab,ti OR 'second heart attack':ab,ti OR 'subendocardial infarction':ab,ti OR 'transmural cardiac infarction':ab,ti OR 'transmural heart infarction':ab,ti OR 'transmural infarction, heart':ab,ti OR 'heart infarction':ab,ti OR ‘Acute Coronary Syndrome’:ab,ti OR ‘ACS’:ab,ti OR ‘Acute Coronary Syndrome*’:ab,ti OR ‘NSTE-ACS’:ab,ti OR ‘Non–ST-Segment Elevation Acute Coronary Syndrome*’:ab,ti OR 'STEMI':ab,ti OR 'NSTEMI':ab,ti OR ‘ST-segment elevation myocardial infarction’:ab,ti OR ‘Non-ST-segment elevation myocardial infarction’:ab,ti OR ‘MINOCA’:ab,ti OR ‘Myocardial infarction with no obstructive coronary atherosclerosis’:ab,ti OR ‘xiongbi’:ab,ti OR ‘xiongbixintong’:ab,ti OR ‘xiongbi Xintong’:ab,ti OR ‘zhenxintong’:ab,ti OR ‘cuxintong’:ab,ti OR ‘Xintong’:ab,ti OR ‘juexintong’:ab,ti OR ‘cardiovascular shock’:ab,ti OR ‘cardiogenic stroke*’:ab,ti

#5 ‘percutaneous coronary intervention'/exp

#6 ‘percutaneous coronary intervention*':ab,ti OR 'percutaneous coronary revascularization*':ab,ti OR 'coronary atherectom*':ab,ti OR 'transluminal coronary angioplast*':ab,ti OR 'transluminal coronary balloon dilation':ab,ti OR 'coronary balloon angioplast*':ab,ti OR 'PCI':ab,ti OR 'Percutaneous Transluminal Coronary Angioplast*':ab,ti OR 'PTCA':ab,ti OR 'Rotational Atherectom*':ab,ti OR 'Directional Coronary Atherectom*':ab,ti OR 'Balloon dilation':ab,ti OR 'Artery Stenting’:ab,ti

#7 #2 OR #3

#8 #4 OR #7

#9 #5 OR #6

#10 #8 AND #9

#11 #1 AND #10

Cochrane

#1 (Tongxinluo capsule*):ti,ab,kw OR (tongxinluo):ti,ab,kw

#2 MeSH descriptor: [Myocardial Infarction] explode all trees

#3 MeSH descriptor: [Acute Coronary Syndrome] explode all trees

#4 (myocardial infarct*):ti,ab,kw OR (heart infarct*):ti,ab,kw OR (cardiac infarct*):ti,ab,kw OR (cardiovascular stroke*):ti,ab,kw OR (cardiogenic shock):ti,ab,kw OR (heart attack*):ti,ab,kw OR (Acute Coronary Syndrome):ti,ab,kw OR (ACS):ti,ab,kw OR (Acute Coronary Syndrome*):ti,ab,kw OR (NSTE-ACS):ti,ab,kw OR (Non–ST-Segment Elevation Acute Coronary Syndrome*):ti,ab,kw OR (STEMI):ti,ab,kw OR (NSTEMI):ti,ab,kw OR (ST-segment elevation myocardial infarction):ti,ab,kw OR (Non-ST-segment elevation myocardial infarction):ti,ab,kw OR (MINOCA):ti,ab,kw OR (Myocardial infarction with no obstructive coronary atherosclerosis):ti,ab,kw OR (xiongbi):ti,ab,kw OR (xiongbixintong):ti,ab,kw OR (xiongbi Xintong):ti,ab,kw OR (zhenxintong):ti,ab,kw OR (cuxintong):ti,ab,kw OR (Xintong):ti,ab,kw OR (juexintong):ti,ab,kw OR (cardiovascular shock):ti,ab,kw OR (cardiogenic stroke*):ti,ab,kw

#5 MeSH descriptor: [Percutaneous Coronary Intervention] explode all trees

#6 (percutaneous coronary intervention):ti,ab,kw OR (percutaneous coronary intervention*):ti,ab,kw OR (percutaneous coronary revascularization*):ti,ab,kw OR (coronary atherectom*):ti,ab,kw OR (transluminal coronary angioplast*):ti,ab,kw OR (transluminal coronary balloon dilation):ti,ab,kw OR (coronary balloon angioplast*):ti,ab,kw OR (PCI):ti,ab,kw OR (Percutaneous Transluminal Coronary Angioplast*):ti,ab,kw OR (PTCA):ti,ab,kw OR (Rotational Atherectom*):ti,ab,kw OR (Directional Coronary Atherectom*):ti,ab,kw OR (Balloon dilation):ti,ab,kw OR (Artery Stenting):ti,ab,kw

#7 #2 OR #3

#8 #4 OR #7

#9 #5 OR #6

#10 #8 AND #9

#11 #1 AND #10

41.Yindan Xinnaotong soft capsules

CNKI

(TKA=‘银丹心脑通软胶囊’ + ‘银丹心脑通’) and ((TKA=‘心肌梗死’ + ‘心梗’ + ‘心肌梗塞’ + ‘急性冠脉综合征’+ ‘ACS’ + ‘STEMI’ + ‘NSTEMI’ + ‘胸痹’ + ‘胸痹心痛’ + ‘真心痛’ + ‘卒心痛’ + ‘心痛’ + ‘猝心痛’ + ‘厥心痛’) and (TKA=‘PCI’ + ‘PTCA’ + ‘经皮冠状动脉介入治疗术’ + ‘经皮冠状动脉介入’ + ‘经皮冠状动脉’ + ‘冠脉介入’ + ‘经皮冠脉’ + ‘血管成形术’ + ‘支架置入术’ + ‘冠状动脉成形术’ + ‘球囊’ + ‘支架’))

WangFang Data

主题:(“银丹心脑通软胶囊” OR “银丹心脑通”) and ((主题:心肌梗死 OR 心梗 OR 心肌梗塞 OR 急性冠脉综合征 OR ACS OR STEMI OR NSTEMI OR 胸痹 OR 胸痹心痛 OR 真心痛 OR 卒心痛 OR 心痛 OR 猝心痛 OR 厥心痛) and 主题:(PCI OR PTCA OR 经皮冠状动脉介入治疗术 OR 经皮冠状动脉介入 OR 经皮冠状动脉 OR 冠脉介入 OR 经皮冠脉 OR 血管成形术 OR 支架置入术 OR 冠状动脉成形术 OR 球囊 OR 支架))

VIP

(M=(“银丹心脑通软胶囊” OR “银丹心脑通”) OR R=(“银丹心脑通软胶囊” OR “银丹心脑通”)) AND ((M=(心肌梗死 OR 心梗 OR 心肌梗塞 OR 急性冠脉综合征 OR STEMI OR NSTEMI OR 胸痹 OR 胸痹心痛 OR 真心痛 OR 卒心痛 OR 心痛 OR 猝心痛 OR 厥心痛) OR R=(心肌梗死 OR 心梗 OR 心肌梗塞 OR ACS OR STEMI OR NSTEMI OR 胸痹 OR 胸痹心痛 OR 真心痛 OR 卒心痛 OR 心痛 OR 猝心痛 OR 厥心痛)) AND (M=(PCI OR PTCA OR 经皮冠状动脉介入治疗术 OR 经皮冠状动脉介入 OR 经皮冠状动脉 OR 冠脉介入 OR 经皮冠脉 OR 血管成形术 OR 支架置入术 OR 冠状动脉成形术 OR 球囊 OR 支架) OR R=(PCI OR PTCA OR 经皮冠状动脉介入治疗术 OR 经皮冠状动脉介入 OR 经皮冠状动脉 OR 冠脉介入 OR 经皮冠脉 OR 血管成形术 OR 支架置入术 OR 冠状动脉成形术 OR 球囊 OR 支架)))

Web of Science

#1 TS=(yindan xinnaotong OR YindanXinnaotong OR yindanxinnaotong soft capsule* OR yindan xinnaotong soft capsule*)

#2 TS=(myocardial infarct* OR heart infarct* OR cardiac infarct* OR cardiovascular stroke* OR cardiogenic shock OR heart attack* OR ACS OR STEMI OR NSTEMI OR ST-segment elevation myocardial infarction OR Non-ST-segment elevation myocardial infarction OR MINOCA OR Myocardial infarction with no obstructive coronary atherosclerosis OR xiongbi OR xiongbixintong OR xiongbi Xintong OR zhenxintong OR cuxintong OR Xintong OR juexintong OR cardiovascular shock OR cardiogenic stroke*)

#3 TS=(percutaneous coronary intervention OR percutaneous coronary intervention* OR percutaneous coronary revascularization* OR coronary atherectom* OR transluminal coronary angioplast* OR transluminal coronary balloon dilation OR coronary balloon angioplast* OR PCI OR Percutaneous Transluminal Coronary Angioplast* OR PTCA OR Rotational Atherectom* OR Directional Coronary Atherectom* OR Balloon dilation OR Artery Stenting)

#4 #2 AND #3

#5 #1 AND #4

PubMed

#1 “yindan xinnaotong”[Title/Abstract] OR “YindanXinnaotong”[Title/Abstract] OR “yindanxinnaotong soft capsule*”[Title/Abstract] OR “yindan xinnaotong soft capsule*”[Title/Abstract]

#2 "myocardial infarction"[MeSH Terms] OR “Acute Coronary Syndrome”[MeSH Terms] OR "myocardial infarct*"[Title/Abstract] OR “Acute Coronary Syndrome”[Title/Abstract] OR “ACS”[Title/Abstract] OR “Acute Coronary Syndrome*”[Title/Abstract]OR “NSTE-ACS”[Title/Abstract] OR “Non–ST-Segment Elevation Acute Coronary Syndrome*”[Title/Abstract] OR "heart infarct*"[Title/Abstract] OR "cardiac infarct*"[Title/Abstract] OR "cardiovascular stroke*"[Title/Abstract] OR "cardiogenic shock"[Title/Abstract] OR “cardiovascular shock” [Title/Abstract] OR “cardiogenic stroke*” [Title/Abstract] OR "heart attack*"[Title/Abstract] OR "STEMI"[Title/Abstract] OR "NSTEMI"[Title/Abstract] OR “ST-segment elevation myocardial infarction”[Title/Abstract] OR “Non-ST-segment elevation myocardial infarction”[Title/Abstract] OR “MINOCA”[Title/Abstract] OR “Myocardial infarction with no obstructive coronary atherosclerosis” [Title/Abstract] OR “xiongbi” [Title/Abstract] OR “xiongbixintong” [Title/Abstract] OR “xiongbi xintong” [Title/Abstract] OR “zhenxintong” [Title/Abstract] OR “cuxintong” [Title/Abstract] OR “Xintong” [Title/Abstract] OR “juexintong” [Title/Abstract]

#3 “percutaneous coronary intervention” [MeSH Terms] OR “percutaneous coronary intervention*”[Title/Abstract] OR “percutaneous coronary revascularization*” [Title/Abstract] OR “coronary atherectom*”[Title/Abstract] OR “transluminal coronary angioplast*”[Title/Abstract] OR “transluminal coronary balloon dilation”[Title/Abstract] OR “coronary balloon angioplast*”[Title/Abstract] OR “PCI”[Title/Abstract] OR “Percutaneous Transluminal Coronary Angioplast*”[Title/Abstract] OR “PTCA”[Title/Abstract] OR “Rotational Atherectom*”[Title/Abstract] OR

“Directional Coronary Atherectom*”[Title/Abstract] OR “Balloon dilation”[Title/Abstract] OR “Artery Stenting”[Title/Abstract]

#4 #2 AND #3

#1 AND #4

Embase

#1 'yindan xinnaotong':ab,ti OR ‘YindanXinnaotong’:ab,ti OR ‘yindanxinnaotong soft capsule*’:ab,ti OR ‘yindan xinnaotong soft capsule*':ab,ti

#2 'heart infarction'/exp

#3 'Acute Coronary Syndrome'/exp

#4 'cardiac infarct':ab,ti OR 'cardiac infarction':ab,ti OR 'cardial infarct':ab,ti OR 'heart attack':ab,ti OR 'heart infarct':ab,ti OR 'heart micro infarction':ab,ti OR 'heart muscle infarction':ab,ti OR 'infarction, heart':ab,ti OR 'myocardial infarct':ab,ti OR 'myocardial infarction':ab,ti OR 'myocardium infarct':ab,ti OR 'myocardium infarction':ab,ti OR 'premonitory infarction sign':ab,ti OR 'second heart attack':ab,ti OR 'subendocardial infarction':ab,ti OR 'transmural cardiac infarction':ab,ti OR 'transmural heart infarction':ab,ti OR 'transmural infarction, heart':ab,ti OR 'heart infarction':ab,ti OR ‘Acute Coronary Syndrome’:ab,ti OR ‘ACS’:ab,ti OR ‘Acute Coronary Syndrome*’:ab,ti OR ‘NSTE-ACS’:ab,ti OR ‘Non–ST-Segment Elevation Acute Coronary Syndrome*’:ab,ti OR 'STEMI':ab,ti OR 'NSTEMI':ab,ti OR ‘ST-segment elevation myocardial infarction’:ab,ti OR ‘Non-ST-segment elevation myocardial infarction’:ab,ti OR ‘MINOCA’:ab,ti OR ‘Myocardial infarction with no obstructive coronary atherosclerosis’:ab,ti OR ‘xiongbi’:ab,ti OR ‘xiongbixintong’:ab,ti OR ‘xiongbi Xintong’:ab,ti OR ‘zhenxintong’:ab,ti OR ‘cuxintong’:ab,ti OR ‘Xintong’:ab,ti OR ‘juexintong’:ab,ti OR ‘cardiovascular shock’:ab,ti OR ‘cardiogenic stroke*’:ab,ti

#5 ‘percutaneous coronary intervention'/exp

#6 ‘percutaneous coronary intervention*':ab,ti OR 'percutaneous coronary revascularization*':ab,ti OR 'coronary atherectom*':ab,ti OR 'transluminal coronary angioplast*':ab,ti OR 'transluminal coronary balloon dilation':ab,ti OR 'coronary balloon angioplast*':ab,ti OR 'PCI':ab,ti OR 'Percutaneous Transluminal Coronary Angioplast*':ab,ti OR 'PTCA':ab,ti OR 'Rotational Atherectom*':ab,ti OR 'Directional Coronary Atherectom*':ab,ti OR 'Balloon dilation':ab,ti OR 'Artery Stenting’:ab,ti

#7 #2 OR #3

#8 #4 OR #7

#9 #5 OR #6

#10 #8 AND #9

#11 #1 AND #10

Cochrane

#1 (yindan xinnaotong):ti,ab,kw OR (YindanXinnaotong):ti,ab,kw OR (yindanxinnaotong soft capsule*):ti,ab,kw OR (yindan xinnaotong soft capsule*):ti,ab,kw

#2 MeSH descriptor: [Myocardial Infarction] explode all trees

#3 MeSH descriptor: [Acute Coronary Syndrome] explode all trees

#4 (myocardial infarct*):ti,ab,kw OR (heart infarct*):ti,ab,kw OR (cardiac infarct*):ti,ab,kw OR (cardiovascular stroke*):ti,ab,kw OR (cardiogenic shock):ti,ab,kw OR (heart attack*):ti,ab,kw OR (Acute Coronary Syndrome):ti,ab,kw OR (ACS):ti,ab,kw OR (Acute Coronary Syndrome*):ti,ab,kw OR (NSTE-ACS):ti,ab,kw OR (Non–ST-Segment Elevation Acute Coronary Syndrome*):ti,ab,kw OR (STEMI):ti,ab,kw OR (NSTEMI):ti,ab,kw OR (ST-segment elevation myocardial infarction):ti,ab,kw OR (Non-ST-segment elevation myocardial infarction):ti,ab,kw OR (MINOCA):ti,ab,kw OR (Myocardial infarction with no obstructive coronary atherosclerosis):ti,ab,kw OR (xiongbi):ti,ab,kw OR (xiongbixintong):ti,ab,kw OR (xiongbi Xintong):ti,ab,kw OR (zhenxintong):ti,ab,kw OR (cuxintong):ti,ab,kw OR (Xintong):ti,ab,kw OR (juexintong):ti,ab,kw OR (cardiovascular shock):ti,ab,kw OR (cardiogenic stroke*):ti,ab,kw

#5 MeSH descriptor: [Percutaneous Coronary Intervention] explode all trees

#6 (percutaneous coronary intervention):ti,ab,kw OR (percutaneous coronary intervention*):ti,ab,kw OR (percutaneous coronary revascularization*):ti,ab,kw OR (coronary atherectom*):ti,ab,kw OR (transluminal coronary angioplast*):ti,ab,kw OR (transluminal coronary balloon dilation):ti,ab,kw OR (coronary balloon angioplast*):ti,ab,kw OR (PCI):ti,ab,kw OR (Percutaneous Transluminal Coronary Angioplast*):ti,ab,kw OR (PTCA):ti,ab,kw OR (Rotational Atherectom*):ti,ab,kw OR (Directional Coronary Atherectom*):ti,ab,kw OR (Balloon dilation):ti,ab,kw OR (Artery Stenting):ti,ab,kw

#7 #2 OR #3

#8 #4 OR #7

#9 #5 OR #6

#10 #8 AND #9

#11 #1 AND #10

42.Ginkgo biloba capsules

CNKI

(TKA=‘银杏叶胶囊’ + ‘银杏叶’) and ((TKA=‘心肌梗死’ + ‘心梗’ + ‘心肌梗塞’ + ‘急性冠脉综合征’+ ‘ACS’ + ‘STEMI’ + ‘NSTEMI’ + ‘胸痹’ + ‘胸痹心痛’ + ‘真心痛’ + ‘卒心痛’ + ‘心痛’ + ‘猝心痛’ + ‘厥心痛’) and (TKA=‘PCI’ + ‘PTCA’ + ‘经皮冠状动脉介入治疗术’ + ‘经皮冠状动脉介入’ + ‘经皮冠状动脉’ + ‘冠脉介入’ + ‘经皮冠脉’ + ‘血管成形术’ + ‘支架置入术’ + ‘冠状动脉成形术’ + ‘球囊’ + ‘支架’))

WangFang Data

主题:(“银杏叶胶囊” OR “银杏叶”) and ((主题:心肌梗死 OR 心梗 OR 心肌梗塞 OR 急性冠脉综合征 OR ACS OR STEMI OR NSTEMI OR 胸痹 OR 胸痹心痛 OR 真心痛 OR 卒心痛 OR 心痛 OR 猝心痛 OR 厥心痛) and 主题:(PCI OR PTCA OR 经皮冠状动脉介入治疗术 OR 经皮冠状动脉介入 OR 经皮冠状动脉 OR 冠脉介入 OR 经皮冠脉 OR 血管成形术 OR 支架置入术 OR 冠状动脉成形术 OR 球囊 OR 支架))

VIP

(M=(“银杏叶胶囊” OR “银杏叶”) OR R=(“银杏叶胶囊” OR “银杏叶”)) AND ((M=(心肌梗死 OR 心梗 OR 心肌梗塞 OR 急性冠脉综合征 OR STEMI OR NSTEMI OR 胸痹 OR 胸痹心痛 OR 真心痛 OR 卒心痛 OR 心痛 OR 猝心痛 OR 厥心痛) OR R=(心肌梗死 OR 心梗 OR 心肌梗塞 OR ACS OR STEMI OR NSTEMI OR 胸痹 OR 胸痹心痛 OR 真心痛 OR 卒心痛 OR 心痛 OR 猝心痛 OR 厥心痛)) AND (M=(PCI OR PTCA OR 经皮冠状动脉介入治疗术 OR 经皮冠状动脉介入 OR 经皮冠状动脉 OR 冠脉介入 OR 经皮冠脉 OR 血管成形术 OR 支架置入术 OR 冠状动脉成形术 OR 球囊 OR 支架) OR R=(PCI OR PTCA OR 经皮冠状动脉介入治疗术 OR 经皮冠状动脉介入 OR 经皮冠状动脉 OR 冠脉介入 OR 经皮冠脉 OR 血管成形术 OR 支架置入术 OR 冠状动脉成形术 OR 球囊 OR 支架)))

Web of Science

#1 TS=(Ginkgo biloba capsule* OR Ginkgo Leaf capsule* OR Ginkgo biloba OR Ginkgo Leaf)

#2 TS=(myocardial infarct* OR heart infarct* OR cardiac infarct* OR cardiovascular stroke* OR cardiogenic shock OR heart attack* OR ACS OR STEMI OR NSTEMI OR ST-segment elevation myocardial infarction OR Non-ST-segment elevation myocardial infarction OR MINOCA OR Myocardial infarction with no obstructive coronary atherosclerosis OR xiongbi OR xiongbixintong OR xiongbi Xintong OR zhenxintong OR cuxintong OR Xintong OR juexintong OR cardiovascular shock OR cardiogenic stroke*)

#3 TS=(percutaneous coronary intervention OR percutaneous coronary intervention* OR percutaneous coronary revascularization* OR coronary atherectom* OR transluminal coronary angioplast* OR transluminal coronary balloon dilation OR coronary balloon angioplast* OR PCI OR Percutaneous Transluminal Coronary Angioplast* OR PTCA OR Rotational Atherectom* OR Directional Coronary Atherectom* OR Balloon dilation OR Artery Stenting)

#4 #2 AND #3

#5 #1 AND #4

PubMed

#1 “Ginkgo biloba capsule*”[Title/Abstract] OR “Ginkgo Leaf capsule*”[Title/Abstract] OR “Ginkgo biloba”[Title/Abstract] OR “Ginkgo Leaf”[Title/Abstract]

#2 "myocardial infarction"[MeSH Terms] OR “Acute Coronary Syndrome”[MeSH Terms] OR "myocardial infarct*"[Title/Abstract] OR “Acute Coronary Syndrome”[Title/Abstract] OR “ACS”[Title/Abstract] OR “Acute Coronary Syndrome*”[Title/Abstract]OR “NSTE-ACS”[Title/Abstract] OR “Non–ST-Segment Elevation Acute Coronary Syndrome*”[Title/Abstract] OR "heart infarct*"[Title/Abstract] OR "cardiac infarct*"[Title/Abstract] OR "cardiovascular stroke*"[Title/Abstract] OR "cardiogenic shock"[Title/Abstract] OR “cardiovascular shock” [Title/Abstract] OR “cardiogenic stroke*” [Title/Abstract] OR "heart attack*"[Title/Abstract] OR "STEMI"[Title/Abstract] OR "NSTEMI"[Title/Abstract] OR “ST-segment elevation myocardial infarction”[Title/Abstract] OR “Non-ST-segment elevation myocardial infarction”[Title/Abstract] OR “MINOCA”[Title/Abstract] OR “Myocardial infarction with no obstructive coronary atherosclerosis” [Title/Abstract] OR “xiongbi” [Title/Abstract] OR “xiongbixintong” [Title/Abstract] OR “xiongbi xintong” [Title/Abstract] OR “zhenxintong” [Title/Abstract] OR “cuxintong” [Title/Abstract] OR “Xintong” [Title/Abstract] OR “juexintong” [Title/Abstract]

#3 “percutaneous coronary intervention” [MeSH Terms] OR “percutaneous coronary intervention*”[Title/Abstract] OR “percutaneous coronary revascularization*” [Title/Abstract] OR “coronary atherectom*”[Title/Abstract] OR “transluminal coronary angioplast*”[Title/Abstract] OR “transluminal coronary balloon dilation”[Title/Abstract] OR “coronary balloon angioplast*”[Title/Abstract] OR “PCI”[Title/Abstract] OR “Percutaneous Transluminal Coronary Angioplast*”[Title/Abstract] OR “PTCA”[Title/Abstract] OR “Rotational Atherectom*”[Title/Abstract] OR

“Directional Coronary Atherectom*”[Title/Abstract] OR “Balloon dilation”[Title/Abstract] OR “Artery Stenting”[Title/Abstract]

#4 #2 AND #3

#1 AND #4

Embase

#1 'Ginkgo biloba capsule*':ab,ti OR ‘Ginkgo Leaf capsule*’:ab,ti OR ‘Ginkgo biloba’:ab,ti OR ‘Ginkgo Leaf':ab,ti

#2 'heart infarction'/exp

#3 'Acute Coronary Syndrome'/exp

#4 'cardiac infarct':ab,ti OR 'cardiac infarction':ab,ti OR 'cardial infarct':ab,ti OR 'heart attack':ab,ti OR 'heart infarct':ab,ti OR 'heart micro infarction':ab,ti OR 'heart muscle infarction':ab,ti OR 'infarction, heart':ab,ti OR 'myocardial infarct':ab,ti OR 'myocardial infarction':ab,ti OR 'myocardium infarct':ab,ti OR 'myocardium infarction':ab,ti OR 'premonitory infarction sign':ab,ti OR 'second heart attack':ab,ti OR 'subendocardial infarction':ab,ti OR 'transmural cardiac infarction':ab,ti OR 'transmural heart infarction':ab,ti OR 'transmural infarction, heart':ab,ti OR 'heart infarction':ab,ti OR ‘Acute Coronary Syndrome’:ab,ti OR ‘ACS’:ab,ti OR ‘Acute Coronary Syndrome*’:ab,ti OR ‘NSTE-ACS’:ab,ti OR ‘Non–ST-Segment Elevation Acute Coronary Syndrome*’:ab,ti OR 'STEMI':ab,ti OR 'NSTEMI':ab,ti OR ‘ST-segment elevation myocardial infarction’:ab,ti OR ‘Non-ST-segment elevation myocardial infarction’:ab,ti OR ‘MINOCA’:ab,ti OR ‘Myocardial infarction with no obstructive coronary atherosclerosis’:ab,ti OR ‘xiongbi’:ab,ti OR ‘xiongbixintong’:ab,ti OR ‘xiongbi Xintong’:ab,ti OR ‘zhenxintong’:ab,ti OR ‘cuxintong’:ab,ti OR ‘Xintong’:ab,ti OR ‘juexintong’:ab,ti OR ‘cardiovascular shock’:ab,ti OR ‘cardiogenic stroke*’:ab,ti

#5 ‘percutaneous coronary intervention'/exp

#6 ‘percutaneous coronary intervention*':ab,ti OR 'percutaneous coronary revascularization*':ab,ti OR 'coronary atherectom*':ab,ti OR 'transluminal coronary angioplast*':ab,ti OR 'transluminal coronary balloon dilation':ab,ti OR 'coronary balloon angioplast*':ab,ti OR 'PCI':ab,ti OR 'Percutaneous Transluminal Coronary Angioplast*':ab,ti OR 'PTCA':ab,ti OR 'Rotational Atherectom*':ab,ti OR 'Directional Coronary Atherectom*':ab,ti OR 'Balloon dilation':ab,ti OR 'Artery Stenting’:ab,ti

#7 #2 OR #3

#8 #4 OR #7

#9 #5 OR #6

#10 #8 AND #9

#11 #1 AND #10

Cochrane

#1 (Ginkgo biloba capsule*):ti,ab,kw OR (Ginkgo Leaf capsule*):ti,ab,kw OR (Ginkgo biloba):ti,ab,kw OR (Ginkgo Leaf):ti,ab,kw

#2 MeSH descriptor: [Myocardial Infarction] explode all trees

#3 MeSH descriptor: [Acute Coronary Syndrome] explode all trees

#4 (myocardial infarct*):ti,ab,kw OR (heart infarct*):ti,ab,kw OR (cardiac infarct*):ti,ab,kw OR (cardiovascular stroke*):ti,ab,kw OR (cardiogenic shock):ti,ab,kw OR (heart attack*):ti,ab,kw OR (Acute Coronary Syndrome):ti,ab,kw OR (ACS):ti,ab,kw OR (Acute Coronary Syndrome*):ti,ab,kw OR (NSTE-ACS):ti,ab,kw OR (Non–ST-Segment Elevation Acute Coronary Syndrome*):ti,ab,kw OR (STEMI):ti,ab,kw OR (NSTEMI):ti,ab,kw OR (ST-segment elevation myocardial infarction):ti,ab,kw OR (Non-ST-segment elevation myocardial infarction):ti,ab,kw OR (MINOCA):ti,ab,kw OR (Myocardial infarction with no obstructive coronary atherosclerosis):ti,ab,kw OR (xiongbi):ti,ab,kw OR (xiongbixintong):ti,ab,kw OR (xiongbi Xintong):ti,ab,kw OR (zhenxintong):ti,ab,kw OR (cuxintong):ti,ab,kw OR (Xintong):ti,ab,kw OR (juexintong):ti,ab,kw OR (cardiovascular shock):ti,ab,kw OR (cardiogenic stroke*):ti,ab,kw

#5 MeSH descriptor: [Percutaneous Coronary Intervention] explode all trees

#6 (percutaneous coronary intervention):ti,ab,kw OR (percutaneous coronary intervention*):ti,ab,kw OR (percutaneous coronary revascularization*):ti,ab,kw OR (coronary atherectom*):ti,ab,kw OR (transluminal coronary angioplast*):ti,ab,kw OR (transluminal coronary balloon dilation):ti,ab,kw OR (coronary balloon angioplast*):ti,ab,kw OR (PCI):ti,ab,kw OR (Percutaneous Transluminal Coronary Angioplast*):ti,ab,kw OR (PTCA):ti,ab,kw OR (Rotational Atherectom*):ti,ab,kw OR (Directional Coronary Atherectom*):ti,ab,kw OR (Balloon dilation):ti,ab,kw OR (Artery Stenting):ti,ab,kw

#7 #2 OR #3

#8 #4 OR #7

#9 #5 OR #6

#10 #8 AND #9

#11 #1 AND #10

43.Ginkgo leaf drop pills

CNKI

(TKA=‘银杏叶滴丸’ + ‘银杏叶’) and ((TKA=‘心肌梗死’ + ‘心梗’ + ‘心肌梗塞’ + ‘急性冠脉综合征’+ ‘ACS’ + ‘STEMI’ + ‘NSTEMI’ + ‘胸痹’ + ‘胸痹心痛’ + ‘真心痛’ + ‘卒心痛’ + ‘心痛’ + ‘猝心痛’ + ‘厥心痛’) and (TKA=‘PCI’ + ‘PTCA’ + ‘经皮冠状动脉介入治疗术’ + ‘经皮冠状动脉介入’ + ‘经皮冠状动脉’ + ‘冠脉介入’ + ‘经皮冠脉’ + ‘血管成形术’ + ‘支架置入术’ + ‘冠状动脉成形术’ + ‘球囊’ + ‘支架’))

WangFang Data

主题:(“银杏叶滴丸” OR “银杏叶”) and ((主题:心肌梗死 OR 心梗 OR 心肌梗塞 OR 急性冠脉综合征 OR ACS OR STEMI OR NSTEMI OR 胸痹 OR 胸痹心痛 OR 真心痛 OR 卒心痛 OR 心痛 OR 猝心痛 OR 厥心痛) and 主题:(PCI OR PTCA OR 经皮冠状动脉介入治疗术 OR 经皮冠状动脉介入 OR 经皮冠状动脉 OR 冠脉介入 OR 经皮冠脉 OR 血管成形术 OR 支架置入术 OR 冠状动脉成形术 OR 球囊 OR 支架))

VIP

(M=(“银杏叶滴丸” OR “银杏叶”) OR R=(“银杏叶滴丸” OR “银杏叶”)) AND ((M=(心肌梗死 OR 心梗 OR 心肌梗塞 OR 急性冠脉综合征 OR STEMI OR NSTEMI OR 胸痹 OR 胸痹心痛 OR 真心痛 OR 卒心痛 OR 心痛 OR 猝心痛 OR 厥心痛) OR R=(心肌梗死 OR 心梗 OR 心肌梗塞 OR ACS OR STEMI OR NSTEMI OR 胸痹 OR 胸痹心痛 OR 真心痛 OR 卒心痛 OR 心痛 OR 猝心痛 OR 厥心痛)) AND (M=(PCI OR PTCA OR 经皮冠状动脉介入治疗术 OR 经皮冠状动脉介入 OR 经皮冠状动脉 OR 冠脉介入 OR 经皮冠脉 OR 血管成形术 OR 支架置入术 OR 冠状动脉成形术 OR 球囊 OR 支架) OR R=(PCI OR PTCA OR 经皮冠状动脉介入治疗术 OR 经皮冠状动脉介入 OR 经皮冠状动脉 OR 冠脉介入 OR 经皮冠脉 OR 血管成形术 OR 支架置入术 OR 冠状动脉成形术 OR 球囊 OR 支架)))

Web of Science

#1 TS=(Ginkgo leaf drop pill OR Ginkgo Biloba dropping pill OR Ginkgo biloba OR Ginkgo Leaf)

#2 TS=(myocardial infarct* OR heart infarct* OR cardiac infarct* OR cardiovascular stroke* OR cardiogenic shock OR heart attack* OR ACS OR STEMI OR NSTEMI OR ST-segment elevation myocardial infarction OR Non-ST-segment elevation myocardial infarction OR MINOCA OR Myocardial infarction with no obstructive coronary atherosclerosis OR xiongbi OR xiongbixintong OR xiongbi Xintong OR zhenxintong OR cuxintong OR Xintong OR juexintong OR cardiovascular shock OR cardiogenic stroke*)

#3 TS=(percutaneous coronary intervention OR percutaneous coronary intervention* OR percutaneous coronary revascularization* OR coronary atherectom* OR transluminal coronary angioplast* OR transluminal coronary balloon dilation OR coronary balloon angioplast* OR PCI OR Percutaneous Transluminal Coronary Angioplast* OR PTCA OR Rotational Atherectom* OR Directional Coronary Atherectom* OR Balloon dilation OR Artery Stenting)

#4 #2 AND #3

#5 #1 AND #4

PubMed

#1 “Ginkgo leaf drop pill”[Title/Abstract] OR “Ginkgo Biloba dropping pill”[Title/Abstract] OR “Ginkgo biloba”[Title/Abstract] OR “Ginkgo Leaf”[Title/Abstract]

#2 "myocardial infarction"[MeSH Terms] OR “Acute Coronary Syndrome”[MeSH Terms] OR "myocardial infarct*"[Title/Abstract] OR “Acute Coronary Syndrome”[Title/Abstract] OR “ACS”[Title/Abstract] OR “Acute Coronary Syndrome*”[Title/Abstract]OR “NSTE-ACS”[Title/Abstract] OR “Non–ST-Segment Elevation Acute Coronary Syndrome*”[Title/Abstract] OR "heart infarct*"[Title/Abstract] OR "cardiac infarct*"[Title/Abstract] OR "cardiovascular stroke*"[Title/Abstract] OR "cardiogenic shock"[Title/Abstract] OR “cardiovascular shock” [Title/Abstract] OR “cardiogenic stroke*” [Title/Abstract] OR "heart attack*"[Title/Abstract] OR "STEMI"[Title/Abstract] OR "NSTEMI"[Title/Abstract] OR “ST-segment elevation myocardial infarction”[Title/Abstract] OR “Non-ST-segment elevation myocardial infarction”[Title/Abstract] OR “MINOCA”[Title/Abstract] OR “Myocardial infarction with no obstructive coronary atherosclerosis” [Title/Abstract] OR “xiongbi” [Title/Abstract] OR “xiongbixintong” [Title/Abstract] OR “xiongbi xintong” [Title/Abstract] OR “zhenxintong” [Title/Abstract] OR “cuxintong” [Title/Abstract] OR “Xintong” [Title/Abstract] OR “juexintong” [Title/Abstract]

#3 “percutaneous coronary intervention” [MeSH Terms] OR “percutaneous coronary intervention*”[Title/Abstract] OR “percutaneous coronary revascularization*” [Title/Abstract] OR “coronary atherectom*”[Title/Abstract] OR “transluminal coronary angioplast*”[Title/Abstract] OR “transluminal coronary balloon dilation”[Title/Abstract] OR “coronary balloon angioplast*”[Title/Abstract] OR “PCI”[Title/Abstract] OR “Percutaneous Transluminal Coronary Angioplast*”[Title/Abstract] OR “PTCA”[Title/Abstract] OR “Rotational Atherectom*”[Title/Abstract] OR

“Directional Coronary Atherectom*”[Title/Abstract] OR “Balloon dilation”[Title/Abstract] OR “Artery Stenting”[Title/Abstract]

#4 #2 AND #3

#1 AND #4

Embase

#1 'Ginkgo leaf drop pill':ab,ti OR ‘Ginkgo Biloba dropping pill’:ab,ti OR ‘Ginkgo biloba’:ab,ti OR ‘Ginkgo Leaf':ab,ti

#2 'heart infarction'/exp

#3 'Acute Coronary Syndrome'/exp

#4 'cardiac infarct':ab,ti OR 'cardiac infarction':ab,ti OR 'cardial infarct':ab,ti OR 'heart attack':ab,ti OR 'heart infarct':ab,ti OR 'heart micro infarction':ab,ti OR 'heart muscle infarction':ab,ti OR 'infarction, heart':ab,ti OR 'myocardial infarct':ab,ti OR 'myocardial infarction':ab,ti OR 'myocardium infarct':ab,ti OR 'myocardium infarction':ab,ti OR 'premonitory infarction sign':ab,ti OR 'second heart attack':ab,ti OR 'subendocardial infarction':ab,ti OR 'transmural cardiac infarction':ab,ti OR 'transmural heart infarction':ab,ti OR 'transmural infarction, heart':ab,ti OR 'heart infarction':ab,ti OR ‘Acute Coronary Syndrome’:ab,ti OR ‘ACS’:ab,ti OR ‘Acute Coronary Syndrome*’:ab,ti OR ‘NSTE-ACS’:ab,ti OR ‘Non–ST-Segment Elevation Acute Coronary Syndrome*’:ab,ti OR 'STEMI':ab,ti OR 'NSTEMI':ab,ti OR ‘ST-segment elevation myocardial infarction’:ab,ti OR ‘Non-ST-segment elevation myocardial infarction’:ab,ti OR ‘MINOCA’:ab,ti OR ‘Myocardial infarction with no obstructive coronary atherosclerosis’:ab,ti OR ‘xiongbi’:ab,ti OR ‘xiongbixintong’:ab,ti OR ‘xiongbi Xintong’:ab,ti OR ‘zhenxintong’:ab,ti OR ‘cuxintong’:ab,ti OR ‘Xintong’:ab,ti OR ‘juexintong’:ab,ti OR ‘cardiovascular shock’:ab,ti OR ‘cardiogenic stroke*’:ab,ti

#5 ‘percutaneous coronary intervention'/exp

#6 ‘percutaneous coronary intervention*':ab,ti OR 'percutaneous coronary revascularization*':ab,ti OR 'coronary atherectom*':ab,ti OR 'transluminal coronary angioplast*':ab,ti OR 'transluminal coronary balloon dilation':ab,ti OR 'coronary balloon angioplast*':ab,ti OR 'PCI':ab,ti OR 'Percutaneous Transluminal Coronary Angioplast*':ab,ti OR 'PTCA':ab,ti OR 'Rotational Atherectom*':ab,ti OR 'Directional Coronary Atherectom*':ab,ti OR 'Balloon dilation':ab,ti OR 'Artery Stenting’:ab,ti

#7 #2 OR #3

#8 #4 OR #7

#9 #5 OR #6

#10 #8 AND #9

#11 #1 AND #10

Cochrane

#1 (Ginkgo leaf drop pill):ti,ab,kw OR (Ginkgo Biloba dropping pill):ti,ab,kw OR (Ginkgo biloba):ti,ab,kw OR (Ginkgo Leaf):ti,ab,kw

#2 MeSH descriptor: [Myocardial Infarction] explode all trees

#3 MeSH descriptor: [Acute Coronary Syndrome] explode all trees

#4 (myocardial infarct*):ti,ab,kw OR (heart infarct*):ti,ab,kw OR (cardiac infarct*):ti,ab,kw OR (cardiovascular stroke*):ti,ab,kw OR (cardiogenic shock):ti,ab,kw OR (heart attack*):ti,ab,kw OR (Acute Coronary Syndrome):ti,ab,kw OR (ACS):ti,ab,kw OR (Acute Coronary Syndrome*):ti,ab,kw OR (NSTE-ACS):ti,ab,kw OR (Non–ST-Segment Elevation Acute Coronary Syndrome*):ti,ab,kw OR (STEMI):ti,ab,kw OR (NSTEMI):ti,ab,kw OR (ST-segment elevation myocardial infarction):ti,ab,kw OR (Non-ST-segment elevation myocardial infarction):ti,ab,kw OR (MINOCA):ti,ab,kw OR (Myocardial infarction with no obstructive coronary atherosclerosis):ti,ab,kw OR (xiongbi):ti,ab,kw OR (xiongbixintong):ti,ab,kw OR (xiongbi Xintong):ti,ab,kw OR (zhenxintong):ti,ab,kw OR (cuxintong):ti,ab,kw OR (Xintong):ti,ab,kw OR (juexintong):ti,ab,kw OR (cardiovascular shock):ti,ab,kw OR (cardiogenic stroke*):ti,ab,kw

#5 MeSH descriptor: [Percutaneous Coronary Intervention] explode all trees

#6 (percutaneous coronary intervention):ti,ab,kw OR (percutaneous coronary intervention*):ti,ab,kw OR (percutaneous coronary revascularization*):ti,ab,kw OR (coronary atherectom*):ti,ab,kw OR (transluminal coronary angioplast*):ti,ab,kw OR (transluminal coronary balloon dilation):ti,ab,kw OR (coronary balloon angioplast*):ti,ab,kw OR (PCI):ti,ab,kw OR (Percutaneous Transluminal Coronary Angioplast*):ti,ab,kw OR (PTCA):ti,ab,kw OR (Rotational Atherectom*):ti,ab,kw OR (Directional Coronary Atherectom*):ti,ab,kw OR (Balloon dilation):ti,ab,kw OR (Artery Stenting):ti,ab,kw

#7 #2 OR #3

#8 #4 OR #7

#9 #5 OR #6

#10 #8 AND #9

#11 #1 AND #10

44.	Ginkgo Ketone Ester Dispersible tablets

CNKI

(TKA=‘银杏酮酯分散片’ + ‘银杏酮酯’) and ((TKA=‘心肌梗死’ + ‘心梗’ + ‘心肌梗塞’ + ‘急性冠脉综合征’+ ‘ACS’ + ‘STEMI’ + ‘NSTEMI’ + ‘胸痹’ + ‘胸痹心痛’ + ‘真心痛’ + ‘卒心痛’ + ‘心痛’ + ‘猝心痛’ + ‘厥心痛’) and (TKA=‘PCI’ + ‘PTCA’ + ‘经皮冠状动脉介入治疗术’ + ‘经皮冠状动脉介入’ + ‘经皮冠状动脉’ + ‘冠脉介入’ + ‘经皮冠脉’ + ‘血管成形术’ + ‘支架置入术’ + ‘冠状动脉成形术’ + ‘球囊’ + ‘支架’))

WangFang Data

主题:(“银杏酮酯分散片” OR “银杏酮酯”) and ((主题:心肌梗死 OR 心梗 OR 心肌梗塞 OR 急性冠脉综合征 OR ACS OR STEMI OR NSTEMI OR 胸痹 OR 胸痹心痛 OR 真心痛 OR 卒心痛 OR 心痛 OR 猝心痛 OR 厥心痛) and 主题:(PCI OR PTCA OR 经皮冠状动脉介入治疗术 OR 经皮冠状动脉介入 OR 经皮冠状动脉 OR 冠脉介入 OR 经皮冠脉 OR 血管成形术 OR 支架置入术 OR 冠状动脉成形术 OR 球囊 OR 支架))

VIP

(M=(“银杏酮酯分散片” OR “银杏酮酯”) OR R=(“银杏酮酯分散片” OR “银杏酮酯”)) AND ((M=(心肌梗死 OR 心梗 OR 心肌梗塞 OR 急性冠脉综合征 OR STEMI OR NSTEMI OR 胸痹 OR 胸痹心痛 OR 真心痛 OR 卒心痛 OR 心痛 OR 猝心痛 OR 厥心痛) OR R=(心肌梗死 OR 心梗 OR 心肌梗塞 OR ACS OR STEMI OR NSTEMI OR 胸痹 OR 胸痹心痛 OR 真心痛 OR 卒心痛 OR 心痛 OR 猝心痛 OR 厥心痛)) AND (M=(PCI OR PTCA OR 经皮冠状动脉介入治疗术 OR 经皮冠状动脉介入 OR 经皮冠状动脉 OR 冠脉介入 OR 经皮冠脉 OR 血管成形术 OR 支架置入术 OR 冠状动脉成形术 OR 球囊 OR 支架) OR R=(PCI OR PTCA OR 经皮冠状动脉介入治疗术 OR 经皮冠状动脉介入 OR 经皮冠状动脉 OR 冠脉介入 OR 经皮冠脉 OR 血管成形术 OR 支架置入术 OR 冠状动脉成形术 OR 球囊 OR 支架)))

Web of Science

#1 TS=(Ginkgo Ketone Ester Dispersible tablet* OR Ginkgo Ketone Ester Dispersible)

#2 TS=(myocardial infarct* OR heart infarct* OR cardiac infarct* OR cardiovascular stroke* OR cardiogenic shock OR heart attack* OR ACS OR STEMI OR NSTEMI OR ST-segment elevation myocardial infarction OR Non-ST-segment elevation myocardial infarction OR MINOCA OR Myocardial infarction with no obstructive coronary atherosclerosis OR xiongbi OR xiongbixintong OR xiongbi Xintong OR zhenxintong OR cuxintong OR Xintong OR juexintong OR cardiovascular shock OR cardiogenic stroke*)

#3 TS=(percutaneous coronary intervention OR percutaneous coronary intervention* OR percutaneous coronary revascularization* OR coronary atherectom* OR transluminal coronary angioplast* OR transluminal coronary balloon dilation OR coronary balloon angioplast* OR PCI OR Percutaneous Transluminal Coronary Angioplast* OR PTCA OR Rotational Atherectom* OR Directional Coronary Atherectom* OR Balloon dilation OR Artery Stenting)

#4 #2 AND #3

#5 #1 AND #4

PubMed

#1 “Ginkgo Ketone Ester Dispersible tablet*”[Title/Abstract] OR “Ginkgo Ketone Ester Dispersible”[Title/Abstract]

#2 "myocardial infarction"[MeSH Terms] OR “Acute Coronary Syndrome”[MeSH Terms] OR "myocardial infarct*"[Title/Abstract] OR “Acute Coronary Syndrome”[Title/Abstract] OR “ACS”[Title/Abstract] OR “Acute Coronary Syndrome*”[Title/Abstract]OR “NSTE-ACS”[Title/Abstract] OR “Non–ST-Segment Elevation Acute Coronary Syndrome*”[Title/Abstract] OR "heart infarct*"[Title/Abstract] OR "cardiac infarct*"[Title/Abstract] OR "cardiovascular stroke*"[Title/Abstract] OR "cardiogenic shock"[Title/Abstract] OR “cardiovascular shock” [Title/Abstract] OR “cardiogenic stroke*” [Title/Abstract] OR "heart attack*"[Title/Abstract] OR "STEMI"[Title/Abstract] OR "NSTEMI"[Title/Abstract] OR “ST-segment elevation myocardial infarction”[Title/Abstract] OR “Non-ST-segment elevation myocardial infarction”[Title/Abstract] OR “MINOCA”[Title/Abstract] OR “Myocardial infarction with no obstructive coronary atherosclerosis” [Title/Abstract] OR “xiongbi” [Title/Abstract] OR “xiongbixintong” [Title/Abstract] OR “xiongbi xintong” [Title/Abstract] OR “zhenxintong” [Title/Abstract] OR “cuxintong” [Title/Abstract] OR “Xintong” [Title/Abstract] OR “juexintong” [Title/Abstract]

#3 “percutaneous coronary intervention” [MeSH Terms] OR “percutaneous coronary intervention*”[Title/Abstract] OR “percutaneous coronary revascularization*” [Title/Abstract] OR “coronary atherectom*”[Title/Abstract] OR “transluminal coronary angioplast*”[Title/Abstract] OR “transluminal coronary balloon dilation”[Title/Abstract] OR “coronary balloon angioplast*”[Title/Abstract] OR “PCI”[Title/Abstract] OR “Percutaneous Transluminal Coronary Angioplast*”[Title/Abstract] OR “PTCA”[Title/Abstract] OR “Rotational Atherectom*”[Title/Abstract] OR

“Directional Coronary Atherectom*”[Title/Abstract] OR “Balloon dilation”[Title/Abstract] OR “Artery Stenting”[Title/Abstract]

#4 #2 AND #3

#1 AND #4

Embase

#1 'Ginkgo Ketone Ester Dispersible tablet*':ab,ti OR ‘Ginkgo Ketone Ester Dispersible’:ab,ti

#2 'heart infarction'/exp

#3 'Acute Coronary Syndrome'/exp

#4 'cardiac infarct':ab,ti OR 'cardiac infarction':ab,ti OR 'cardial infarct':ab,ti OR 'heart attack':ab,ti OR 'heart infarct':ab,ti OR 'heart micro infarction':ab,ti OR 'heart muscle infarction':ab,ti OR 'infarction, heart':ab,ti OR 'myocardial infarct':ab,ti OR 'myocardial infarction':ab,ti OR 'myocardium infarct':ab,ti OR 'myocardium infarction':ab,ti OR 'premonitory infarction sign':ab,ti OR 'second heart attack':ab,ti OR 'subendocardial infarction':ab,ti OR 'transmural cardiac infarction':ab,ti OR 'transmural heart infarction':ab,ti OR 'transmural infarction, heart':ab,ti OR 'heart infarction':ab,ti OR ‘Acute Coronary Syndrome’:ab,ti OR ‘ACS’:ab,ti OR ‘Acute Coronary Syndrome*’:ab,ti OR ‘NSTE-ACS’:ab,ti OR ‘Non–ST-Segment Elevation Acute Coronary Syndrome*’:ab,ti OR 'STEMI':ab,ti OR 'NSTEMI':ab,ti OR ‘ST-segment elevation myocardial infarction’:ab,ti OR ‘Non-ST-segment elevation myocardial infarction’:ab,ti OR ‘MINOCA’:ab,ti OR ‘Myocardial infarction with no obstructive coronary atherosclerosis’:ab,ti OR ‘xiongbi’:ab,ti OR ‘xiongbixintong’:ab,ti OR ‘xiongbi Xintong’:ab,ti OR ‘zhenxintong’:ab,ti OR ‘cuxintong’:ab,ti OR ‘Xintong’:ab,ti OR ‘juexintong’:ab,ti OR ‘cardiovascular shock’:ab,ti OR ‘cardiogenic stroke*’:ab,ti

#5 ‘percutaneous coronary intervention'/exp

#6 ‘percutaneous coronary intervention*':ab,ti OR 'percutaneous coronary revascularization*':ab,ti OR 'coronary atherectom*':ab,ti OR 'transluminal coronary angioplast*':ab,ti OR 'transluminal coronary balloon dilation':ab,ti OR 'coronary balloon angioplast*':ab,ti OR 'PCI':ab,ti OR 'Percutaneous Transluminal Coronary Angioplast*':ab,ti OR 'PTCA':ab,ti OR 'Rotational Atherectom*':ab,ti OR 'Directional Coronary Atherectom*':ab,ti OR 'Balloon dilation':ab,ti OR 'Artery Stenting’:ab,ti

#7 #2 OR #3

#8 #4 OR #7

#9 #5 OR #6

#10 #8 AND #9

#11 #1 AND #10

Cochrane

#1 (Ginkgo Ketone Ester Dispersible tablet*):ti,ab,kw OR (Ginkgo Ketone Ester Dispersible):ti,ab,kw

#2 MeSH descriptor: [Myocardial Infarction] explode all trees

#3 MeSH descriptor: [Acute Coronary Syndrome] explode all trees

#4 (myocardial infarct*):ti,ab,kw OR (heart infarct*):ti,ab,kw OR (cardiac infarct*):ti,ab,kw OR (cardiovascular stroke*):ti,ab,kw OR (cardiogenic shock):ti,ab,kw OR (heart attack*):ti,ab,kw OR (Acute Coronary Syndrome):ti,ab,kw OR (ACS):ti,ab,kw OR (Acute Coronary Syndrome*):ti,ab,kw OR (NSTE-ACS):ti,ab,kw OR (Non–ST-Segment Elevation Acute Coronary Syndrome*):ti,ab,kw OR (STEMI):ti,ab,kw OR (NSTEMI):ti,ab,kw OR (ST-segment elevation myocardial infarction):ti,ab,kw OR (Non-ST-segment elevation myocardial infarction):ti,ab,kw OR (MINOCA):ti,ab,kw OR (Myocardial infarction with no obstructive coronary atherosclerosis):ti,ab,kw OR (xiongbi):ti,ab,kw OR (xiongbixintong):ti,ab,kw OR (xiongbi Xintong):ti,ab,kw OR (zhenxintong):ti,ab,kw OR (cuxintong):ti,ab,kw OR (Xintong):ti,ab,kw OR (juexintong):ti,ab,kw OR (cardiovascular shock):ti,ab,kw OR (cardiogenic stroke*):ti,ab,kw

#5 MeSH descriptor: [Percutaneous Coronary Intervention] explode all trees

#6 (percutaneous coronary intervention):ti,ab,kw OR (percutaneous coronary intervention*):ti,ab,kw OR (percutaneous coronary revascularization*):ti,ab,kw OR (coronary atherectom*):ti,ab,kw OR (transluminal coronary angioplast*):ti,ab,kw OR (transluminal coronary balloon dilation):ti,ab,kw OR (coronary balloon angioplast*):ti,ab,kw OR (PCI):ti,ab,kw OR (Percutaneous Transluminal Coronary Angioplast*):ti,ab,kw OR (PTCA):ti,ab,kw OR (Rotational Atherectom*):ti,ab,kw OR (Directional Coronary Atherectom*):ti,ab,kw OR (Balloon dilation):ti,ab,kw OR (Artery Stenting):ti,ab,kw

#7 #2 OR #3

#8 #4 OR #7

#9 #5 OR #6

#10 #8 AND #9

#11 #1 AND #10

45.Ginkgo Ketone Ester dropping pills

CNKI

(TKA=‘银杏酮酯滴丸’ + ‘银杏酮酯’) and ((TKA=‘心肌梗死’ + ‘心梗’ + ‘心肌梗塞’ + ‘急性冠脉综合征’+ ‘ACS’ + ‘STEMI’ + ‘NSTEMI’ + ‘胸痹’ + ‘胸痹心痛’ + ‘真心痛’ + ‘卒心痛’ + ‘心痛’ + ‘猝心痛’ + ‘厥心痛’) and (TKA=‘PCI’ + ‘PTCA’ + ‘经皮冠状动脉介入治疗术’ + ‘经皮冠状动脉介入’ + ‘经皮冠状动脉’ + ‘冠脉介入’ + ‘经皮冠脉’ + ‘血管成形术’ + ‘支架置入术’ + ‘冠状动脉成形术’ + ‘球囊’ + ‘支架’))

WangFang Data

主题:(“银杏酮酯滴丸” OR “银杏酮酯”) and ((主题:心肌梗死 OR 心梗 OR 心肌梗塞 OR 急性冠脉综合征 OR ACS OR STEMI OR NSTEMI OR 胸痹 OR 胸痹心痛 OR 真心痛 OR 卒心痛 OR 心痛 OR 猝心痛 OR 厥心痛) and 主题:(PCI OR PTCA OR 经皮冠状动脉介入治疗术 OR 经皮冠状动脉介入 OR 经皮冠状动脉 OR 冠脉介入 OR 经皮冠脉 OR 血管成形术 OR 支架置入术 OR 冠状动脉成形术 OR 球囊 OR 支架))

VIP

(M=(“银杏酮酯滴丸” OR “银杏酮酯”) OR R=(“银杏酮酯滴丸” OR “银杏酮酯”)) AND ((M=(心肌梗死 OR 心梗 OR 心肌梗塞 OR 急性冠脉综合征 OR STEMI OR NSTEMI OR 胸痹 OR 胸痹心痛 OR 真心痛 OR 卒心痛 OR 心痛 OR 猝心痛 OR 厥心痛) OR R=(心肌梗死 OR 心梗 OR 心肌梗塞 OR ACS OR STEMI OR NSTEMI OR 胸痹 OR 胸痹心痛 OR 真心痛 OR 卒心痛 OR 心痛 OR 猝心痛 OR 厥心痛)) AND (M=(PCI OR PTCA OR 经皮冠状动脉介入治疗术 OR 经皮冠状动脉介入 OR 经皮冠状动脉 OR 冠脉介入 OR 经皮冠脉 OR 血管成形术 OR 支架置入术 OR 冠状动脉成形术 OR 球囊 OR 支架) OR R=(PCI OR PTCA OR 经皮冠状动脉介入治疗术 OR 经皮冠状动脉介入 OR 经皮冠状动脉 OR 冠脉介入 OR 经皮冠脉 OR 血管成形术 OR 支架置入术 OR 冠状动脉成形术 OR 球囊 OR 支架)))

Web of Science

#1 TS=(Ginkgo Ketone Ester dropping pill OR Ginkgo Ketone dropping pill OR Ginkgo Ketone Ester OR Ginkgo Ketone)

#2 TS=(myocardial infarct* OR heart infarct* OR cardiac infarct* OR cardiovascular stroke* OR cardiogenic shock OR heart attack* OR ACS OR STEMI OR NSTEMI OR ST-segment elevation myocardial infarction OR Non-ST-segment elevation myocardial infarction OR MINOCA OR Myocardial infarction with no obstructive coronary atherosclerosis OR xiongbi OR xiongbixintong OR xiongbi Xintong OR zhenxintong OR cuxintong OR Xintong OR juexintong OR cardiovascular shock OR cardiogenic stroke*)

#3 TS=(percutaneous coronary intervention OR percutaneous coronary intervention* OR percutaneous coronary revascularization* OR coronary atherectom* OR transluminal coronary angioplast* OR transluminal coronary balloon dilation OR coronary balloon angioplast* OR PCI OR Percutaneous Transluminal Coronary Angioplast* OR PTCA OR Rotational Atherectom* OR Directional Coronary Atherectom* OR Balloon dilation OR Artery Stenting)

#4 #2 AND #3

#5 #1 AND #4

PubMed

#1 “Ginkgo Ketone Ester dropping pill”[Title/Abstract] OR “Ginkgo Ketone dropping pill”[Title/Abstract] OR “Ginkgo Ketone Ester”[Title/Abstract] OR “Ginkgo Ketone”[Title/Abstract]

#2 "myocardial infarction"[MeSH Terms] OR “Acute Coronary Syndrome”[MeSH Terms] OR "myocardial infarct*"[Title/Abstract] OR “Acute Coronary Syndrome”[Title/Abstract] OR “ACS”[Title/Abstract] OR “Acute Coronary Syndrome*”[Title/Abstract]OR “NSTE-ACS”[Title/Abstract] OR “Non–ST-Segment Elevation Acute Coronary Syndrome*”[Title/Abstract] OR "heart infarct*"[Title/Abstract] OR "cardiac infarct*"[Title/Abstract] OR "cardiovascular stroke*"[Title/Abstract] OR "cardiogenic shock"[Title/Abstract] OR “cardiovascular shock” [Title/Abstract] OR “cardiogenic stroke*” [Title/Abstract] OR "heart attack*"[Title/Abstract] OR "STEMI"[Title/Abstract] OR "NSTEMI"[Title/Abstract] OR “ST-segment elevation myocardial infarction”[Title/Abstract] OR “Non-ST-segment elevation myocardial infarction”[Title/Abstract] OR “MINOCA”[Title/Abstract] OR “Myocardial infarction with no obstructive coronary atherosclerosis” [Title/Abstract] OR “xiongbi” [Title/Abstract] OR “xiongbixintong” [Title/Abstract] OR “xiongbi xintong” [Title/Abstract] OR “zhenxintong” [Title/Abstract] OR “cuxintong” [Title/Abstract] OR “Xintong” [Title/Abstract] OR “juexintong” [Title/Abstract]

#3 “percutaneous coronary intervention” [MeSH Terms] OR “percutaneous coronary intervention*”[Title/Abstract] OR “percutaneous coronary revascularization*” [Title/Abstract] OR “coronary atherectom*”[Title/Abstract] OR “transluminal coronary angioplast*”[Title/Abstract] OR “transluminal coronary balloon dilation”[Title/Abstract] OR “coronary balloon angioplast*”[Title/Abstract] OR “PCI”[Title/Abstract] OR “Percutaneous Transluminal Coronary Angioplast*”[Title/Abstract] OR “PTCA”[Title/Abstract] OR “Rotational Atherectom*”[Title/Abstract] OR

“Directional Coronary Atherectom*”[Title/Abstract] OR “Balloon dilation”[Title/Abstract] OR “Artery Stenting”[Title/Abstract]

#4 #2 AND #3

#1 AND #4

Embase

#1 'Ginkgo Ketone Ester dropping pill':ab,ti OR ‘Ginkgo Ketone dropping pill’:ab,ti OR ‘Ginkgo Ketone Ester’:ab,ti OR ‘Ginkgo Ketone':ab,ti

#2 'heart infarction'/exp

#3 'Acute Coronary Syndrome'/exp

#4 'cardiac infarct':ab,ti OR 'cardiac infarction':ab,ti OR 'cardial infarct':ab,ti OR 'heart attack':ab,ti OR 'heart infarct':ab,ti OR 'heart micro infarction':ab,ti OR 'heart muscle infarction':ab,ti OR 'infarction, heart':ab,ti OR 'myocardial infarct':ab,ti OR 'myocardial infarction':ab,ti OR 'myocardium infarct':ab,ti OR 'myocardium infarction':ab,ti OR 'premonitory infarction sign':ab,ti OR 'second heart attack':ab,ti OR 'subendocardial infarction':ab,ti OR 'transmural cardiac infarction':ab,ti OR 'transmural heart infarction':ab,ti OR 'transmural infarction, heart':ab,ti OR 'heart infarction':ab,ti OR ‘Acute Coronary Syndrome’:ab,ti OR ‘ACS’:ab,ti OR ‘Acute Coronary Syndrome*’:ab,ti OR ‘NSTE-ACS’:ab,ti OR ‘Non–ST-Segment Elevation Acute Coronary Syndrome*’:ab,ti OR 'STEMI':ab,ti OR 'NSTEMI':ab,ti OR ‘ST-segment elevation myocardial infarction’:ab,ti OR ‘Non-ST-segment elevation myocardial infarction’:ab,ti OR ‘MINOCA’:ab,ti OR ‘Myocardial infarction with no obstructive coronary atherosclerosis’:ab,ti OR ‘xiongbi’:ab,ti OR ‘xiongbixintong’:ab,ti OR ‘xiongbi Xintong’:ab,ti OR ‘zhenxintong’:ab,ti OR ‘cuxintong’:ab,ti OR ‘Xintong’:ab,ti OR ‘juexintong’:ab,ti OR ‘cardiovascular shock’:ab,ti OR ‘cardiogenic stroke*’:ab,ti

#5 ‘percutaneous coronary intervention'/exp

#6 ‘percutaneous coronary intervention*':ab,ti OR 'percutaneous coronary revascularization*':ab,ti OR 'coronary atherectom*':ab,ti OR 'transluminal coronary angioplast*':ab,ti OR 'transluminal coronary balloon dilation':ab,ti OR 'coronary balloon angioplast*':ab,ti OR 'PCI':ab,ti OR 'Percutaneous Transluminal Coronary Angioplast*':ab,ti OR 'PTCA':ab,ti OR 'Rotational Atherectom*':ab,ti OR 'Directional Coronary Atherectom*':ab,ti OR 'Balloon dilation':ab,ti OR 'Artery Stenting’:ab,ti

#7 #2 OR #3

#8 #4 OR #7

#9 #5 OR #6

#10 #8 AND #9

#11 #1 AND #10

Cochrane

#1 (Ginkgo Ketone Ester dropping pill):ti,ab,kw OR (Ginkgo Ketone dropping pill):ti,ab,kw OR (Ginkgo Ketone Ester):ti,ab,kw OR (Ginkgo Ketone):ti,ab,kw

#2 MeSH descriptor: [Myocardial Infarction] explode all trees

#3 MeSH descriptor: [Acute Coronary Syndrome] explode all trees

#4 (myocardial infarct*):ti,ab,kw OR (heart infarct*):ti,ab,kw OR (cardiac infarct*):ti,ab,kw OR (cardiovascular stroke*):ti,ab,kw OR (cardiogenic shock):ti,ab,kw OR (heart attack*):ti,ab,kw OR (Acute Coronary Syndrome):ti,ab,kw OR (ACS):ti,ab,kw OR (Acute Coronary Syndrome*):ti,ab,kw OR (NSTE-ACS):ti,ab,kw OR (Non–ST-Segment Elevation Acute Coronary Syndrome*):ti,ab,kw OR (STEMI):ti,ab,kw OR (NSTEMI):ti,ab,kw OR (ST-segment elevation myocardial infarction):ti,ab,kw OR (Non-ST-segment elevation myocardial infarction):ti,ab,kw OR (MINOCA):ti,ab,kw OR (Myocardial infarction with no obstructive coronary atherosclerosis):ti,ab,kw OR (xiongbi):ti,ab,kw OR (xiongbixintong):ti,ab,kw OR (xiongbi Xintong):ti,ab,kw OR (zhenxintong):ti,ab,kw OR (cuxintong):ti,ab,kw OR (Xintong):ti,ab,kw OR (juexintong):ti,ab,kw OR (cardiovascular shock):ti,ab,kw OR (cardiogenic stroke*):ti,ab,kw

#5 MeSH descriptor: [Percutaneous Coronary Intervention] explode all trees

#6 (percutaneous coronary intervention):ti,ab,kw OR (percutaneous coronary intervention*):ti,ab,kw OR (percutaneous coronary revascularization*):ti,ab,kw OR (coronary atherectom*):ti,ab,kw OR (transluminal coronary angioplast*):ti,ab,kw OR (transluminal coronary balloon dilation):ti,ab,kw OR (coronary balloon angioplast*):ti,ab,kw OR (PCI):ti,ab,kw OR (Percutaneous Transluminal Coronary Angioplast*):ti,ab,kw OR (PTCA):ti,ab,kw OR (Rotational Atherectom*):ti,ab,kw OR (Directional Coronary Atherectom*):ti,ab,kw OR (Balloon dilation):ti,ab,kw OR (Artery Stenting):ti,ab,kw

#7 #2 OR #3

#8 #4 OR #7

#9 #5 OR #6

#10 #8 AND #9

#11 #1 AND #10

46.Shexiang Baoxin pills

CNKI

(TKA=‘麝香保心丸’ + ‘麝香保心’) and ((TKA=‘心肌梗死’ + ‘心梗’ + ‘心肌梗塞’ + ‘急性冠脉综合征’+ ‘ACS’ + ‘STEMI’ + ‘NSTEMI’ + ‘胸痹’ + ‘胸痹心痛’ + ‘真心痛’ + ‘卒心痛’ + ‘心痛’ + ‘猝心痛’ + ‘厥心痛’) and (TKA=‘PCI’ + ‘PTCA’ + ‘经皮冠状动脉介入治疗术’ + ‘经皮冠状动脉介入’ + ‘经皮冠状动脉’ + ‘冠脉介入’ + ‘经皮冠脉’ + ‘血管成形术’ + ‘支架置入术’ + ‘冠状动脉成形术’ + ‘球囊’ + ‘支架’))

WangFang Data

主题:(“麝香保心丸” OR “麝香保心”) and ((主题:心肌梗死 OR 心梗 OR 心肌梗塞 OR 急性冠脉综合征 OR ACS OR STEMI OR NSTEMI OR 胸痹 OR 胸痹心痛 OR 真心痛 OR 卒心痛 OR 心痛 OR 猝心痛 OR 厥心痛) and 主题:(PCI OR PTCA OR 经皮冠状动脉介入治疗术 OR 经皮冠状动脉介入 OR 经皮冠状动脉 OR 冠脉介入 OR 经皮冠脉 OR 血管成形术 OR 支架置入术 OR 冠状动脉成形术 OR 球囊 OR 支架))

VIP

(M=(“麝香保心丸” OR “麝香保心”) OR R=(“麝香保心丸” OR “麝香保心”)) AND ((M=(心肌梗死 OR 心梗 OR 心肌梗塞 OR 急性冠脉综合征 OR STEMI OR NSTEMI OR 胸痹 OR 胸痹心痛 OR 真心痛 OR 卒心痛 OR 心痛 OR 猝心痛 OR 厥心痛) OR R=(心肌梗死 OR 心梗 OR 心肌梗塞 OR ACS OR STEMI OR NSTEMI OR 胸痹 OR 胸痹心痛 OR 真心痛 OR 卒心痛 OR 心痛 OR 猝心痛 OR 厥心痛)) AND (M=(PCI OR PTCA OR 经皮冠状动脉介入治疗术 OR 经皮冠状动脉介入 OR 经皮冠状动脉 OR 冠脉介入 OR 经皮冠脉 OR 血管成形术 OR 支架置入术 OR 冠状动脉成形术 OR 球囊 OR 支架) OR R=(PCI OR PTCA OR 经皮冠状动脉介入治疗术 OR 经皮冠状动脉介入 OR 经皮冠状动脉 OR 冠脉介入 OR 经皮冠脉 OR 血管成形术 OR 支架置入术 OR 冠状动脉成形术 OR 球囊 OR 支架)))

Web of Science

#1 TS=(Shexiang Baoxin pill OR Shexiang Baoxin Wan OR MUSKARDIA OR shexiangbaoxin OR shexiang baoxin)

#2 TS=(myocardial infarct* OR heart infarct* OR cardiac infarct* OR cardiovascular stroke* OR cardiogenic shock OR heart attack* OR ACS OR STEMI OR NSTEMI OR ST-segment elevation myocardial infarction OR Non-ST-segment elevation myocardial infarction OR MINOCA OR Myocardial infarction with no obstructive coronary atherosclerosis OR xiongbi OR xiongbixintong OR xiongbi Xintong OR zhenxintong OR cuxintong OR Xintong OR juexintong OR cardiovascular shock OR cardiogenic stroke*)

#3 TS=(percutaneous coronary intervention OR percutaneous coronary intervention* OR percutaneous coronary revascularization* OR coronary atherectom* OR transluminal coronary angioplast* OR transluminal coronary balloon dilation OR coronary balloon angioplast* OR PCI OR Percutaneous Transluminal Coronary Angioplast* OR PTCA OR Rotational Atherectom* OR Directional Coronary Atherectom* OR Balloon dilation OR Artery Stenting)

#4 #2 AND #3

#5 #1 AND #4

PubMed

#1 “Shexiang Baoxin pill”[Title/Abstract] OR “Shexiang Baoxin Wan”[Title/Abstract] OR “MUSKARDIA”[Title/Abstract] OR “shexiangbaoxin”[Title/Abstract] OR “shexiang baoxin”[Title/Abstract]

#2 "myocardial infarction"[MeSH Terms] OR “Acute Coronary Syndrome”[MeSH Terms] OR "myocardial infarct*"[Title/Abstract] OR “Acute Coronary Syndrome”[Title/Abstract] OR “ACS”[Title/Abstract] OR “Acute Coronary Syndrome*”[Title/Abstract]OR “NSTE-ACS”[Title/Abstract] OR “Non–ST-Segment Elevation Acute Coronary Syndrome*”[Title/Abstract] OR "heart infarct*"[Title/Abstract] OR "cardiac infarct*"[Title/Abstract] OR "cardiovascular stroke*"[Title/Abstract] OR "cardiogenic shock"[Title/Abstract] OR “cardiovascular shock” [Title/Abstract] OR “cardiogenic stroke*” [Title/Abstract] OR "heart attack*"[Title/Abstract] OR "STEMI"[Title/Abstract] OR "NSTEMI"[Title/Abstract] OR “ST-segment elevation myocardial infarction”[Title/Abstract] OR “Non-ST-segment elevation myocardial infarction”[Title/Abstract] OR “MINOCA”[Title/Abstract] OR “Myocardial infarction with no obstructive coronary atherosclerosis” [Title/Abstract] OR “xiongbi” [Title/Abstract] OR “xiongbixintong” [Title/Abstract] OR “xiongbi xintong” [Title/Abstract] OR “zhenxintong” [Title/Abstract] OR “cuxintong” [Title/Abstract] OR “Xintong” [Title/Abstract] OR “juexintong” [Title/Abstract]

#3 “percutaneous coronary intervention” [MeSH Terms] OR “percutaneous coronary intervention*”[Title/Abstract] OR “percutaneous coronary revascularization*” [Title/Abstract] OR “coronary atherectom*”[Title/Abstract] OR “transluminal coronary angioplast*”[Title/Abstract] OR “transluminal coronary balloon dilation”[Title/Abstract] OR “coronary balloon angioplast*”[Title/Abstract] OR “PCI”[Title/Abstract] OR “Percutaneous Transluminal Coronary Angioplast*”[Title/Abstract] OR “PTCA”[Title/Abstract] OR “Rotational Atherectom*”[Title/Abstract] OR

“Directional Coronary Atherectom*”[Title/Abstract] OR “Balloon dilation”[Title/Abstract] OR “Artery Stenting”[Title/Abstract]

#4 #2 AND #3

#1 AND #4

Embase

#1 'Shexiang Baoxin pill':ab,ti OR ‘Shexiang Baoxin Wan’:ab,ti OR ‘MUSKARDIA’:ab,ti OR ‘shexiangbaoxin':ab,ti OR ‘shexiang baoxin’:ab,ti

#2 'heart infarction'/exp

#3 'Acute Coronary Syndrome'/exp

#4 'cardiac infarct':ab,ti OR 'cardiac infarction':ab,ti OR 'cardial infarct':ab,ti OR 'heart attack':ab,ti OR 'heart infarct':ab,ti OR 'heart micro infarction':ab,ti OR 'heart muscle infarction':ab,ti OR 'infarction, heart':ab,ti OR 'myocardial infarct':ab,ti OR 'myocardial infarction':ab,ti OR 'myocardium infarct':ab,ti OR 'myocardium infarction':ab,ti OR 'premonitory infarction sign':ab,ti OR 'second heart attack':ab,ti OR 'subendocardial infarction':ab,ti OR 'transmural cardiac infarction':ab,ti OR 'transmural heart infarction':ab,ti OR 'transmural infarction, heart':ab,ti OR 'heart infarction':ab,ti OR ‘Acute Coronary Syndrome’:ab,ti OR ‘ACS’:ab,ti OR ‘Acute Coronary Syndrome*’:ab,ti OR ‘NSTE-ACS’:ab,ti OR ‘Non–ST-Segment Elevation Acute Coronary Syndrome*’:ab,ti OR 'STEMI':ab,ti OR 'NSTEMI':ab,ti OR ‘ST-segment elevation myocardial infarction’:ab,ti OR ‘Non-ST-segment elevation myocardial infarction’:ab,ti OR ‘MINOCA’:ab,ti OR ‘Myocardial infarction with no obstructive coronary atherosclerosis’:ab,ti OR ‘xiongbi’:ab,ti OR ‘xiongbixintong’:ab,ti OR ‘xiongbi Xintong’:ab,ti OR ‘zhenxintong’:ab,ti OR ‘cuxintong’:ab,ti OR ‘Xintong’:ab,ti OR ‘juexintong’:ab,ti OR ‘cardiovascular shock’:ab,ti OR ‘cardiogenic stroke*’:ab,ti

#5 ‘percutaneous coronary intervention'/exp

#6 ‘percutaneous coronary intervention*':ab,ti OR 'percutaneous coronary revascularization*':ab,ti OR 'coronary atherectom*':ab,ti OR 'transluminal coronary angioplast*':ab,ti OR 'transluminal coronary balloon dilation':ab,ti OR 'coronary balloon angioplast*':ab,ti OR 'PCI':ab,ti OR 'Percutaneous Transluminal Coronary Angioplast*':ab,ti OR 'PTCA':ab,ti OR 'Rotational Atherectom*':ab,ti OR 'Directional Coronary Atherectom*':ab,ti OR 'Balloon dilation':ab,ti OR 'Artery Stenting’:ab,ti

#7 #2 OR #3

#8 #4 OR #7

#9 #5 OR #6

#10 #8 AND #9

#11 #1 AND #10

Cochrane

#1 (Shexiang Baoxin pill):ti,ab,kw OR (Shexiang Baoxin Wan):ti,ab,kw OR (MUSKARDIA):ti,ab,kw OR (shexiangbaoxin):ti,ab,kw OR (shexiang baoxin):ti,ab,kw

#2 MeSH descriptor: [Myocardial Infarction] explode all trees

#3 MeSH descriptor: [Acute Coronary Syndrome] explode all trees

#4 (myocardial infarct*):ti,ab,kw OR (heart infarct*):ti,ab,kw OR (cardiac infarct*):ti,ab,kw OR (cardiovascular stroke*):ti,ab,kw OR (cardiogenic shock):ti,ab,kw OR (heart attack*):ti,ab,kw OR (Acute Coronary Syndrome):ti,ab,kw OR (ACS):ti,ab,kw OR (Acute Coronary Syndrome*):ti,ab,kw OR (NSTE-ACS):ti,ab,kw OR (Non–ST-Segment Elevation Acute Coronary Syndrome*):ti,ab,kw OR (STEMI):ti,ab,kw OR (NSTEMI):ti,ab,kw OR (ST-segment elevation myocardial infarction):ti,ab,kw OR (Non-ST-segment elevation myocardial infarction):ti,ab,kw OR (MINOCA):ti,ab,kw OR (Myocardial infarction with no obstructive coronary atherosclerosis):ti,ab,kw OR (xiongbi):ti,ab,kw OR (xiongbixintong):ti,ab,kw OR (xiongbi Xintong):ti,ab,kw OR (zhenxintong):ti,ab,kw OR (cuxintong):ti,ab,kw OR (Xintong):ti,ab,kw OR (juexintong):ti,ab,kw OR (cardiovascular shock):ti,ab,kw OR (cardiogenic stroke*):ti,ab,kw

#5 MeSH descriptor: [Percutaneous Coronary Intervention] explode all trees

#6 (percutaneous coronary intervention):ti,ab,kw OR (percutaneous coronary intervention*):ti,ab,kw OR (percutaneous coronary revascularization*):ti,ab,kw OR (coronary atherectom*):ti,ab,kw OR (transluminal coronary angioplast*):ti,ab,kw OR (transluminal coronary balloon dilation):ti,ab,kw OR (coronary balloon angioplast*):ti,ab,kw OR (PCI):ti,ab,kw OR (Percutaneous Transluminal Coronary Angioplast*):ti,ab,kw OR (PTCA):ti,ab,kw OR (Rotational Atherectom*):ti,ab,kw OR (Directional Coronary Atherectom*):ti,ab,kw OR (Balloon dilation):ti,ab,kw OR (Artery Stenting):ti,ab,kw

#7 #2 OR #3

#8 #4 OR #7

#9 #5 OR #6

#10 #8 AND #9

#11 #1 AND #10

47.Shexiang Tongxin dripping pills

CNKI

(TKA=‘麝香通心滴丸’ + ‘麝香通心’) and ((TKA=‘心肌梗死’ + ‘心梗’ + ‘心肌梗塞’ + ‘急性冠脉综合征’+ ‘ACS’ + ‘STEMI’ + ‘NSTEMI’ + ‘胸痹’ + ‘胸痹心痛’ + ‘真心痛’ + ‘卒心痛’ + ‘心痛’ + ‘猝心痛’ + ‘厥心痛’) and (TKA=‘PCI’ + ‘PTCA’ + ‘经皮冠状动脉介入治疗术’ + ‘经皮冠状动脉介入’ + ‘经皮冠状动脉’ + ‘冠脉介入’ + ‘经皮冠脉’ + ‘血管成形术’ + ‘支架置入术’ + ‘冠状动脉成形术’ + ‘球囊’ + ‘支架’))

WangFang Data

主题:(“麝香通心滴丸” OR “麝香通心”) and ((主题:心肌梗死 OR 心梗 OR 心肌梗塞 OR 急性冠脉综合征 OR ACS OR STEMI OR NSTEMI OR 胸痹 OR 胸痹心痛 OR 真心痛 OR 卒心痛 OR 心痛 OR 猝心痛 OR 厥心痛) and 主题:(PCI OR PTCA OR 经皮冠状动脉介入治疗术 OR 经皮冠状动脉介入 OR 经皮冠状动脉 OR 冠脉介入 OR 经皮冠脉 OR 血管成形术 OR 支架置入术 OR 冠状动脉成形术 OR 球囊 OR 支架))

VIP

(M=(“麝香通心滴丸” OR “麝香通心”) OR R=(“麝香通心滴丸” OR “麝香通心”)) AND ((M=(心肌梗死 OR 心梗 OR 心肌梗塞 OR 急性冠脉综合征 OR STEMI OR NSTEMI OR 胸痹 OR 胸痹心痛 OR 真心痛 OR 卒心痛 OR 心痛 OR 猝心痛 OR 厥心痛) OR R=(心肌梗死 OR 心梗 OR 心肌梗塞 OR ACS OR STEMI OR NSTEMI OR 胸痹 OR 胸痹心痛 OR 真心痛 OR 卒心痛 OR 心痛 OR 猝心痛 OR 厥心痛)) AND (M=(PCI OR PTCA OR 经皮冠状动脉介入治疗术 OR 经皮冠状动脉介入 OR 经皮冠状动脉 OR 冠脉介入 OR 经皮冠脉 OR 血管成形术 OR 支架置入术 OR 冠状动脉成形术 OR 球囊 OR 支架) OR R=(PCI OR PTCA OR 经皮冠状动脉介入治疗术 OR 经皮冠状动脉介入 OR 经皮冠状动脉 OR 冠脉介入 OR 经皮冠脉 OR 血管成形术 OR 支架置入术 OR 冠状动脉成形术 OR 球囊 OR 支架)))

Web of Science

#1 TS=(Shexiang Tongxin dripping pill OR shexiang tongxin OR shexiangtongxin)

#2 TS=(myocardial infarct* OR heart infarct* OR cardiac infarct* OR cardiovascular stroke* OR cardiogenic shock OR heart attack* OR ACS OR STEMI OR NSTEMI OR ST-segment elevation myocardial infarction OR Non-ST-segment elevation myocardial infarction OR MINOCA OR Myocardial infarction with no obstructive coronary atherosclerosis OR xiongbi OR xiongbixintong OR xiongbi Xintong OR zhenxintong OR cuxintong OR Xintong OR juexintong OR cardiovascular shock OR cardiogenic stroke*)

#3 TS=(percutaneous coronary intervention OR percutaneous coronary intervention* OR percutaneous coronary revascularization* OR coronary atherectom* OR transluminal coronary angioplast* OR transluminal coronary balloon dilation OR coronary balloon angioplast* OR PCI OR Percutaneous Transluminal Coronary Angioplast* OR PTCA OR Rotational Atherectom* OR Directional Coronary Atherectom* OR Balloon dilation OR Artery Stenting)

#4 #2 AND #3

#5 #1 AND #4

PubMed

#1 “Shexiang Tongxin dripping pill”[Title/Abstract] OR “shexiang tongxin”[Title/Abstract] OR “shexiangtongxin”[Title/Abstract]

#2 "myocardial infarction"[MeSH Terms] OR “Acute Coronary Syndrome”[MeSH Terms] OR "myocardial infarct*"[Title/Abstract] OR “Acute Coronary Syndrome”[Title/Abstract] OR “ACS”[Title/Abstract] OR “Acute Coronary Syndrome*”[Title/Abstract]OR “NSTE-ACS”[Title/Abstract] OR “Non–ST-Segment Elevation Acute Coronary Syndrome*”[Title/Abstract] OR "heart infarct*"[Title/Abstract] OR "cardiac infarct*"[Title/Abstract] OR "cardiovascular stroke*"[Title/Abstract] OR "cardiogenic shock"[Title/Abstract] OR “cardiovascular shock” [Title/Abstract] OR “cardiogenic stroke*” [Title/Abstract] OR "heart attack*"[Title/Abstract] OR "STEMI"[Title/Abstract] OR "NSTEMI"[Title/Abstract] OR “ST-segment elevation myocardial infarction”[Title/Abstract] OR “Non-ST-segment elevation myocardial infarction”[Title/Abstract] OR “MINOCA”[Title/Abstract] OR “Myocardial infarction with no obstructive coronary atherosclerosis” [Title/Abstract] OR “xiongbi” [Title/Abstract] OR “xiongbixintong” [Title/Abstract] OR “xiongbi xintong” [Title/Abstract] OR “zhenxintong” [Title/Abstract] OR “cuxintong” [Title/Abstract] OR “Xintong” [Title/Abstract] OR “juexintong” [Title/Abstract]

#3 “percutaneous coronary intervention” [MeSH Terms] OR “percutaneous coronary intervention*”[Title/Abstract] OR “percutaneous coronary revascularization*” [Title/Abstract] OR “coronary atherectom*”[Title/Abstract] OR “transluminal coronary angioplast*”[Title/Abstract] OR “transluminal coronary balloon dilation”[Title/Abstract] OR “coronary balloon angioplast*”[Title/Abstract] OR “PCI”[Title/Abstract] OR “Percutaneous Transluminal Coronary Angioplast*”[Title/Abstract] OR “PTCA”[Title/Abstract] OR “Rotational Atherectom*”[Title/Abstract] OR

“Directional Coronary Atherectom*”[Title/Abstract] OR “Balloon dilation”[Title/Abstract] OR “Artery Stenting”[Title/Abstract]

#4 #2 AND #3

#1 AND #4

Embase

#1 'Shexiang Tongxin dripping pill':ab,ti OR ‘shexiang tongxin’:ab,ti OR ‘shexiangtongxin’:ab,ti

#2 'heart infarction'/exp

#3 'Acute Coronary Syndrome'/exp

#4 'cardiac infarct':ab,ti OR 'cardiac infarction':ab,ti OR 'cardial infarct':ab,ti OR 'heart attack':ab,ti OR 'heart infarct':ab,ti OR 'heart micro infarction':ab,ti OR 'heart muscle infarction':ab,ti OR 'infarction, heart':ab,ti OR 'myocardial infarct':ab,ti OR 'myocardial infarction':ab,ti OR 'myocardium infarct':ab,ti OR 'myocardium infarction':ab,ti OR 'premonitory infarction sign':ab,ti OR 'second heart attack':ab,ti OR 'subendocardial infarction':ab,ti OR 'transmural cardiac infarction':ab,ti OR 'transmural heart infarction':ab,ti OR 'transmural infarction, heart':ab,ti OR 'heart infarction':ab,ti OR ‘Acute Coronary Syndrome’:ab,ti OR ‘ACS’:ab,ti OR ‘Acute Coronary Syndrome*’:ab,ti OR ‘NSTE-ACS’:ab,ti OR ‘Non–ST-Segment Elevation Acute Coronary Syndrome*’:ab,ti OR 'STEMI':ab,ti OR 'NSTEMI':ab,ti OR ‘ST-segment elevation myocardial infarction’:ab,ti OR ‘Non-ST-segment elevation myocardial infarction’:ab,ti OR ‘MINOCA’:ab,ti OR ‘Myocardial infarction with no obstructive coronary atherosclerosis’:ab,ti OR ‘xiongbi’:ab,ti OR ‘xiongbixintong’:ab,ti OR ‘xiongbi Xintong’:ab,ti OR ‘zhenxintong’:ab,ti OR ‘cuxintong’:ab,ti OR ‘Xintong’:ab,ti OR ‘juexintong’:ab,ti OR ‘cardiovascular shock’:ab,ti OR ‘cardiogenic stroke*’:ab,ti

#5 ‘percutaneous coronary intervention'/exp

#6 ‘percutaneous coronary intervention*':ab,ti OR 'percutaneous coronary revascularization*':ab,ti OR 'coronary atherectom*':ab,ti OR 'transluminal coronary angioplast*':ab,ti OR 'transluminal coronary balloon dilation':ab,ti OR 'coronary balloon angioplast*':ab,ti OR 'PCI':ab,ti OR 'Percutaneous Transluminal Coronary Angioplast*':ab,ti OR 'PTCA':ab,ti OR 'Rotational Atherectom*':ab,ti OR 'Directional Coronary Atherectom*':ab,ti OR 'Balloon dilation':ab,ti OR 'Artery Stenting’:ab,ti

#7 #2 OR #3

#8 #4 OR #7

#9 #5 OR #6

#10 #8 AND #9

#11 #1 AND #10

Cochrane

#1 (Shexiang Tongxin dripping pill):ti,ab,kw OR (shexiang tongxin):ti,ab,kw OR (shexiangtongxin):ti,ab,kw

#2 MeSH descriptor: [Myocardial Infarction] explode all trees

#3 MeSH descriptor: [Acute Coronary Syndrome] explode all trees

#4 (myocardial infarct*):ti,ab,kw OR (heart infarct*):ti,ab,kw OR (cardiac infarct*):ti,ab,kw OR (cardiovascular stroke*):ti,ab,kw OR (cardiogenic shock):ti,ab,kw OR (heart attack*):ti,ab,kw OR (Acute Coronary Syndrome):ti,ab,kw OR (ACS):ti,ab,kw OR (Acute Coronary Syndrome*):ti,ab,kw OR (NSTE-ACS):ti,ab,kw OR (Non–ST-Segment Elevation Acute Coronary Syndrome*):ti,ab,kw OR (STEMI):ti,ab,kw OR (NSTEMI):ti,ab,kw OR (ST-segment elevation myocardial infarction):ti,ab,kw OR (Non-ST-segment elevation myocardial infarction):ti,ab,kw OR (MINOCA):ti,ab,kw OR (Myocardial infarction with no obstructive coronary atherosclerosis):ti,ab,kw OR (xiongbi):ti,ab,kw OR (xiongbixintong):ti,ab,kw OR (xiongbi Xintong):ti,ab,kw OR (zhenxintong):ti,ab,kw OR (cuxintong):ti,ab,kw OR (Xintong):ti,ab,kw OR (juexintong):ti,ab,kw OR (cardiovascular shock):ti,ab,kw OR (cardiogenic stroke*):ti,ab,kw

#5 MeSH descriptor: [Percutaneous Coronary Intervention] explode all trees

#6 (percutaneous coronary intervention):ti,ab,kw OR (percutaneous coronary intervention*):ti,ab,kw OR (percutaneous coronary revascularization*):ti,ab,kw OR (coronary atherectom*):ti,ab,kw OR (transluminal coronary angioplast*):ti,ab,kw OR (transluminal coronary balloon dilation):ti,ab,kw OR (coronary balloon angioplast*):ti,ab,kw OR (PCI):ti,ab,kw OR (Percutaneous Transluminal Coronary Angioplast*):ti,ab,kw OR (PTCA):ti,ab,kw OR (Rotational Atherectom*):ti,ab,kw OR (Directional Coronary Atherectom*):ti,ab,kw OR (Balloon dilation):ti,ab,kw OR (Artery Stenting):ti,ab,kw

#7 #2 OR #3

#8 #4 OR #7

#9 #5 OR #6

#10 #8 AND #9

#11 #1 AND #10
