# Supplementary material for: Blood Activating and Stasis Removing Chinese Patent Medicine in Perioperative Period of Percutaneous Coronary Intervention for Myocardial Infarction: A Protocol for a Living Clinical Practice Guideline
Source: Clin Public Health Guidel. 2026 Apr 1;3(2):e70068. doi: 10.1002/gin2.70068 (PMC13563398; doi:10.1002/gin2.70068)
Supplement: Supplementary file 9 [file GIN2-3-e70068-s009.docx]

**Voting Procedures and Decision Criteria for Formulating Recommendations**

**1.Criteria for Determining Recommendation Strength**

- **Strong recommendation:** Benefits clearly outweigh harms, at least 70% experts give a recommendation (strong), and certainty of evidence is moderate or high.
- **Weak recommendation:** Benefits outweigh harms, at least 70% experts give a recommendation (strong + weak).
- **Strong against recommendation:** Harms clearly outweigh benefits, at least 70% experts give a recommendation (strong against), and certainty of evidence is moderate or high.
- **Weak against recommendation:** Harms outweigh benefits, at least 70% experts give a recommendation (strong against + weak against).

**2.Recommendation Development Process**

We will use the nominal group technique (NGT) and the ETD table (Supplementary File 8) to guide the voting process. The structured voting will consider benefits, harms, certainty of evidence, importance of outcomes, feasibility and cost. The GCG will use the ETD table to vote on the Chinese patent medicine, with the following options: "strong recommendation," "weak recommendation," "strong recommendation against," "weak recommendation against," or "unable to make a recommendation."

**Step 1: Evidence Presentation**
The GDG will present the results of the systematic review to experts, reporting on the benefit and harm of each Chinese patent medicine in critical and important outcomes and the certainty of evidence

**Step 2: First Round of Anonymous Expert Voting**

After the host (selected from the methodologists of the GDG) briefly reviews the recommendation strength criteria and outcome importance classification, all experts will be organized to vote anonymously. Each expert will independently rate the strength of recommendation for each Chinese patent medicine. This voting process will be completed independently, without discussion, to ensure that the judgment of experts is not influenced by others' opinions. To ensure consistency and transparency in the voting process, we will organize a pre-voting training session to guide experts to focus on key dimensions such as benefit-risk balance, and provide detailed decision-making criteria for each dimension based on the EtD framework.

**Step 3: Results Summary and Determination of Whether Discussion is Needed**
GDG will summarize and analyze the results of the first round of voting and identify Chinese patent medicines that did not form a clear recommendation. These will be brought to discussion.

**Step 4: Focused Discussion and Second Round of Voting**
If recommendation cannot be formed, the host will guide experts to discuss the following issues:

- Are there any Chinese patent medicines for which the certainty of evidence and effects are controversial or subject to different interpretations, with no consensus among experts?
- Are there any Chinese patent medicines for which, despite insufficient evidence, some experts believe based on clinical experience or theory that they may have potential therapeutic effects, and therefore warrant further consideration?

Experts with differing opinions will present their positions and explain the reasons behind their interpretations, and the methodology expert will quickly clarify evidence, particularly addressing areas where the evidence is controversial. After discussion, a second anonymous vote will be conducted.

If recommendation is still not reached after the second round of voting, the Chinese patent medicine will be considered as unable to make a recommendation and will not be recommended.
