# Supplementary material for: Blood Activating and Stasis Removing Chinese Patent Medicine in Perioperative Period of Percutaneous Coronary Intervention for Myocardial Infarction: A Protocol for a Living Clinical Practice Guideline
Source: Clin Public Health Guidel. 2026 Apr 1;3(2):e70068. doi: 10.1002/gin2.70068 (PMC13563398; doi:10.1002/gin2.70068)
Supplement: Supplementary file 10 [file GIN2-3-e70068-s010.docx]

**Selection Process of Chinese Patent Medicines for Systematic Evidence Retrieval in This Guideline**

**1. Purpose**

To identify Chinese patent medicines for evidence retrieval and evaluation in the guideline, the Guideline Development Group (GDG) conducted a comprehensive selection process with the aim of selecting medicines with blood-activating and stasis-resolving properties, wide clinical use, and relevance to the perioperative management of myocardial infarction (MI) following PCI.

**2. Information Sources**
The selection process referred to the following authoritative sources:

*1. Pharmacopoeia of the People's Republic of China*

2. the *National Essential Medicines List*

3. the *National Medical Insurance Drug List*

*4. Drug Information Platform*

**3. Inclusion Criteria**
The Chinese patent medicines were selected based on the following criteria:

1. Possess blood-activating and stasis-resolving properties and are used for cardiovascular-related conditions;

2. Listed in the authoritative sources mentioned above

To further ensure the scientific and clinical relevance of the selected medicines, the GDG refined the preliminary list based on the *2019 Brand of Chinese Medicine Scientific and Technological Competitiveness Report Summary*. This report assessed Chinese medicines based on brand influence, technological innovation, market performance, and clinical research evidence, and identified Chinese patent medicines with significant competitiveness and widespread application in the cardiovascular field. This filtering process excluded medicines not listed in the report, ensuring that the selected medicines had strong scientific backing and market recognition.

4. **Expert Survey**

To further identify the most commonly used and clinically effective Chinese patent medicines in practice, the GDG distributed structured questionnaires to 576 cardiovascular experts.

This multi-step selection process was necessary due to the unique characteristics of TCM; therefore, integrating regulatory sources (e.g., the Pharmacopoeia and insurance lists), brand competitiveness (to reflect real-world application and innovation capacity), and frontline clinicians’ input (to capture actual clinical practice) helped ensure that the medicines selected for evaluation were not only evidence-informed but also clinically relevant and feasible for guideline recommendations. This comprehensive approach was designed to balance scientific rigor with practical applicability in the context of TCM.

**5.Final List of Selected Medicines**

| No. | Name of Medicine |
| --- | --- |
| 1 | Dazhu Hongjingtian injection |
| 2 | Dazhu Hongjingtian capsule |
| 3 | Danhong injection |
| 4 | Danshen injection |
| 5 | Danshen Dizhuye |
| 6 | Danxiang Guanxin injection |
| 7 | Danlou tablet |
| 8 | Xinyuan capsule |
| 9 | Xinkeshu tablet |
| 10 | Xinnaoning capsule |
| 11 | XinNaoQingRuanJiaoNang |
| 12 | Xinnaoshutong capsule |
| 13 | Xinshu capsule |
| 14 | Kedaling tablet |
| 15 | Lemai granule |
| 16 | Xinxuekang soft capsule |
| 17 | Di'ao Xinxuekang capsule |
| 18 | xuefu zhuyu capsule |
| 19 | Xueshuan Xinmaining Tablet |
| 20 | Dengzhanshengmai capsule |
| 21 | Dengzhanxixin injection |
| 22 | dengzhanxixin granule |
| 23 | Qishen Yiqi dropping pill |
| 24 | Kudiezi injection |
| 25 | Salvianolate injection |
| 26 | breviscapine |
| 27 | Safflower yellow pigment for injection |
| 28 | Xiangdan injection |
| 29 | Compound Danshen tablet |
| 30 | compound Danshen dripping pill |
| 31 | Compound Xueshuantong Capsule |
| 32 | yangxinshi tablet |
| 33 | Guanxin Danshen dropping pill |
| 34 | guanxinshutong capsule |
| 35 | guanxinjing |
| 36 | Suxiao Jiuxin pill |
| 37 | Naoxintong Capsule |
| 38 | Naoxinqing tablet |
| 39 | Yixinshu capsule |
| 40 | NuoDiKangJiaoNang |
| 41 | Tongxinluo capsule |
| 42 | yindan xinnaotong |
| 43 | Ginkgo biloba capsule |
| 44 | Ginkgo leaf drop pill |
| 45 | Ginkgo Ketone Ester Dispersible tablet |
| 46 | Ginkgo Ketone Ester dropping pill |
| 47 | Shexiang Baoxin pill |
