# Supplementary material for: Blood Activating and Stasis Removing Chinese Patent Medicine in Perioperative Period of Percutaneous Coronary Intervention for Myocardial Infarction: A Protocol for a Living Clinical Practice Guideline
Source: Clin Public Health Guidel. 2026 Apr 1;3(2):e70068. doi: 10.1002/gin2.70068 (PMC13563398; doi:10.1002/gin2.70068)
Supplement: Supplementary file 11 [file GIN2-3-e70068-s011.docx]

**The details of subgroup analyses**

| Subgroup | Subgroup level | Hypothesis for the direction of subgroup effect |
| --- | --- | --- |
| Myocardial Infarction Type | 1. ST-segment elevation myocardial infarction  2. Non-ST-segment elevation myocardial infarction | Hypothesis 1: The effect will be larger in people with ST-segment elevation than in people without ST-segment elevation |
| Dosage Form | 1. Pill  2. Tablets  3. Injections  4. Capsules | Hypothesis 1: At least two dosage forms of the same Chinese patent medicine have different effects. |
| Traditional Chinese Medicine Syndrome Type | 1. Qi Deficiency and Blood Stasis  2. Phlegm-blood Stasis Mutual Binding  3. Qi Stagnation and Blood Stasis  4. Cold Congealing the Heart Vessels  5. Qi and Yin Deficiency  6. Syndrome of Qi Deficiency and Yang Collapse | Hypothesis 1: The effects of the same Chinese patent medicine differ for at least two Traditional Chinese Medicine Syndrome types. |

**Reference**

1. Ryan TJ, Faxon DP, Gunnar RM, Kennedy JW, King SB 3rd, Loop FD, et al. Guidelines for percutaneous transluminal coronary angioplasty. A report of the American College of Cardiology/American Heart Association Task Force on Assessment of Diagnostic and Therapeutic Cardiovascular Procedures (Subcommittee on Percutaneous Transluminal Coronary Angioplasty). Circulation. 1988;78(2):486-502. [PubMed] (https://pubmed.ncbi.nlm.nih.gov/30153967)
2. Killip T, 3rd, Kimball JT. Treatment of myocardial infarction in a coronary care unit. A two year experience with 250 patients. Am J Cardiol 1967;20(4):457-64. doi: 10.1016/0002-9149(67)90023-9 [PubMed] (https://pubmed.ncbi.nlm.nih.gov/6059183)
3. Campeau L. Letter: Grading of angina pectoris. Circulation 1976;54(3):522-3. [PubMed] (https://pubmed.ncbi.nlm.nih.gov/11992130)

4. https://professional.heart.org/en/guidelines-and-statements/classification

5. [Doctor Society of Integrative Medicine, Chinese Medical Doctor Association](javascript:void(0)), Intensive Care Medicine Professional Committee of the Chinese Society of Integrative Medicine, et al. Diagnosis and treatment Guideline of Integrative Chinese and Western Medicine for Acute Myocardial Infarction. Zhongguo Zhong Xi Yi Jie He Za Zhi 2018;38(03):272-84
