# Supplementary material for: Blood Activating and Stasis Removing Chinese Patent Medicine in Perioperative Period of Percutaneous Coronary Intervention for Myocardial Infarction: A Protocol for a Living Clinical Practice Guideline
Source: Clin Public Health Guidel. 2026 Apr 1;3(2):e70068. doi: 10.1002/gin2.70068 (PMC13563398; doi:10.1002/gin2.70068)
Supplement: Supplementary file 12 [file GIN2-3-e70068-s012.docx]

Evidence Summary Table

| Outcome | Evidence Source | Intervention Group | Control  Group | Number of Studies | Events in Intervention Group / Total | Events in Control Group / Total | Heterogeneity | | | RR（95% CI） | Baseline Risk | RD*1000 | NNTnet | Final Certainty of Evidence |
| --- | --- | --- | --- | --- | --- | --- | --- | --- | --- | --- | --- | --- | --- | --- |
|  |  |  |  |  |  |  | I^2^ | Q | *P* |  |  |  |  |  |
| MACE at one month | Direct comparison | Standard treatment+Compound Danshen dripping pill | Standard treatment | X | XX/XXX | XX/XXX | X | X | X | XX (XX, XXX) | XX per 1000 | XX fewer (XX fewer, XX more) |  | High/moderate/low/very low |
| Major bleeding at one month | Indirect comparison | Standard treatment+Danhong injection | Standard treatment+Tongxinluo capsule | X | XX/XXX | XX/XXX | X | X | X | XX (XX, XXX) | XX per 1000 | XX fewer (XX fewer, XXX more) |  | High/moderate/low/very low |
